# Neoadjuvant PD1 plus CTLA-4 immune checkpoint blockade in surgically resectable recurrent glioblastoma: a randomized surgical window-of-opportunity trial

**DOI:** 10.1038/s41467-026-77433-z

**Published:** 2026-09-05

**Authors:** Lu Sun, Lauren F. Markus, Julio C. Sanchez, Thomas J. Lai, Eudocia Quant Lee, Gang Li, Jiyoon Kim, Zian Zhuang, Brenda Acevedo, Richard G. Everson, Ingo K. Mellinghoff, Elisa Aquilanti, L. Nicolas Gonzalez Castro, J. Ricardo McFaline-Figueroa, Lakshmi Nayak, Rameen Beroukhim, Robert A. Chong, Jacqueline Stone, Thomas Kaley, Lauren Schaff, Jessica Wilcox, Catharina Westergaard, Alyssa Russ, Maria Lavallee, Ugonma Chukwueke, Annick D. Van den Abbeele, Michael Lim, Benjamin M. Ellingson, David A. Reardon, Timothy F. Cloughesy, Robert M. Prins, Patrick Y. Wen

**Affiliations:** 1 Department of Neurosurgery, David Geffen School of Medicine, University of California Los Angeles, Los Angeles, CA USA; 2 Center for Neuro-Oncology, Dana-Farber Cancer Institute, Boston, MA USA; 3 Department of Biostatistics, Jonathan and Karin Fielding School of Public Health, University of California Los Angeles, Los Angeles, CA USA; 4 Jonsson Comprehensive Cancer Center, University of California Los Angeles, Los Angeles, CA USA; 5 Department of Neurology, Memorial Sloan Kettering Cancer Center, New York, NY USA; 6 Human Oncology and Pathogenesis Program, Memorial Sloan Kettering Cancer Center, New York, NY USA; 7 Massachusetts General Brigham Cancer Institute, Boston, MA USA; 8 Brain & Spine Tumor Center, Perlmutter Cancer Center, NYU Langone Health, New York, NY USA; 9 Department of Neurology, David Geffen School of Medicine, University of California Los Angeles, Los Angeles, CA USA; 10 Department of Imaging, Dana-Farber Cancer Institute, Boston, MA USA; 11 Department of Radiology, Brigham and Women’s Hospital, Harvard Medical School, Boston, MA USA; 12 Department of Neurosurgery, Stanford University, Palo Alto, CA USA; 13 UCLA Brain Tumor Imaging Laboratory, Department of Radiological Sciences, David Geffen School of Medicine, University of California Los Angeles, Los Angeles, CA USA; 14 Department of Medical and Molecular Pharmacology, David Geffen School of Medicine, University of California Los Angeles, Los Angeles, CA USA; 15 Parker Institute for Cancer Immunotherapy, San Francisco, CA USA

**Keywords:** Cancer immunotherapy, CNS cancer, Immunosurveillance

## Abstract

We conducted a randomized surgical window-of-opportunity trial (NCT04606316) in recurrent, resectable glioblastoma. Between 2021 and 2024, 71 patients were screened, and 63 were randomized (intention-to-treat [ITT] population), and 58 received study treatment. Patients received pre-surgical immune checkpoint blockade (ICB) with dual anti-PD1 nivolumab + anti-CTLA4 ipilimumab (Arm 1), nivolumab alone (Arm 2), or placebo (Arm 3). Following surgery, Arms 1 and 3 received dual ICB, while Arm 2 continued nivolumab until progression or unacceptable toxicity. The primary endpoint, tumor-infiltrating lymphocyte (TIL) density, was met for Arm 1, as neoadjuvant dual ICB significantly increased TIL density compared with untreated control (Arm 3). As a secondary endpoint, median overall survival in the ITT population was 402 days (95% CI, 265–571) among patients who received dual ICB (Arms 1 and 3) and 273 days (95% CI, 166–506) for those assigned to nivolumab alone (Arm 2). No unanticipated toxicities were observed. Exploratory analyses showed that dual ICB elicited robust intratumoral and systemic immune activation, including increased interferon-related gene expression in blood. Higher TIL density and early systemic interferon-signature induction were associated with improved survival, whereas tumor mutational burden was not. Our results demonstrate pharmacodynamic activity of dual ICB in glioblastoma, with survival outcomes comparing favorably to similar studies.

## Abstract (web summary)

Here the authors report clinical and immunological outcomes of a randomized surgical window-of-opportunity trial designed to test whether dual immune checkpoint blockade targeting PD-1 and CTLA-4 could overcome the adaptive immune resistance previously reported with single-agent anti-PD-1 therapy in patients with surgically resectable recurrent glioblastoma.

## Introduction

Glioblastoma is the most aggressive primary brain cancer in adults and two-thirds of patients are inherently resistant to the current standard-of-care systemic therapies^1^. Despite the impressive intracranial activity of immune-checkpoint blockade (ICB) in pathologies such as melanoma brain metastases^2–6^, glioblastoma has been largely resistant to single-agent immune checkpoint blockade, except in isolated case reports associated with mismatch repair deficiency^7–10^. Pre-clinical studies, however, have argued that the PD-1/PD-L1 axis is immunologically relevant and suggest the existence of a therapeutic window^11–16^. We previously reported multiple surgical window-of-opportunity trials of neoadjuvant followed by adjuvant PD-1-directed ICB in patients with recurrent glioblastoma undergoing surgical resection^17–19^. Bulk gene expression analysis comparing neoadjuvant-pretreated versus untreated tumors uncovered a molecular response signature that consisted of downregulation of cell cycle/cancer proliferation genes and upregulation of T cell/interferon-γ-related genes. Cell cycle gene signatures were inversely correlated with overall survival^17^. Importantly, the addition of neoadjuvant anti-PD-1 ICB was associated with the induction of adaptive immune resistance within the tumor, such as the CTLA-4 co-inhibitory pathways^18,19^.

In this work, we show that dual ICB targeting PD-1 and CTLA-4 induces more effective anti-tumor immune responses in surgically resectable recurrent GBM patients. Using T cell receptor sequencing, gene expression profiling and quantitative multiplex immunofluorescence, we define the pharmacodynamic effects of PD-1 and CTLA-4 dual blockade and demonstrate the importance of intratumoral and systemic immune activation in shaping dual ICB responsiveness.

## Results

### Patient characteristics

**Fig. 1:**
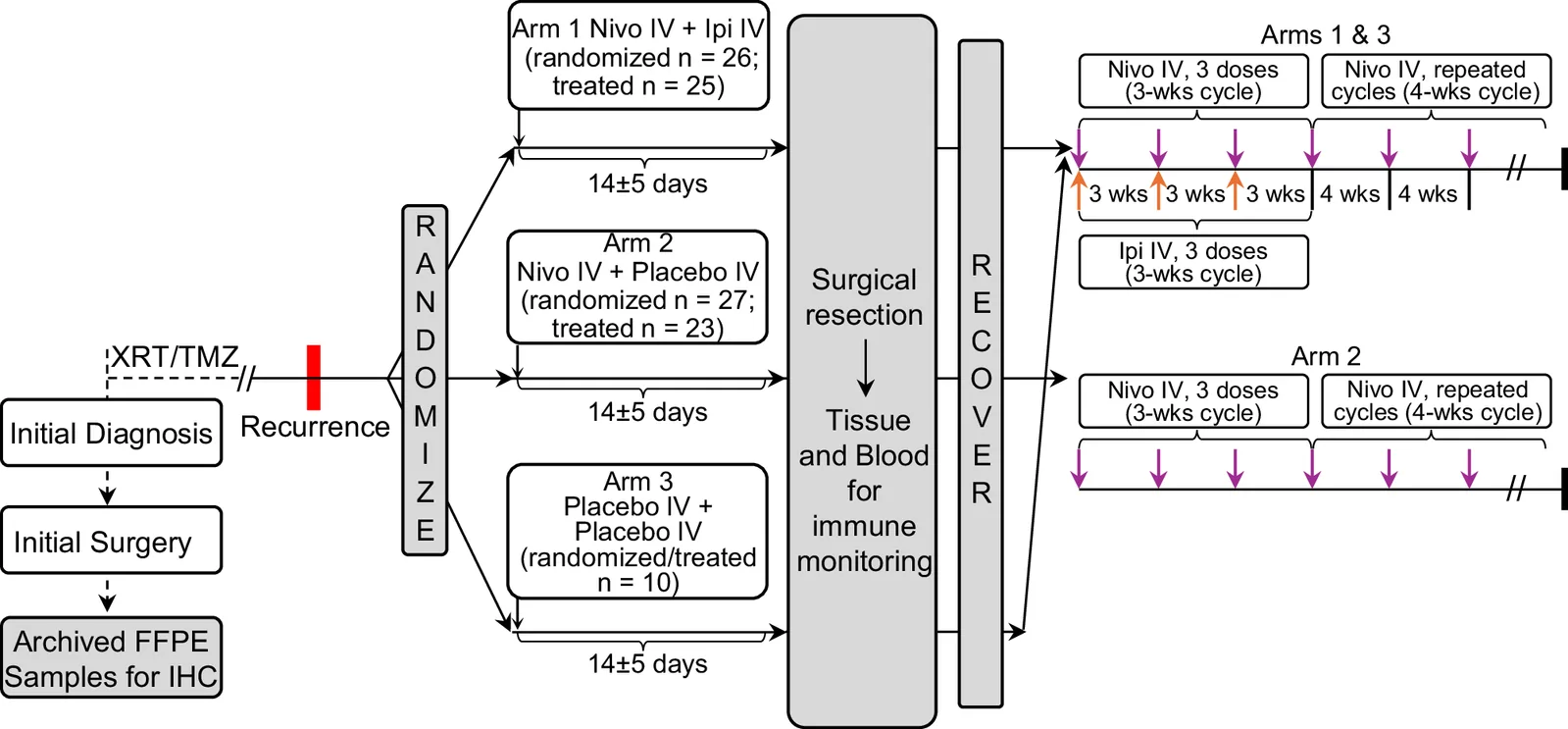
Trial design. Nivo, Nivolumab. Ipi, Ipilimumab. Numbers in the arm boxes indicate randomized assignment. Five randomized patients withdrew consent before treatment initiation and did not receive study therapy: Arm 1, *n* = 1; Arm 2, *n* = 4. The treated population included 58 patients.

We performed a surgical window-of-opportunity trial in relapsed, resectable glioblastoma patients. Seventy-one patients were screened and randomized during a three-year period between January of 2021 and 2024 at four institutions and comprise the intention-to-treat (ITT) population (Supplementary Data 1). Thirteen of these patients did not initiate study therapy including one patient who did not meet eligibility criteria (IDH status) and five patients who elected to withdraw consent after randomization but prior to initiating study therapy. Figure 1 depicts the trial design including treatment arms of the 58 patients who received study therapy. Prior to surgical resection, patients received either dual PD-1 plus CTLA-4 ICB (Arm 1, *n* = 25) with ipilimumab (3 mg/kg) and nivolumab (240 mg/kg), single-agent PD-1 ICB (Arm 2, *n* = 23) with nivolumab (240 mg/kg), or a placebo infusion (Arm 3, *n* = 10). Following surgery, patients in Arms 1 and 3 received dual ICB, whereas patients in Arm 2 still received single-agent nivolumab until disease progression or unacceptable toxicity (Supplementary Note). The use of single-agent nivolumab for post-surgical treatment in Arm 2 was based on our prior study suggesting that neoadjuvant + adjuvant single-agent anti-PD-1 therapy (with pembrolizumab) may be associated with improved survival in recurrent glioblastoma^17^. However, our subsequent validation trial did not confirm a survival benefit with neoadjuvant and adjuvant single-agent pembrolizumab^19^. Considering these findings, the protocol for the current ipilimumab + nivolumab trial was amended at the very end of the trial to treat the remaining Arm 2 patients with dual ICB after surgery. As this amendment occurred late in the trial, it affected only a small subset of patients: specifically, 3 of 23 patients in Arm 2 received neoadjuvant nivolumab followed by adjuvant ipilimumab + nivolumab, whereas the remaining 20 patients received single-agent nivolumab both neoadjuvantly and adjuvantly.

Baseline patient characteristics are shown by treatment group in Table 1. All patients were confirmed isocitrate dehydrogenase (IDH) wild-type GBM. In this patient cohort, no statistically significant differences were observed in age, sex, Karnofsky performance status (KPS), or MGMT methylation status between treatment arms (Table 1 and Supplementary Table 1). Although Arm 2 included a slightly higher number of patients receiving steroid therapy at registration, this difference was not statistically significant. Arm 2 also exhibited the largest and most variable postoperative residual enhancing tumor volumes, whereas Arm 3 had the smallest volumes with minimal variability. However, pairwise permutation testing (1 vs 2, 1 vs 3, and 2 vs 3) demonstrated that none of these comparisons reached statistical significance (Supplementary Fig. 1).

**Table 1:** Patient and tumor characteristics by treatment group

	Arm 1 (*n* = 26)	Arm 2 (*n* = 27)	Arm 3 (*n* = 10)
Age at enrollment, years, mean ± s.d.	58.5 ± 10.7	59.4 ± 7.9	54.7 ± 4.9
**Gender,** ***n* (%)**			
Male	16 (62)	13 (48)	4 (40)
Female	10 (38)	14 (52)	6 (60)
Karnofsky performance status, mean ± s.d.	88 ± 7.6	87 ± 5.8	89 ± 5.4
**Steroid use at registration**			
Patients receiving steroids (*n*)	2	6	3
Daily dosage, median, mg/day (range)	0 (0–10)	0 (0–8)	0 (0–8)
**MGMT status,** ***n* (%)**			
Methylated	9 (35)	12 (44)	1 (10)
Unmethylated	17 (65)	15 (56)	9 (90)
Postoperative tumor volume (mL), mean ± s.e.m.	4.1 ± 1.8	9.3 ± 2.8	1.4 ± 0.5
Tumor mutational burden, mean ± s.d.	3.8 ± 2.0	4.9 ± 2.2	5.5 ± 2.9
**PTEN status,** ***n* (%)**			
Loss	13 (50)	16 (59)	6 (60)
WT	8 (31)	3 (11)	2 (20)
Unknown	5 (19)	8 (30)	2 (20)
**EGFR status,** ***n*** **(%)**			
Gain of function	14 (54)	12 (44)	5 (50)
WT	8 (31)	8 (30)	3 (30)
Unknown	4 (15)	7 (26)	2 (20)
**TERT status,** ***n* (%)**			
Gain of function	17 (65)	15 (56)	7 (70)
WT	4 (15)	5 (19)	1 (10)
Unknown	5 (19)	7 (26)	2 (20)

For steroid use at registration, most patients received dexamethasone. For the two patients receiving prednisone 50 mg/day, their prednisone doses were converted to the corresponding dexamethasone-equivalent amounts. Arm 1: Neoadjuvant Nivo + Ipi; Arm 2: Neoadjuvant Nivo alone; Arm 3: Placebo infusion prior to surgery.

*s.d.* standard deviation, *s.e.m.* standard error of the mean.

### Safety

Nivolumab and/or ipilimumab were generally well tolerated (Supplementary Data 2); the neoadjuvant and adjuvant administration of dual or single ICB was not associated with any unanticipated toxicities in this patient population. Eleven patients in arm 1, 5 patients in arm 2, and 6 patients in arm 3 experienced grade 3–4 adverse events that were deemed possible or likely attributable to the study drug(s). However, no patients in any group had surgery delayed due to adverse events prior to tumor resection. One patient experienced toxicities leading to the discontinuation of ipilimumab. The most common treatment-emergent toxicities were lymphopenia (48%), transaminitis (43%), and muscle weakness (33%). Such treatment-related adverse events are commonly seen in patients with central nervous system tumors and/or those who have received corticosteroids; indeed, several of these adverse events were deemed unlikely to be related to the study drug by the site investigator but were included for completeness of data reporting.

### Dual ICB induces significant upregulation of interferon-related gene expression in tumor tissue compared with PD-1 mAb alone or placebo

**Fig. 2:**
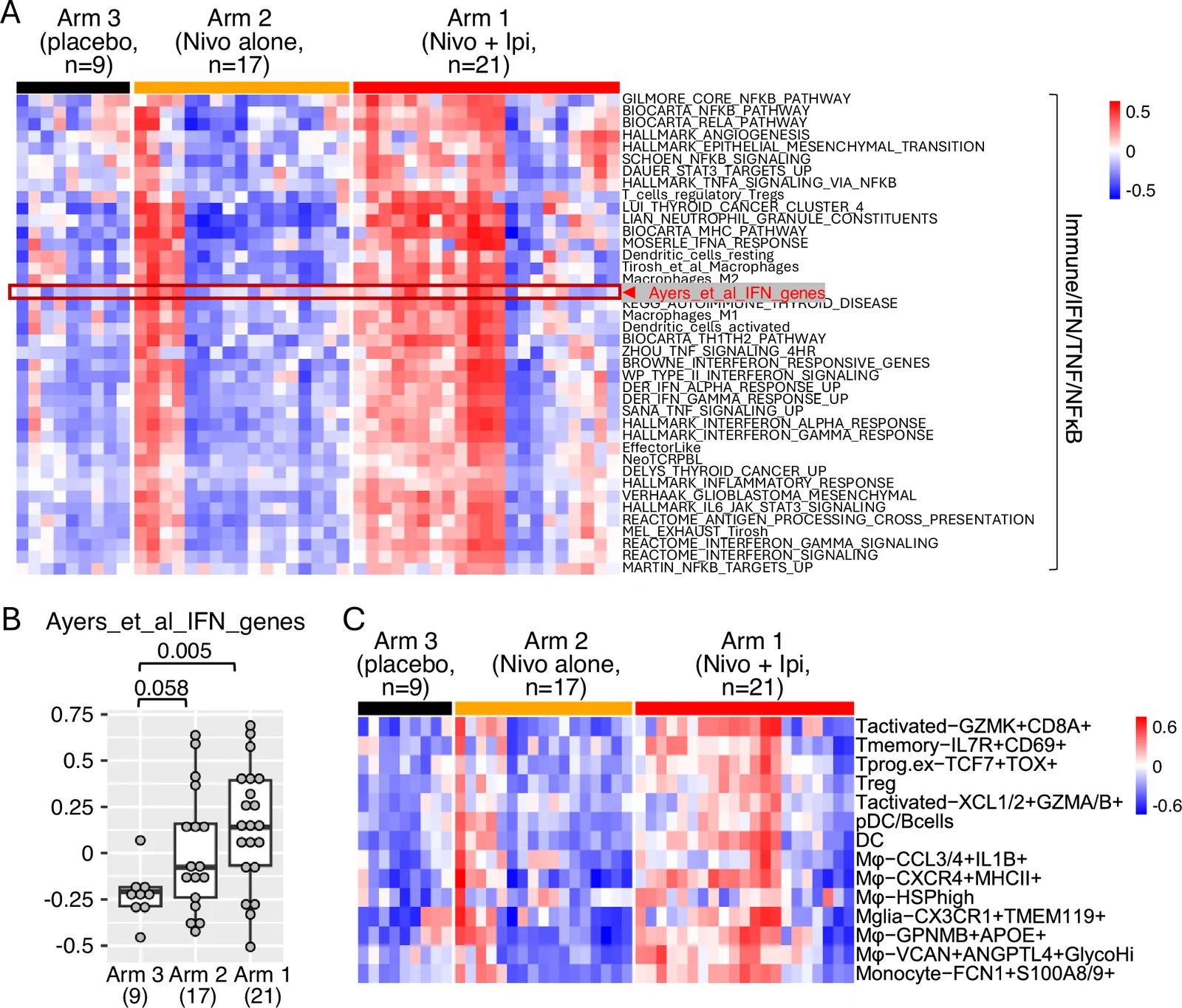
Neoadjuvant administration of PD-1+/- CTLA-4 mAb induces interferon and inflammatory changes within the TME. **A** Heatmap of bulk tumor mRNA expression from 47 patients with RNA‑seq data, displaying GSVA scores for gene signatures significantly enriched in Arm 1 compared with Arm 3. **B** Boxplot of GSVA scores for the representative interferon/T‑cell gene signature Ayers_et_al_IFN_genes across three treatment arms. Arm 1: Neoadjuvant Nivo + Ipi; Arm 2: Neoadjuvant Nivo alone; Arm 3: Placebo infusion prior to surgery. **C** Heatmap of GSVA scores for the lymphoid and myeloid subset-specific gene signatures across Arms 3, 2 and 1. For the boxplot, the values in parentheses indicate the number of patients, each dot represents a patient, the lower and upper bounds indicate the 25th and 75th percentiles, and the middle lines the median values. *P* values were calculated using a 2-sided Wilcoxon’s rank-sum test. Source data are provided as a Source data file.

In an exploratory analysis, we evaluated the gene expression profiles within the tumor microenvironment of all patients treated on this study using bulk RNA sequencing (RNA-seq). Unbiased gene set variation analysis (GSVA) identified a set of interferon and inflammatory signatures that were significantly altered by neoadjuvant ICB (Supplementary Data 3). Relative to the placebo arm, the extent of upregulation was greatest with dual ipilimumab+nivolumab compared to nivolumab alone (Fig. 2A, B and Supplementary Fig. 2A). In addition, we leveraged our previously published single-cell RNAseq data from tumor-infiltrating immune cells from recurrent GBM treated with or without neoadjuvant anti-PD-1^18^; we derived gene signatures for distinct lymphoid and myeloid subsets. Applying these signatures to the current bulk RNA-seq data revealed consistent enrichment of multiple T cell and myeloid populations in Arm A (ipi+nivo) compared with Arm C (placebo) and/or Arm B (nivo only) (Fig. 2C; Supplementary Fig. 2B). Such intratumoral findings suggest that dual neoadjuvant ICB elicits a more robust intratumoral immune response, characterized by enhanced immune cell infiltration and activation within the tumor microenvironment.

### Tumor-infiltrating T cell density is independently associated with survival benefit and directly linked to the extent of interferon gene upregulation

**Fig. 3:**
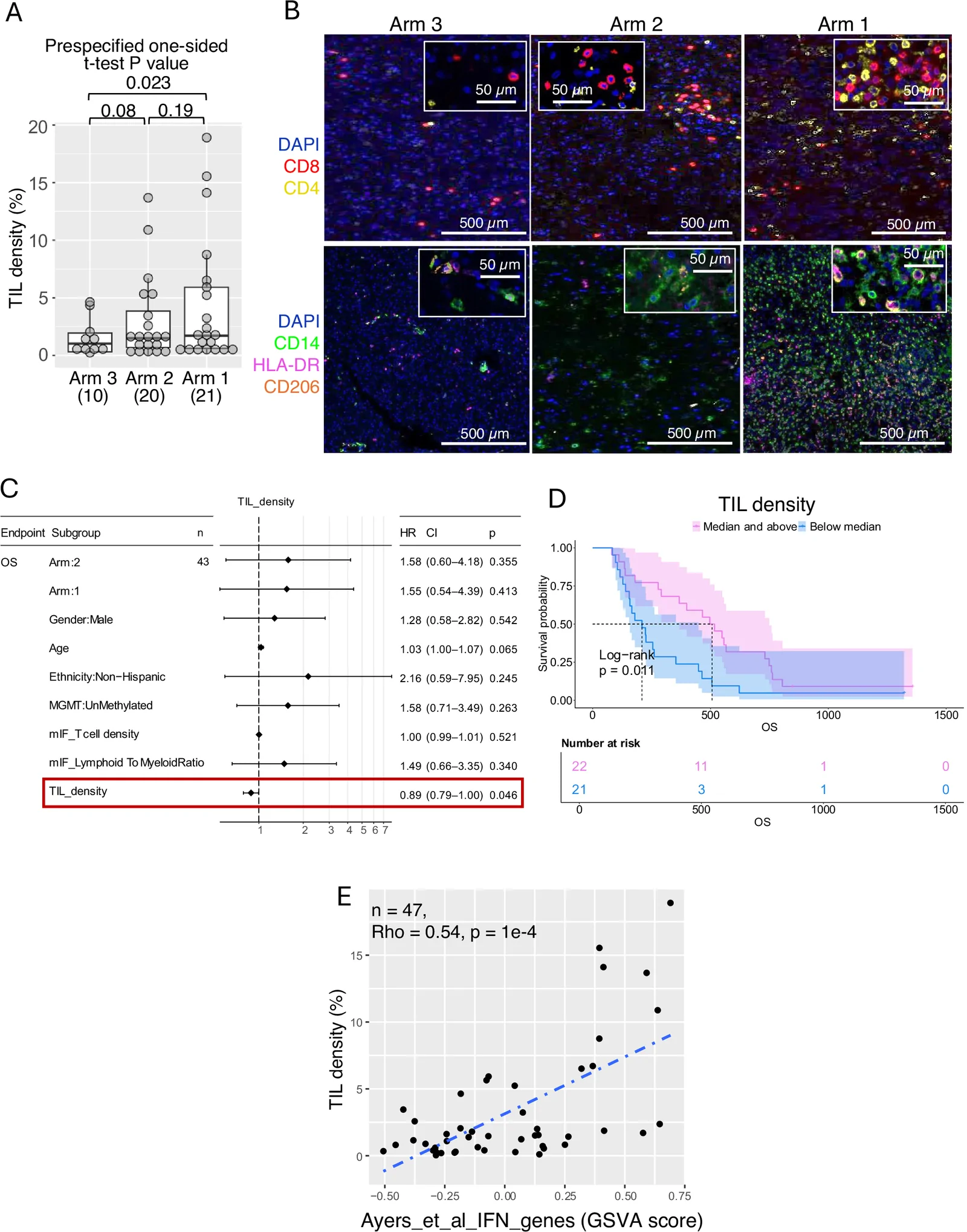
TIL density independently predicts survival and correlates with interferon gene expression. **A** Boxplot of TIL density from 51 patients with TCR‑seq-based TIL density estimates across three neoadjuvant treatment arms. The values in parentheses indicate the number of patients. Each dot represents a patient. The lower and upper bounds indicate the 25th and 75th percentiles, and the middle lines the median values. One-sided Welch’s t-test was used to calculate the *P* values. Arm 1: Neoadjuvant Nivo + Ipi; Arm 2: Neoadjuvant Nivo alone; Arm 3: Placebo infusion prior to surgery. **B** Representative mIF images (from one patient per arm) showing immune cell distribution across treatment arms. **C** Forest plot of Cox proportional hazards (Cox-PH) model assessing TIL density and clinical covariates. *n* = 43 indicates the number of patients with available clinical covariates, mIF data, and TCR‑seq-based TIL density. Diamonds at the centers of the error bars indicate hazard ratio (HR) estimates, error bars denote 95% confidence intervals (CI), and *P* values are based on two-sided Wald statistics (without adjustment for multiple comparisons). **D** Kaplan–Meier curves illustrating the impact of TIL density on overall survival. Log-rank *P* value is shown. **E** Scatter plot showing correlation between TIL density and GSVA scores for Ayers_et_al_IFN_genes signature (Spearman correlation coefficient Rho and two-sided *P* value shown). Source data are provided as a Source data file.

For the primary outcome, we measured the density of alpha beta T cell receptor (TCRαβ)^+^ lymphocytes using TCR sequencing from the tumor tissue of these patients. Neoadjuvant dual ICB (Arm 1) induced the highest tumor-infiltrating lymphocyte (TIL) density, which was significantly greater than in Arm 3 (prespecified one-sided Welch’s t-test, *p* = 0.023; Fig. 3A). We also performed multiplexed immunofluorescent (mIF) staining for CD45, CD4, CD8, the pan-myeloid marker CD14, the perivascular macrophage marker CD206, and the pro-inflammatory macrophage marker HLA-DR (Fig. 3B, Supplementary Fig. 3A, Supplementary Data 4). Although these differences did not reach statistical significance, both CD4⁺ and CD8⁺ T cell densities were highest in Arm 1, consistent with the TCR-based TIL density quantification. Notably, Arm 1 also exhibited the highest density of monocyte-derived macrophages (Supplementary Fig. 3A), suggesting increased overall immune infiltration following dual ICB. Among these macrophages, the density of HLA-DR⁺ macrophages was highest in Arm 1 (Supplementary Fig. 3A). Given that interferon-γ is a known inducer of HLA-DR expression on myeloid cells, this observation is concordant with the elevated interferon signaling observed in Arm 1 by bulk RNA-seq.

Multivariate analysis identified TIL density as an independent predictor of improved overall survival (Fig. 3C, D) and four of the five long-term survivors (> 3 years without progression) had a TIL density above the median (Supplementary Fig. 3B). On a per-patient basis, TIL density correlated with the GSVA scores of interferon-related gene expression (Fig. 3E); however, interferon signatures alone were not associated with survival (Supplementary Fig. 4A).

To further dissect the cellular contributors to IFN signaling, we correlated the interferon signature (Ayers_et_al_IFN_genes) with immune subset-specific signatures as defined in Fig. 2C. GZMK⁺CD8A⁺ T cells, dendritic cells, and monocytes showed the strongest association, while multiple macrophage subsets showed more modest but still notable link (Supplementary Fig. 4B and Supplementary Data 5). These findings indicate that the elevated IFN signal reflects contributions from both lymphoid and myeloid compartments. Notably, myeloid-derived IFN signaling may also contribute to adaptive immune resistance following ICI treatment^18^, potentially explaining the lack of association between IFN signatures and survival.

Unbiased screening of the Molecular Signatures Database (MSigDB) and curated immune signatures revealed strong correlations between survival and pathways linked to T-cell activation (e.g., TCR and IL-2 signaling; Supplementary Fig. 4C and Supplementary Data 6), highlighting the importance of intratumoral T-cell quantity and quality for treatment response and survival outcomes. Consistent with our prior neoadjuvant pembrolizumab trial^17^, increased cell cycle and proliferation signatures were negatively associated with survival (Supplementary Fig. 4D). Tumor mutational burden (TMB), previously linked to immunotherapy response in other cancers^20^, did not differ between treatment arms and showed no survival association (Supplementary Fig. 4E); consistent with recent findings from other studies in GBM^21–23^. Smaller postoperative residual enhancing tumor volume was associated with improved OS (Supplementary Fig. 4F). Our group previously demonstrated that postoperative tumor volume is a prognostic factor for survival in newly diagnosed GBM patients treated with chemoradiation^24^. The current study extends this observation to recurrent GBM patients receiving immunotherapy, supporting its role as a robust prognostic indicator for GBM patients across disease settings. We also examined three key genes frequently altered in glioblastoma—EGFR, PTEN, and TERT promoter—using clinically validated next-generation sequencing platforms (FoundationOne, OncoPanel, or MSK-IMPACT) (Supplementary Data 1). The frequency of these alterations did not differ significantly across treatment arms. However, EGFR gain-of-function variants (e.g., amplification or activating mutations such as EGFRvIII) and TERT gain-of-function changes (promoter mutation or amplification) were each independently associated with worse overall survival (Supplementary Fig. 5A, B), while PTEN loss also demonstrated a trend toward association with poorer survival but did not reach statistical significance (Supplementary Fig. 5C). Overall, our findings strongly suggest that neoadjuvant administration of dual PD-1 and CTLA-4 blockade induces robust T cell-driven pharmacodynamic changes in the tumor immune microenvironment; dual ICB-induced TIL density, but not TMB, is independently associated with a survival benefit.

Utilizing our TCR sequencing data, we asked whether TCR polyclonality differed across the three arms. While not statistically significant, Arm 1 (dual ICB) showed trends toward higher clonality (1 − normalized Shannon entropy), greater diversity (Gini–Simpson index), and increased clone richness (number of unique clonotypes) compared with the other arms (Supplementary Fig. 6A). Notably, the Gini–Simpson index provides a complementary, non-redundant measure to Shannon entropy (i.e., it is not simply the inverse of clonality)^25^, as it captures the contribution of multiple expanded clones rather than being driven by one or a few dominant clones. Thus, the concurrent increase of clonality, diversity and clone richness suggest that dual ICB induces a focused yet polyclonal T cell response in the tumor: increased T cell infiltration (higher richness), a substantial contribution from multiple clonotypes (higher Gini–Simpson diversity), and preferential expansion of selected clones (higher clonality). Higher TCR diversity (Gini–Simpson index) was associated with improved survival outcomes, although this did not reach statistical significance in Cox proportional hazards models (Supplementary Fig. 6B). Importantly, when combined with TIL density, patients with both high TIL density and higher TCR diversity exhibited significantly improved survival (Supplementary Fig. 6C), suggesting that both the abundance and diverse profile of intratumoral TCR repertoire are needed for a productive T cell-driving tumor killing activity^26^. For each arm, we analyzed the top expanded clones and observed minimal sharing of TCR sequences across patients (Supplementary Fig. 6D), indicating that the expanded T cell responses are largely private and likely targeting patient-specific antigens.

### Extended and chronic inflammatory gene expression signaling in peripheral blood predicts early progression and death

**Fig. 4:**
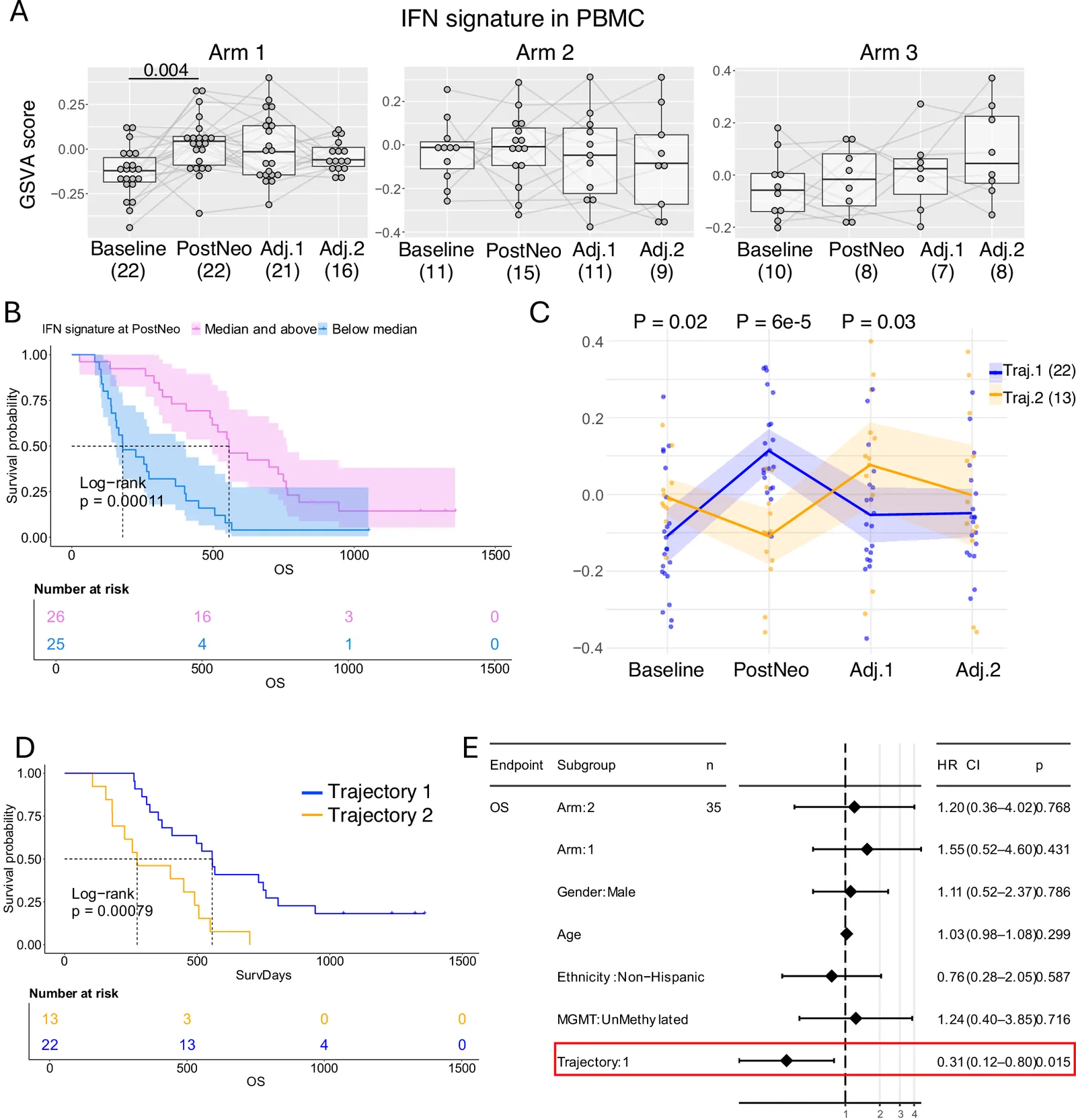
Extended and chronic inflammatory gene expression signaling in peripheral blood predicts early progression and death. **A** Boxplots of GSVA scores for a representative IFN gene signature (HALLMARK_INTERFERON_GAMMA_RESPONSE) in PBMCs across four time points: Baseline, PostNeo, Adjuvant Cycle 1 (Adj.1), and Cycle 2 (Adj.2). The values in parentheses indicate the number of patients. Each dot represents a patient. The lower and upper bounds indicate the 25th and 75th percentiles, and the middle lines median values. For patients with PBMC RNA-seq data at more than one time point, connecting lines illustrate the temporal trajectory for each patient. *P*-values were calculated using a two-sided Wilcoxon rank-sum test. Arm 1: Neoadjuvant Nivo + Ipi; Arm 2: Neoadjuvant Nivo alone; Arm 3: Placebo infusion prior to surgery. **B** Kaplan–Meier curves showing the association between HALLMARK_INTERFERON_GAMMA_RESPONSE signature scores in PBMCs at PostNeo and overall survival. **C** Line graphs depicting temporal HALLMARK_INTERFERON_GAMMA_RESPONSE signature patterns for Trajectory 1 vs. Trajectory 2. The values in parentheses indicate the number of patients with PBMC RNA-seq data at Baseline, PostNeo, and at least one Adjuvant time points. Dots represent individual patients; solid lines indicate mean values; shaded areas denote standard deviation. *P*-values comparing GSVA scores between the two trajectories at each time point were calculated using a two-sided Wilcoxon rank-sum test. **D** Kaplan–Meier curves comparing overall survival between Trajectory 1 and Trajectory 2. **E** Forest plot of Cox-PH models evaluating Trajectory 1 and clinical covariates. *n* = 35 indicates the number of patients having PBMC RNA-seq data at Baseline, PostNeo, and at least one Adjuvant time points. Diamonds at the centers of the error bars indicate HR estimates; error bars indicate 95% CI; *P*-values are based on two-sided Wald statistics (without adjustment for multiple comparisons). Source data are provided as a Source data file.

Although we identified TIL density as an independent correlate of improved survival, its impact appears less pronounced among long-term survivors (Fig. 3D and Supplementary Fig. 3B). This observation suggests that factors beyond intratumoral T-cell abundance—particularly longitudinal dynamics—likely contribute to durable outcomes. To explore if temporal biomarkers could be identified and associated with prolonged survival, we performed RNA-seq on patient derived peripheral blood mononuclear cells (PBMCs) that were collected at different time points; at baseline (pre-treatment), after neoadjuvant dosing (PostNeo), and after the first and second adjuvant ICB doses (Adjuvant cycles; see Fig. 1). We first asked whether the three treatment arms could be distinguished by gene expression changes. Consistent with our intratumoral findings, patients in Arm 1 who received dual ICB pre-surgery exhibited a clear induction of interferon-response genes following neoadjuvant administration (Fig. 4A; Supplementary Fig. 7A; Supplementary Data 7). In contrast, Arm 2 (aPD-1 alone) showed no such induction, while Arm 3 (placebo pre-surgery, dual ICB post-surgery) displayed a delayed trend toward IFN signature activation only during adjuvant cycles. Of note, we could not resolve whether this response reflected type I or type II interferon signaling, as the gene sets overlap substantially (Supplementary Fig. 7A). Interestingly, the Ayers et al. IFN gene signature, which is robustly induced intratumorally by dual ICB (Fig. 2B), was not significantly elevated in PBMCs (Supplementary Fig. 7A), likely because this signature was derived from intratumoral profiles^27^. To further characterize systemic immune changes, we leveraged our previously published PBMC single-cell RNA-seq dataset from recurrent GBM^18^ as a reference and applied CIBERSORTx to deconvolute bulk RNA-seq data and infer temporal changes in immune cell composition. This analysis revealed increases in CD14⁺ monocytes and proliferating T cells following neoadjuvant dual ICB in Arm 1 (Supplementary Fig. 7B), although these changes did not reach statistical significance. We next correlated longitudinal changes in IFN signature scores (Δ from baseline to post-neoadjuvant or adjuvant cycles 1 and 2) with corresponding shifts in inferred immune cell fractions. Across all compartments, CD14⁺ monocytes consistently showed the strongest positive correlations with IFN signaling (Supplementary Fig. 7C), suggesting a central role for circulating monocytes in sensing and responding to systemic inflammatory cues induced by therapy. Importantly, a high IFN signature score at the post neoadjuvant time point was significantly associated with improved survival, suggesting that systemic IFN activation pre-surgery reflects productive anti-tumor immunity (Fig. 4B; Supplementary Data 8). To better understand how each patient’s IFN response changed over the course of treatment, we examined the overall pattern of an established IFN‑related gene signature (HALLMARK_INTERFERON_GAMMA_RESPONSE)^28^ at four time points (Baseline, PostNeo, and Adjuvant Cycles 1 and 2). We leveraged patients who had PBMC RNA-seq data and therefore IFN signature scores—available at Baseline, PostNeo, and at least one Adjuvant time point to construct temporal trajectories. We examined how each patient’s IFN signature score rose or fell over time and clustered patients with similar trajectories (see “Methods”). Using this approach, we identified two distinct types of IFN trajectories (Fig. 4C):

- Trajectory 1: Early IFN upregulation at PostNeo followed by decline during adjuvant cycles.
- Trajectory 2: Delayed IFN activation with persistently high levels throughout adjuvant therapy.

Patients in Trajectory 1 exhibited significantly lower baseline IFN, higher IFN at PostNeo, and lower IFN during adjuvant cycles, whereas Trajectory 2 showed the opposite pattern (higher baseline and adjuvant IFN, but lower IFN at PostNeo). In multivariate analysis, the early, transient IFN-response (Trajectory 1) independently predicted prolonged survival, while delayed, sustained IFN activation (Trajectory 2) was associated with worse outcomes (Fig. 4D, E). These findings suggest that persistent IFN signaling may compromise effective anti-tumor immunity^29,30^.

**Fig. 5:**
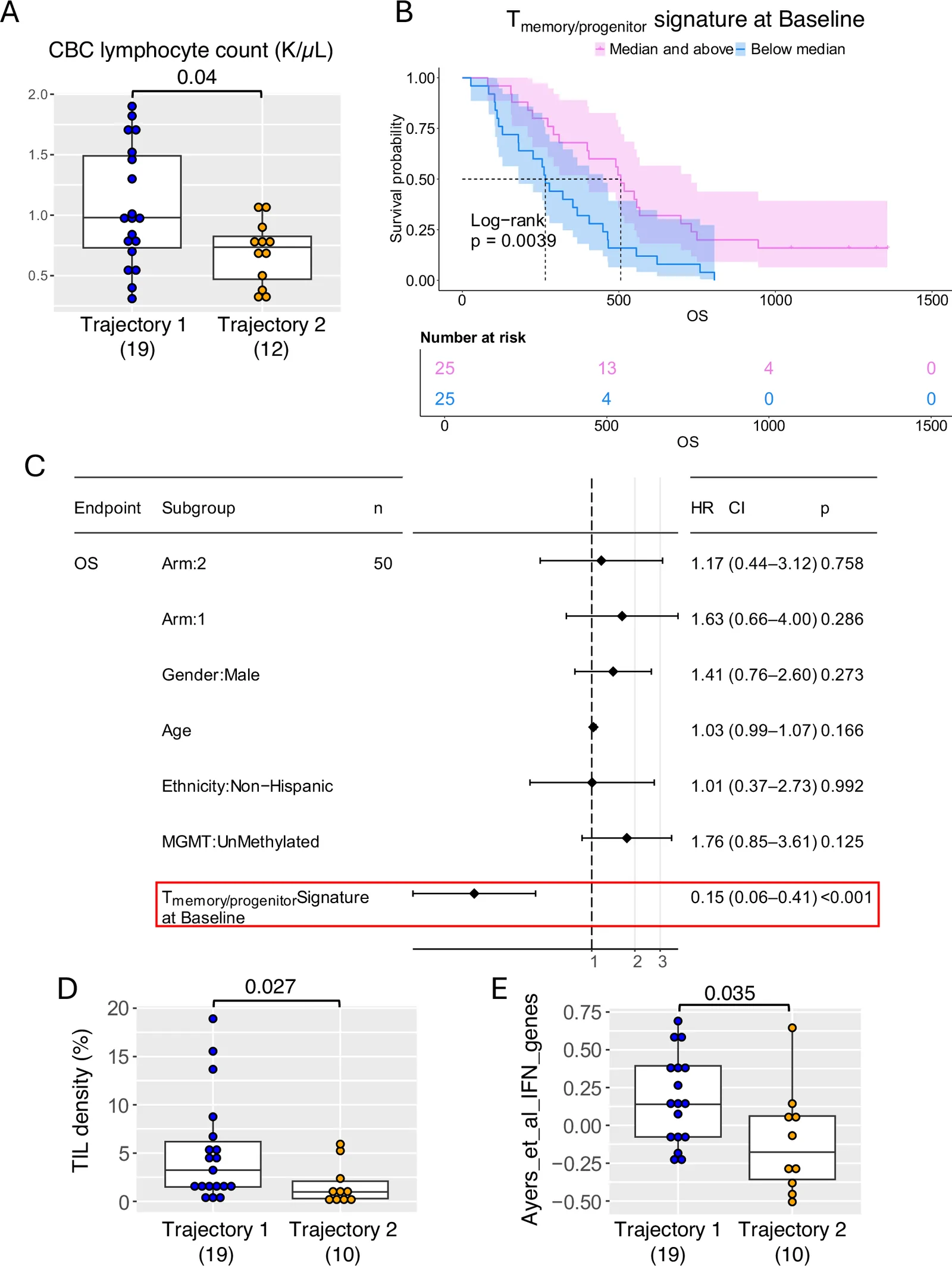
Trajectory 1 exhibits a favorable systemic and intratumoral immune profile. **A** Boxplots comparing CBC lymphocyte counts in PBMCs at Baseline. The values in parentheses indicate the number of patients. Each dot represents a patient. The lower and upper bounds indicate the 25th and 75th percentiles, and the middle lines the median values. *P*-values were calculated using a two-sided Wilcoxon rank-sum test. **B**, **C** Kaplan–Meier curves (**B**) and forest plot of Cox proportional hazards models (**C**) showing the impact of a memory/progenitor-like T-cell signature at Baseline on overall survival. *n* = 50 indicates the number of patients having PBMC RNA-seq data at Baseline. Diamonds at the centers of the error bars indicate HR estimates; error bars indicate 95% confidence intervals (CI); *P*-values are based on two-sided Wald statistics (without adjustment for multiple comparisons). **D**, **E** Boxplots comparing TIL density (**D**) and intratumoral Ayers_et_al_IFN_genes signature scores (**E**) between Trajectory 1 and Trajectory 2. For all boxplots, the values in parentheses indicate the number of patients; dots represent individual patients; boxes indicate the interquartile range (25th–75th percentile); horizontal lines denote median values. *P*-values were calculated using a two-sided Wilcoxon rank-sum test. Source data are provided as a Source data file.

To delineate how early/transient versus delayed/prolonged IFN activation shapes systemic immune composition and signaling, we screened and curated thousands of MSigDB immune signatures, focusing on those differentially expressed between Trajectories 1 and 2 at each time point (Supplementary Data 9). At Baseline, innate immune programs-such as CSF3/G-CSF signaling and Fcγ receptor activation, associated with neutrophils and inflammatory monocytes, were significantly higher in Trajectory 2 than Trajectory 1 (Supplementary Fig. 8A). At PostNeo, type I and II IFN signatures were, as expected, significantly higher in Trajectory 1 than 2 (Supplementary Fig. 8A). By Adjuvant Cycle 1, signatures related to activated monocytes were significantly enriched in Trajectory 2 (Supplementary Fig. 8A). Consistent with these RNA-seq findings, patient and time point matched complete blood count with differential (CBC) analysis revealed higher neutrophil-to-lymphocyte ratio (NLR) and monocyte-to-lymphocyte ratio (MLR) in Trajectory 2 at Baseline and Adjuvant cycles (Supplementary Fig. 8B). Such results have been shown to correlate with poor prognosis across cancer types^31–33^. In contrast, Trajectory 1 exhibited higher baseline lymphocyte levels (Fig. 5A). Unbiased per-gene Cox proportional hazards (Cox-PH) analysis further identified a set of genes linked to memory/progenitor-like T cells—a subset previously shown to be preferentially expanded by anti-CTLA-4 therapy^34^ (Supplementary Data 10). Baseline expression of these genes was higher in Trajectory 1 (Supplementary Fig. 8C) and strongly correlated with improved survival (Fig. 5B, C). Collectively, these data suggest that patients in Trajectory 1 possessed a favorable systemic immune state—characterized by high memory/stem-like T cells and low NLR/MLR—primed for effective anti-tumor T-cell activation. Conversely, patients in Trajectory 2 exhibited chronic inflammation driven by sustained IFN signaling and an increased proportion of suppressive neutrophils and monocytes, which may impair cytotoxic T-cell activation and diminish ICB response^30,35^.

Since higher TIL density is shown to correlate positively with survival (Fig. 3C, D), we next asked whether peripheral IFN trajectories reflect intratumoral immune status. Comparing TIL density and intratumoral IFN signature GSVA scores between patients in Trajectories 1 and 2 revealed significantly higher TIL density and intratumoral IFN scores in Trajectory 1 (Fig. 5D, E). These findings indicate an association between early systemic IFN activation and focal T-cell infiltration within the tumor microenvironment, suggesting that peripheral IFN changes could serve as a useful biomarker for intratumoral immune activation and patient prognosis.

### Dual ICB is associated with extended overall survival

**Fig. 6:**
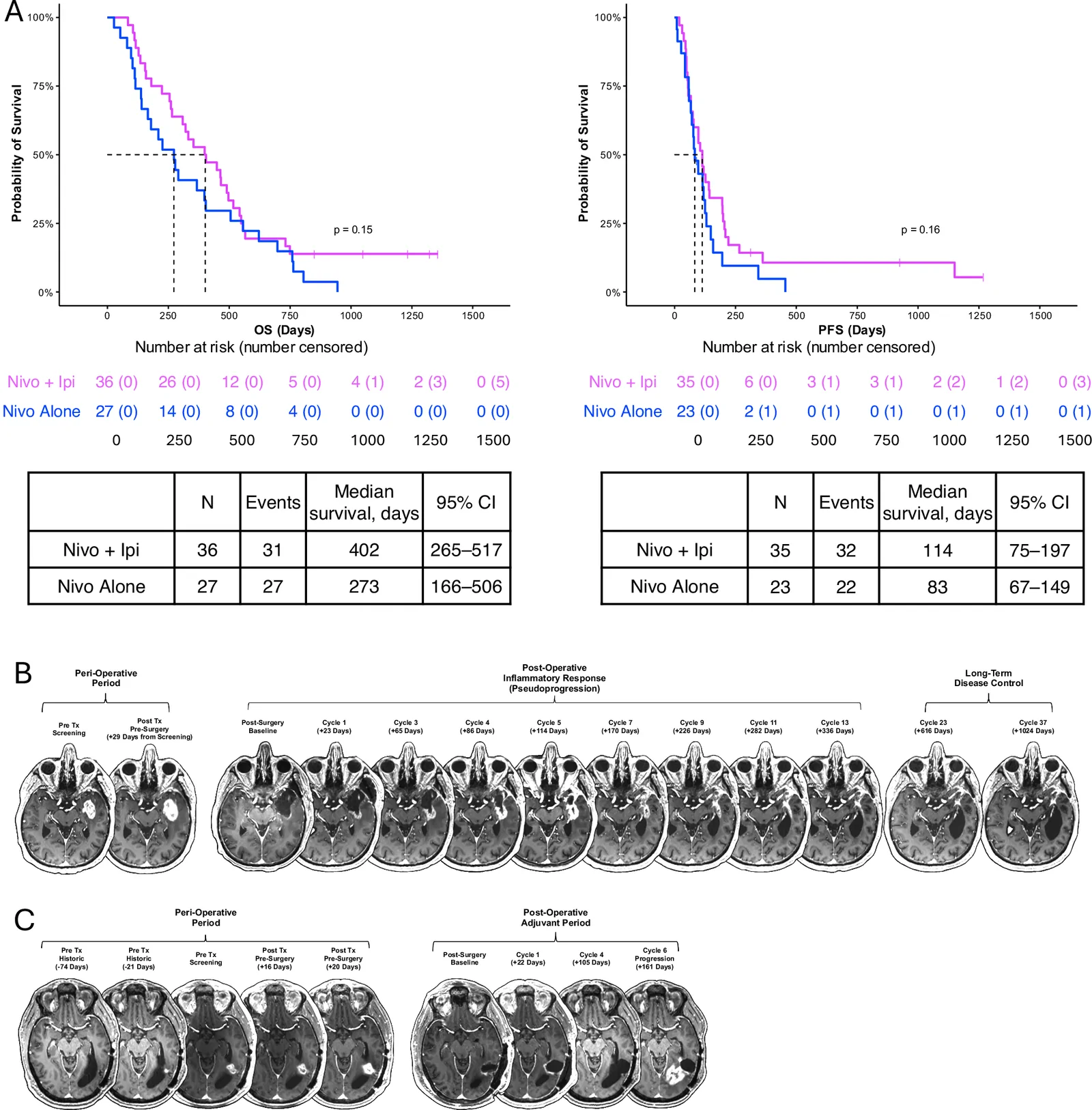
Dual ICB is associated with extended overall survival. **A** Kaplan–Meier survival plot comparing the overall survival and progression-free survival for patients randomized to Arms 1 and 3 (dual ICB) versus Arm 2 (single-agent PD-1 ICB). Log-rank *P* values (without adjustment for multiple comparisons) are shown. **B** A patient with a left temporal recurrent IDH wild type, MGMT promoter methylated glioblastoma with a KPS of 90 and not on any steroids, randomized to Arm 1. During the 29-day peri-operative period, the tumor almost doubled in volume. After gross total resection (post-operative residual enhancing volume of 0.43 mL), the patient experienced an extended period of slow progressive enhancement followed by regression, consistent with an inflammatory response or pseudoprogression. This was followed by long-term disease control extending several years. **C** A patient with a recurrent IDH wild type, MGMT promoter methylated glioblastoma with KPS of 90 and no steroids, randomized to Arm 2. Patient exhibited pre-treatment tumor growth prior to screening, followed by slight expansion of the enhancing lesion during the 20-day peri-operative period. After surgical resection (post-operative residual enhancing volume of 1.35 mL), the patient experienced a period of stability followed by rapid progression by 5 months. The patient later went on to get a repeat surgery of which recurrent disease was confirmed. Source data are provided as a Source data file.

As a secondary endpoint, the median OS was 342 days for Arm 1 (261–543, 95% CL), 273 days for Arm 2 (166–506, 95% CL) and 465 days for Arm 3 (255–NA, 95% CL) (Supplementary Fig. 9A). Twelve-month OS (OS12) was 46% for Arm 1, 40% for Arm 2 and 70% for Arm 3 (Supplementary Fig. 9A). When combining patients who received dual PD-1 + CTLA-4 blockade either before or after surgery (Arms 1 and 3), the median OS was 402 days compared with 273 days for patients treated with single-agent PD-1 blockade (Fig. 6A, Supplementary Fig. 9B). Progression‑free survival (PFS) analysis similarly demonstrated a survival advantage for dual PD‑1 + CTLA‑4 blockade. Median PFS was 105 days for Arm 1 (56–199 days, 95% CL), 170 days for Arm 3 (75–NA days, 95% CL), and 83 days for Arm 2 (67–149 days, 95% CL) (Supplementary Fig. 9C). Six‑month PFS (PFS6) rates were 28% and 50% for Arms 1 and 3, respectively, compared with 13% for Arm 2 (Supplementary Fig. 9C). When Arms 1 and 3 were combined, the median PFS was 114 days (75–197 days, 95% CL), compared with 83 days (67–149 days, 95% CL) for single‑agent PD‑1 blockade (Fig. 6A). The incidence of pseudoprogression likely contributed to the disparity between the PFS times and the OS (Fig. 6B).

Five patients treated with dual ICB achieved long-term survival (OS > 3 years) and remain alive without progression at the data cutoff (Supplementary Data 1), often exhibiting a transient post-operative inflammatory response followed by sustained disease control (Fig. 6B). No patients treated with PD-1 monotherapy experienced long-term survival (Supplementary Data 1), with most patients rapidly progressing with no evidence of long-term disease control (Fig. 6C). Four out of 5 of the long-term survivors had significantly elevated TIL density, corroborating the significant association between the TIL density and survival, but also establishing a potential threshold for responsiveness to dual ICB in this population. Given that this patient population was uniformly IDH-wild-type relapsed GBM, a population with historically poor outcomes, the survival aspects of this dual PD-1 + CTLA-4 blockade compare favorably with other trials in similar settings^19,36^.

## Discussion

This clinical trial was designed to test whether dual ICB could overcome the adaptive immune resistance observed with single-agent PD-1 inhibition in relapsed GBM^18^. Integrated analyses of transcriptional profiles, TCR sequencing, and multiplex immunofluorescence indicate that the trial achieved its primary pharmacodynamic endpoint. Gene expression data reveal a distinct signature of combined PD-1 and CTLA-4 blockade in recurrent glioblastoma, associated with increased tumor-infiltrating T cells and upregulation of T-cell and interferon-related genes; changes more pronounced than with PD-1 ICB monotherapy. Furthermore, neoadjuvant dual ICB elicited significantly stronger systemic interferon activation compared to PD-1 blockade alone. These findings align with the complementary mechanisms of anti-CTLA-4 and anti-PD-1 therapy: CTLA-4 blockade acts early in lymph nodes by removing inhibitory signals on naive and memory T-cell priming, promoting expansion of IFN-γ-producing Th1-like CD4⁺ T cells and progenitor-exhausted CD8⁺ T cells. In contrast, PD-1 blockade primarily reinvigorates terminally exhausted CD8⁺ T cells within tissues, restoring cytotoxic function in the tumor microenvironment^34,37^.

In this study, we also found that treatment-associated tumor-infiltrating lymphocyte (TIL) density was one of the strongest intratumoral pharmacodynamic endpoints, whereas tumor mutational burden was not. Although TIL density correlated with the intratumoral IFN signature score (Rho = 0.54), the IFN score itself was not associated with survival. This suggests that the survival benefit from neoadjuvant ICB is primarily driven by the T cell-mediated response rather than IFN signaling alone. The lack of association may reflect the context-dependent dual role of IFN signaling-either enhancing or impairing ICB efficacy depending on timing and cellular context^38,39^. Furthermore, bulk RNA sequencing limits the cell type-specific resolution of IFN sources and their downstream effects. Future single-cell profiling of this cohort will be essential to delineate the cellular origins and functional consequences of IFN signaling within the tumor microenvironment.

Analysis of temporal IFN signature scores in PBMCs revealed how baseline levels and dynamic changes influence outcomes. Patients with low baseline IFN signaling typically exhibited early induction of IFN-response genes after neoadjuvant therapy, followed by sustained decline. In contrast, patients with high baseline IFN signaling showed delayed activation and persistently elevated IFN levels during adjuvant cycles. The first trajectory was associated with higher baseline lymphocyte counts, lower NLR and MLR across time, increased TIL density, and improved prognosis. This pattern mirrors findings from a phase 2 trial of pembrolizumab (aPD-1) ± JAK inhibitor in treatment-naïve metastatic NSCLC^30^, where responders displayed transient inflammation after PD-1 blockade followed by resolution, while nonresponders exhibited chronic inflammation. These observations underscore that prolonged IFN signaling may shift the balance toward negative immune regulation, impairing effective anti-tumor T-cell activity. Further clinical and pre-clinical studies are needed to define which cell populations and pathways are modulated by acute versus chronic IFN signaling and how systemic IFN dynamics influence intratumoral immunity.

The clinical impact of neoadjuvant versus adjuvant dual ICB (Arm 1 vs. Arm 3) in recurrent glioblastoma remains uncertain in the current setting: although Arm 3 showed a trend toward improved survival compared to Arm 1, statistical significance was limited by small sample size in Arm 3 (*n* = 10). In addition, several patients in Arm 3 had either elevated TIL density or dramatic peripheral blood changes with dual ICB following surgery. Further studies will be essential to determine whether differences in the treatment translate into meaningful and reproducible clinical benefit, and to better understand the underlying biological mechanisms.

When integrated with prior trials of PD-1 blockade alone in comparable patient populations^19,36^, dual ICB appears to confer superior outcomes. And while the clinical outcomes of the recent NRG Oncology clinical trial were not different with dual ICB, this trial did not combine with neoadjuvant treatment or surgical resection and was performed only in the adjuvant setting with newly diagnosed MGMT-unmethylated glioblastoma patients^40^. Our unpublished data also suggests that the TIL density of newly diagnosed glioblastoma tissue is quite low and likely limiting for anti-tumor immunity, unless neoadjuvant strategies to enhance the migration of T cells into the tumor are incorporated^41^.

In summary, our study provides important mechanistic insights into the intratumoral and systemic immune alterations induced by dual ICB (aPD-1 + aCTLA-4) in recurrent GBM. However, the relatively small cohort size limits the robustness of subgroup and survival analyses and precludes definitive clinical conclusions. Therefore, these findings should be considered hypothesis-generating and require validation in larger, adequately powered cohorts. Future trials should be adequately powered for survival endpoints and assess whether the intratumoral and systemic biomarker signatures identified here can predict response to immunotherapy in recurrent glioblastoma.

## Methods

### Ethics statement

This multicenter, randomized, placebo-controlled phase Ib clinical trial (ClinicalTrials.gov identifier NCT04606316) was conducted at the Dana-Farber Cancer Institute, Massachusetts General Hospital, Memorial Sloan Kettering Cancer Center, University of California, Los Angeles, and Stanford University. The study protocol was reviewed and approved by the institutional review board at each participating site and conducted in accordance with the Declaration of Helsinki and Good Clinical Practice guidelines. All participants provided written informed consent before enrollment. Participants were not compensated for study participation.

### Study design and patients

This multicenter, randomized, placebo-controlled phase Ib clinical trial had three parallel treatment arms (Supplementary Fig. 10). The study protocol is available in the Supplementary Information (Supplementary Note). Eligibility criteria for this clinical consisted of the following: patients were ≥18 years of age; had IDH wild-type recurrent glioblastoma; and were candidates for surgical debulking. Other key eligibility criteria included: Karnofsky performance status ≥70; previous first-line therapy with at least radiotherapy; first or second relapse with unequivocal evidence of tumor progression; adequate organ function; no high-dose systemic corticosteroids (defined as >4 mg day^–1^ of dexamethasone or bio-equivalent for at least three consecutive days within two weeks of registration); and absence of previous anti-angiogenic or antivascular endothelial growth factor agents. Sex of the participants was self-reported. This study was not powered to detect differences between males and females. The first participant was enrolled on January 28, 2021, and the last participant was enrolled on November 28, 2023.

Participants were randomized to receive neoadjuvant nivolumab plus ipilimumab (Arm 1), nivolumab plus placebo (Arm 2), or double placebo (Arm 3) prior to surgical resection. Randomization was performed sequentially within 5 days of enrollment, with planned enrollment of 25 participants in Arm 1, 25 participants in Arm 2, and 10 participants in Arm 3. The neoadjuvant treatment administered before surgery was double-blinded. Participants, investigators, study staff, and the sponsor remained unaware of treatment assignments during the neoadjuvant period. Site investigational pharmacists and designated data-monitoring personnel responsible for randomization and safety oversight were unblinded. Postoperative treatment was administered in an open-label manner.

Patients assigned to Group 1 received ipilimumab (3 mg/kg) and nivolumab (240 mg), patients in Group 2 received single-agent nivolumab (240 mg), while patients in Group 3 received a placebo infusion, all by intravenous infusion 14  ±  5 d before scheduled surgical resection. Tumor resection was performed according to institutional standards. After recovery from surgery, patients in Groups 1 and 3 received ipilimumab and nivolumab by intravenous infusion every 3 weeks until either tumor progression or an adverse event requiring study drug discontinuation. Patients in Group 2 continued to receive single-agent nivolumab until tumor progression or unacceptable toxicity requiring study drug discontinuation.

Patients were followed for MRI changes, clinical exams and steroid doses until death or second progression. After second progression, patients were followed every 3 months for vital status until death. Overall survival was defined as time from date of registration to date of confirmed death or date of last follow-up, respectively.

### Sample size

Sixty patients were randomly assigned to Arm 1 (25), Arm 2 (25), or Arm 3 (10) prior to surgery. The two-sample t-test was used to compare different groups (Arm 1 vs Arm 3, Arm 2 vs Arm 3, and Arm 1 vs Arm 2) for the primary endpoint as measured by TIL density. No multiple test adjustment was planned for this early phase clinical trial.

With 25 patients in Arm 1 and 25 patients in Arm 2, there is about 80% power to detect an effect size of 0.71 of TIL density comparing Arm 1 versus Arm 2 at an alpha level of 0.05 (1-sided). With 25 patients in a treatment group (Arm 1 or Arm 2) and 10 patients in Arm 3, there is about 80% power to detect an effect size of 0.95 of TIL density comparing different groups (Arm 1 vs Arm 3, and Arm 2 vs Arm 3) at an alpha level of 0.05 (1-sided).

### Statistical methods

Nominal *p*-values were computed for efficacy analyses as a measure of strength of association between the endpoint and the treatment effect rather than formal tests of hypotheses. Unless otherwise stated, all statistical tests were conducted at the *α* = 0.05 level.

The protocol prespecified a one-sided, two-sample Student’s t-test to evaluate differences in tumor-infiltrating lymphocyte (TIL) density across arms (Arm 1 vs. Arm 3, Arm 2 vs. Arm 3, and Arm 1 vs. Arm 2); however, Welch’s t-test was ultimately used to accommodate unequal variances between groups. Kaplan–Meier (KM) curves and median estimates from the KM curves were provided as appropriate. Median OS was estimated and compared using the KM curves.

### Outcomes

The primary endpoint was tumor-infiltrating lymphocyte (TIL) density in resected tumor tissue. Safety was evaluated in all participants who received at least one dose of study treatment. Adverse events were graded according to the National Cancer Institute Common Terminology Criteria for Adverse Events (CTCAE), version 4.0. Secondary endpoints included multiplex immunohistochemistry-based characterization of the tumor immune microenvironment, evaluation of treatment-associated molecular and transcriptional changes, progression-free survival at 6 months (PFS-6), and overall survival. Prespecified correlative analyses included assessment of immune cell populations, gene expression signatures, and peripheral immune responses following treatment. Missing data were handled using complete-case analyses. No imputation was performed, and participants or specimens with missing values for a given endpoint were excluded from that specific analysis.

### Tumor volume measurement

Anatomic MR images were acquired for all patients in the current study using a 1.5 T or 3 T clinical MR scanner using the consensus recommendations for the international standardized brain tumor imaging protocol (BTIP)^42,43^. Briefly, this protocol included parameter matched pre- and post-contrast 3D T1-weighted inversion recovery gradient recalled echo (IR-GRE or MPRAGE) with a minimum of 1.5 mm isotropic resolution, axial T2-weighted and axial T2-weighted fluid attenuated inversion recovery (FLAIR) images both with minimum slice thickness of 4 mm with no interslice gap, and axial diffusion-weighted imaging (and apparent diffusion coefficient maps) using *b*-values of at least 0 and 1000 s/mm^2^. Post-contrast T1-weighted images were acquired 4–8 min after administration of a low molecular weight gadolinium-based contrast agent (e.g., Magnevist, Gadavist, ProHance, etc.) Volumetric segmentation of enhancing disease was performed using semi-automated methods with the NS-HGlio artificial intelligence device (Neosoma Inc)^44^, followed by neuroradiologist review to finalize regions of interest.

### Response assessment in neuro-oncology (RANO 2.0) criteria

Subjects with measurable (1 cm × 1 cm) contrast-enhancing disease at baseline, prior to surgery, were assessed by MRI under the Response Assessment in Neuro-Oncology (RANO 2.0) criteria^45^. Historic scans were used to confirm progression prior to enrollment using RANO guidelines. Since all patients received surgical resection, most patients did not have measurable enhancing disease after surgery, stable disease was the best response, and patients were monitored for the duration of progression-free survival. Progressive disease (PD) was defined by at least 40% increase in contrast enhancing tumor volume, clear clinical deterioration not attributable to other factors, death, or clear progression of non-measurable disease. To mitigate potential immunotherapy-related pseudoprogression, radiographic progression further required confirmation of progression as defined by subsequent increase in contrast enhancing tumor volume >40% beyond initial progressive enhancement during the first 6 months of treatment during the post-operative phase. Pseudoprogression was explicitly defined as initial progressive enhancement within the first 6 months of treatment followed by disease stability or regression on the confirmation scan^46^. If radiographic progression occurred after 6 months from the start of treatment, no confirmation was required for PD.

### Tumor mutational burden

TMB was calculated as the number of nonsynonymous mutations per megabase (Mb) of genome covered, based on one of three clinically validated next-generation sequencing platforms (FoundationOne, OncoPanel, or MSK-IMPACT), which interrogate approximately 300–500 genes known to be recurrently altered in solid tumors. For the Kaplan–Meier analysis shown in Supplementary Fig. 4E, patients were stratified into high- and low-TMB groups using the cohort median TMB value (4.1 mutations/Mb) as the cutoff. This cutoff is lower than the commonly used threshold of 10 mutations/Mb and is consistent with the typically low TMB observed in glioblastoma.

### RNA and genomic DNA isolation

RNA and genomic DNA were extracted from resected tumor specimens preserved in AllProtect tissue reagent (Qiagen) at the time of surgery. We divided the tumor tissue in half and employed the Qiagen RNeasy Mini Kit (#74104) for RNA isolation and the Qiagen DNeasy Blood and Tissue Kit (#69504) for DNA isolation. Total RNA from PBMCs was extracted using the Qiagen RNeasy Mini Kit.

### Bulk RNA-seq

Bulk RNA-sequencing data were analyzed using a previously established pipeline^17–19^. Briefly, RNA-seq reads were mapped to the GRCh38 human genome reference, after which gene-level expression counts were derived^47^. Enrichment of predefined gene signatures within individual samples was evaluated using Gene Set Variation Analysis^28,48^.

### Analysis of IFN trajectories in PBMCs

To characterize patient-level temporal dynamics of the IFN response, we calculated the GSVA scores for a representative IFN signature (HALLMARK_INTERFERON_GAMMA_RESPONSE)^24^ across four treatment time points (Baseline, PostNeo, and Adjuvant Cycles 1 and 2). For each patient with PBMC RNA-seq data at Baseline, PostNeo, and at least one Adjuvant time points, we then calculated the change (Δ) in HALLMARK_INTERFERON_GAMMA_RESPONSE GSVA scores across consecutive intervals—Baseline to PostNeo, PostNeo to Adjuvant Cycle 1, and Adjuvant Cycle 1 to 2, so each patient is represented by a 3‑dimensional vector describing how their IFN score changed over time. Pairwise Euclidean distances among these vectors were computed to construct a distance matrix, and unsupervised clustering was applied to this matrix to group patients with similar IFN trajectory patterns.

### Tumor-infiltrating lymphocyte density estimation

To assess the tumor-infiltrating lymphocyte density, we employed the immunoSEQ Assay (Adaptive Biotechnologies) using genomic DNA obtained from tumor samples immersed in All Protect (Qiagen). The assay amplifies both the TCRβ CDR3 and selected reference genes, which quantitate the total number of T cells and nucleated cells, respectively. Tumor-infiltrating lymphocyte density is calculated by dividing the total number of T cells by the total number of nucleated cells.

### Multiplex immunofluorescence

Formalin-fixed paraffin-embedded (FFPE) tissue sections were stained using multiplex fluorescence (mIF) to spatially visualize and quantify immune cell populations using the Opal 6-Color Manual IHC Kit and protocol (PerkinElmer). The following antibody panel was used: CD14, CD206, HLA-DR, CD4, CD45, and CD8.

Slides were deparaffinized with xylene and rehydrated through a graded ethanol series. Heat-induced epitope retrieval (HIER) was performed prior to each antibody application using either pH9 antigen retrieval buffer (AR9, PerkinElmer) or pH6 antigen retrieval buffer (AR6, PerkinElmer), depending on the antibody.

The following primary antibodies, clones, dilutions, antigen retrieval conditions, and Opal fluorophores were used: CD14 (EPR3653, 1:3500, Abcam; AR9) with Opal 480 (1:100); CD206 (CL0387, 1:300, Sigma; AR6) with Opal 520 (1:100); HLA-DR (EPR3692, 1:300, Abcam; AR9) with Opal 570 (1:100); CD4 (4B12, 1:50, Dako; AR9) with Opal 620 (1:100); CD45 (2B11 + PD7/26, 1:200, Dako; AR6) with Opal 690 (1:100); and CD8 (C8/144B, 1:200, Dako; AR9), which was detected using TSA-DIG amplification (1:100) followed by Opal Polaris 780 (1:25).

All slides were counterstained with Spectral DAPI (PerkinElmer), mounted with ProLong Diamond Antifade Mountant (Thermo Fisher Scientific), and imaged as whole-slide scans at 20× resolution (0.32 µM/pixel) using a Leica Aperio Versa 200 Slide Scanning Microscope equipped with a 16-bit Andor Zyla 5.5-megapixel fluorescence camera (Translational Pathology Core Laboratory, University of California, Los Angeles). 8-bit images were captured using the equipped filters: DAPI 350/460 excitation/emission (ex/em), green 495/537 ex/em, red 580/625 ex/em, and Cy5 640/690 ex/em. Positive stain quantification and spatial cell analysis were performed using HALO image analysis software (Indica Labs). Positivity thresholds were determined using primary antibody-negative control slides for exclusion of auto-fluorescence and nonspecific background staining.

### Statistics and reproducibility

Differential gene set analysis was conducted on gene sets exhibiting sufficient variability across samples, defined by an IQR ≥ 0.2 on GSVA scores. The statistical significance of differences in GSVA scores between two treatment arms were assessed using the Wilcoxon rank-sum test. Associations between RNA-seq profiles (e.g., gene expression, gene set enrichment scores, PBMC IFN trajectories) and TIL density with overall survival were evaluated using the log-rank test, with Kaplan–Meier curves generated via the survminer package in R. Multivariable Cox proportional hazards (Cox-PH) regression was used to estimate hazard ratios (HRs) with 95% confidence intervals (CIs), adjusting for clinical covariates including treatment arm, age, ethnicity, gender, and MGMT status. Three treatment arms were defined:

Arm 1: Neoadjuvant + adjuvant dual ICB.

Arm 2: Neoadjuvant + adjuvant single anti-PD-1 ICB.

Arm 3 (the reference group for Cox-PH analysis): Presurgical placebo followed by adjuvant dual ICB.

### Reporting summary

Further information on research design is available in the Nature Portfolio Reporting Summary linked to this article.

## Supplementary information

Supplementary Information

Description of Additional Supplementary Files

Supplementary Data 1

Supplementary Data 2

Supplementary Data 3

Supplementary Data 4

Supplementary Data 5

Supplementary Data 6

Supplementary Data 7

Supplementary Data 8

Supplementary Data 9

Supplementary Data 10

Reporting Summary

Transparent Peer Review file

## Source data

Source Data

## Data Availability

The study design and de-identified patient information used for the analysis in the manuscript are provided as “Methods” and Supplementary Data 1. The study protocol is available as Supplementary Note in the Supplementary Information. The RNAseq data generated in this study have been deposited in the Gene Expression Omnibus repository under accession code GSE318645 and are publicly available with no restrictions. The Bulk TCR-seq data (including CDR3 sequences, frequencies, and counts) can be accessed through the Adaptive Biotech immuneACCESS database at https://clients.adaptivebiotech.com/pub/sun_2026_ci. Processed bulk TCR-seq data have also been deposited in GEO and are accessible under accession number GSE344555. The remaining data are available within the Article and Supplementary Information file. Source data are provided with this paper.

## Code availability

All data figures are generated using publicly available R packages, as stated in the “Methods” section. The codes for PBMC RNAseq analysis and tumor TCRseq analysis are available at: https://github.com/slulu10/GBM_BulkOmics.

## References

[1] Wen, P. Y. et al. Glioblastoma in Adults: A Society for Neuro-Oncology (SNO) and European Society of Neuro-Oncology (EANO) consensus review on current management and future directions. *Neuro. Oncol.* **27**, 2751–2788, 10.1093/neuonc/noaf177 (2025). PMC12908567 40827022

[2] Larkin, J. et al. Combined nivolumab and ipilimumab or monotherapy in untreated melanoma. *N. Engl. J. Med.* **373**, 23–34, 10.1056/NEJMoa1504030 (2015). PMC5698905 26027431

[3] Larkin, J. et al. Five-year survival with combined nivolumab and ipilimumab in advanced melanoma. *N. Engl. J. Med.* **381**, 1535–1546, 10.1056/NEJMoa1910836 (2019). 31562797

[4] Long, G. V. et al. Combination nivolumab and ipilimumab or nivolumab alone in melanoma brain metastases: a multicentre randomised phase 2 study. *Lancet Oncol.* **19**, 672–681, 10.1016/s1470-2045(18)30139-6 (2018). 29602646

[5] Long, G. V. et al. Ipilimumab plus nivolumab versus nivolumab alone in patients with melanoma brain metastases (ABC): 7-year follow-up of a multicentre, open-label, randomised, phase 2 study. *Lancet Oncol.* **26**, 320–330, 10.1016/S1470-2045(24)00735-6 (2025). 39978375

[6] Long, G. V. et al. Overall survival and response with nivolumab and relatlimab in advanced melanoma. *NEJM Evid.* **2**, EVIDoa2200239, 10.1056/EVIDoa2200239 (2023). 38320023

[7] Blumenthal, D. T. et al. Pembrolizumab: first experience with recurrent primary central nervous system (CNS) tumors. *J. Neurooncol.* **129**, 453–460, 10.1007/s11060-016-2190-1 (2016). 27377654

[8] Bouffet, E. et al. Immune checkpoint inhibition for hypermutant glioblastoma multiforme resulting from germline biallelic mismatch repair Deficiency. *J. Clin. Oncol.* **34**, 2206–2211, 10.1200/JCO.2016.66.6552 (2016). 27001570

[9] Johanns, T. M. et al. Immunogenomics of hypermutated glioblastoma: a patient with germline POLE deficiency treated with checkpoint blockade immunotherapy. *Cancer Discov.* **6**, 1230–1236, 10.1158/2159-8290.CD-16-0575 (2016). PMC5140283 27683556

[10] Reardon, D. A. et al. Effect of nivolumab vs bevacizumab in patients with recurrent glioblastoma: the Checkmate 143 phase 3 randomized clinical trial. *JAMA Oncol.* 10.1001/jamaoncol.2020.1024 (2020). PMC7243167 32437507

[11] Antonios, J. P. et al. Immunosuppressive tumor-infiltrating myeloid cells mediate adaptive immune resistance via a PD-1/PD-L1 mechanism in glioblastoma. *Neuro Oncol.* **19**, 796–807, 10.1093/neuonc/now287 (2017). PMC5464463 28115578

[12] Antonios, J. P. et al. PD-1 blockade enhances the vaccination-induced immune response in glioma. *JCI Insight* **1**, e87059. 10.1172/jci.insight.87059 (2016). PMC4951098 27453950

[13] Bloch, O. et al. Gliomas promote immunosuppression through induction of B7-H1 expression in tumor-associated macrophages. *Clin. Cancer Res.* **19**, 3165–3175, 10.1158/1078-0432.CCR-12-3314 (2013). PMC3742575 23613317

[14] Reardon, D. A. et al. Glioblastoma eradication following immune checkpoint blockade in an orthotopic, immunocompetent model. *Cancer Immunol. Res.* **4**, 124–135, 10.1158/2326-6066.CIR-15-0151 (2016). 26546453

[15] Wainwright, D. A. et al. Durable therapeutic efficacy utilizing combinatorial blockade against IDO, CTLA-4, and PD-L1 in mice with brain tumors. *Clin. Cancer Res.* **20**, 5290–5301, 10.1158/1078-0432.CCR-14-0514 (2014). PMC4182350 24691018

[16] Zeng, J. et al. Anti-PD-1 blockade and stereotactic radiation produce long-term survival in mice with intracranial gliomas. *Int J. Radiat. Oncol. Biol. Phys.* **86**, 343–349, 10.1016/j.ijrobp.2012.12.025 (2013). PMC3963403 23462419

[17] Cloughesy, T. F. et al. Neoadjuvant anti-PD-1 immunotherapy promotes a survival benefit with intratumoral and systemic immune responses in recurrent glioblastoma. *Nat. Med.* **25**, 477–486, 10.1038/s41591-018-0337-7 (2019). PMC6408961 30742122

[18] Lee, A. H. et al. Neoadjuvant PD-1 blockade induces T cell and cDC1 activation but fails to overcome the immunosuppressive tumor associated macrophages in recurrent glioblastoma. *Nat. Commun.* **12**, 6938. 10.1038/s41467-021-26940-2 (2021). PMC8626557 34836966

[19] McFaline-Figueroa, J. R. et al. Neoadjuvant anti-PD1 immunotherapy for surgically accessible recurrent glioblastoma: clinical and molecular outcomes of a stage 2 single-arm expansion cohort. *Nat. Commun.* **15**, 10757. 10.1038/s41467-024-54326-7 (2024). PMC11685579 39737895

[20] Snyder, A. et al. Genetic basis for clinical response to CTLA-4 blockade in melanoma. *N. Engl. J. Med.* **371**, 2189–2199, 10.1056/NEJMoa1406498 (2014). PMC4315319 25409260

[21] Ghannam, J. Y. et al. Tumor transcriptional state predicts survival in immune-checkpoint-blockade-treated glioblastoma. *Nat Cancer* **7**, 964–982, 10.1038/s43018-026-01179-2 (2026). 42237038

[22] Gromeier, M. et al. Very low mutation burden is a feature of inflamed recurrent glioblastomas responsive to cancer immunotherapy. *Nat. Commun.* **12**, 352. 10.1038/s41467-020-20469-6 (2021). PMC7806846 33441554

[23] Touat, M. et al. Mechanisms and therapeutic implications of hypermutation in gliomas. *Nature* **580**, 517–523, 10.1038/s41586-020-2209-9 (2020). PMC8235024 32322066

[24] Ellingson, B. M. et al. Validation of postoperative residual contrast-enhancing tumor volume as an independent prognostic factor for overall survival in newly diagnosed glioblastoma. *Neuro Oncol.* **20**, 1240–1250, 10.1093/neuonc/noy053 (2018). PMC6071654 29660006

[25] Chiffelle, J. et al. T-cell repertoire analysis and metrics of diversity and clonality. *Curr. Opin. Biotechnol.* **65**, 284–295, 10.1016/j.copbio.2020.07.010 (2020). 32889231

[26] Porciello, N., Franzese, O., D’Ambrosio, L., Palermo, B. & Nisticò, P. T-cell repertoire diversity: friend or foe for protective antitumor response? *J. Exp. Clin. Cancer Res.* **41**, 356, 10.1186/s13046-022-02566-0 (2022). PMC9773533 36550555

[27] Ayers, M. et al. IFN-gamma-related mRNA profile predicts clinical response to PD-1 blockade. *J. Clin. Invest* **127**, 2930–2940, 10.1172/JCI91190 (2017). PMC5531419 28650338

[28] Liberzon, A. et al. The Molecular Signatures Database (MSigDB) hallmark gene set collection. *Cell Syst.* **1**, 417–425, 10.1016/j.cels.2015.12.004 (2015). PMC4707969 26771021

[29] Boukhaled, G. M. et al. Pre-encoded responsiveness to type I interferon in the peripheral immune system defines outcome of PD1 blockade therapy. *Nat. Immunol.* **23**, 1273–1283, 10.1038/s41590-022-01262-7 (2022). 35835962

[30] Mathew, D. et al. Combined JAK inhibition and PD-1 immunotherapy for non-small cell lung cancer patients. *Science* **384**, eadf1329. 10.1126/science.adf1329 (2024). PMC11327955 38900877

[31] Choi, Y. H. et al. A high monocyte-to-lymphocyte ratio predicts poor prognosis in patients with advanced gallbladder cancer receiving chemotherapy. *Cancer Epidemiol. Biomark. Prev.* **28**, 1045–1051, 10.1158/1055-9965.EPI-18-1066 (2019). 30842131

[32] Howard, R., Kanetsky, P. A. & Egan, K. M. Exploring the prognostic value of the neutrophil-to-lymphocyte ratio in cancer. *Sci. Rep.* **9**, 19673. 10.1038/s41598-019-56218-z (2019). PMC6928022 31873162

[33] Obeagu, E. I. Monocyte-to-lymphocyte ratio as a predictive marker for breast cancer treatment outcomes: a narrative review. *Ann. Med. Surg.* **87**, 7306–7310, 10.1097/ms9.0000000000003871 (2025). PMC12577881 41180720

[34] Wang, K. et al. Combination anti-PD-1 and anti-CTLA-4 therapy generates waves of clonal responses that include progenitor-exhausted CD8(+) T cells. *Cancer Cell* **42**, 1582–1597.e1510, 10.1016/j.ccell.2024.08.007 (2024). PMC11387127 39214097

[35] Qiu, J. et al. Cancer cells resistant to immune checkpoint blockade acquire interferon-associated epigenetic memory to sustain T cell dysfunction. *Nat. Cancer* **4**, 43–61, 10.1038/s43018-022-00490-y (2023). 36646856

[36] Schalper, K. A. et al. Neoadjuvant nivolumab modifies the tumor immune microenvironment in resectable glioblastoma. *Nat. Med.* **25**, 470–476, 10.1038/s41591-018-0339-5 (2019). 30742120

[37] Wei, S. C. et al. Distinct cellular mechanisms underlie anti-CTLA-4 and anti-PD-1 checkpoint blockade. *Cell* **170**, 1120–1133 e1117, 10.1016/j.cell.2017.07.024 (2017). PMC5591072 28803728

[38] Benci, J. L. et al. Tumor interferon signaling regulates a multigenic resistance program to immune checkpoint blockade. *Cell* **167**, 1540–1554.e1512, 10.1016/j.cell.2016.11.022 (2016). PMC5385895 27912061

[39] Purbey, P. K. et al. Opposing tumor-cell-intrinsic and -extrinsic roles of the IRF1 transcription factor in antitumor immunity. *Cell Rep.* **43**, 114289. 10.1016/j.celrep.2024.114289 (2024). PMC11315447 38833371

[40] Lassman, A. B. et al. Dual immune check point blockade in MGMT-unmethylated newly diagnosed glioblastoma: NRG Oncology BN007, a randomized phase II/III clinical trial. *J. Clin. Oncol.* **43**, 3032–3040, 10.1200/JCO-25-00618 (2025). PMC12440284 40779733

[41] Long, G. V. et al. Neoadjuvant triplet immune checkpoint blockade in newly diagnosed glioblastoma. *Nat. Med.* **31**, 1557–1566, 10.1038/s41591-025-03512-1 (2025). PMC12092302 40016450

[42] Ellingson, B. M. et al. Consensus recommendations for a standardized Brain Tumor Imaging Protocol in clinical trials. *Neuro Oncol.* **17**, 1188–1198, 10.1093/neuonc/nov095 (2015). PMC4588759 26250565

[43] Sanvito, F., Kaufmann, T. J., Cloughesy, T. F., Wen, P. Y. & Ellingson, B. M. Standardized brain tumor imaging protocols for clinical trials: current recommendations and tips for integration. *Front Radio.* **3**, 1267615. 10.3389/fradi.2023.1267615 (2023). PMC10751345 38152383

[44] Abayazeed, A. H. et al. NS-HGlio: a generalizable and repeatable HGG segmentation and volumetric measurement AI algorithm for the longitudinal MRI assessment to inform RANO in trials and clinics. *Neurooncol Adv.* **5**, vdac184. 10.1093/noajnl/vdac184 (2023). PMC9850874 36685009

[45] Wen, P. Y. et al. RANO 2.0: update to the response assessment in neuro-oncology criteria for high- and low-grade gliomas in adults. *J. Clin. Oncol.* **41**, 5187–5199, 10.1200/JCO.23.01059 (2023). PMC10860967 37774317

[46] Ellingson, B. M., Wen, P. Y. & Cloughesy, T. F. Modified criteria for radiographic response assessment in glioblastoma clinical trials. *Neurotherapeutics* **14**, 307–320, 10.1007/s13311-016-0507-6 (2017). PMC5398984 28108885

[47] Anders, S., Pyl, P. T. & Huber, W. HTSeq—a Python framework to work with high-throughput sequencing data. *Bioinformatics* **31**, 166–169, 10.1093/bioinformatics/btu638 (2015). PMC4287950 25260700

[48] Subramanian, A. et al. Gene set enrichment analysis: a knowledge-based approach for interpreting genome-wide expression profiles. *Proc. Natl. Acad. Sci. USA* **102**, 15545–15550, 10.1073/pnas.0506580102 (2005). PMC1239896 16199517

