# Supplementary material for: Neoadjuvant PD1 plus CTLA-4 immune checkpoint blockade in surgically resectable recurrent glioblastoma: a randomized surgical window-of-opportunity trial
Source: Nat Commun. 2026 Sep 5;17:10560. doi: 10.1038/s41467-026-77433-z (PMC13642311; doi:10.1038/s41467-026-77433-z)
Supplement: Supplementary file 1 [file 41467_2026_77433_MOESM13_ESM.pdf]

nature portfolio

Corresponding author(s): Patrick Wen, Robert Prins, Tim Cloughesy

Last updated by author(s): Jul 20, 2026

Reporting Summary

Nature Portfolio wishes to improve the reproducibility of the work that we publish. This form provides structure for consistency and transparency in reporting. For further information on Nature Portfolio policies, see our Editorial Policies and the Editorial Policy Checklist.

Statistics

For all statistical analyses, confirm that the following items are present in the figure legend, table legend, main text, or Methods section.

n/a	Confirmed	
☐	☒	The exact sample size (*n*) for each experimental group/condition, given as a discrete number and unit of measurement
☐	☒	A statement on whether measurements were taken from distinct samples or whether the same sample was measured repeatedly
☐	☒	The statistical test(s) used AND whether they are one- or two-sided<br>*Only common tests should be described solely by name; describe more complex techniques in the Methods section.*
☐	☒	A description of all covariates tested
☐	☒	A description of any assumptions or corrections, such as tests of normality and adjustment for multiple comparisons
☐	☒	A full description of the statistical parameters including central tendency (e.g. means) or other basic estimates (e.g. regression coefficient) AND variation (e.g. standard deviation) or associated estimates of uncertainty (e.g. confidence intervals)
☐	☒	For null hypothesis testing, the test statistic (e.g. *F*, *t*, *r*) with confidence intervals, effect sizes, degrees of freedom and *P* value noted<br>*Give P values as exact values whenever suitable.*
☒	☐	For Bayesian analysis, information on the choice of priors and Markov chain Monte Carlo settings
☒	☐	For hierarchical and complex designs, identification of the appropriate level for tests and full reporting of outcomes
☒	☐	Estimates of effect sizes (e.g. Cohen's *d*, Pearson's *r*), indicating how they were calculated

Our web collection on statistics for biologists contains articles on many of the points above.

Software and code

Policy information about availability of computer code

Data collection	Data was collected with Microsoft Excel or R on a secure server.
Data analysis	Statistical analysis was performed using GraphPad Prism v. 10.1.1, Insight Toolkit program (ITK, 2021, version 5.3.0; https://itk.org/) , 3D Slicer program (3D Slicer, 2019, version 4.10.2; https://www.slicer.org/), Robust Brain Extraction program (ROBEX, 2013, version 1.2; https://www.nitrc.org/projects/robex/), ITK-SNAP (ITK-SNAP, version 3.8.0; http://www.itksnap.org/pmwiki/pmwiki.php).<br><br>For bulk RNAseq, paired-end, 2 × 100 base pair (bp) transcriptome reads were mapped to the Genome Reference Consortium Human Build 38 (GRCh38) reference genome using HISAT2 (version 2.0.6). The gene level counts were generated by the HTSeq-count program (version 0.5.4p5). To calculate single sample gene set enrichment, we used the Gene Set Variation Analysis (GSVA) package (version 1.32.0). To compute the GSVA scores, the filtered, log2 normalized gene expression were supplied to the GSVA program using the 'kcdf=Gaussian' mode. We manually selected gene sets from the c2.cgp, c6, c7, hallmark geneset collections of the Broad Institute's Molecular Signatures Database (version 7.0) and the study by Ayers et al. (PMID: 28650338).<br><br>To assess the tumor-infiltrating lymphocyte density, we employed the immunoSEQ Assay (Adaptive Biotechnologies) using genomic DNA obtained from tumor samples immersed in All Protect (Qiagen). The assay amplifies both the TCRβ CDR3 and selected reference genes, which quantitate the total number of T cells and nucleated cells, respectively. Tumor-infiltrating lymphocyte density is calculated by dividing the total number of T cells by the total number of nucleated cells.<br><br>Cox proportional hazards (Coxph) analysis was conducted, and Kaplan-Meier plots were created utilizing the survival (version 3.4.0) and survminer (version 0.4.9) packages in R. Forest plots were produced with the survivalAnalysis package (version 0.3.0), and boxplots were produced using the ggplot2 package (version 3.4.0).

We did not generate/utilize any custom software/algorithms generated for the analysis.

For manuscripts utilizing custom algorithms or software that are central to the research but not yet described in published literature, software must be made available to editors and reviewers. We strongly encourage code deposition in a community repository (e.g. GitHub). See the Nature Portfolio guidelines for submitting code & software for further information.

Data

Policy information about availability of data

All manuscripts must include a data availability statement. This statement should provide the following information, where applicable:

- Accession codes, unique identifiers, or web links for publicly available datasets
- A description of any restrictions on data availability
- For clinical datasets or third party data, please ensure that the statement adheres to our policy

The study design and de-identified patient information used for the analysis in the manuscript are provided as Methods and Supplementary Data 1. The RNAseq data generated in this study have been deposited in the Gene Expression Omnibus repository under accession code GSE318645 and are publicly available with no restrictions. The processed TCR-seq data (including CDR3 sequences, frequencies, and counts) used to generate Supplementary Figures 4J-M have been deposited in the Zenodo repository and are publicly available with no restrictions (DOI: https://doi.org/10.5281/zenodo.21725791). The remaining data are available within the Article, and Supplementary Information file. Source data are provided with this paper.

Research involving human participants, their data, or biological material

Policy information about studies with human participants or human data. See also policy information about sex, gender (identity/presentation), and sexual orientation and race, ethnicity and racism.

Reporting on sex and gender	Self-reported sex was collected at enrollment and recorded as male or female. Among the 63 randomized patients, 33 were male and 30 were female. This study was not powered to detect differences between males and females.
Reporting on race, ethnicity, or other socially relevant groupings	Self-assigned race/ethnicity data was collected from study participants at enrollment. Race categories included American Indian or Alaska Native, Black or African American, White or Other. Ethnicity categories included Hispanic or Latino, Not Hispanic or Unknown.
Population characteristics	Disease relevant clinical characteristics of the patient population are included in Supplementary data file 1
Recruitment	Participants were recruited by site-specific co-investigators at their respective institutions based on protocol eligibility criteria and verified by the study coordinator. Self-selection bias may be present and may affect survival.
Ethics oversight	This study was approved by institutional review boards (IRB) at all sites (Dana-Farber Cancer Institute; Massachusetts General Hospital; Memorial Sloan Kettering Cancer Center; University of California, Los Angeles; Stanford University) and was conducted according to the Declaration of Helsinki. All patients provided written informed consent. Participants were not compensated for study participation.

Note that full information on the approval of the study protocol must also be provided in the manuscript.

Field-specific reporting

Please select the one below that is the best fit for your research. If you are not sure, read the appropriate sections before making your selection.

☒ Life sciences ☐ Behavioural & social sciences ☐ Ecological, evolutionary & environmental sciences

For a reference copy of the document with all sections, see nature.com/documents/nr-reporting-summary-flat.pdf

Life sciences study design

All studies must disclose on these points even when the disclosure is negative.

Sample size	In the initial design, 63 patients will be randomly assigned to Arm 1 (26), Arm 2 (27), or Arm 3 (10) prior to surgery. In the actual study, a total of 58 patients received study therapy: Arm 1 (n=25), Arm 2 (n=23), Arm 3 (n=10). The two-sample t-test will be used to compare different groups (Arm 1 vs Arm 3, Arm 2 vs Arm 3, and Arm 1 vs Arm 2) for the primary endpoint as measured by TIL density. No multiple test adjustment is planned for this early phase clinical trial.<br>With 25 patients in Arm 1 and 23 patients in Arm 2, there is about 80% power to detect an effect size of 0.73 of TIL density comparing Arm 1 versus Arm 2 at an alpha level of 0.05 (1-sided). With 25 patients in a Arm 1 and 10 patients in Arm 3, there is about 80% power to detect an effect size of 0.95 of TIL density comparing Arm 1 versus Arm 3, at an alpha level of 0.05 (1-sided).
Data exclusions	Thirteen of these patients did not initiate study therapy including 1 patient who did not meet eligibility criteria (IDH status) and five patients who elected to withdraw consent after randomization but prior to initiating study therapy.
Replication	Data collected on individual patient samples could not be replicated due to the limited quantity of available specimens.
Randomization	Patients were randomized at enrollment into 3 groups (placebo, single agent nivolumab, and ipilimumab + nivolumab).

Blinding	Investigators were not blinded to treatment groups.

Reporting for specific materials, systems and methods

We require information from authors about some types of materials, experimental systems and methods used in many studies. Here, indicate whether each material, system or method listed is relevant to your study. If you are not sure if a list item applies to your research, read the appropriate section before selecting a response.

Materials & experimental systems

n/a	Involved in the study	
☐	☒	Antibodies
☒	☐	Eukaryotic cell lines
☒	☐	Palaeontology and archaeology
☒	☐	Animals and other organisms
☐	☒	Clinical data
☒	☐	Dual use research of concern
☒	☐	Plants

Methods

n/a	Involved in the study	
☒	☐	ChIP-seq
☒	☐	Flow cytometry
☐	☒	MRI-based neuroimaging

Antibodies

Antibodies used	The following primary antibodies, clones, dilutions, antigen retrieval conditions, and Opal fluorophores were used: CD14 (EPR3653, 1:3500, Abcam; AR9) with Opal 480 (1:100); CD206 (CL0387, 1:300, Sigma; AR6) with Opal 520 (1:100); HLA-DR (EPR3692, 1:300, Abcam; AR9) with Opal 570 (1:100); CD4 (4B12, 1:50, Dako; AR9) with Opal 620 (1:100); CD45 (2B11+PD7/26, 1:200, Dako; AR6) with Opal 690 (1:100); and CD8 (C8/144B, 1:200, Dako; AR9), which was detected using TSA-DIG amplification (1:100) followed by Opal Polaris 780 (1:25).
Validation	Please refer to the Result and Method sections in the manuscript.

Clinical data

Policy information about clinical studies

All manuscripts should comply with the ICMJE guidelines for publication of clinical research and a completed CONSORT checklist must be included with all submissions.

Clinical trial registration	NCT04606316
Study protocol	The study protocol has been submitted as supplementary material.
Data collection	Data was collected by study personnel at participating institutions between January of 2021 and 2024 . Overall survival was defined as time from date of registration to date of confirmed death or date of last follow-up, respectively.
Outcomes	This clinical trial was designed to test whether dual ICB could overcome the adaptive immune resistance observed with single-agent PD-1 inhibition in relapsed GBM, as measured by TIL density. Integrated analyses of transcriptional profiles, TCR sequencing, and multiplex immunofluorescence indicate that the trial achieved its primary pharmacodynamic endpoint.

Magnetic resonance imaging

Experimental design

Design type	Anatomic MR images were acquired for all patients in the current study for tumor volume measurement
Design specifications	The current study involved acquisition of anatomic MRI at screening, prior to surgery, after surgery, and every treatment cycle until tumor progression.
Behavioral performance measures	N/A

Acquisition

Imaging type(s)	Structural/Anatomic
Field strength	1.5T/3T
Sequence & imaging parameters	All sequences and imaging parameters adhered to the standardized brain tumor imaging protocol (BTIP) consensus recommendations. This included parameter-matched pre- and post-contrast T1-weighted images (MPRAGE or IR-SPGR) with FOV = 256mm, matrix size = 256x256, < 1.5mm isotropic resolution with no interslice gap, minimal TE, TR > 2sec (MPRAGE) or 5-15ms (IR-SPGR), TI = 400ms (IR-SPGR) or 1100ms (MPRAGE), and flip angle = 10-15 degrees. Additionally, axial T2-weighted turbo spin echo images were acquired with FOV = 240-256mm, matrix size ≤256x256,

slice thickness ≤4mm with no interslice gap, TE = 80-120ms, TR > 2500ms, flip angle = ≥80/160 degrees, and axial T2-weighted fluid attenuated inversion recovery (FLAIR) images were acquired with FOV = 240-256mm, matrix size ≤256x256, slice thickness ≤4mm with no interslice gap, TE = 80-120ms, TR > 2500ms, TI = 2000-2500ms, flip angle = ≥80/160 degrees. Axial diffusion-weighted images (DWI) (3 directions) were also acquired with FOV = 240mm, matrix size = 128x128, slice thickness ≤4mm with no interslice gap, minimal TE, TR > 5000ms, and b-values of 0 and 1000 s/mm^2.

Area of acquisition: Whole brain

Diffusion MRI: ☐ Used ☒ Not used

Preprocessing

Preprocessing software: Volumetric segmentation of enhancing disease was performed using semi-automated methods with the NS-HGlio artificial intelligence device (Neosoma Inc) followed by neuroradiologist review to finalize regions of interest.

Normalization: N/A

Normalization template: Images were not normalized/registered to a standard template space. All images were registered to the patient-specific screening MRI exam as mentioned above.

Noise and artifact removal: No noise or artifact removal was performed.

Volume censoring: N/A

Statistical modeling & inference

Model type and settings: N/A

Effect(s) tested: N/A

Specify type of analysis: ☐ Whole brain ☒ ROI-based ☐ Both

Anatomical location(s): Areas of contrast enhancing tumor burden

Statistic type for inference: N/A

(See Eklund et al. 2016)

Correction: N/A

Models & analysis

n/a	Involved in the study
☒	☐ Functional and/or effective connectivity
☒	☐ Graph analysis
☒	☐ Multivariate modeling or predictive analysis
