# Supplementary material for: Neoadjuvant PD1 plus CTLA-4 immune checkpoint blockade in surgically resectable recurrent glioblastoma: a randomized surgical window-of-opportunity trial
Source: Nat Commun. 2026 Sep 5;17:10560. doi: 10.1038/s41467-026-77433-z (PMC13642311; doi:10.1038/s41467-026-77433-z)
Supplement: Supplementary file 2 [file 41467_2026_77433_MOESM14_ESM.pdf]

nature portfolio

Peer Review File

# Neoadjuvant PD1 plus CTLA-4 immune checkpoint blockade in surgically resectable recurrent glioblastoma: a randomized surgical window-of-opportunity trial

Corresponding Author: Dr Robert Prins

Parts of this Peer Review File have been redacted as indicated to maintain the confidentiality of unpublished data.

**This file contains all reviewer reports in order by version, followed by all author rebuttals in order by version.**

Version 1:

Reviewer comments:

Reviewer #1

(Remarks to the Author)
The authors conducted a phase II trial (NCT04606316) evaluating neoadjuvant dual PD-1 + CTLA-4 blockade versus PD-1 monotherapy or placebo in 63 patients with relapsed, resectable glioblastoma. The results reveal enhanced intratumoral and systemic immune activation with dual ICB. Patients receiving dual ICB showed higher TIL density, increased expression of interferon-related response genes, and improved overall survival compared with other arms. This study is interesting and timely. It is well written and presents important correlations. Combining Arms 1 and 3 results in a significant OS difference compared to PD-1 blockade alone (Arm 2). This work will be of great interest to the field. I have a few comments and questions:

1. Arm 3 patients received the same adjuvant dual therapy as Aim 1, but seemed to fare better, despite a smaller sample. With two long-term survivors each from Arm 3 (n=10) and Arm 1 (n=25), does this imply post-surgery adjuvant therapy (Arm 3) could be more effective than Arm 1? Just a thought looking at the data, I recognize sample size is small to make any conclusions.
2. The sentence in Lines 104–106 is unclear, making it difficult to follow the subsequent data. Were all patients in Arm 3 treated with dual ICB post-surgery? The mention of “n=3” is confusing and needs clarification.
3. In Figure 3B, the second-row images are neither quantified nor discussed in the text. The staining includes myeloid markers (CD14, CD206, HLA-DR). Since these data are available, it would strengthen the manuscript to quantify and characterize the myeloid profile from the multiplex IF staining, add corresponding graphs next to the images, and discuss the findings.
4. Please provide higher-resolution inserts for Figure 3B. It would also be beneficial to align these images with the quantification in Figure 3A, as the visual differences appear striking while the quantitative analysis shows no significant change.
5. The method used to define high and low tumor mutational burden (TMB) is unclear. Please specify how these groups were determined, as this information is missing from the Methods section.

Reviewer #2

(Remarks to the Author)
This manuscript presents a well-conducted and clinically relevant window-of-opportunity study investigating dual versus single immune checkpoint inhibition in recurrent glioblastoma. The integration of neoadjuvant and adjuvant treatment with comprehensive immune profiling provides valuable insights into treatment-associated immune dynamics. The identification of interferon-related trajectories and T cell–associated features as correlates of outcome is of particular interest and highlights the translational potential of the study. The work is appreciated and represents an important contribution to the field. At the same time, several major aspects require further clarification and strengthening.

Major Points:
1. The use of TCR sequencing is currently limited to the assessment of T cell abundance. The dataset would allow a more

detailed analysis of clonality and diversity. It would be informative to evaluate whether clonal expansion correlates with survival and treatment response. In addition, the comparison of shared or enriched TCR motifs across patients could provide insight into antigen-specific responses and potential disease-associated patterns.
2. The role of the extent of resection in the context of neoadjuvant therapy remains unclear. It would be relevant to explore whether postoperative residual tumor volume influences immune activation or survival. Even with limited statistical power, an exploratory analysis could provide important clinical insights regarding the interaction between surgical strategy and immunotherapy efficacy.
3. The cohort size represents a limitation, particularly for subgroup and survival analyses. The study is well designed for mechanistic insights but not powered for definitive clinical conclusions. This aspect should be addressed more explicitly when interpreting outcome-related findings.
4. The handling of imaging-based endpoints requires further clarification. The discrepancy between progression-free survival and overall survival suggests a relevant impact of pseudoprogression. It should be specified which criteria were applied to distinguish progression from treatment-related effects and how these cases were managed within the analysis.
5. The central conclusions are based on bulk RNA sequencing, which limits cell type–specific interpretation. Interferon-related signatures cannot be clearly assigned to defined cellular compartments. This should be acknowledged as a limitation, as different immune cell populations may contribute differently to the observed signals.
6. The myeloid compartment is not explored in sufficient depth despite its known relevance in glioblastoma. The data suggest systemic inflammatory differences, but the contribution of specific myeloid subsets is not addressed. A more detailed discussion of this aspect would strengthen the biological interpretation of the findings.

Reviewer #3

(Remarks to the Author)
This manuscript describes a surgical window of opportunity clinical trial in 63 patients with recurrent GBM. Three arms were investigated (dual PD-1 +CTLA-4 ICB with nivo + ipi versus nivo alone versus placebo prior to resection). Multi-omic data was collected to better understand the treatment response amongst the 3 arms.

This study is clearly presented, and the results are interesting further underscoring that there may be a role for ICB in recurrent GBM in select patient populations and that dual ICB treatment prior to resection elicited a more robust intratumoral and systemic immune activation.

The median OS was longest for Arm 3 (465 days) which was unexpected since this arm did not receive any neoadjuvant treatment. The authors acknowledge it was a small N for this arm making interpretation challenging, but are there any other potential explanations beyond also noting the elevated TIL density in a few of the patients?

Reviewer #4

(Remarks to the Author)
This manuscript describes a surgical window-of-opportunity trial (NCT04606316) evaluating immune activation in 63 patients with relapsed, resectable glioblastoma.
While the presentation of the systemic and intratumoral immune activation data seems generally appropriate and may justify publication, the reviewer notes significant concerns regarding patient flow (CONSORT), and the statistical rigor of the survival analysis that needs to be adressed.

Detailed comments:

1. Revision CONSORT diagram:
The current patient flow is unclear and must be revised to meet standard reporting guidelines.
- The abstract mentions 63 participants, a number missing in the diagram.
- The 5 participants that withdrew consent after randomization should be included under “Randomized”.
- For the 7 participants, that were excluded for other reasons, it should be clarified if this was prior to randomization.
- The description of the set involving 58 participants that received study therapy should be renamed.
- Explicitly state which participants were used for the analysis of the clinical endpoint (PFS and OS) rather than including trial results.

2. Analysis of OS
The OS analysis requires significant clarification to address apparent discrepancies with the study protocol. Because the abstract highlights these survival benefits explicitly, there is a significant risk of misleading the clinical community. The authors must provide a transparent, Intention-to-Treat (ITT) analysis to justify there results. Without such a foundation, the survival claims must be re-formulated as strictly exploratory.
- The authors report using False Discovery Rate (FDR) adjustments, yet the protocol states no multiple test adjustments were planned. Which method was used? Which hypothesis set was used? Please clarify if this was a post-hoc decision and whether the hypothesis set was defined prior to unblinding. The explanation and clarification should also clarify the star used in Figure 6A.
- Table 1 reflects all treated participants, yet Figure 6A appears to use a modified set (N=38 in pooled Arms 1/3; N=20 in Arm 2, N=5 Screen Failed). Clarify if the 5 screen failures mentioned are patients who were randomized but never received treatment. The arms 1 and 3 are still short 3 additional subjects. Are these the ones mentioned in “Initially we planned to treat patients in Arms 1 and 3 with dual ICB in the post-surgical setting, while patients in Arm 2 received single agent

nivolumab until progression or unacceptable toxicities."?
- To ensure robustness and to preserve randomization as much as possible, Figure 6A should be presented for both the Intention-to-Treat (ITT) set (all randomized patients) and the Treated/As-Randomized set. The current modified analysis should be moved to the supplementary material.
- Figure 6A should be revised to clarify what #of blank rows and number of rows with impossible data represent.

Version 2:

Reviewer comments:

Reviewer #1

(Remarks to the Author)
The manuscript is much improved in its current form, and the authors have done an excellent job addressing the comments.

Reviewer #2

(Remarks to the Author)
No further comments

Reviewer #4

(Remarks to the Author)
The authors have adequately addressed the comments raised.

**Open Access** This Peer Review File is licensed under a Creative Commons Attribution 4.0 International License, which permits use, sharing, adaptation, distribution and reproduction in any medium or format, as long as you give appropriate credit to the original author(s) and the source, provide a link to the Creative Commons license, and indicate if changes were made.
In cases where reviewers are anonymous, credit should be given to 'Anonymous Referee' and the source.
The images or other third party material in this Peer Review File are included in the article's Creative Commons license, unless indicated otherwise in a credit line to the material. If material is not included in the article's Creative Commons license and your intended use is not permitted by statutory regulation or exceeds the permitted use, you will need to obtain permission directly from the copyright holder.
To view a copy of this license, visit https://creativecommons.org/licenses/by/4.0/

**REVIEWER COMMENTS**

Reviewer #1 (Remarks to the Author):

The authors conducted a phase II trial (NCT04606316) evaluating neoadjuvant dual PD-1 + CTLA-4 blockade versus PD-1 monotherapy or placebo in 63 patients with relapsed, resectable glioblastoma. The results reveal enhanced intratumoral and systemic immune activation with dual ICB. Patients receiving dual ICB showed higher TIL density, increased expression of interferon-related response genes, and improved overall survival compared with other arms. This study is interesting and timely. It is well written and presents important correlations. Combining Arms 1 and 3 results in a significant OS difference compared to PD-1 blockade alone (Arm 2). This work will be of great interest to the field. I have a few comments and questions:

1. Arm 3 patients received the same adjuvant dual therapy as Aim 1, but seemed to fare better, despite a smaller sample. With two long-term survivors each from Arm 3 (n=10) and Arm 1 (n=25), does this imply post-surgery adjuvant therapy (Arm 3) could be more effective than Arm 1? Just a thought looking at the data, I recognize sample size is small to make any conclusions.

**Response:** We thank the reviewer for the thoughtful and insightful comments. We agree that the observation that adjuvant ipilimumab plus nivolumab (Arm 3) may be more effective than neoadjuvant ipilimumab plus nivolumab (Arm 1) is intriguing and could have important implications for future therapeutic strategy design. However, we also recognize that a substantially larger sample size would be required to draw definitive conclusions.

To further explore this observation, we performed additional TCR-seq analyses comparing intratumoral and peripheral T cell repertoires across the three arms. The key findings are summarized below:

1. **Intratumoral TCR repertoire**: Tumors from Arm 3 showed a trend toward higher TCR diversity, as measured by the Gini–Simpson index, compared with Arms 1 and 2 (**Extended Data Figure 3K**). This metric emphasizes contributions from multiple expanded clones and is less sensitive to low-frequency clonotypes, making it suitable for TIL analyses.
2. **Peripheral (PBMC) TCR repertoire**: Using TRUST4 to infer TCR profiles from bulk RNA-seq data (**https://doi.org/10.1038/s41592-021-01142-2**). we observed that Arm 3 tended to maintain higher TCR diversity during adjuvant treatment cycles relative to Arm 1, based on Shannon diversity, which better captures the broad PBMC repertoire (**Reviewer-only figure 1A**)
3. Greater TCR diversity in both tumor (**Extended Data Figure 3L**) and adjuvant cycle PBMCs (**Reviewer-only figure 1B**) was associated with improved survival outcomes.

Together, these results suggest that although Arm 3 exhibits lower immediate intratumoral T cell and IFN activity at the time of surgery, it may sustain a broader systemic T cell repertoire over time. Prior studies have linked increased TCR diversity with improved survival and enhanced responses to ICI [1-3].

Because the PBMC findings are based on TRUST4-inferred repertoires in a small cohort, we have included these results as reviewer-only figures rather than in the main manuscript. Whether these observations hold up in larger cohorts, and the mechanisms underlying potential differences between neoadjuvant and adjuvant dual ICB, warrant further investigation.

*[editorial note: unpublished, confidential data redacted]*

2. The sentence in Lines 104–106 is unclear, making it difficult to follow the subsequent data. Were all patients in Arm 3 treated with dual ICB post-surgery? The mention of “n=3” is confusing and needs clarification.

**Response:** We thank the reviewer for highlighting this ambiguity and agree that the original wording could be confusing. This sentence was intended to explain why a small subset of patients in Arm 2 crossed over to receive dual ICB rather

than nivolumab alone. We have revised this sentence, along with the preceding text, to clarify the treatment assignments and explicitly explain this protocol amendment:

"*Following surgery, patients in Arms 1 and 3 received dual ICB, whereas patients in Arm 2 still received single-agent nivolumab until disease progression or unacceptable toxicity. The use of single-agent nivolumab for post-surgical treatment in Arm 2 was based on our prior study suggesting that neoadjuvant + adjuvant single-agent anti–PD-1 therapy (with pembrolizumab) may be associated with improved survival in recurrent glioblastoma[4]. However, our subsequent validation trial did not confirm a survival benefit with neoadjuvant and adjuvant single-agent pembrolizumab [5]. Considering these findings, the protocol for the current ipilimumab + nivolumab trial was amended at the very end of the trial to treat the remaining Arm 2 patients with dual ICB after surgery. As this amendment occurred late in the trial, it affected only a small subset of patients: specifically, 3 of 23 patients in Arm 2 received neoadjuvant nivolumab followed by adjuvant ipilimumab + nivolumab, whereas the remaining 20 patients received single-agent nivolumab both neoadjuvantly and adjuvantly.*"

3. In Figure 3B, the second-row images are neither quantified nor discussed in the text. The staining includes myeloid markers (CD14, CD206, HLA-DR). Since these data are available, it would strengthen the manuscript to quantify and characterize the myeloid profile from the multiplex IF staining, add corresponding graphs next to the images, and discuss the findings.

**Response:** We thank the reviewer for this constructive suggestion. We have now quantified the myeloid subsets from the multiplex immunofluorescence (mIF) staining, added the corresponding graphs and the following new text describing these findings:

"*We also performed mIF staining for CD45, CD4, CD8, the pan-myeloid marker CD14, the perivascular macrophage marker CD206, and the pro-inflammatory macrophage marker HLA-DR. Although these differences did not reach statistical significance, both $CD4^+$ and $CD8^+$ T cell densities were highest in Arm 1, consistent with the TCR-based TIL density quantification. Notably, Arm 1 also exhibited the highest density of monocyte-derived macrophages (**Extended Data Figure 3A**), suggesting increased overall immune infiltration following dual ICB. Among these macrophages, the density of HLA-$DR^+$ macrophages were highest in Arm 1 (**Extended Data Figure 3A**). Given that interferon-γ is a known inducer of HLA-DR expression on myeloid cells, this observation is concordant with the elevated interferon signaling observed in Arm 1 by bulk RNA-seq.*"

4. Please provide higher-resolution inserts for Figure 3B. It would also be beneficial to align these images with the quantification in Figure 3A, as the visual differences appear striking while the quantitative analysis shows no significant change.

**Response:** We thank the reviewer for this constructive suggestion. We have revised **Figure 3B** by selecting representative regions that better reflect the overall quantitative findings in Figure 3A. We also added higher-resolution (20×) inserts to better illustrate the staining patterns.

5. The method used to define high and low tumor mutational burden (TMB) is unclear. Please specify how these groups were determined, as this information is missing from the Methods section.

**Response:** We thank the reviewer for noting that information regarding TMB was missing from the Methods section. We have now added the following text to the revised Methods:

"*TMB was calculated as the number of nonsynonymous mutations per megabase (Mb) of genome covered, based on one of three clinically validated next-generation sequencing platforms (FoundationOne, OncoPanel, or MSK-IMPACT), which interrogate approximately 300–500 genes known to be recurrently altered in solid tumors. For the Kaplan–Meier analysis*

*shown in Figure 3F, patients were stratified into high- and low-TMB groups using the cohort median TMB value (4.1 mutations/Mb) as the cutoff.*

*This cutoff is lower than the commonly used threshold of 10 mutations/Mb and is consistent with the typically low TMB observed in glioblastoma*."

Reviewer #2 (Remarks to the Author):

This manuscript presents a well-conducted and clinically relevant window-of-opportunity study investigating dual versus single immune checkpoint inhibition in recurrent glioblastoma. The integration of neoadjuvant and adjuvant treatment with comprehensive immune profiling provides valuable insights into treatment-associated immune dynamics. The identification of interferon-related trajectories and T cell–associated features as correlates of outcome is of particular interest and highlights the translational potential of the study. The work is appreciated and represents an important contribution to the field. At the same time, several major aspects require further clarification and strengthening.

Major Points:
1. The use of TCR sequencing is currently limited to the assessment of T cell abundance. The dataset would allow a more detailed analysis of clonality and diversity. It would be informative to evaluate whether clonal expansion correlates with survival and treatment response. In addition, the comparison of shared or enriched TCR motifs across patients could provide insight into antigen-specific responses and potential disease-associated patterns.

**Response:** We thank the reviewer for this helpful suggestion. We have now included a more in-depth analysis of the tumor TCR sequencing data in the revised manuscript. The main observations are as follows:

1. Arm 1 (dual ICB) demonstrated trends toward higher clonality (1 – normalized Shannon entropy), higher diversity (Gini–Simpson index), and greater clone richness (number of unique clonotypes) compared with other arms (**Extended Data Figure 3K**). Notably, the Gini–Simpson index provides a complementary and non-redundant measure to Shannon entropy (i.e., not simply the inverse of clonality), as it emphasizes the contribution of many expanded clones rather than one or a few highly dominant clones. The concurrent increase of clonality, diversity and clone richness suggest that dual ICB induces a focused yet polyclonal T cell response in the tumor: increased T cell infiltration (higher richness), a substantial contribution from multiple clonotypes (higher diversity), and preferential expansion of selected clones (higher clonality).
2. Higher TCR diversity (Gini–Simpson index) was associated with improved survival outcomes (**Extended Data Figure 3L**), although this did not reach statistical significance in Cox proportional hazards models. Importantly, when combined with TIL density, patients with both high TIL infiltration and higher TCR diversity exhibited significantly improved survival (**Extended Data Figure 3M**).
3. For each arm, we analyzed the top expanded clones and observed minimal sharing of TCR sequences across patients, indicating that the expanded T cell responses are largely private and likely targeting patient-specific antigens (**Extended Data Figure 3N**).

We have amended the text in the Results section (Lines 208-227) to include a detailed description of how elevated TIL density and TCR diversity together was linked to longer survival.

2. The role of the extent of resection in the context of neoadjuvant therapy remains unclear. It would be relevant to explore whether postoperative residual tumor volume influences immune activation or survival. Even with limited statistical power, an exploratory analysis could provide important clinical insights regarding the interaction between surgical strategy and immunotherapy efficacy.

*[editorial note: unpublished, confidential data redacted]*

**Response:** We thank the reviewer for this helpful suggestion. We have now included additional analyses of postoperative residual tumor volume in the revised manuscript. As shown in **Figure 3G**, smaller postoperative residual enhancing tumor volume is associated with improved OS, regardless of treatment arm and other clinical variables.

Our group previously demonstrated that postoperative tumor volume is a prognostic factor for survival in newly diagnosed GBM patients treated with chemoradiation [6]. The current study extends this observation to recurrent GBM patients receiving immunotherapy, supporting its role as a robust prognostic indicator for GBM patients.

We also assessed the relationship between postoperative residual tumor volume and intratumoral immune features, including TIL density and IFN signature scores, and found no significant correlations (**Reviewer-only Figure 2**). These findings suggest that postoperative residual tumor volume influences clinical outcomes, potentially by reducing overall tumor burden and enhancing the effectiveness of adjuvant therapies. The interaction between residual tumor burden and the tumor's immune activation, as well as its specific impact on immunotherapy efficacy, needs further investigation in larger cohorts.

3. The cohort size represents a limitation, particularly for subgroup and survival analyses. The study is well designed for mechanistic insights but not powered for definitive clinical conclusions. This aspect should be addressed more explicitly when interpreting outcome-related findings.

**Response:** We thank the reviewer for pointing out this important limitation of our study. We have added an explicit statement addressing this limitation in the Discussion section**.**

*"In summary, our study provides important mechanistic insights into the intratumoral and systemic immune alterations induced by dual ICB (aPD-1 + aCTLA-4) in recurrent GBM. However, the relatively small cohort size limits the robustness of subgroup and survival analyses and precludes definitive clinical conclusions. Therefore, these findings should be considered hypothesis-generating and require validation in larger, adequately powered cohorts."*

4. The handling of imaging-based endpoints requires further clarification. The discrepancy between progression-free survival and overall survival suggests a relevant impact of pseudoprogression. It should be specified which criteria were applied to distinguish progression from treatment-related effects and how these cases were managed within the analysis.

**Response:** We apologize for this lack of clarity. We have utilized the updated response assessment in neuro-oncology (RANO 2.0) criteria for post-surgical monitoring of patients, including the requirement of confirmation of disease progression during the first 6 months of treatment. This confirmation of progression includes the requirement of continued tumor growth beyond initial progressive enhancement - essentially 2 PD events in a row – otherwise, the initial progressive enhancement event is defined as pseudoprogression and the patient is treated until the next progressive disease event. We have included this in the Methods section.

5. The central conclusions are based on bulk RNA sequencing, which limits cell type–specific interpretation. Interferon-related signatures cannot be clearly assigned to defined cellular compartments. This should be acknowledged as a limitation, as different immune cell populations may contribute differently to the observed signals.

**Response:** We agree with the reviewer that bulk RNA sequencing limits our ability to resolve changes in individual cell populations and their signaling pathways in response to dual ICB treatment. We have added this limitation to the Discussion.

*"Furthermore, bulk RNA sequencing limits the cell type–specific resolution of IFN sources and their downstream effects. Future single-cell profiling of this cohort will be essential to delineate the cellular origins and functional consequences of IFN signaling within the tumor microenvironment."*

In addition, we leveraged our previously published single-cell RNAseq data from tumor infiltrating immune cells from recurrent GBM treated with or without neoadjuvant anti–PD-1 (pembrolizumab) (Lee Nat Commun, 2022). From this dataset, we derived gene signatures for distinct lymphoid and myeloid subsets. We then tested these signatures in our bulk RNA-seq data.

As shown in **Figure 2C** and **Extended Data Figure 2B**, multiple T cell and myeloid signatures showed consistent enrichment patterns across samples. These signatures were significantly enriched in Arm A (Ipi+Nivo) compared with Arm C (placebo) and/or Arm B (Nivo only). This pattern suggests increased overall immune infiltration with dual ICB.

We further correlated the interferon signature (Ayers_et_al_IFN_genes) with these subset-specific signatures. $GZMK^+CD8A^+$ T cells, dendritic cells, and monocytes showed the strongest correlations, while several macrophage subsets showed more modest but still notable correlations (**Extended Data Figure 3D** and **Supplementary Table 4**). These results suggest that the elevated IFN signal reflects contributions from both lymphoid and myeloid cells. However, the precise roles of these populations in shaping the tumor microenvironment and treatment response will require further investigation using single-cell–level approaches.

6. The myeloid compartment is not explored in sufficient depth despite its known relevance in glioblastoma. The data suggest systemic inflammatory differences, but the contribution of specific myeloid subsets is not addressed. A more detailed discussion of this aspect would strengthen the biological interpretation of the findings.

**Response:** We thank the reviewer for the constructive suggestions. We have expanded our analysis of PBMCs, by leveraging our previously published PBMC scRNA-seq dataset from recurrent GBM patients (Lee Nat Commun, 2022) as a reference and performed CIBERSORTx deconvolution to infer temporal changes in immune cell composition. This analysis suggested an increase in $CD14^+$ monocytes and proliferating T cells following neoadjuvant dual ICB treatment, although these changes did not reach statistical significance (**Extended Data Figure 4B**).

To further investigate these associations, we correlated changes in IFN scores (Δ from baseline to PostNeo or adjuvant cycles 1 and 2) with corresponding changes in inferred immune cell fractions. Among all compartments, $CD14^+$ monocytes consistently showed the strongest positive correlations with IFN (**Extended Data Figure 4C**).

Together, these results suggest that neoadjuvant dual ICB induces systemic inflammation. While proliferating T cells exhibit an early systemic response, the monocyte compartment appears to play a central role in sensing and responding to inflammatory signals (e.g., IFN-related pathways), and may contribute to sustained systemic inflammation.

Reviewer #3 (Remarks to the Author):

This manuscript describes a surgical window of opportunity clinical trial in 63 patients with recurrent GBM. Three arms were investigated (dual PD-1 +CTLA-4 ICB with nivo + ipi versus nivo alone versus placebo prior to resection). Multi-omic data was collected to better understand the treatment response amongst the 3 arms.

This study is clearly presented, and the results are interesting further underscoring that there may be a role for ICB in recurrent GBM in select patient populations and that dual ICB treatment prior to resection elicited a more robust intratumoral and systemic immune activation.

The median OS was longest for Arm 3 (465 days) which was unexpected since this arm did not receive any neoadjuvant treatment. The authors acknowledge it was a small N for this arm making interpretation challenging, but are there any other potential explanations beyond also noting the elevated TIL density in a few of the patients?

**Response:** We thank the reviewer for highlighting this interesting difference between Arms 1 and 3. We have performed additional analyses to further investigate this aspect; please see our response to Reviewer 1, Comment 1 for detailed results.

Reviewer #4 (Remarks to the Author):

This manuscript describes a surgical window-of-opportunity trial (NCT04606316) evaluating immune activation in 63 patients with relapsed, resectable glioblastoma.
While the presentation of the systemic and intratumoral immune activation data seems generally appropriate and may justify publication, the reviewer notes significant concerns regarding patient flow (CONSORT), and the statistical rigor of the survival analysis that needs to be addressed.

Detailed comments:

1. Revision CONSORT diagram:
The current patient flow is unclear and must be revised to meet standard reporting guidelines.
- The abstract mentions 63 participants, a number missing in the diagram.
- The 5 participants that withdrew consent after randomization should be included under “Randomized”.
- For the 7 participants, that were excluded for other reasons, it should be clarified if this was prior to randomization.
- The description of the set involving 58 participants that received study therapy should be renamed.
- Explicitly state which participants were used for the analysis of the clinical endpoint (PFS and OS) rather than including trial results.

2. Analysis of OS
The OS analysis requires significant clarification to address apparent discrepancies with the study protocol. Because the abstract highlights these survival benefits explicitly, there is a significant risk of misleading the clinical community. The authors must provide a transparent, Intention-to-Treat (ITT) analysis to justify there results. Without such a foundation, the survival claims must be re-formulated as strictly exploratory.

**Response:** We thank the reviewer for identifying the discrepancies. We revised the CONSORT diagram, Table 1, Extended Data Figure 1A, Figure 1 and Figure 6 to clarify the analysis populations. The five patients previously labeled as screen failures were actually patients who were randomized but withdrew consent before treatment initiation. These patients have now been included in the randomized/ITT population according to their assigned arms, resulting in an ITT population of n = 63. The treated population, defined as randomized patients who received any study therapy, remains n = 58. OS was analyzed in the ITT population, while PFS was analyzed among patients with available PFS data, because PFS data were unavailable for the five patients who withdrew consent before treatment initiation.

- The authors report using False Discovery Rate (FDR) adjustments, yet the protocol states no multiple test adjustments were planned. Which method was used? Which hypothesis set was used? Please clarify if this was a post-hoc decision and whether the hypothesis set was defined prior to unblinding. The explanation and clarification should also clarify the star used in Figure 6A.

**Response:** We clarified that no FDR adjustment was applied to the clinical endpoint analyses. FDR correction was applied only to the differential gene expression and gene set analyses to account for multiple testing across genes and gene sets. The prior statement indicating FDR adjustment for clinical endpoints analyses was included in error and has been removed; all reported p-values are nominal and unadjusted. Accordingly, the asterisk in the previous version of Figure 6, which was intended to denote nominal $p < 0.05$, has been removed, as there are no statistically significant comparisons in the updated Figure 6A.

- Table 1 reflects all treated participants, yet Figure 6A appears to use a modified set (N=38 in pooled Arms 1/3; N=20 in Arm 2, N=5 Screen Failed). Clarify if the 5 screen failures mentioned are patients who were randomized but never received treatment. The arms 1 and 3 are still short 3 additional subjects. Are these the ones mentioned in “Initially we

planned to treat patients in Arms 1 and 3 with dual ICB in the post-surgical setting, while patients in Arm 2 received single agent nivolumab until progression or unacceptable toxicities."?

- To ensure robustness and to preserve randomization as much as possible, Figure 6A should be presented for both the Intention-to-Treat (ITT) set (all randomized patients) and the Treated/As-Randomized set. The current modified analysis should be moved to the supplementary material.
- Figure 6A should be revised to clarify what #of blank rows and number of rows with impossible data represent.

**Response:** We have generated a new Figure 6A and revised Extended Data Figure 6A to include all 63 randomized (ITT) patients. The modified OS analysis has been moved to Extended Data Figure 6B.

In addition, Figure 6A and Extended Data Figures 6A–B have been updated to display the original n at day 0 to improve clarity and minimize confusion.

We also removed the tables showing the number of blank rows and rows with invalid data, as these were intermediate outputs generated by GraphPad and not intended for presentation.

**Cited Publications**


1 Kjær, A. *et al.* Low T cell diversity associates with poor outcome in bladder cancer: A comprehensive longitudinal analysis of the T cell receptor repertoire. *Cell Reports Medicine* **6** (2025). https://doi.org/10.1016/j.xcrm.2025.102101

2 Valpione, S. *et al.* Immune-awakening revealed by peripheral T cell dynamics after one cycle of immunotherapy. *Nat Cancer* **1**, 210-221 (2020). https://doi.org/10.1038/s43018-019-0022-x

3 Yost, K. E. *et al.* Clonal replacement of tumor-specific T cells following PD-1 blockade. *Nat Med* **25**, 1251-1259 (2019). https://doi.org/10.1038/s41591-019-0522-3

4 Cloughesy, T. F. *et al.* Neoadjuvant anti-PD-1 immunotherapy promotes a survival benefit with intratumoral and systemic immune responses in recurrent glioblastoma. *Nat Med* **25**, 477-486 (2019). https://doi.org/10.1038/s41591-018-0337-7

5 McFaline-Figueroa, J. R. *et al.* Neoadjuvant anti-PD1 immunotherapy for surgically accessible recurrent glioblastoma: clinical and molecular outcomes of a stage 2 single-arm expansion cohort. *Nat Commun* **15**, 10757 (2024). https://doi.org/10.1038/s41467-024-54326-7

6 Patel, S. *et al.* Are pediatric brain tumors on the rise in the USA? Significant incidence and survival findings from the SEER database analysis. *Childs Nerv Syst* **30**, 147-154 (2014). https://doi.org/10.1007/s00381-013-2307-1
