# Supplementary material for: Neoadjuvant PD1 plus CTLA-4 immune checkpoint blockade in surgically resectable recurrent glioblastoma: a randomized surgical window-of-opportunity trial
Source: Nat Commun. 2026 Sep 5;17:10560. doi: 10.1038/s41467-026-77433-z (PMC13642311; doi:10.1038/s41467-026-77433-z)
Supplement: Supplementary file 3 [file 41467_2026_77433_MOESM1_ESM.pdf]

	KPS score 70–80	KPS score 90–100
**Arm 1**	6	19
**Arm 2**	7	16
**Arm 3**	2	8

Supplementary Table 1 Distribution of patients with KPS scores of 70–80 or 90–100. A Chi-square test of independence showed no significant difference in KPS distribution across Arms ($\chi^2 = 0.475$, $p = 0.788$). Arm 1: Neoadjuvant Nivo + Ipi; Arm 2: Neoadjuvant Nivo alone; Arm 3: Placebo infusion prior to surgery.

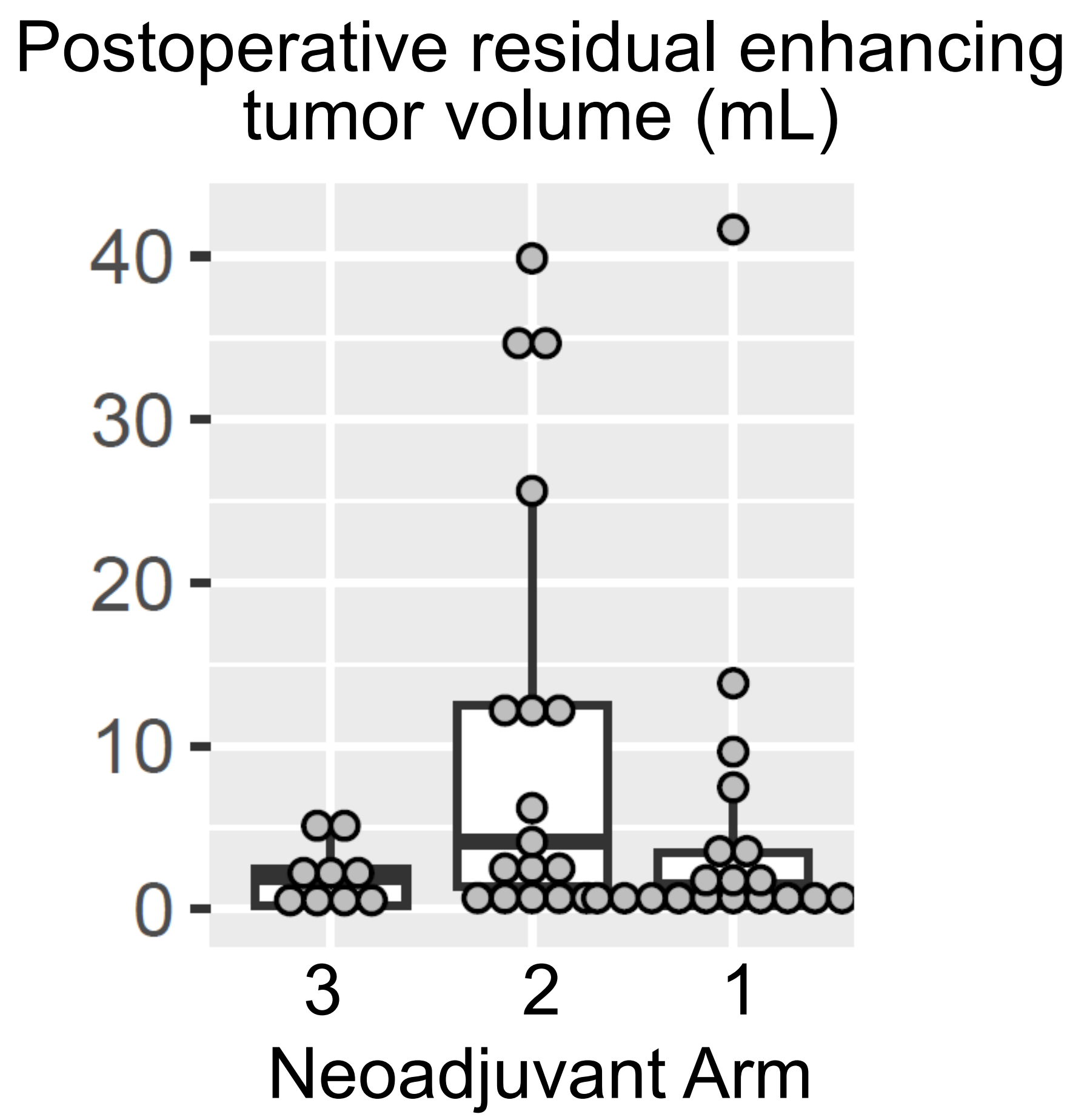

Supplementary Figure 1 Boxplot of postoperative residual tumor volume across three neoadjuvant treatment arms. Each dot represents a patient. The lower and upper bounds indicate the 25th and 75th percentiles, and the middle lines the median values. Because of near-zero variance in Arm 3 and marked skew in Arm 2, pairwise permutation tests (10,000 iterations) were applied for pairwise comparisons. No comparison reached statistical significance:
Arms 1 vs 2 (median difference = –1.55 mL, p = 0.13),
Arms 1 vs 3 (median difference = –0.54 mL, p = 0.64), and
Arms 2 vs 3 (median difference = 1.01 mL, p = 0.40).
Arm 1: Neoadjuvant Nivo + Ipi; Arm 2: Neoadjuvant Nivo alone; Arm 3: Placebo infusion prior to surgery.
Arm 1: n = 24; Arm 2: n = 22; Arm 3: n=10.

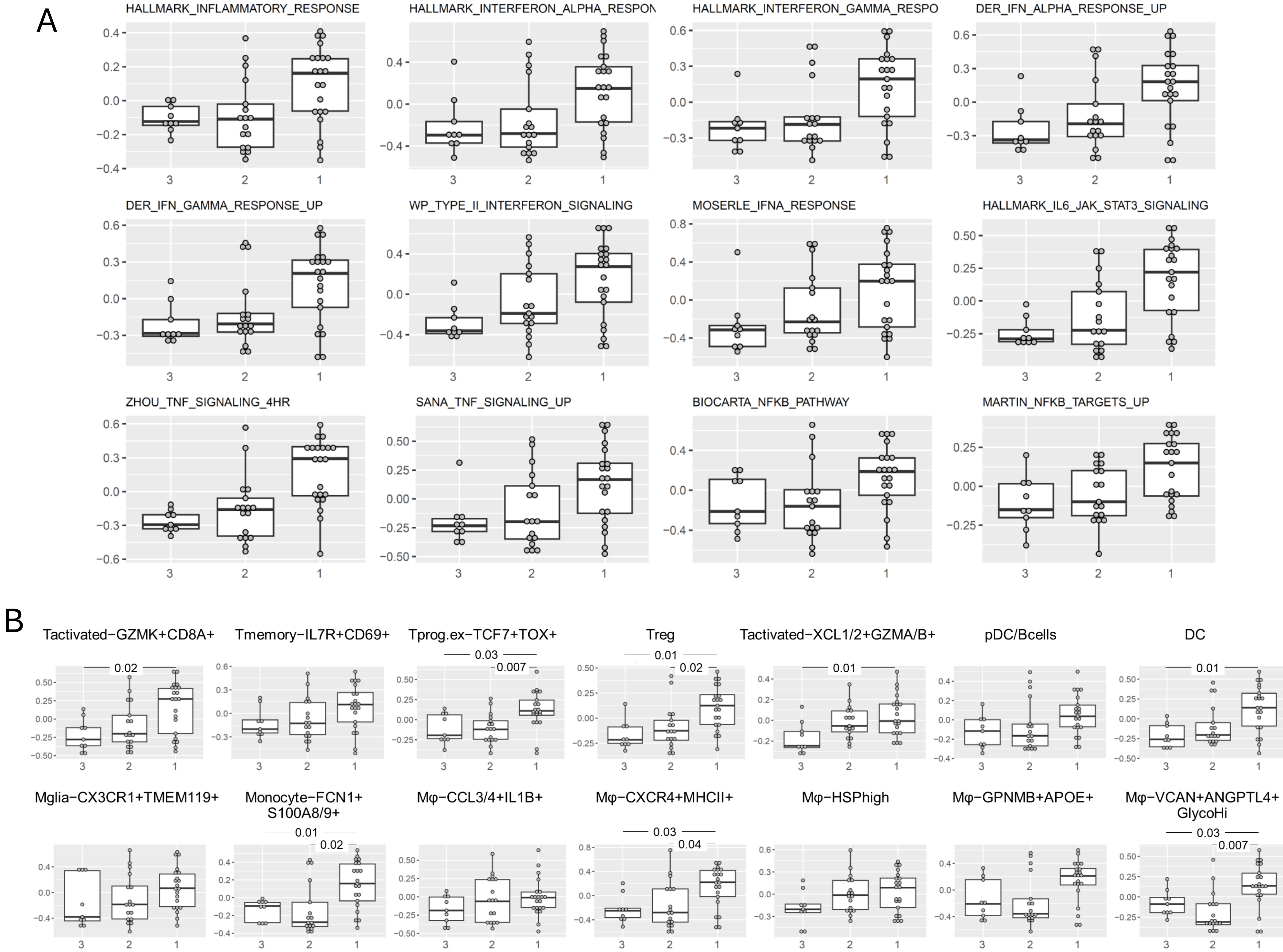

Supplementary Figure 2 (A) Boxplots of GSVA scores for interferon and T-cell–related gene signatures across three treatment arms. All signatures were significantly upregulated in Arm 1 compared to Arm 3 ($p < 0.05$). **(B)** Boxplots of GSVA scores for the lymphoid and myeloid subset-specific gene signatures across three treatment arms. In each boxplot, dots represent individual patients; boxes indicate the interquartile range (25th–75th percentiles); and horizontal lines denote median values. P values were calculated using a two-sided Wilcoxon rank-sum test. Multiple comparisons were corrected using False Discovery Rate (FDR) method adjustment. Arm 1: Neoadjuvant Nivo + Ipi; Arm 2: Neoadjuvant Nivo alone; Arm 3: Placebo infusion prior to surgery. Arm 1: n = 21; Arm 2: n = 17; Arm 3: n=9.

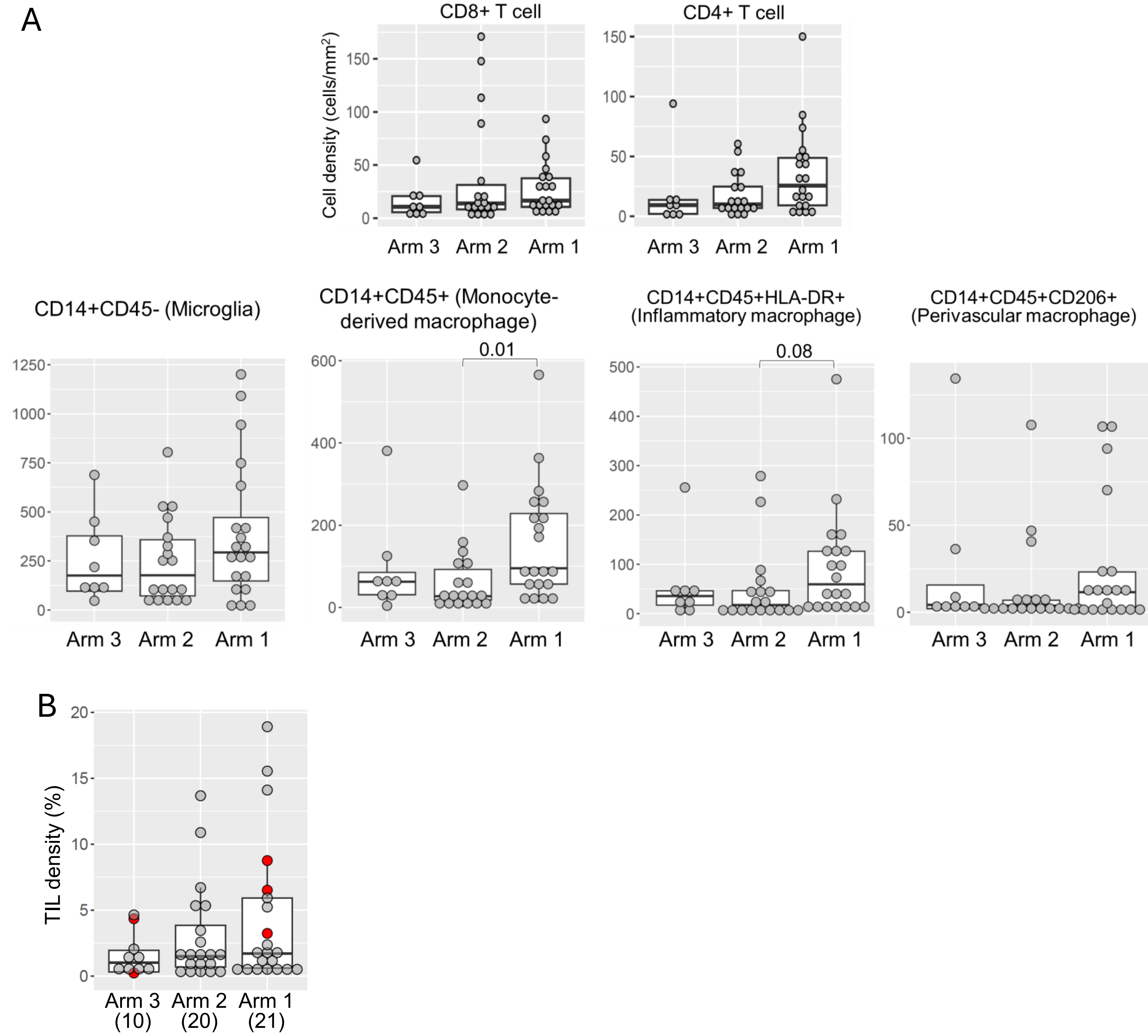

Supplementary Figure 3 (A) Boxplots of T cell and myeloid cell density quantified by mIF. P-values were calculated using a two-sided Wilcoxon rank-sum test. Arm 1: n = 20; Arm 2: n = 18; Arm 3: n = 8. **(B)** Boxplot of TIL density across three neoadjuvant treatment arms. Arm 1: n = 21; Arm 2: n = 20; Arm 3: n = 10). The red dot indicate the patients that achieved long term survival. In each boxplot, dots represent individual patients; boxes indicate the interquartile range (25th–75th percentiles); and horizontal lines denote median values. The lower and upper bounds indicate the 25th and 75th percentiles, and the middle lines the median values. Arm 1: Neoadjuvant Nivo + Ipi; Arm 2: Neoadjuvant Nivo alone; Arm 3: Placebo infusion prior to surgery.

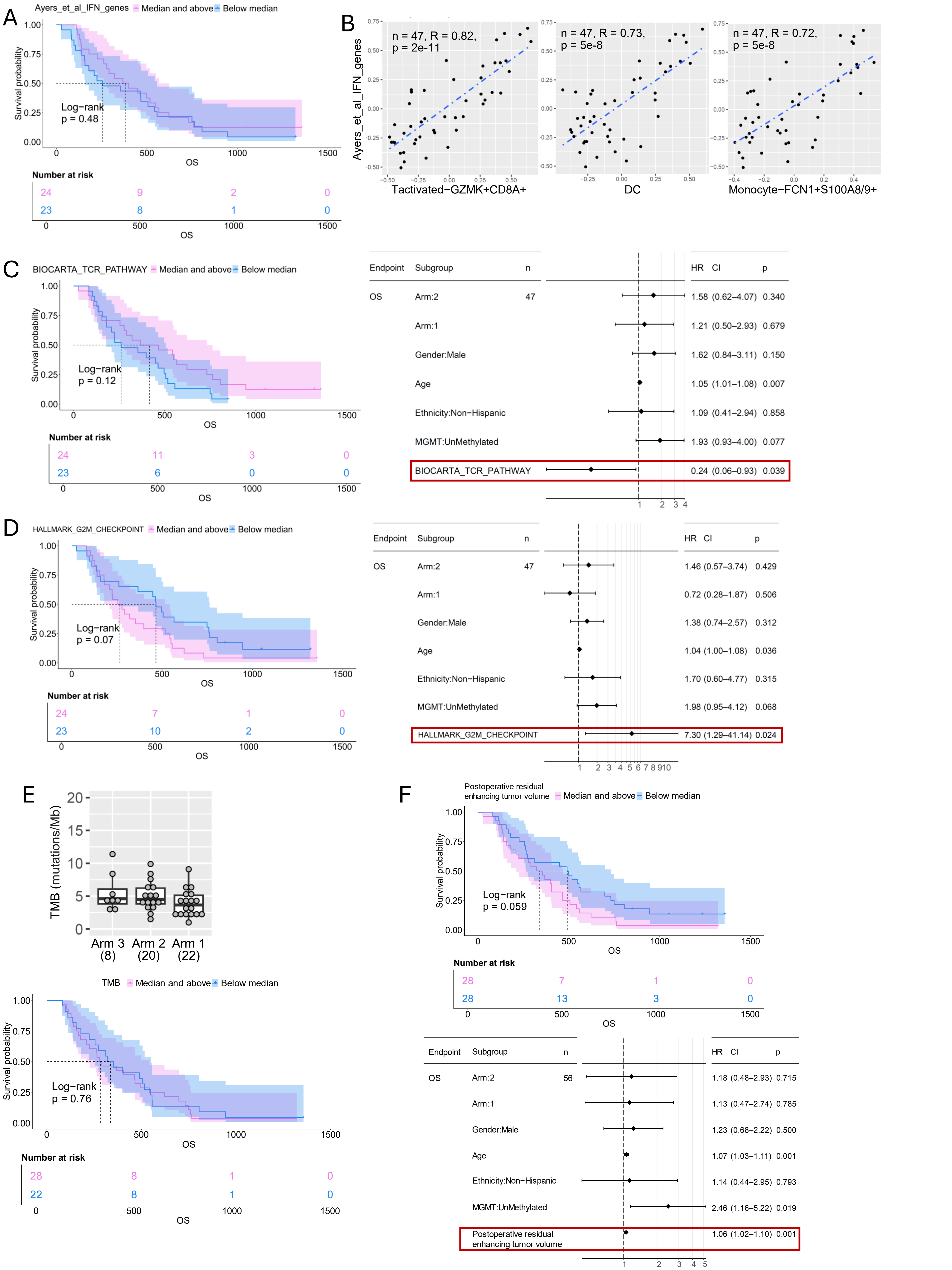

A
Ayers_et_al_IFN_genes Median and above Below median
Survival probability
Log−rank
p = 0.48
OS
Number at risk
24 9 2 0
23 8 1 0
B
Ayers_et_al_IFN_genes
n = 47, R = 0.82,
p = 2e-11
Tactivated−GZMK+CD8A+
n = 47, R = 0.73,
p = 5e-8
DC
n = 47, R = 0.72,
p = 5e-8
Monocyte−FCN1+S100A8/9+
C
BIOCARTA_TCR_PATHWAY Median and above Below median
Survival probability
Log−rank
p = 0.12
OS
Number at risk
24 11 3 0
23 6 0 0
Endpoint Subgroup n HR CI p
OS Arm:2 47 1.58 (0.62–4.07) 0.340
Arm:1 1.21 (0.50–2.93) 0.679
Gender:Male 1.62 (0.84–3.11) 0.150
Age 1.05 (1.01–1.08) 0.007
Ethnicity:Non−Hispanic 1.09 (0.41–2.94) 0.858
MGMT:UnMethylated 1.93 (0.93–4.00) 0.077
BIOCARTA_TCR_PATHWAY 0.24 (0.06–0.93) 0.039
D
HALLMARK_G2M_CHECKPOINT Median and above Below median
Survival probability
Log−rank
p = 0.07
OS
Number at risk
24 7 1 0
23 10 2 0
Endpoint Subgroup n HR CI p
OS Arm:2 47 1.46 (0.57–3.74) 0.429
Arm:1 0.72 (0.28–1.87) 0.506
Gender:Male 1.38 (0.74–2.57) 0.312
Age 1.04 (1.00–1.08) 0.036
Ethnicity:Non−Hispanic 1.70 (0.60–4.77) 0.315
MGMT:UnMethylated 1.98 (0.95–4.12) 0.068
HALLMARK_G2M_CHECKPOINT 7.30 (1.29–41.14) 0.024
E
TMB (mutations/Mb)
Arm 3 (8)
Arm 2 (20)
Arm 1 (22)
TMB Median and above Below median
Survival probability
Log−rank
p = 0.76
OS
Number at risk
28 8 1 0
22 8 1 0
F
Postoperative residual enhancing tumor volume Median and above Below median
Survival probability
Log−rank
p = 0.059
OS
Number at risk
28 7 1 0
28 13 3 0
Endpoint Subgroup n HR CI p
OS Arm:2 56 1.18 (0.48–2.93) 0.715
Arm:1 1.13 (0.47–2.74) 0.785
Gender:Male 1.23 (0.68–2.22) 0.500
Age 1.07 (1.03–1.11) 0.001
Ethnicity:Non-Hispanic 1.14 (0.44–2.95) 0.793
MGMT:UnMethylated 2.46 (1.16–5.22) 0.019
Postoperative residual enhancing tumor volume 1.06 (1.02–1.10) 0.001

Supplementary Figure 4 (A) Kaplan–Meier analysis showing the impact of a representative interferon gene signature, Ayers_et_al_IFN_genes, on overall survival. **(B)** Scatter plots of correlations between GSVA scores for lymphoid and myeloid subset signatures and the Ayers_et_al_IFN_genes signature (sample number n, Pearson R and FDR-adjusted P values shown). **(C)** Cox proportional hazards regression and Kaplan–Meier analysis showing the impact of a representative T cell-related gene signature, BIOCARTA_TCR_PATHWAY, on overall survival. **(D)** Cox proportional hazards regression and Kaplan–Meier analysis showing the impact of a representative cell cycle gene signature, HALLMARK_G2M_CHECKPOINT, on overall survival. **(E)** (top) Boxplots of tumor mutational burden. Dots represent individual patients; boxes indicate the interquartile range (25th–75th percentiles); and horizontal lines denote median values. (bottom) Kaplan–Meier curves and Cox-Ph model evaluating the effect of tumor mutational burden (TMB) on overall survival. **(F)** Kaplan–Meier curves and Cox-PH model evaluating the effect of postoperative tumor volume on OS. For Cox-Ph models, n indicate the number of patients with available clinical covariates, diamonds at the centers of the error bars indicate HR estimates, error bars denote 95% confidence intervals (CI), and P values are based on two-sided Wald statistics (without adjustment for multiple comparisons). Log-rank P values are shown on the Kaplan-Meier plots. Arm 1: Neoadjuvant Nivo + Ipi; Arm 2: Neoadjuvant Nivo alone; Arm 3: Placebo infusion prior to surgery.

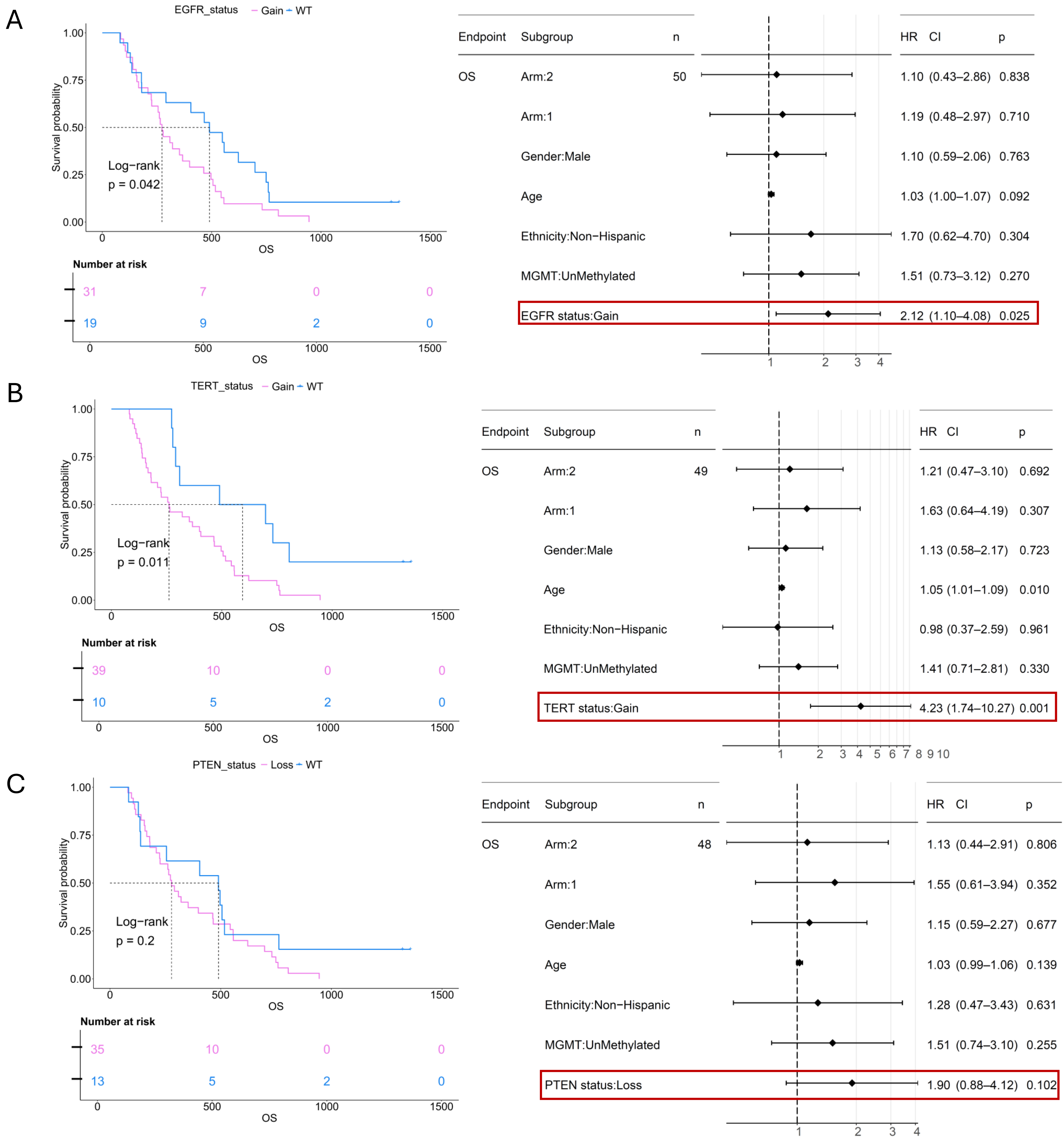

Supplementary Figure 5 (A) Cox proportional hazards regression and Kaplan–Meier analysis showing the impact of EGFR gain-of-function on overall survival. **(B)** Cox proportional hazards regression and Kaplan–Meier analysis showing the impact of TERT gain-of-function on overall survival. **(C)** Cox proportional hazards regression and Kaplan–Meier analysis showing the impact of PTEN loss on overall survival**.** For Cox-Ph models, n indicate the number of patients with available clinical covariates, diamonds at the centers of the error bars indicate HR estimates, error bars denote 95% confidence intervals (CI), and P values are based on two-sided Wald statistics (without adjustment for multiple comparisons). Log-rank P values are shown on the Kaplan-Meier plots.

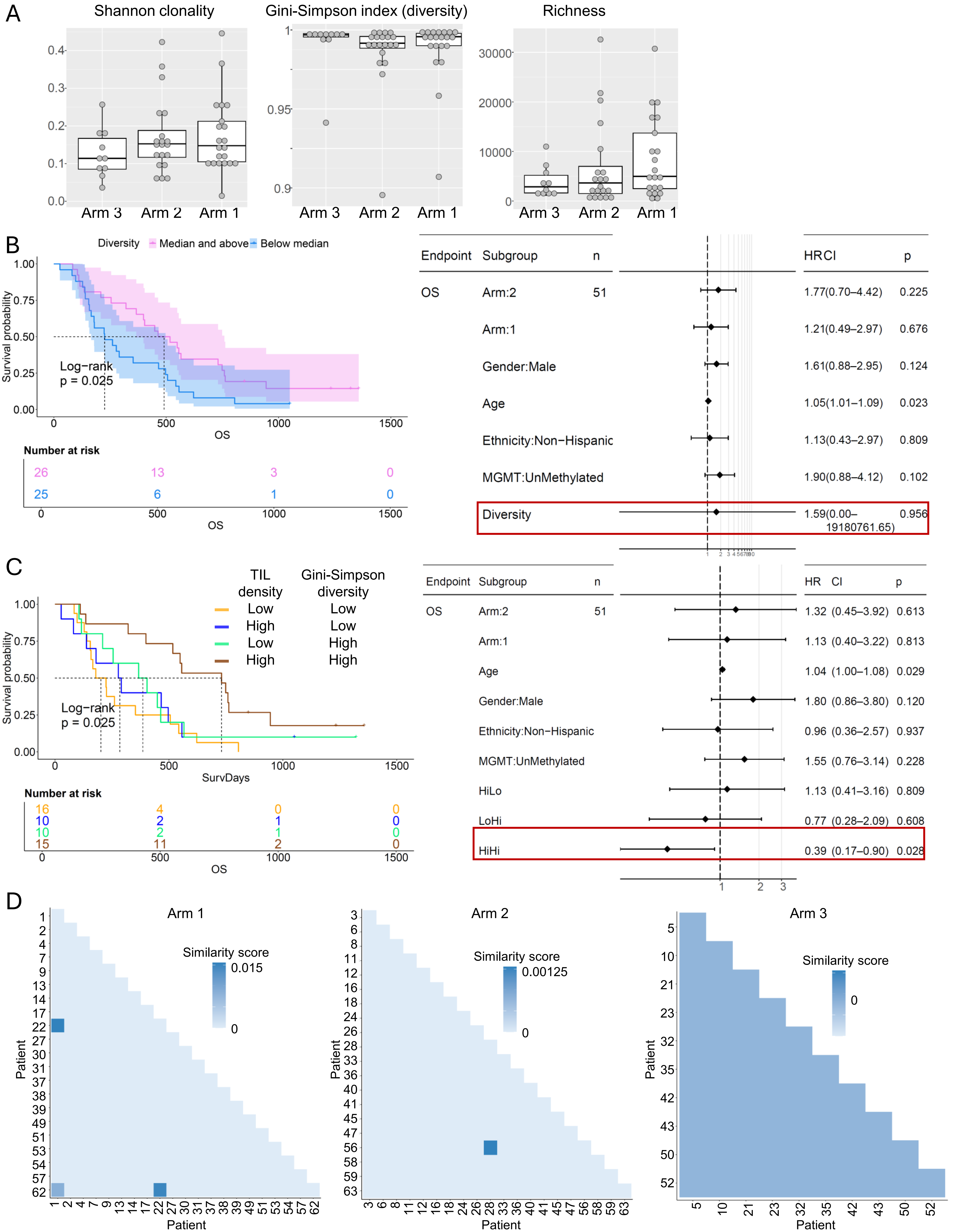

A
Shannon clonality
Gini-Simpson index (diversity)
Richness
Arm 3
Arm 2
Arm 1
B
Diversity
Median and above
Below median
Survival probability
Log-rank
p = 0.025
OS
Number at risk
26 13 3 0
25 6 1 0
Endpoint Subgroup n HR CI p
OS Arm:2 51 1.77(0.70–4.42) 0.225
Arm:1 1.21(0.49–2.97) 0.676
Gender:Male 1.61(0.88–2.95) 0.124
Age 1.05(1.01–1.09) 0.023
Ethnicity:Non−Hispanic 1.13(0.43–2.97) 0.809
MGMT:UnMethylated 1.90(0.88–4.12) 0.102
Diversity 1.59(0.00–19180761.65) 0.956
C
TIL density
Gini-Simpson diversity
Low Low
High Low
Low High
High High
Survival probability
Log-rank
p = 0.025
SurvDays
Number at risk
16 4 0 0
10 2 1 0
10 2 1 0
15 11 2 0
OS
Endpoint Subgroup n HR CI p
OS Arm:2 51 1.32 (0.45–3.92) 0.613
Arm:1 1.13 (0.40–3.22) 0.813
Age 1.04 (1.00–1.08) 0.029
Gender:Male 1.80 (0.86–3.80) 0.120
Ethnicity:Non−Hispanic 0.96 (0.36–2.57) 0.937
MGMT:UnMethylated 1.55 (0.76–3.14) 0.228
HiLo 1.13 (0.41–3.16) 0.809
LoHi 0.77 (0.28–2.09) 0.608
HiHi 0.39 (0.17–0.90) 0.028
D
Arm 1
Arm 2
Arm 3
Similarity score
0.015
0.00125
0
Patient

Supplementary Figure 6 (A) Boxplots of T-cell clonality, diversity, and richness across three neoadjuvant treatment arms, computed from bulk TCR-seq data from 51 patients. The values in parentheses indicate the number of patients. Each dot represents a patient. The lower and upper bounds indicate the 25th and 75th percentiles, and the middle lines the median values. Arm 1: Neoadjuvant Nivo + Ipi; Arm 2: Neoadjuvant Nivo alone; Arm 3: Placebo infusion prior to surgery. Arm 3: n = 10; Arm 2: n = 20; Arm 1: n = 21). **(B)** Cox proportional hazards regression and Kaplan–Meier analysis showing the impact of intratumoral TCR diversity on overall survival. **(C)** Cox proportional hazards regression and Kaplan–Meier analysis showing the impact of intratumoral TIL density and TCR diversity on overall survival. **(D)** Pairwise similarity scores of TCR clonotypes across samples from different treatment arms. The similarity score ranges from 0 to 1 where 1 indicates the sequence frequencies are identical in the two samples and 0 indicates no shared frequencies. Arm 1: Neoadjuvant Nivo + Ipi; Arm 2: Neoadjuvant Nivo alone; Arm 3: Placebo infusion prior to surgery. Arm 3: n = 10; Arm 2: n = 20; Arm 1: n = 21.

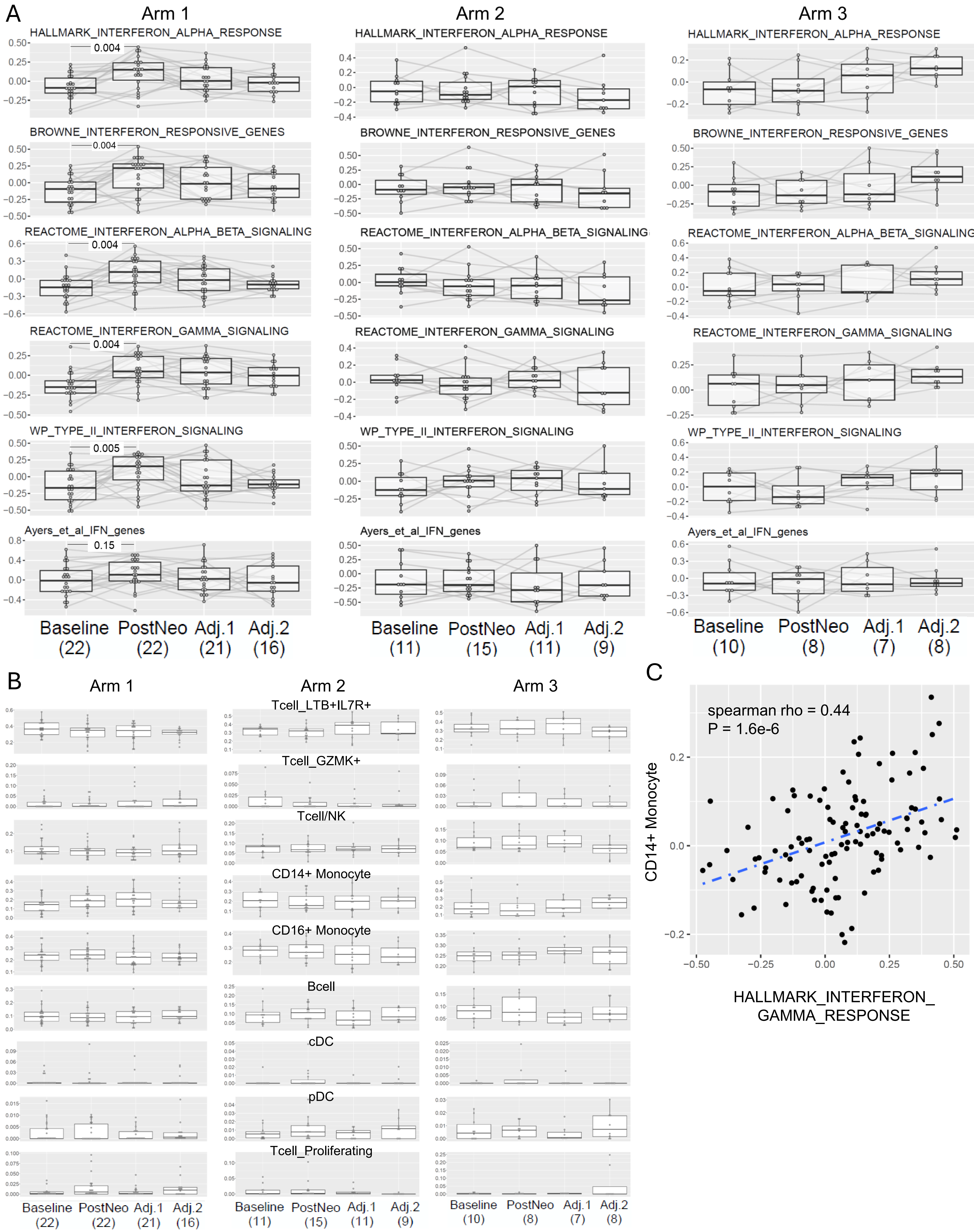
A
Arm 1
Arm 2
Arm 3
HALLMARK_INTERFERON_ALPHA_RESPONSE
0.004
BROWNE_INTERFERON_RESPONSIVE_GENES
0.004
REACTOME_INTERFERON_ALPHA_BETA_SIGNALING
0.004
REACTOME_INTERFERON_GAMMA_SIGNALING
0.004
WP_TYPE_II_INTERFERON_SIGNALING
0.005
Ayers_et_al_IFN_genes
0.15
Baseline (22) PostNeo (22) Adj.1 (21) Adj.2 (16)
Baseline (11) PostNeo (15) Adj.1 (11) Adj.2 (9)
Baseline (10) PostNeo (8) Adj.1 (7) Adj.2 (8)
B
Arm 1
Arm 2
Arm 3
Tcell_LTB+IL7R+
Tcell_GZMK+
Tcell/NK
CD14+ Monocyte
CD16+ Monocyte
Bcell
cDC
pDC
Tcell_Proliferating
Baseline (22) PostNeo (22) Adj.1 (21) Adj.2 (16)
Baseline (11) PostNeo (15) Adj.1 (11) Adj.2 (9)
Baseline (10) PostNeo (8) Adj.1 (7) Adj.2 (8)
C
spearman rho = 0.44
P = 1.6e-6
CD14+ Monocyte
HALLMARK_INTERFERON_GAMMA_RESPONSE

Supplementary Figure 7 (A) Boxplots of GSVA scores for interferon-related gene signatures across three treatment arms. Dot represent individual patients; boxes indicate the interquartile range (25th–75th percentiles); horizontal lines denote median values. P values were calculated using a two-sided Wilcoxon rank-sum test. Arm 1: Neoadjuvant Nivo + Ipi; Arm 2: Neoadjuvant Nivo alone; Arm 3: Placebo infusion prior to surgery. **(B)** Boxplots of tumor fraction as estimated by Cibersort X across three treatment arms. Dot represent individual patients; boxes indicate the interquartile range (25th–75th percentiles); horizontal lines denote median values. Arm 1: Neoadjuvant Nivo + Ipi; Arm 2: Neoadjuvant Nivo alone; Arm 3: Placebo infusion prior to surgery. **(C)** Correlation between CD14+ monocyte fraction as estimated by Cibersort X and IFN signature scores across all time point intervals of all patients (number of patients: 50; number of time intervals: 162)

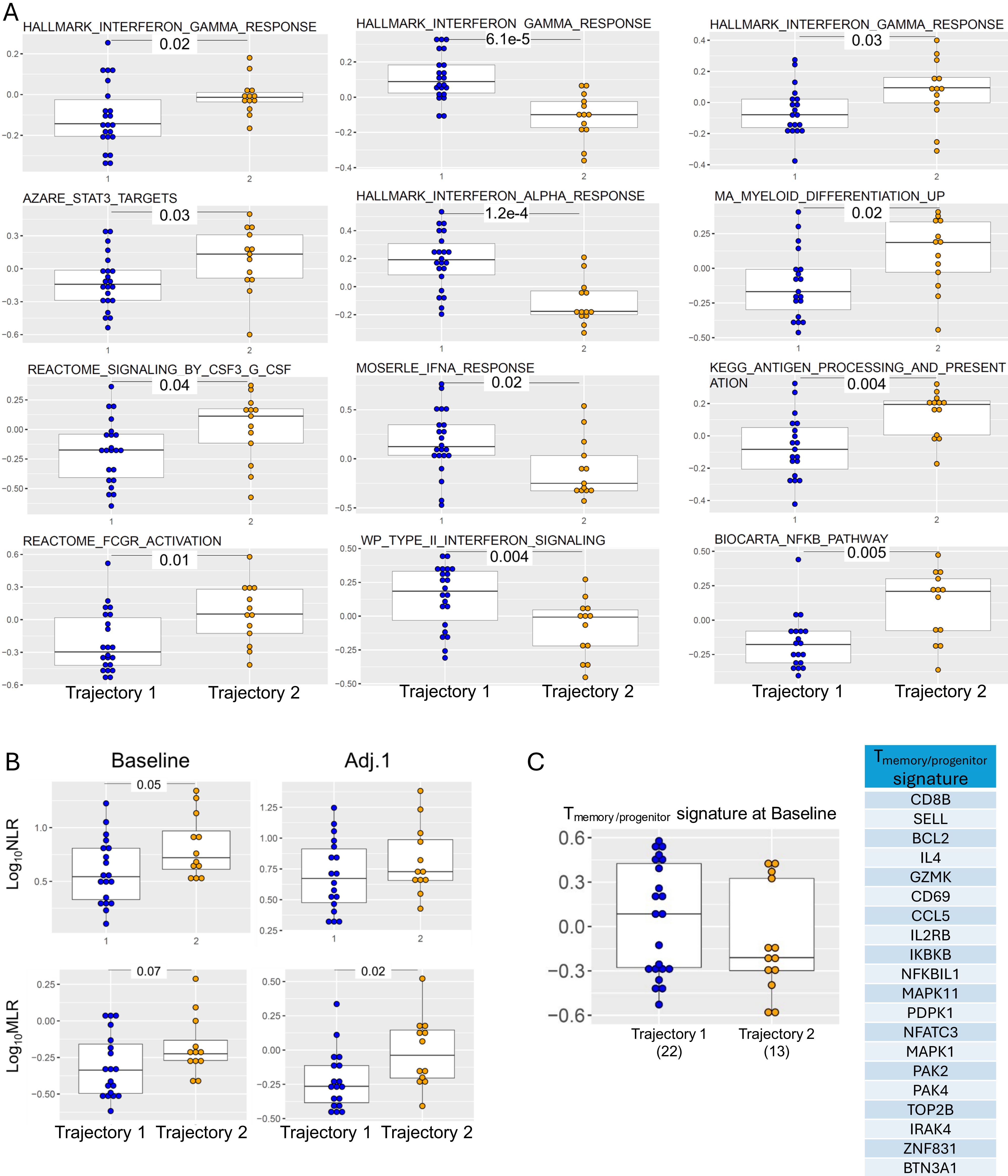

Supplementary Figure 8 (A) Boxplots showing GSVA scores for gene signatures differentially enriched between Trajectory 1 and Trajectory 2 across the three treatment arms. Sample numbers by timepoint: Baseline (Trajectory 1, n = 22; Trajectory 2, n = 13), PostNeo (Trajectory 1, n = 22; Trajectory 2, n = 13), and Adj.1 (Trajectory 1, n = 19; Trajectory 2, n = 13).

(B) Boxplots of NLR and MLR (log_{10} scale) comparing Trajectory 1 and Trajectory 2 at Baseline and Adjuvant Cycle 1. Sample numbers by timepoint: Baseline (Trajectory 1, n = 19; Trajectory 2, n = 12), and Adj.1 (Trajectory 1, n = 18; Trajectory 2, n = 12). **(C)** Boxplots of GSVA scores for the T memory/progenitor signature comparing Trajectory 1 and Trajectory 2 at Baseline, along with the list of genes comprising this signature. For boxplots, dots represent individual patients; boxes indicate the interquartile range (25th–75th percentile); horizontal lines denote median values. P-values were calculated using a two-sided Wilcoxon rank-sum test.

A

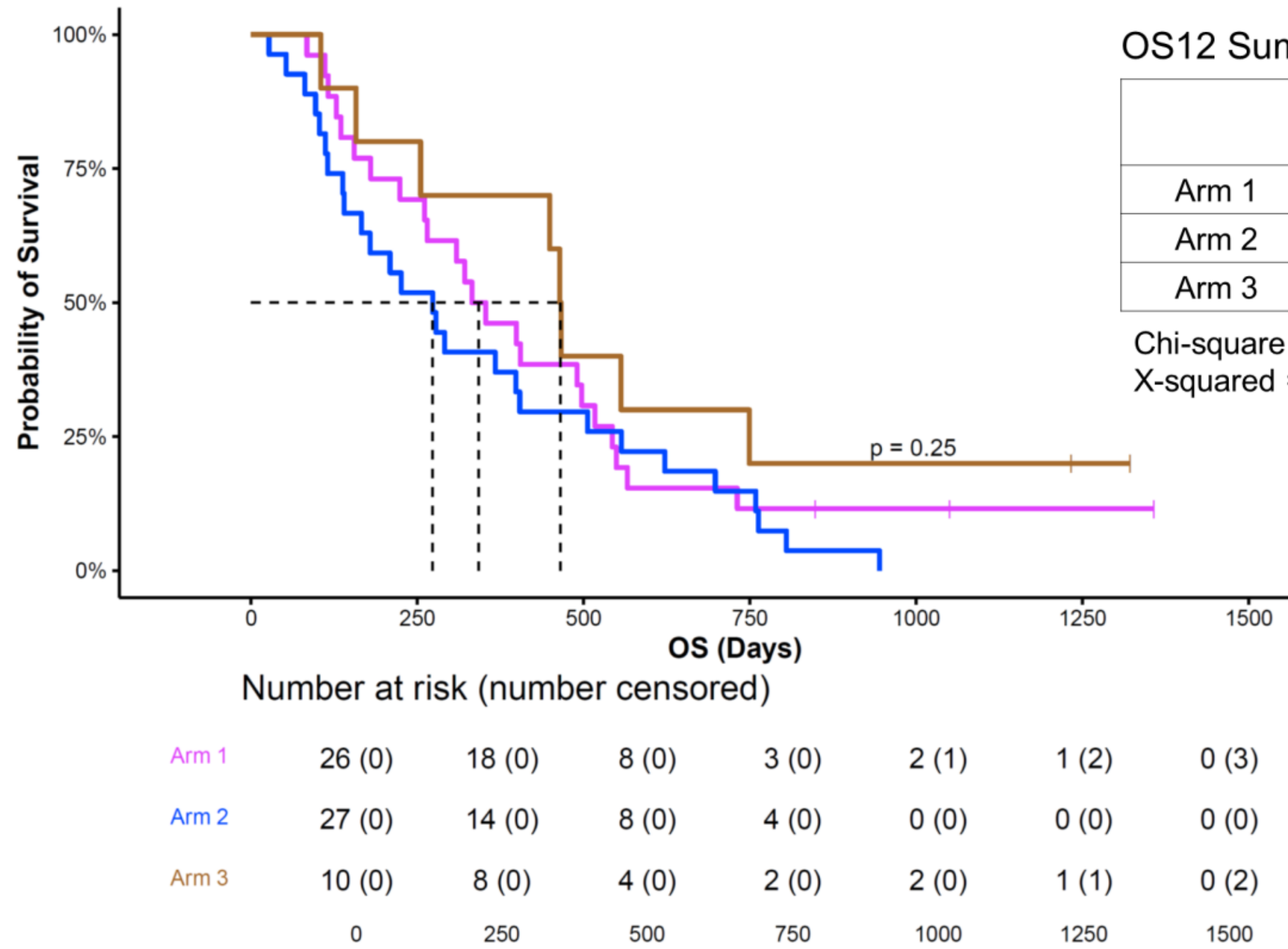

OS12 Summary

	n	Death at 12 months	Alive at 12 months
Arm 1	26	14	12
Arm 2	27	16	11
Arm 3	10	3	7

Chi-square test:
X-squared = 2.54, df = 2, p-value = 0.28

	n	events	median	95% CI
Arm = 1	26	23	342	261-543
Arm = 2	27	27	273	166-506
Arm = 3	10	8	465	255-NA

B

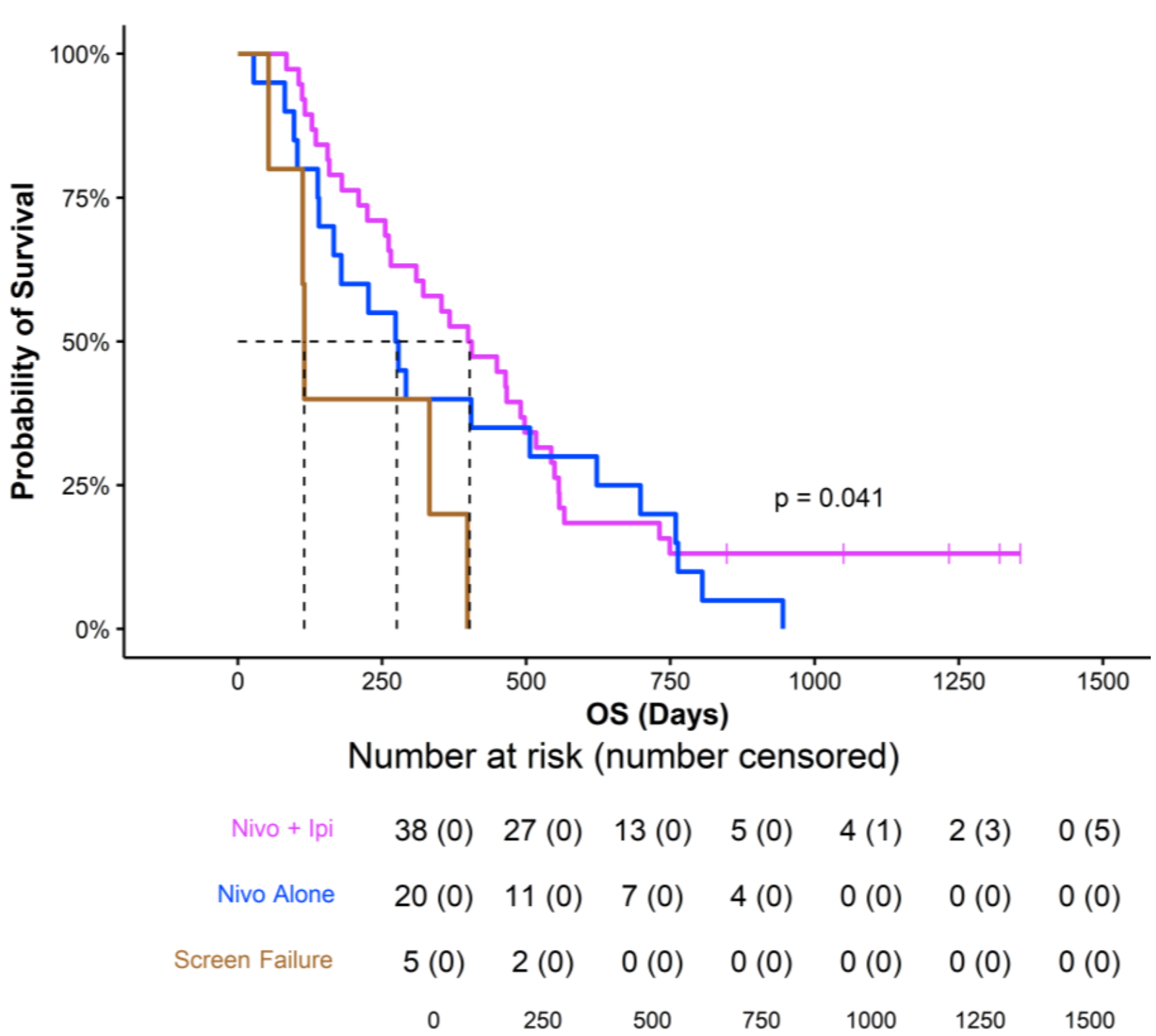

	N	Events	Median survival, days	95% CI
Nivo + Ipi	38	33	402	265–543
Nivo Alone	20	20	276	166–698
Screen Failure	5	5	115	112–NE

C

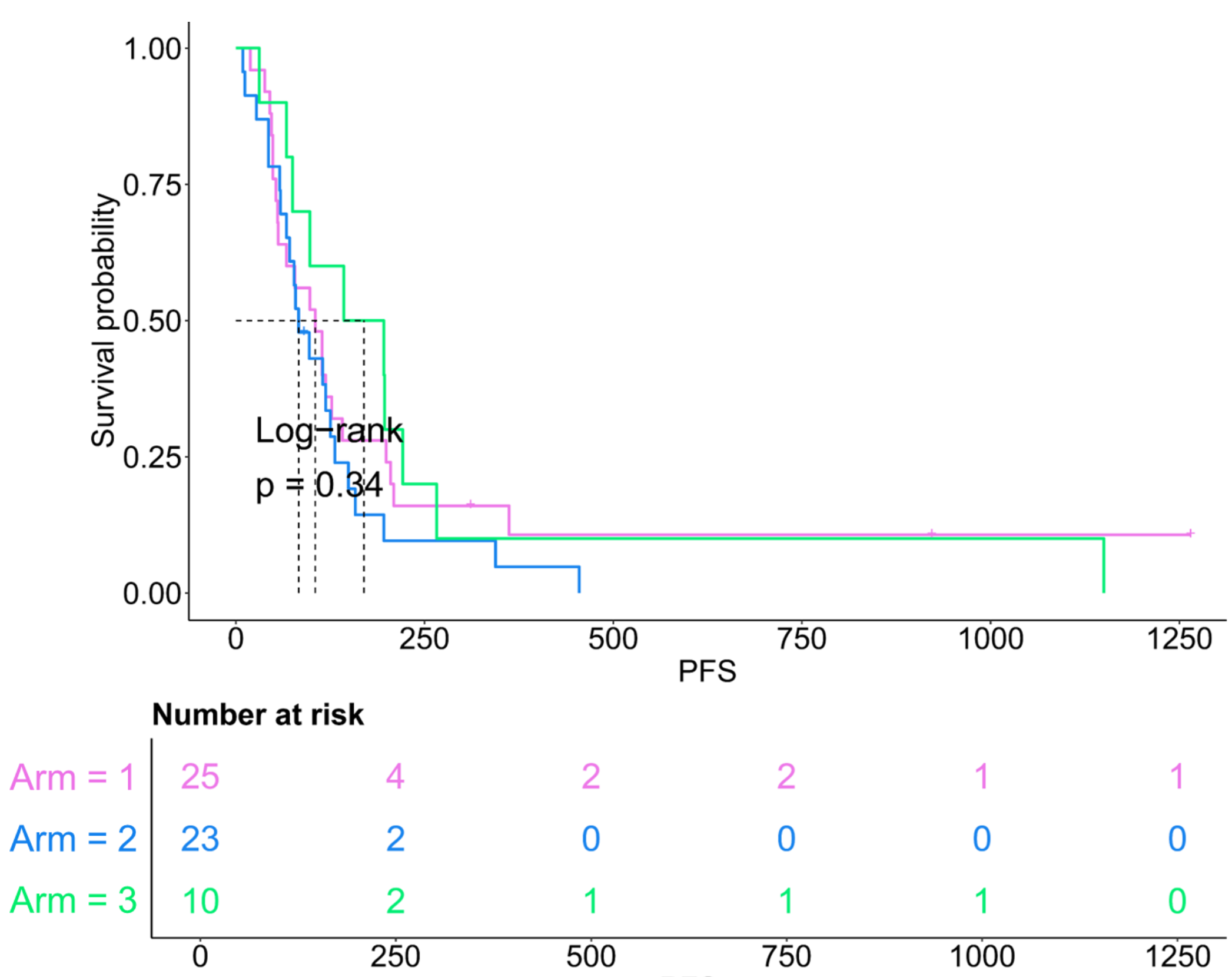

	PFS6	
	No progression at six months	Progression at six months
Arm 1	7	18
Arm 2	3	20
Arm 3	5	5

Chi-square test:
X-squared = 5.0694, df = 2, p-value = 0.07928

	n	events	median	95% CI
Arm = 1	25	22	105	56-199
Arm = 2	23	22	83	67-149
Arm = 3	10	10	170	75-NA

Supplementary Figure 9 (A) Kaplan–Meier plot comparing overall survival among the three pre-surgical treatment arms. **(B)** Kaplan–Meier plot comparing overall survival among patients who received dual ICB, single-agent PD-1 blockade, and screen failures (randomized patients who withdrew consent prior to treatment initiation). **(C)** Kaplan–Meier plot comparing progression-free survival among the three pre-surgical treatment arms. Arm 1: Neoadjuvant Nivo + Ipi; Arm 2: Neoadjuvant Nivo alone; Arm 3: Placebo infusion prior to surgery.

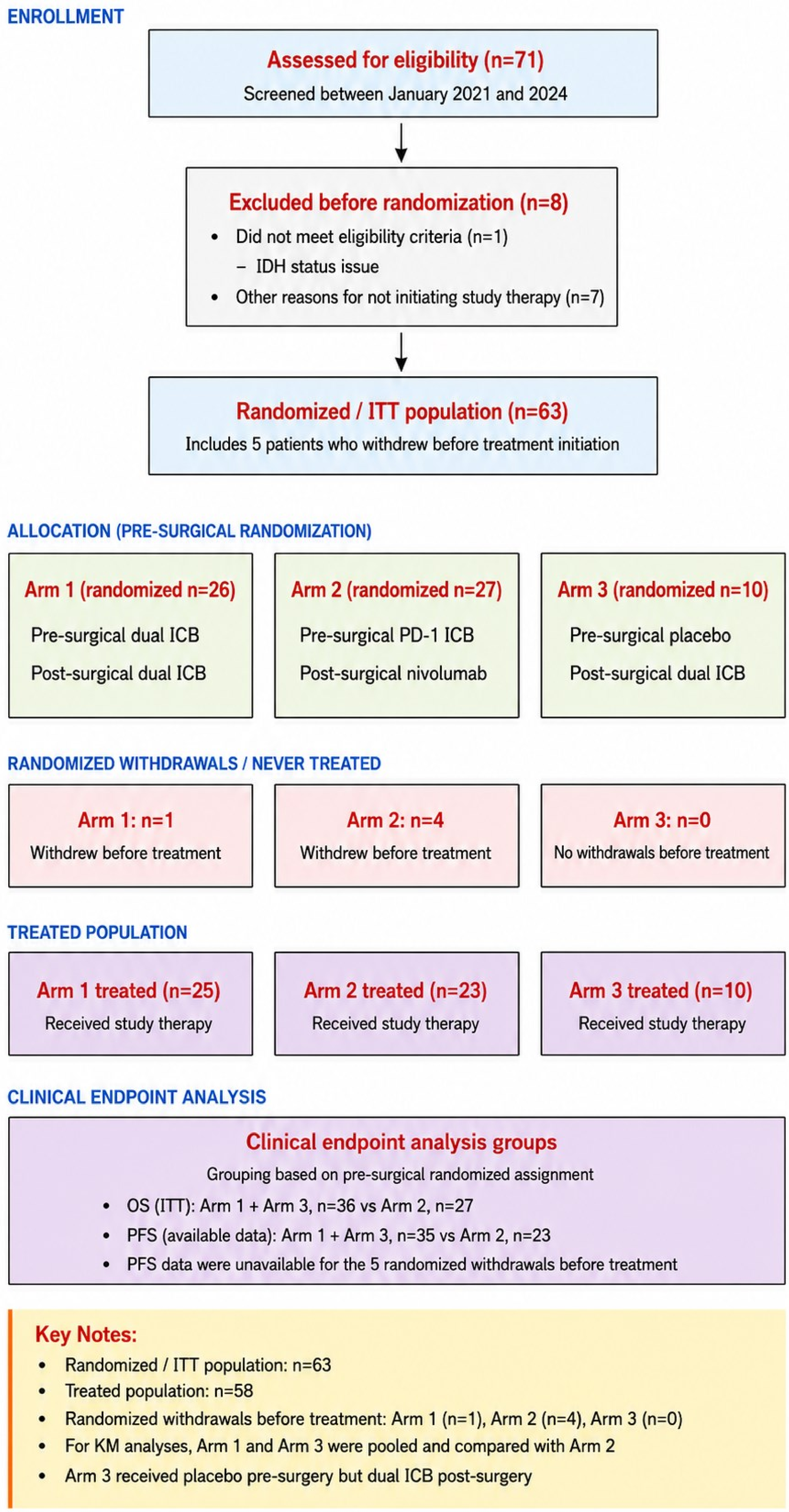

Supplementary Figure 10 CONSORT flow diagram: Dual immune checkpoint blockade in recurrent glioblastoma (NCT04606316).

Supplementary Note

A Phase Ib Clinical Trial to Evaluate Early Immunologic Pharmacodynamic Parameters Following Neoadjuvant Anti-PD-1 (Nivolumab), or the Combination of Anti-PD-1 Plus Anti-CTLA-4 (Nivolumab Plus Ipilimumab) in Patients With Surgically Accessible Glioblastoma

Protocol Version No. / Date: 5.0 / January 29, 2023

DF/HCC Protocol No.: 20-494

Bristol-Myers Squibb Protocol No.: CA209-7GR

Coordinating Center: Dana-Farber Cancer Institute

Sponsor-Investigator
Patrick Wen, MD
Dana-Farber Cancer Institute
Center for Neuro-Oncology
Boston, MA 02115-6084
Phone: 617-632-2166
Fax: 617-632-4773
patrick_wen@dfci.harvard.edu

Co-Principal Investigator
Timothy Cloughesy, MD
University of California, Los Angeles
Department of Neurology
Los Angeles, CA 90095
Phone: 310-825-5321
Fax: 310-825-0644
tcloughesy@mednet.ucla.edu

Co-Principal Investigator
David Reardon, MD
Dana-Farber Cancer Institute
Center for Neuro-Oncology
Boston, MA 02115-6084
Phone: 617-632-2166
Fax: 617-632-4773
david_reardon@dfci.harvard.edu

Co-Principal Investigator
Robert Prins, PhD
University of California, Los Angeles
Department of Neurosurgery
Los Angeles, CA 90095
Phone: 310-825-4207
Fax: 310-206-2093
rprins@mednet.ucla.edu

Co-Investigator
Howard Colman, MD
Huntsman Cancer Institute
University of Utah
Salt Lake City, UT 84112
Phone: 801-587-4042
Fax: 801-585-8613
howard.colman@hci.utah.edu

Co-Investigator
Thomas Kaley, MD
Memorial Sloan-Kettering Cancer Center
New York, NY 10065
Phone: 212-639-5122
Fax: 646-422-2147
kaleyt@mskcc.org

Co-Investigator
Isabel Arrillaga-Romany, MD
Massachusetts General Hospital
Department of Neurology
Boston, MA 02114
Phone: 617-724-8770
Fax: 617-643-8769
iarrillaga@mgh.harvard.edu

Statistician
Jiyoon Kim, MS
University of California, Los Angeles
Los Angeles, CA 90095
Phone: 310-206-1290
Fax: 310-267-2113
jiyoonkim@mednet.ucla.edu

Responsible Research Nurse: **Lisa Doherty, APRN, BC, OCN**
Dana-Farber Cancer Institute
lisa_doherty@dfci.harvard.edu

Study Agents: Nivolumab and Ipilimumab supplied by Bristol-Myers Squibb
^{89}Zr-Df-IAB22M2C supplied by ImaginAb

IND#: 151810
IND Sponsor: Patrick Wen, MD

Funding/Contractual Sponsor: Dana-Farber/Partners CancerCare, Inc.
Funding Support: Bristol-Myers Squibb

TRIAL SUMMARY

**Abbreviated Title**	Nivolumab and Ipilimumab in Surgically Accessible Recurrent Glioblastoma
**Trial Phase**	Ib
**Clinical Indication**	Surgically resectable recurrent glioblastoma
**Trial Type**	Interventional
**Type of Control**	Pre-surgery placebo control
**Route of Administration**	Intravenous
**Trial Blinding**	Neoadjuvant treatment period is blinded, and post-surgery treatment period is unblinded
**Treatment Groups**	The study will enroll bevacizumab naïve, recurrent glioblastoma patients in first or second relapse.<br>Arm 1: Pre-surgery nivolumab (3mg/kg) plus ipilimumab (1mg/kg);<br>Arm 2: Pre-surgery nivolumab (3mg/kg) plus ipilimumab-placebo;<br>Arm 3: Pre-surgery nivolumab-placebo plus ipilimumab-placebo;<br>All subjects will receive post-surgery treatment with nivolumab (3 mg/kg) q3wks in combination with ipilimumab (1mg/kg) q3wks for 9 weeks and then nivolumab (480 mg) alone q4wks. Subjects will be treated until progression, development of unacceptable toxicities, or withdrawal for a period of 2 years, but patients can continue to be treated beyond that point if they are clinically benefiting from the drug, after discussion with the Sponsor-Investigator.<br>Subjects also have the option of undergoing ^{89}Zr-Df-IAB22M2C PET scans.
**Number of Trial Subjects**	Approximately 60 eligible patients will be enrolled with 25 patients on Arm 1, 25 patients on Arm 2 and 10 patients on Arm 3.
**Estimated Duration of Trial**	The Sponsor estimates that the trial will require approximately 24 months from the time the first subject signs the informed consent until the last subject's last visit.
**Duration of Participation**	Each subject will participate in the trial from the time the subject signs the Informed Consent Form through the final protocol-specified contact. Within 14 days of screening, eligible subjects will be registered and then randomized into three arms within 5 days of registration. Subjects will receive one dose of nivolumab and ipilimumab (Arm 1), nivolumab and placebo (Arm 2), or placebo only within 14±5 days prior to surgery. After surgery subjects will receive nivolumab with ipilimumab for three cycles, then nivolumab alone. Treatment will continue until documented confirmed disease progression, unacceptable adverse event(s), intercurrent illness that prevents further administration of treatment, investigator's decision to withdraw the subject, subject withdraws consent, pregnancy of the subject, noncompliance with trial treatment or procedure requirements. After the end of treatment, each subject will be followed for 30 days for adverse event monitoring (serious adverse events will be collected for up to 100 days after the end of treatment). Subjects who discontinue for reasons other than disease progression will have post-treatment follow-up for disease status until disease progression, initiating a non-study cancer treatment, withdrawing consent, or becoming lost to follow-up. All subjects will be followed by telephone or medical record review for overall survival until death, withdrawal of consent, or the end of the study.

STUDY SCHEMA

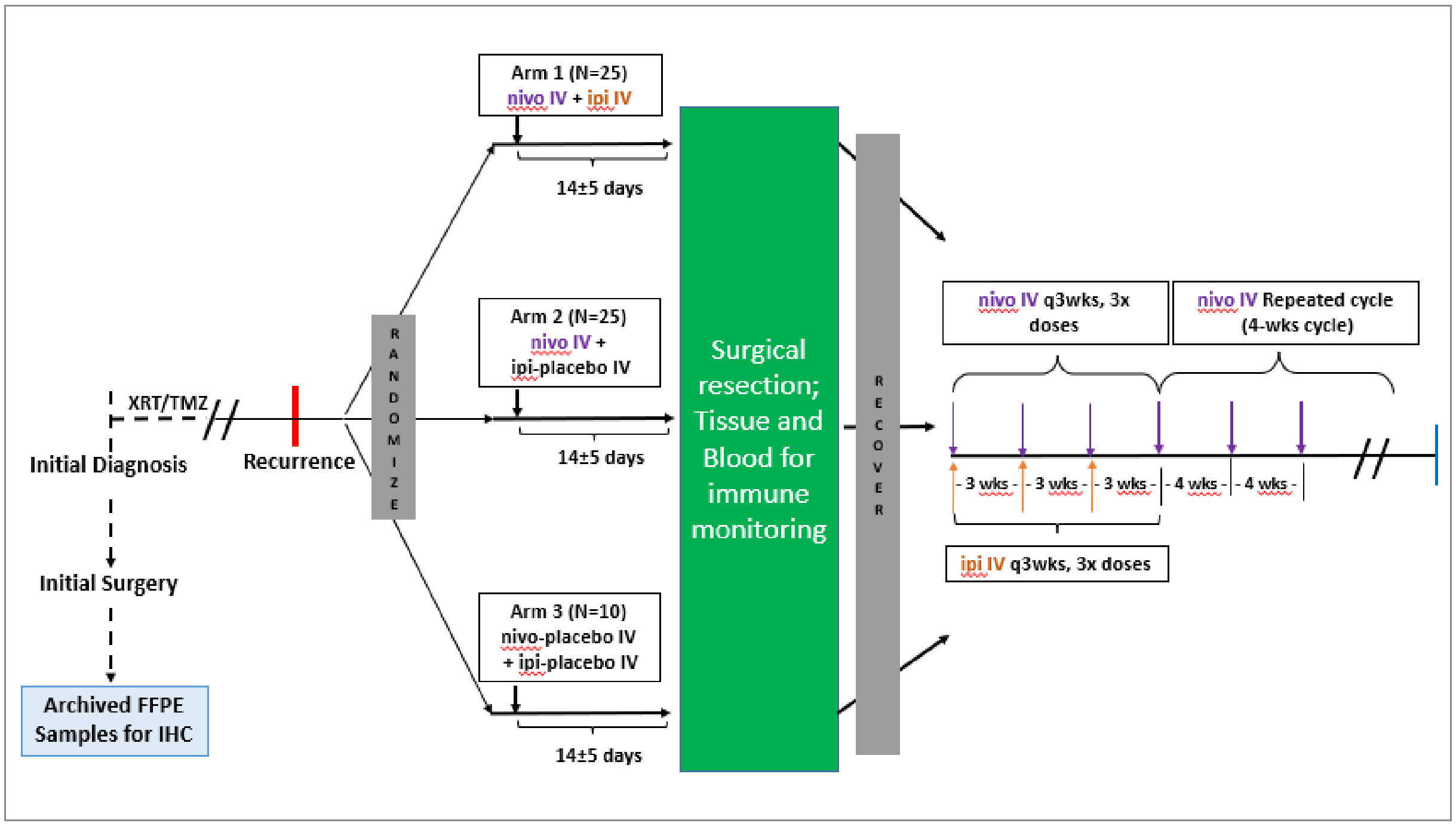

PET IMAGING SCHEMA

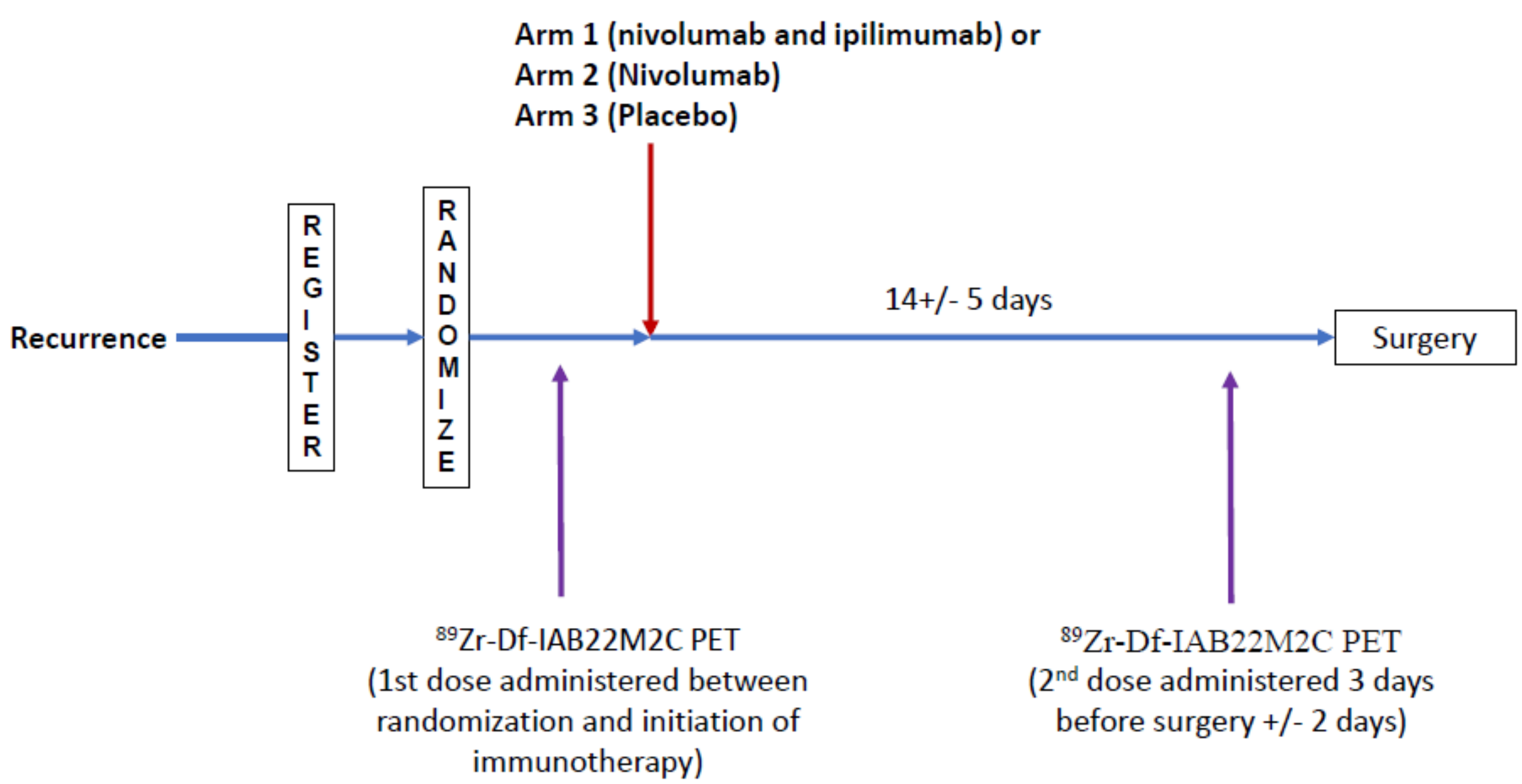

TABLE OF CONTENTS

1. OBJECTIVES 6
   1.1 Study Design 6
   1.2 Primary Objectives 7
   1.3 Secondary Objectives 7
   1.4 Exploratory Objectives 7

2. BACKGROUND 8
   2.1 Study Disease 8
   2.2 IND Agents 8
   2.3 Rationale 17
   2.4 Correlative Studies Background 21

3. PARTICIPANT SELECTION 22
   3.1 Eligibility Criteria 22
   3.2 Exclusion Criteria 25
   3.3 Inclusion of Women and Minorities 27

4. REGISTRATION AND RANDOMIZATION PROCEDURES 27
   4.1 General Guidelines for DF/HCC Institutions 27
   4.2 Registration Process for DF/HCC Institutions 27
   4.3 General Guidelines for Other Investigative Sites 27
   4.4 Registration Process for Other Investigative Sites 28

5. TREATMENT PLAN 28
   5.1 Treatment Regimen 28
   5.2 Pre-Treatment Criteria 30
   5.3 Agent Administration 30
   5.4 General Concomitant Medication and Supportive Care Guidelines 32
   5.5 Criteria for Taking a Participant Off Treatment 34
   5.6 Duration of Follow-Up 35
   5.7 Criteria for Taking a Patient Off Study 35
   5.8 Beginning and End of the Trial 36

6. DOSING DELAYS/DOSE MODIFICATIONS 36
   6.1 Toxicity Management 36
   6.2 Nivolumab 37
   6.3 Ipilimumab 41

7. ADVERSE EVENTS: LIST AND REPORTING REQUIREMENTS 44
   7.1 Expected Toxicities 45
   7.2 Adverse Event Characteristics 53
   7.3 Adverse Event Reporting 57
   7.4 Reporting to the Food and Drug Administration (FDA) 61
   7.5 Reporting to Hospital Risk Management 61
   7.6 Routine Adverse Event Reporting 61

8. PHARMACEUTICAL INFORMATION 62
8.1 Nivolumab 63
8.2 Ipilimumab 65
8.3 ^{89}Zr-Df-IAB22M2C 66

9. BIOMARKER, CORRELATIVE, AND SPECIAL STUDIES 68
9.1 Immunophenotyping 68
9.2 TIL Density and TCR Overlap 68
9.3 IHC Measurements 69
9.4 Gene Expression Signatures and Somatic Mutations 69
9.5 Sample Shipping Instructions and Details 69

10. STUDY CALENDAR 72

11. MEASUREMENT OF EFFECT 75
11.1 Antitumor Effect Definitions 75
11.2 Methods for Evaluation of Disease 75
11.3 Response Criteria 75
11.4 Evaluation of Best Response 77
11.5 Study Continuation Beyond Initial Progressive Disease 78
11.6 Central Radiology Review 80

12. DATA REPORTING / REGULATORY REQUIREMENTS 81
12.1 Data Reporting 81
12.2 Data Safety Monitoring 81
12.3 Multicenter Guidelines 82

13. STATISTICAL CONSIDERATIONS 82
13.1 Statistical Analysis Plan Summary 82
13.2 Statistical Analysis Plan 82
13.3 Stratification Factors 84
13.4 Analysis Endpoints 84
13.5 Analysis Population 84
13.6 Statistical Methods 85
13.7 Summary of Baseline Characteristics and Demographics 86
13.8 Compliance (Medication Adherence) 86
13.9 Extent of Exposure 86

14. PUBLICATION PLAN 87

REFERENCES 89

APPENDIX A KARNOFSKY PERFORMANCE STATUS 95

APPENDIX B DF/HCC MULTI-CENTER DATA AND SAFETY MONITORING PLAN 96

APPENDIX C MANAGEMENT ALGORITHMS 107

APPENDIX D REPORTABLE AE COVERSHEET 122

1. OBJECTIVES

The principle goal of this study is to understand, in surgically resectable recurrent glioblastoma, if neoadjuvant immune checkpoint therapy with nivolumab, or the combination of nivolumab plus ipilimumab elicits a systemic immunologic response, a tumoral immunologic response and toxicity and if that response can be correlated with other non-invasive measures of immune checkpoint therapy with nivolumab, or the combination of nivolumab plus ipilimumab bioactivity (tumor, blood, imaging, and clinical outcomes).

Our hypothesis is that a functional immune defect exists within the T lymphocyte compartment in glioblastoma patients. The neoadjuvant use of immune checkpoint therapy with anti-PD-1, or the combination of anti-PD-1 plus anti-CTLA-4 in this patient population will lead to a statistically significant increase in tumor infiltrating lymphocytes when compared to a concurrent control population. The combination of peripheral blood, tumor tissue and imaging evaluations should provide insight into immune activation, antitumor effect and toxicity with immune checkpoint inhibitors.

Additionally, we hypothesize that neoadjuvant immune checkpoint therapy with nivolumab, or the combination of nivolumab plus ipilimumab will be tolerable with an acceptable safety profile in the recurrent glioblastoma patient population. We also hypothesize that there will be a transcriptional increase in interferon-gamma related genes and decrease in cell cycle related genes between the neoadjuvant arms (Arms 1 and 2) versus no neoadjuvant treatment arm (Arm 3) and that a greater percentage of patients who received neoadjuvant treatment (Arm 1 or Arm 2) will not have experienced tumor progression at 6 months after treatment in comparison with a historical 6-month progression-free survival (PFS6) rate.

1.1 Study Design

This is a multicenter, randomized phase Ib trial of neoadjuvant anti-PD-1 (nivolumab), anti-CTLA-4 (ipilimumab) or the combination of anti-PD-1 plus anti-CTLA-4 (nivolumab plus ipilimumab) among bevacizumab naïve patients with surgically resectable glioblastoma at first or second relapse. Subjects will be randomized to one of three treatment arms and receive a single dose of blinded neoadjuvant therapy or placebo within 14 ± 5 days prior to surgery, as detailed in the treatment plan. The neoadjuvant period will be blinded, and the post-operative treatment period will be unblinded. Post-operatively, subjects will receive nivolumab plus ipilimumab for three doses followed by nivolumab monotherapy beginning upon adequate surgical recovery as detailed in the treatment plan. Treatment with study therapy will continue until confirmed disease progression, unacceptable toxicities, or other discontinuation criteria as described in Section 5.6.

After recovering from surgery and resuming therapy, subjects will be evaluated every 8 weeks with radiographic imaging to assess response to treatment. Response Assessment in Neuro-Oncology (RANO) criteria [1] will be used as the primary efficacy endpoint of response rate. RANO will be adapted to incorporate Immunotherapy RANO guidance [2] described in Section

11.0 due to the tumor response patterns seen with immune therapy treatment (e.g., tumor flare), and these criteria will be used by the sites for treatment decisions. Adverse events will be monitored throughout the trial and graded in severity according to the guidelines outlined in the NCI Common Terminology Criteria for Adverse Events version 4.0 (CTCAE v4.0). Safety will be assessed by quantifying the toxicities and grades experienced by subjects who have received nivolumab and the combination of nivolumab plus ipilimumab.

Subjects who discontinue treatment for reasons other than disease progression will have post-treatment follow-up of disease status until disease progression, initiation of a non-study cancer treatment, withdrawing consent, or becoming lost to follow-up. All subjects will be followed by telephone contact or medical record review for overall survival until death, withdrawal of consent or the end of the study, whichever comes first.

1.2 Primary Objectives

- To test the hypothesis that administration of neoadjuvant immune checkpoint therapy with nivolumab, or the combination of nivolumab plus ipilimumab will induce statistically significant increases in tumor infiltrating T lymphocyte (TIL) density in glioblastoma patients compared to an untreated concurrent control versus the null hypothesis of no difference between the treatment and control groups.

- To evaluate safety of study drug therapy in this patient population.

1.3 Secondary Objectives

- To evaluate the influence of the neoadjuvant administration on the cell cycle-related genetic signature within the tumor microenvironment of recurrent glioblastoma.

- To estimate therapeutic benefit of nivolumab and ipilimumab in recurrent glioblastoma patient population as measured by percent 6-month progression-free survival (PFS6) and by overall survival (OS).

1.4 Exploratory Objectives

To evaluate the associations between exploratory biomarkers and clinical outcomes and adverse events. These include:

- Estimating the correlation of quantitative assessments of TIL density or the IFN-gamma associated genetic signature with clinical responses

- Estimating correlation of quantitative assessments of TIL density or clonality with clinical responses to neoadjuvant immune checkpoint therapy in glioblastoma patients.

- Estimating efficacy of neoadjuvant immune checkpoint therapy by PFS, second PFS and OS-12 as defined by RANO and iRANO.

- Exploring effect of neoadjuvant immune checkpoint therapy on TIL proliferation (CD8+KI-67+ staining).

- Estimating difference in PD-1 and PDL-1 IHC expression between patients receiving neoadjuvant immune checkpoint therapy and those who do not as well as between archived and study samples.

- Exploring whether oligoclonal T cell populations within tumor tissue are similarly expanded in peripheral blood after neoadjuvant immune checkpoint therapy, the magnitude of which correlates with clinical responses.

- Exploring if changes in specific MRI parameters correlate with tumor and peripheral blood immune responses.
- Estimating and comparing PFS6 between randomized arms.

- Assessing the potential change in ^{89}Zr-Df-IAB22M2C uptake in tumor tissue between baseline and after neoadjuvant immune checkpoint blockade and correlation with CD8 infiltrate in tumor tissue.

- Explore the correlation of visual and semi-quantitative ^{89}Zr-Df-IAB22M2C PET measurements with clinical outcome.

2. BACKGROUND

2.1 Study Disease

Glioblastoma is the most common primary glial neoplasm of the central nervous system in adults.[3] Despite rigorous therapeutic efforts, overall prognosis remains dismal. Specifically, multimodality therapy integrating surgery, radiation therapy and temozolomide chemotherapy is associated with a median progression-free survival of only 6.9 months, and a median overall survival of 14.6 months.[3] Following progression, salvage therapies have historically been of nominal benefit with PFS-6 rates under 10% noted in recent meta-analyses.[4-6]

2.2 IND Agents

2.2.1 **Nivolumab**

Please refer to the Investigator's Brochure and/or package insert for additional detailed information on nivolumab.

2.2.1.1 Pharmaceutical and Therapeutic Background

The importance of intact immune surveillance in controlling outgrowth of neoplastic transformation has been known for decades.[7] Accumulating evidence shows a correlation between tumor-infiltrating lymphocytes (TILs) in cancer tissue and favorable prognosis in

various malignancies.[8-12] In particular, the presence of CD8+ T-cells and the ratio of CD8+ effector T-cells / FoxP3+ regulatory T-cells seems to correlate with improved prognosis and long-term survival in many solid tumors.

The PD-1 receptor-ligand interaction is a major pathway hijacked by tumors to suppress immune control. The normal function of PD-1, expressed on the cell surface of activated T-cells under healthy conditions, is to down-modulate unwanted or excessive immune responses, including autoimmune reactions. PD-1 (encoded by the gene Pdcd1) is an Ig superfamily member related to CD28 and CTLA-4 which has been shown to negatively regulate antigen receptor signaling upon engagement of its ligands (PD-L1 and/or PD-L2).[13, 14] The structure of murine PD-1 has been resolved.[15] PD-1 and family members are type I transmembrane glycoproteins containing an Ig Variable-type (V-type) domain responsible for ligand binding and a cytoplasmic tail which is responsible for the binding of signaling molecules. The cytoplasmic tail of PD-1 contains 2 tyrosine-based signaling motifs, an immunoreceptor tyrosine-based inhibition motif (ITIM) and an immunoreceptor tyrosine-based switch motif (ITSM). Following T-cell stimulation, PD-1 recruits the tyrosine phosphatases SHP-1 and SHP-2 to the ITSM motif within its cytoplasmic tail, leading to the dephosphorylation of effector molecules such as CD3ζ, PKCθ and ZAP70 which are involved in the CD3 T-cell signaling cascade.[16-19] The mechanism by which PD-1 down modulates T-cell responses is similar to, but distinct from that of CTLA-4 as both molecules regulate an overlapping set of signaling proteins.[20, 21] PD-1 was shown to be expressed on activated lymphocytes including peripheral CD4+ and CD8+ T-cells, B-cells, T regs and Natural Killer cells.[22, 23] Expression has also been shown during thymic development on CD4-CD8- (double negative) T-cells as well as subsets of macrophages and dendritic cells.[24] The ligands for PD-1 (PD-L1 and PD-L2) are constitutively expressed or can be induced in a variety of cell types, including non-hematopoietic tissues as well as in various tumors.[21, 25-27] Both ligands are type I transmembrane receptors containing both IgV- and IgC-like domains in the extracellular region and contain short cytoplasmic regions with no known signaling motifs. Binding of either PD-1 ligand to PD-1 inhibits T-cell activation triggered through the T-cell receptor. PD-L1 is expressed at low levels on various non-hematopoietic tissues, most notably on vascular endothelium, whereas PD-L2 protein is only detectably expressed on antigen-presenting cells found in lymphoid tissue or chronic inflammatory environments. PD-L2 is thought to control immune T-cell activation in lymphoid organs, whereas PD-L1 serves to dampen unwarranted T-cell function in peripheral tissues.[21] Although healthy organs express little (if any) PD-L1, a variety of cancers were demonstrated to express abundant levels of this T-cell inhibitor. PD-1 has been suggested to regulate tumor-specific T-cell expansion in subjects with melanoma (MEL).[28] This suggests that the PD-1/PD-L1 pathway plays a critical role in tumor immune evasion and should be considered as an attractive target for therapeutic intervention.

Nivolumab (previously known as BMS-936558 and MDX1106) is a potent and highly selective humanized monoclonal antibody (mAb) of the IgG4 isotype designed to directly block the interaction between PD-1 and its ligands, PD-L1 and PD-L2.

2.2.1.2 Preclinical and Clinical Trial Data

Please refer to the nivolumab Investigator's Brochure for additional preclinical and clinical data.

2.2.1.3 Ongoing Clinical Trials

Ongoing clinical trials are being conducted in advanced melanoma, non-small cell lung cancer, head and neck cancer, urothelial tract cancer, triple negative breast cancer, gastric cancer, and hematologic malignancies. For trial details please refer to the Investigator's Brochure.

2.2.1.4 Rationale for Nivolumab Dose

The nivolumab dose of 240 mg every 2 weeks (Q2W) was selected based on clinical data and modeling and simulation approaches using population PK (PPK) and exposure-response analyses of data from studies in multiple tumor types (melanoma, non-small-cell lung cancer [NSCLC], and renal cell carcinoma [RCC]) where body weight normalized dosing (mg/kg) has been used.

Population PK analyses have shown that the PK of nivolumab is linear with proportional exposure over a dose range of 0.1 to 10 mg/kg, and no differences in PK across ethnicities and tumor types were observed. Nivolumab clearance and volume of distribution were found to increase as the body weight increases, but less than the proportional with increasing weight, indicating that mg/kg dosing represents an over-adjustment for the effect of body weight on nivolumab PK. The PPK model previously developed using data from NSCLC subjects has recently been updated, using data from 1544 subjects from 7 studies investigating nivolumab in the treatment of melanoma, NSCLC, and RCC. In this dataset, the median (minimum - maximum) weight was 77 kg (35 kg - 160 kg) and thus, an approximately equivalent dose of 3 mg/kg for an 80 kg subject, nivolumab 240 mg Q2W was selected for future studies. To predict relevant summary exposures of nivolumab 240 mg Q2W, the PPK model was used to simulate virtual trials, each consisting of two arms, nivolumab 3 mg/kg Q2W and 240 mg Q2W. In the simulations, the simulated patient populations consisted of subjects randomly sampled from aforementioned pooled database of cancer patients. Because no differences in PK were noted across ethnicities and tumor types, these simulated melanoma and NSCLC data will be applicable to patients with other tumor types. The simulated measure of exposure of interest, time-averaged concentrations (Cavgss) for 240 mg Q2W are predicted to be similar for all subjects in reference to 80 kg subjects receiving 3 mg/kg Q2W.

PPK and exposure response analyses have been performed to support use of nivolumab 240 mg Q2W, 360 mg Q3W, and 480 mg Q4W dosing regimens in subjects with cancer in addition to the 3 mg/kg Q2W regimen. A flat dose of nivolumab 240 mg Q2W was selected since it is equivalent to a dose of 3 mg/kg for subjects weighing 80 kg, the observed median body weight in subjects treated with nivolumab in clinical trials, while the nivolumab 360 mg Q3W and 480 mg Q4W regimens allow flexibility of dosing with less frequent visits and in combination with other agents using alternative dosing schedules to Q2W. Using a PPK model, the overall distributions of nivolumab exposures (Cmax, Cavg, and Cmin after the first dose or at steady-state) are comparable between 3 mg/kg Q2W or 240 mg Q2W dosing regimens. Following nivolumab 360 mg Q3W and 480 mg Q4W, Cavgss are predicted to be similar to those following nivolumab 3 mg/kg Q2W or 240 mg Q2W, while Cminss are predicted to be approximately 6% and 16% lower, respectively, and are not considered to be clinically relevant. Following nivolumab 360 mg Q3W and 480 mg Q4W, maxss are predicted to be approximately ~3% and 43% higher, respectively, relative to that following nivolumab 3 mg/kg Q2W dosing. However, the range of nivolumab exposures following 240 mg Q2W, 360 mg Q3W, and 480

mg Q4W dosing regimens across body weights (35 to 160 kg) are predicted to be maintained well below corresponding exposures observed with the safe and well-tolerated 10 mg/kg nivolumab Q2W dosing regimen. Given the similarity of nivolumab PK across tumor types and the similar exposures predicted following administration of 480 mg flat-dosing every 4 weeks, 240 mg flat dosing every 2 weeks compared to 3 mg/kg every 2 weeks, it is expected that the safety and efficacy profile of nivolumab 480 mg Q4W and 240 mg Q2W will be similar to that of nivolumab 3 mg/kg Q2W. Thus, a flat dose of nivolumab 240 mg every 3 weeks in combination with ipilimumab (3 mg/kg, q3wks) for 12 weeks followed by nivolumab at 480 mg every 4 weeks and was initially used in this study. As indicated in Section 2.2.2.6 this regimen is associated with increased toxicity. As a result the protocol was amended to change the dosing to nivolumab 3mg/kg with ipilimumab 1mg/kg, q3weeks for 9 weeks, followed by nivolumab 480 mg every 4 weeks.

2.2.2 **Ipilimumab**

Please refer to the ipilimumab Investigator's Brochure and/or package insert for additional preclinical and clinical data.

2.2.2.1 Product Development Background

Ipilimumab (BMS-734016, MDX010, MDX-CTLA4) is a fully human monoclonal immunoglobulin (Ig) G1κ specific for human cytotoxic T-lymphocyte antigen 4 (CTLA-4, cluster of differentiation [CD] 152), which is expressed on a subset of activated T cells. CTLA-4 is a negative regulator of T-cell activity. Ipilimumab is a monoclonal antibody (mAb) that binds to CTLA-4 and blocks the interaction of CTLA-4 with its ligands, CD80/CD86. Blockade of CTLA-4 has been shown to augment T-cell activation and proliferation, including the activation and proliferation of tumor-infiltrating T-effector cells. Inhibition of CTLA-4 signaling can also reduce T-regulatory cell (Treg) function, which may contribute to a general increase in T-cell responsiveness, including the anti-tumor response. Yervoy™ (ipilimumab) 3 mg/kg has been approved for use in advanced melanoma in over 47 countries, including the United States (US, 25-Mar-2011), the European Union (EU, 13-Jul 2011), and Australia (Jul-2011).

Yervoy 10 mg/kg is approved as adjuvant treatment of unresectable or metastatic melanoma in the US. The European Commission (EC) decision for Yervoy Type II variation extending the indication to previously untreated melanoma patients was granted on 31-Oct-2013. Yervoy is approved in the EU for the treatment of adolescents and in the US for the treatment of melanoma in pediatric patients.

2.2.2.2 Clinical Pharmacokinetics

The PK of ipilimumab has been extensively studied in subjects with melanoma, at the ~3-mg/kg and 10-mg/kg doses administered as a 1.5-hour IV infusion. The PK of ipilimumab was characterized by population pharmacokinetic (PPK) analysis and determined to be linear and time invariant in the dose range of 0.3 to 10 mg/kg. The population pharmacokinetics of ipilimumab was studied in 785 subjects (3,200 serum concentrations) with advanced melanoma in 4 Phase 2 studies (CA184004, CA184007, CA184008, and CA184022), 1 Phase 3 study

(CA184024), and 1 Phase 1 study (CA184078). The PPK analysis demonstrated that the PK of ipilimumab is linear, the exposures are dose proportional across the tested dose range of 0.3 mg/kg to 10 mg/kg, and the model parameters are time-invariant, similar to that determined by noncompartmental analyses. Upon repeated dosing of ipilimumab, administered q3w, minimal systemic accumulation was observed by an accumulation index of 1.5-fold or less, and ipilimumab steady-state concentrations were achieved by the third dose. The ipilimumab clearance (CL) of 16.8 mL/h from PPK analysis is consistent with that determined by noncompartmental PK analysis and shown in Table 5.2.1-1. The terminal T-HALF and Vss of ipilimumab calculated from the model were 15.4 days and 7.47 L, respectively, which are consistent with that determined by noncompartmental analysis. Volume of central compartment (Vc) and peripheral compartment were reported to be 4.35 L and 3.28 L, respectively, suggesting that ipilimumab first distributes into plasma volume and, subsequently, into extracellular fluid space. CL of ipilimumab and Vc were found to increase with increase in BW. However, there was no significant increase in exposure with increase in BW when dosed on a milligram/kilogram basis, supporting dosing of ipilimumab based on a weight normalized regimen. The PK of ipilimumab is not affected by age, gender, race, and immunogenicity (ADA status); concomitant use of chemotherapy; prior therapy; BW; performance status; or tumor type. Other covariates had effects that were either not statistically significant or of minimal clinical relevance.

The population pharmacokinetics of ipilimumab when administered alone or in combination with nivolumab to subjects with melanoma, non-small cell lung cancer, renal cell carcinoma, small cell lung cancer, hepatocellular carcinoma, and colorectal cancer was also assessed. Ipilimumab CL when given in combination with nivolumab 3 mg/kg q3w was not significantly different from that seen with ipilimumab monotherapy. The CL of ipilimumab was higher when administered in combination with nivolumab 1 mg/kg q3w or 3 mg/kg q3w compared to ipilimumab monotherapy; however, the magnitude of these differences are not considered to be clinically relevant (< 20%). Thus, there is no apparent effect of nivolumab on the CL of ipilimumab. The CL of ipilimumab in subjects with NSCLC, RCC, HCC, and CRC was not significantly different relative to subjects with melanoma. The CL of ipilimumab was lower in subjects with SCLC relative to subjects with melanoma; however, the magnitude of the difference was not considered to be clinically relevant. Ipilimumab CL was not significantly different in the presence of anti-ipilimumab antibodies.

2.2.2.3 Clinical Efficacy

BMS and MDX (acquired by BMS in Sep-2009) have co-sponsored an extensive clinical development program for ipilimumab, encompassing more than 22,571 subjects (total number of subjects enrolled in ipilimumab studies) in several cancer types in completed and ongoing studies, including a compassionate use program. The focus of the clinical program is in melanoma, prostate cancer, and lung cancer, with advanced melanoma being the most comprehensively studied indication. Ipilimumab is being investigated both as monotherapy and in combination with other modalities such as chemotherapy, radiation therapy, and other immunotherapies. All studies were conducted in accordance with Good Clinical Practices, as defined by the International Conference on Harmonization and in accordance with the ethical principles underlying European Union Directive 2001/20/EC and the United States Code of Federal Regulations, Title 21, Part 50 (21CFR50).

Ipilimumab Monotherapy in Melanoma, Prostate, and Cancer

Phase 3 programs are ongoing in melanoma and lung cancer. In melanoma, 4 completed Phase 3 studies (MDX010-20, CA184024, CA184029, and CA184169) have demonstrated a clinically meaningful and statistically significant survival benefit in pretreated advanced melanoma, previously untreated advanced melanoma, and adjuvant melanoma with a manageable safety profile. Studies MDX010-20 and CA184024 were completed in subjects with advanced melanoma. Study CA184029 is complete with a final clinical study report, but still has ongoing patients in follow-up.

Ipilimumab Monotherapy in Metastatic Castrate Resistant Prostate Cancer (mCRPC)

The completed Phase 3 study (CA184043) evaluated ipilimumab in subjects with mCRPC who had progressed during or following treatment with docetaxel. Eligible subjects were randomized to a single dose of bone-directed RT, followed by either ipilimumab 10 mg/kg or placebo (799 randomized: 399 ipilimumab and 400 placebo). This study did not meet its primary endpoint of OS. The safety profile in this study was consistent with the previously defined adverse event (AE) profile at the same dose. A second Phase 3 study (CA184095) evaluated ipilimumab 10 mg/kg versus placebo in subjects with asymptomatic or minimally symptomatic, chemotherapy-naïve mCRPC with no visceral metastases. The study did not meet its primary endpoint of demonstrating a statistically significant prolongation of survival for the ipilimumab group (10 mg/kg) compared with the placebo group. The safety profile of ipilimumab in this study was generally consistent with the experience in other studies using 10 mg/kg ipilimumab. No new safety signals were identified in this patient population.

Ipilimumab in Monotherapy and Combination Therapy in Lung Cancer

Activity was observed in a large Phase 2 study in lung cancer (NSCLC and SCLC; Study CA18404155) in combination with chemotherapy. Two Phase 3 studies evaluated ipilimumab in combination with chemotherapy in squamous NSCLC (CA184104) and SCLC (CA184156). Neither study met its primary endpoint of demonstrating a statistically significant prolongation of OS for the ipilimumab group (10 mg/kg ipilimumab/standard of care chemotherapy) over the placebo group (placebo/standard of care chemotherapy) among randomized subjects who received at least 1 dose of blinded study therapy; however, no new safety concerns were identified in the course of standard clinical safety monitoring of the 2 studies. While the types of safety events observed in subjects receiving ipilimumab do not appear to change, even in combination with other anti-cancer agents, the proportion of subjects experiencing 1 type or another immune related adverse events (irAE) may be impacted by the choice of combination partner. Skin and GI irAEs predominate in monotherapy studies. In combination with DTIC (melanoma), the incidence of skin and GI irAEs was lower than expected and the incidence of hepatic irAEs was higher. In combination with paclitaxel and carboplatin (NSCLC), the incidence of all types of irAEs appeared to be numerically lower compared to the incidence observed for ipilimumab monotherapy in the Phase 2 program. In a Phase 1 study (CA184161), the concomitant administration of vemurafenib and ipilimumab in subjects with BRAF V600 mutated metastatic melanoma resulted in asymptomatic and reversible increases in aspartate aminotransferase (AST) and alanine aminotransferase (ALT), exceeding the incidence to be expected when either agent is administered as a monotherapy, leading to discontinuation of this treatment. All were reversible with dose reduction, interruption, or permanent discontinuation

of the study drugs or administration of corticosteroids. In a Phase 2 study (CA184240), sequential treatment with vemurafenib followed by 10 mg/kg ipilimumab in subjects with BRAF V600-mutated metastatic melanoma was tolerable with a manageable safety profile. No significant BRAF V600 signals of hepatobiliary toxicity were reported. The benefit/risk profile of this sequence needs to be evaluated further based on individual subject characteristics and new treatment options. Ipilimumab is also being evaluated in clinical studies conducted independently by the Cancer Therapy Evaluation Program of the US NCI, as well as in several additional externally sponsored studies.

The PK of ipilimumab in combination with nivolumab was well described by a linear 2-compartment model with time-varying CL. Ipilimumab CL decreased with time and the decrease was ~22% in subjects receiving ipilimumab in combination with nivolumab. The CL of ipilimumab was higher when administered in combination with nivolumab 1 mg/kg q2w, 1 mg/kg q3w, or 3 mg/kg q2w compared to ipilimumab monotherapy; however, the magnitude of these differences are not considered to be clinically relevant (< 20%). Ipilimumab CL when given in combination with nivolumab 0.3 mg/kg or 3 mg/kg q3w was not significantly different from that seen with ipilimumab monotherapy.

2.2.2.4 Clinical Safety

Ipilimumab is associated with inflammatory events resulting from increased or excessive immune activity, likely to be related to its mechanism of action. Inflammatory events were analyzed in 2 ways:

<u>Immune-related Adverse Events (irAEs)</u>

irAEs are defined as AEs that are 1) related to ipilimumab per investigator and 2) consistent with an inflammatory process. The most common treatment-related AEs associated with the use of ipilimumab were immune related (named immune-related AEs or irAEs), reflecting ipilimumab’s mechanism of action. The irAEs primarily involved the gastrointestinal (GI) tract (eg, diarrhea and colitis); skin (eg, pruritus and rash); and, less frequently, the liver (eg, transaminase elevations), endocrine glands (including the thyroid, pituitary and adrenal glands, manifested by hypothyroidism, hypophysitis with hypopituitarism, or adrenal insufficiency, respectively), and nervous system (e.g. motor neuropathy with or without sensory neuropathy). Immune-related AEs were generally manageable using symptomatic or immuno-suppressive therapy as recommended through detailed diagnosis and management guidelines, as described in Section 7.

<u>Immune-mediated Adverse Reactions (imARs)</u>

imARs represent the subset of events for which a nonimmune etiology could not be established and included the following specific events of clinical interest: enterocolitis, dermatitis, hepatitis, neuropathies, endocrinopathies, and other. The definition of imARs and the methodology for imAR analysis were developed jointly by BMS and the US Food and Drug Administration for the first approval of 3 mg/kg ipilimumab monotherapy in the US.

Since the imAR definition was developed after completion of the original registrational studies, imARs were originally retrospectively adjudicated by the Sponsor in a blinded fashion to investigator attribution (including for Studies MDX010-20 and CA184024). In more recent studies, imARs were adjudicated by the investigator based on the same case definitions.

Importantly, case definitions for imARs should not be used as guidance for AE management, as events are assessed retrospectively, and adjudication includes consideration of the eventual outcome of the event.

2.2.2.5 Rationale for Ipilimumab Dose

The ipilimumab dosing schedule for this study was 3 mg/kg administered intravenously over 90 minutes every 3 weeks for a total of 4 doses including one neoadjuvant dose and up to 3 post-operative doses. The rationale for this dosing schedule is based on the FDA approval of ipilimumab for unresectable or metastatic melanoma in adult and pediatric patients. However, given the increased toxicity of nivolumab 240 mg and ipilimumab 3 mg/kg every 3 weeks, as indicated in Section 2.2.2.6, the dosing schedule has been revised to nivolumab 3mg/kg and ipilimumab 1mg/kg every due to the improved tolerability and equivalent efficacy of this regimen.[29]

2.2.2.6 Rationale For Amended Dose

The initial dose in this trial of ipilimumab 3mg/kg and nivolumab 240mg every 3 weeks was based on the original regulatory approval of the combination. However, in the first 7 patients one came off treatment for toxicity, and 3 discontinued treatment because of poor quality of life. An alternate regimen using a lower dose of ipilimumab of 1 mg/kg and nivolumab 3 mg/kg every 3 weeks has been found to be better tolerated with equivalent efficacy [29]. The protocol has been amended to incorporate the lower dose regimen for a total of up to 4 doses including one neoadjuvant dose and up to 3 post-operative doses if in Arm A or 3 post-operative doses if in Arms B and C, followed by nivolumab 480mg every 4 weeks.

2.2.2.7 Rational For Amended Schedule

Recent data from the validation cohort from DFCI protocol 16-225 failed to confirm the previous data indicating that neoadjuvant checkpoint blockade improves survival. Given these results suggesting that neoadjuvant and adjuvant nivolumab may not improve outcome the protocol has been amended to allow all patients to receive the combination of ipilimumab and nivolumab following surgery.

2.2.3 **^{89}Zr-Df-IAB22M2C**

Please refer to the ^{89}Zr-Df-IAB22M2C Investigator's Brochure for additional preclinical and clinical data.

2.2.3.1 Although immunotherapies that recruit and enhance effector function of tumor infiltrating T cells, including immune checkpoint inhibitors, have revolutionized the treatment of multiple cancer indications, only a subset of treated participants respond. One important predictor of benefit from such immunotherapeutic approaches is the presence and change in tumor infiltrating CD8+ T cells. Identifying the presence and changes in CD8+ TILs can be challenging, particularly for participants with CNS tumors, and usually requires invasive procedures that include repeat tissue biopsy which may not be representative of the immune

status of the entire tumor. The ability to visualize CD8+ T cells in vivo before and after treatment would result in a major advancement in the understanding of local and systemic immune response in immuno-oncology.

ImaginAb has developed ^{89}Zr-Df-IAB22M2C, an ~80 kDa minibody (Mb) with high affinity (binding EC50 = 0.4nM) for the CD8 glycoprotein that is conjugated with desferrioxamine (Df) and radiolabeled with ^{89}Zr for imaging CD8+ T cells in humans. The Mb fragment was designed to address the shortcomings of full-length monoclonal antibodies when used for molecular imaging. Intact monoclonal antibodies have prolonged blood circulation time that result in a significant background signal after administration and require several days before high contrast images can be obtained. Furthermore, the Fc region of monoclonal antibodies can lead to activation of immune cell effector functions, which is undesirable in a diagnostic imaging setting. The Mb format circumvents these issues as it is a bivalent homodimer with each monomer consisting of a single-chain variable fragment (scFv) linked to the human IgG1 CH3 domain. The lack of Fc receptor interaction domains on the Mb platform yields a molecule that is pharmacologically inert with respect to Fcγ receptor mediated effector functions. The smaller size and inability of the Mb to interact with the FcγR and the neonatal Fc recycling receptor results in an inert imaging agent with rapid clearance. IAB22M2C was engineered from the humanized heavy and light chain sequences of murine OKT8 antibody that targets human CD8.

CD8 is expressed exclusively on hematopoietic cells including cytotoxic T-lymphocytes (CTLs), some natural killer (NK) and natural killer T cells (NKT) and on a subset of dendritic cells. The CD8 T cell marker is a cell-surface glycoprotein complex that serves as a co-receptor for major histocompatibility complex class I (MHC-I)-restricted recognition of antigens by the T-cell receptor (TCR).[30]

The binding affinity and specificity of IAB22M2C and Df-IAB22M2C have been established using flow cytometry with CD8 expressing purified human T cells, HPB-ALL leukemia cells (DSMZ, Braunschweig Germany) and PC3 prostate cancer cells engineered to express huCD8 (ImaginAb, Inc.). IAB22M2C does not show significant cross-reactivity to the non-human primate ortholog of CD8 as demonstrated by lack of binding to fresh PBMCs obtained from cynomolgus monkeys. Similar results demonstrating lack of cross-reactivity were obtained with rhesus monkey PBMCs.

The pharmacology of Df-IAB22M2C was evaluated using in vitro assays and in vivo models. No measurable effects of the conjugated Mb, Df-IAB22M2C on proliferation or activation of T cells was detected using a panel of functional in vitro assays. In vivo studies conducted in Hu-CD34 NSG™ mice also showed no acute effects of Df-IAB22M2C on T cell populations or cytokine release after intravenous administration. In contrast, IAB22FL, the full length hIgG1 protein comprised of the same variable sequences that binds to the same epitope as IAB22M2C, depleted CD8 T cells in Hu-NSG™ mice engrafted with human CD34 stem cells. The inert pharmacology of Df-IAB22M2C compared with Df-IAB22FL can be attributed to the removal of the CH2 domain and corresponding loss of effector functions that are associated with intact IgG1s. Neither protein mediated any detectable cytokine release in these animals. Preclinical

imaging and biodistribution studies have shown favorable pharmacokinetics and demonstrated the ability of ^{89}Zr-Df-IAB22M2C to detect infiltrating CD8 T cells in a Hu-PBMC NSG™ model of GvHD. To date, neither IAB22M2C nor ^{89}Zr-Df-IAB22M2C have mediated any CD8-specific adverse pharmacological effects. When considered together, these results demonstrate that ^{89}Zr-Df-IAB22M2C is a promising agent for immunoPET imaging of CD8+ T cells in response to immunotherapies for treating different cancers.

To date, two studies have evaluated ^{89}Zr-Df-IAB22M2C in human cancer participants. NCT03107663 was a 2-stage phase 1 study that evaluated ^{89}Zr-Df-IAB22M2C among 15 participants with advanced solid tumors.[31] Participants received approximately 111 MBq (3 mCi) of ^{89}Zr-Df-IAB22M2C at minibody doses of 0.2 to 10 mg. Biodistribution was dependent on the minibody mass administered with prominent activity noted in spleen, bone marrow, normal lymph nodes and tumor as early as 2 hours after injection with most uptake detected within 24 hours. All tissue activities were relatively similar between the expanded cohorts at the 0.5 mg to 1.5 mg doses. Of note, there were no immediate or delayed side effects attributed to ^{89}Zr-Df-IAB22M2C. This study demonstrated that ^{89}Zr-Df-IAB22M2C was safe, had favorable kinetics for early imaging and could successfully target CD8+ T cell-rich tissues. The recommended phase 1 dose defined was 3 mCi of ^{89}Zr-Df-IAB22M2C linked with 1.5 mg of the minibody. NCT03802123 is an ongoing phase 2 study evaluating ^{89}Zr-Df-IAB22M2C among up to 47 advanced solid tumor participants undergoing standard therapy. ^{89}Zr-Df-IAB22M2C is administered at a dose of up to 3 mCi (+/- 20%) conjugated to 1.5 mg of minibody. In addition to safety, the primary endpoint of this study is analysis of ^{89}Zr-Df-IAB22M2C uptake as determined by SUV-based quantitative measures relative to CD8+ TIL density determined by IHC from tumor biopsy samples.

2.3 Rationale

2.3.1 Rationale for the Trial and Selected Subject Population

More effective treatments are needed for glioblastoma. Glioblastoma is one of the most lethal of human cancers, with very few long-term survivors and no definitive cures for this disease. These tumors invade and infiltrate the surrounding brain, making complete surgical excision impossible. They are also among the most radiation and chemotherapy resistant cancers, with a median survival of 12-18 months from initial diagnosis, even with surgery, radiation and chemotherapy [3, 32]. The glioblastoma patient population has dismal outcomes and innovative

approaches are desperately needed.

Immunotherapy is ideally suited to target isolated pockets of infiltrating tumor cells in the brain. There has been a long-standing interest in applying tumor immunotherapy approaches to primary brain tumors. Unfortunately, these efforts have largely shown little in the way of objective therapeutic efficacy.[33]

Figure 1: **PD-1 expression on T cells from glioblastoma patients**

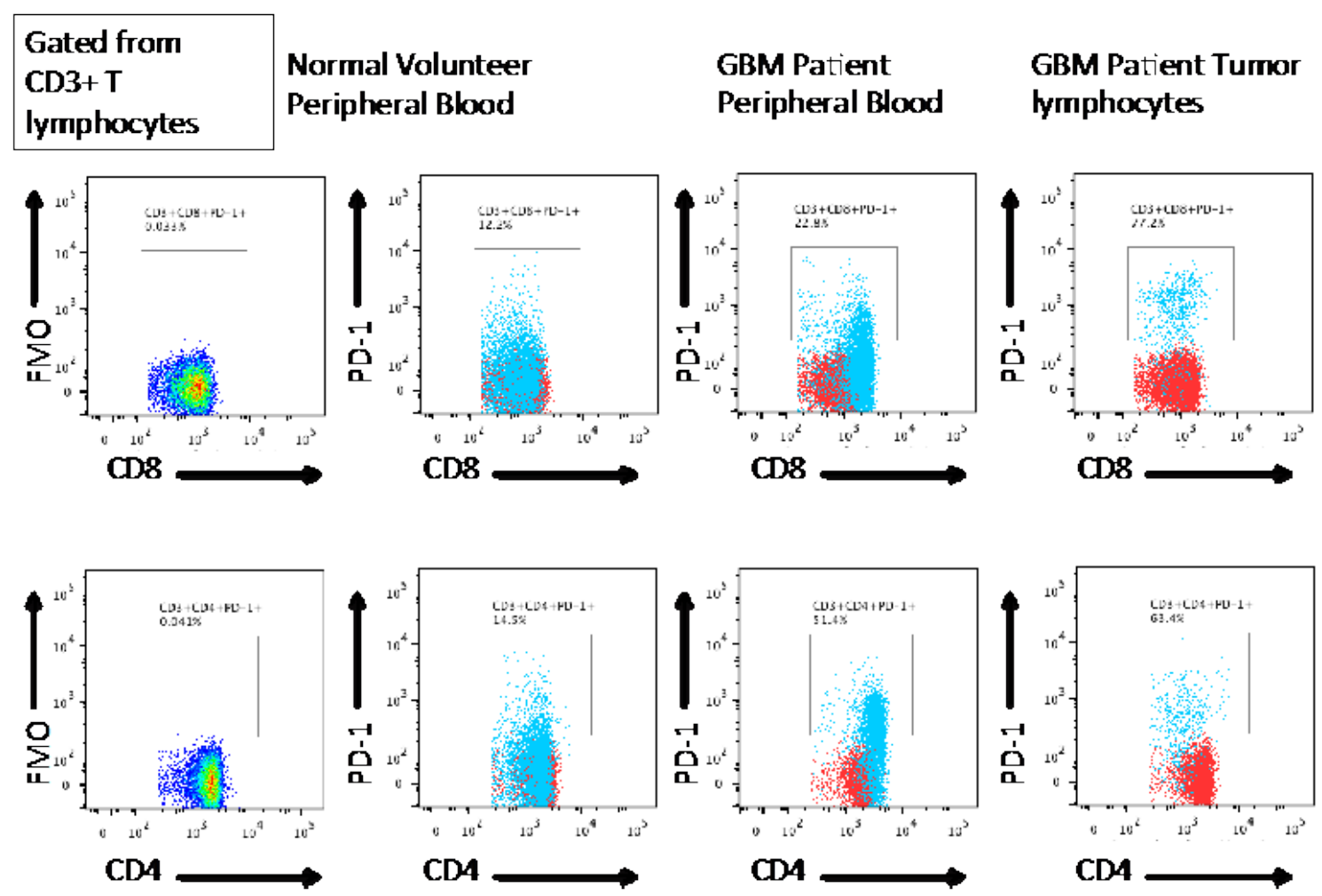

Part of the difficulty has been: 1) the "immune privilege" of the central nervous system (CNS), 2) the limited trafficking of immune cells into the CNS, and 3) the lack of well-defined, highly immunogenic tumor-rejection antigens (TRA) in gliomas. Our recent pre-clinical studies have demonstrated that we can potentially overcome some of these critical barriers to progress in brain tumor immunotherapy. In a syngeneic, orthotopic glioma mouse model, we demonstrated that PD-1 mAb significantly increased the infiltration of T lymphocytes into murine GL261 gliomas. In addition, peripheral blood and tumor infiltrating lymphocyte staining of samples from glioblastoma patients demonstrated elevated PD-1 expression by $CD4^+$ and $CD8^+$ T cells compared with normal donor patients, strongly suggestive of local, functional T defects (Figure 1) [34, 35]. PD-1/PD-L1 IHC staining has also definitively revealed clear expression of PD-1 on lymphocytic infiltrates and dynamically upregulated expression of PD-L1 on macrophages and tumor cells within recurrent glioblastoma tissue (Figure 2)[36]. Thus, our studies document a discrete target for therapeutic intervention in patients with a deadly disease, with pre-clinical evidence of mechanism and therapeutic benefit. Advances in tumor immunotherapy may be of tremendous importance and relevance to primary brain tumors, particularly if these

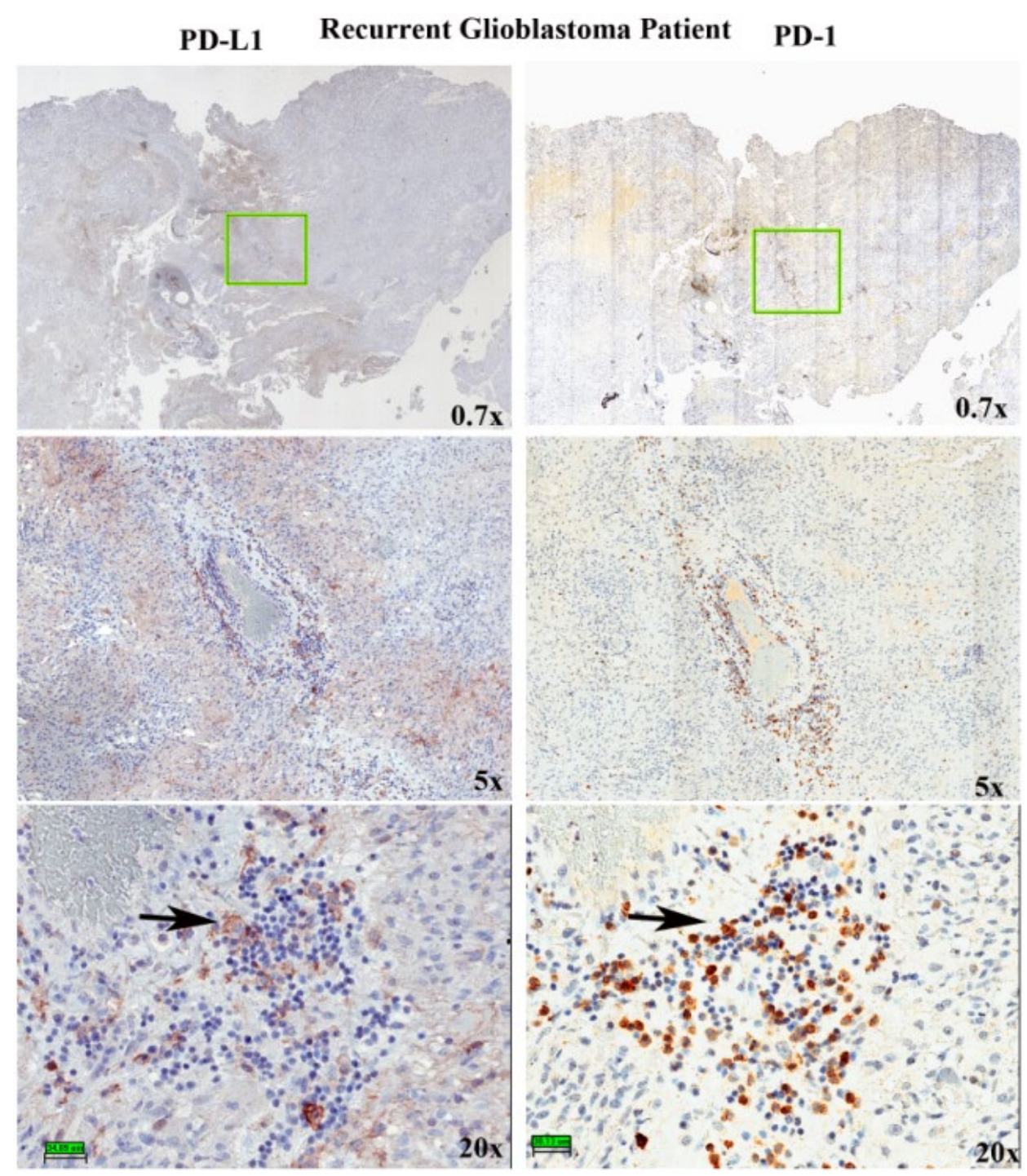

Figure 2: Immunohistochemical staining for PD-1 and PD-L1 in recurrent glioblastoma. Representative PD-1 and PD-L1 IHC staining of a recurrent glioblastoma patient. Staining was performed at Merck Laboratories (FlexTRS97). Note the clear lymphocytic staining of PD-1 and adjacent cellular localization of PD-L1.

modulators of immunity have the capability of tracking and destroying infiltrating tumor cells.

Tissue based biomarker design: Based on our pre-clinical findings in glioblastoma, as well as the findings of our colleagues at UCLA studying anti-PD-1 therapy in melanoma patients, we believe that a series of novel tumor tissue-based assays will represent valid pharmacodynamic biomarkers for the bioactivity of this new agent in tumor tissue of glioblastoma patients for this proposed clinical trial. “Window of opportunity” and/or “neoadjuvant” therapy trials allow for the assessment of investigational agents upon the local tumor microenvironment in surgically resectable cancers [37, 38]. As such, we propose a series of tissue and peripheral blood-based biomarkers in glioblastoma patients treated with neoadjuvant immunotherapy. A next generation sequencing and digital droplet PCR -PCR-based assay will quantitatively assess the density of T lymphocytes within tumor tissue and the T cell receptor (TCR) clonality [39]. Data from UCLA strongly suggests that TIL density and TCR clonality can be reliably quantified from tumor tissue and are correlated with clinical responses to anti-PD-1 therapy in melanoma patients and more recently in glioblastoma [36, 40] . Such data also suggest that not only is the number of T lymphocytes important, but also the diversity for the response to anti-PD-1 therapy. These data are insightful as objective responses are known to occur with PD-1 inhibition in melanoma, however, it is unclear if any clinical or, for that matter, tissue biomarker responses might occur with PD-1 in the setting of recurrent/progressive glioblastoma. Similarly, multiplex IF has recently been shown to be able to accurately quantify immune cell infiltration in situ, and evaluate the co-expression of 6-8 distinct markers [41-45]. Finally, transcriptional signatures of treated tumor tissue have been recently shown to predict immunologic and clinical responses to checkpoint blockade in cancer patients [36, 46].

Neoadjuvant Trials of PD-1 Antibody Blockade in Recurrent Glioblastoma: Recently we published a surgical trial of neoadjuvant pembrolizumab, a PD1 antibody, to address the immunologic effects of this agent in recurrent glioblastoma. In this trial 30 evaluable patients with recurrent glioblastoma requiring reoperation were randomized to receive pembrolizumab (200 mg) or no treatment prior to surgery [36]. 10-12 days later patients underwent reoperation. Following recover from surgery patients received pembrolizumab (200mg every 3 weeks) until progression or development of unacceptable toxicity. Patients who were randomized to receive neoadjuvant pembrolizumab, with continued adjuvant therapy following surgery, had significantly extended overall survival compared to patients that were randomized to receive adjuvant, post-surgical programmed cell death protein 1 (PD-1) blockade alone. The median overall survival of the neoadjuvant treatment cohort was 417 d (13.7 months) from registration date, while that of the adjuvant treatment cohort was 228 d (7.5 months; hazard ratio 0.39, 95% confidence interval 0.17–0.94; $P = 0.04$, log-rank test; **Figure 3**). Neoadjuvant PD-1 blockade was associated with upregulation of T cell– and interferon-γ-related gene expression, but downregulation of cell-cycle-related gene expression within the tumor, which was not seen in patients that received adjuvant therapy alone (**Figure 4**). Focal induction of PDL1 in the tumor microenvironment, enhanced clonal expansion of T cells, decreased PD-1 expression on peripheral blood T cells and a decreasing monocytic population was observed more frequently in the neoadjuvant group than in patients treated only in the adjuvant setting. These findings suggest that the neoadjuvant administration of PD-1 blockade enhances both the local and systemic antitumor immune response and may represent a more efficacious approach to the

treatment of this uniformly lethal brain tumor.

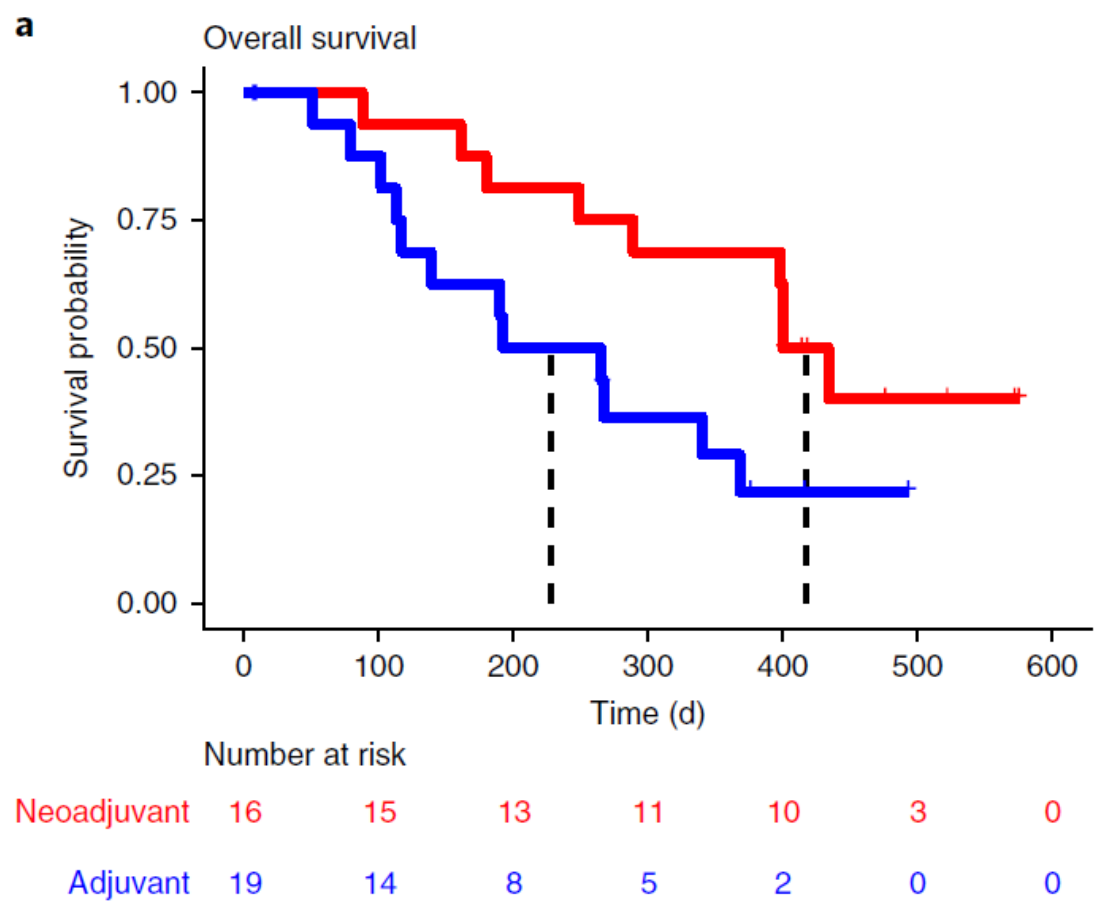

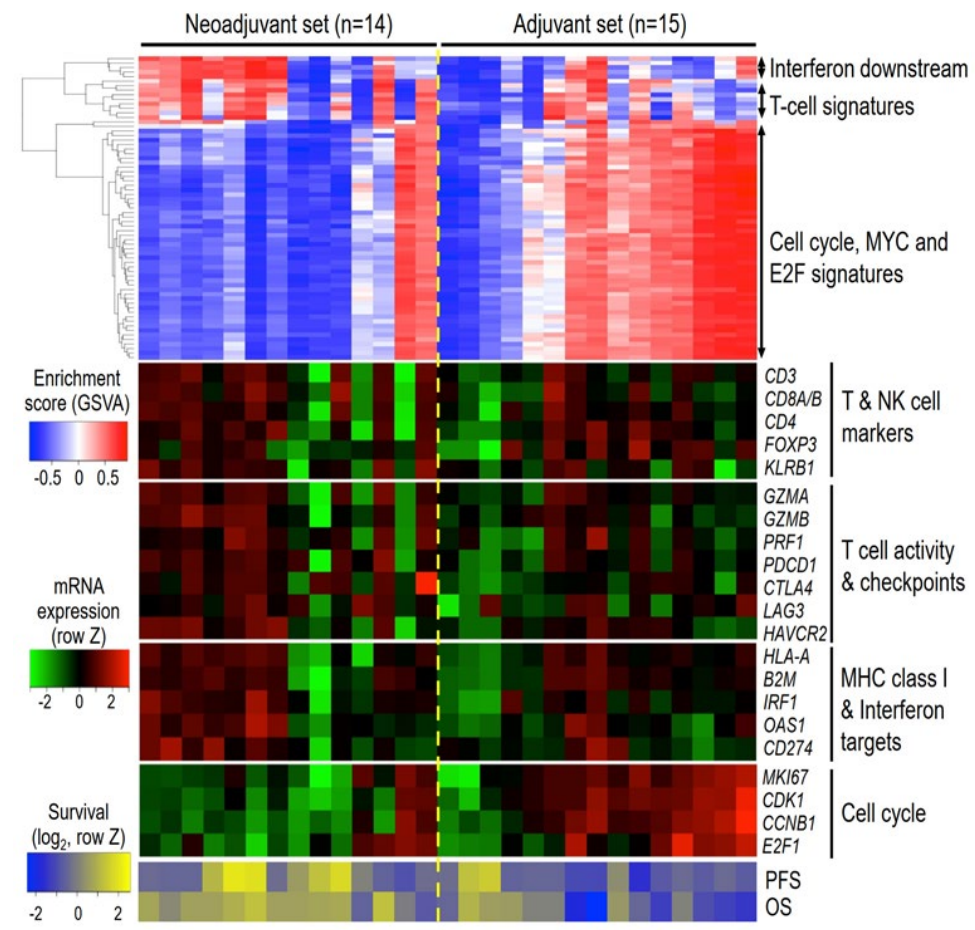

Figure 3: Kaplan–Meier plot of overall survival showing significantly increased survival in patients **(red)** who received neoadjuvant pembrolizumab compared to those who received only adjuvant pembrolizumab **(blue).**

Figure 4: (Top) Heatmap showing the GSVA enrichment scores of gene sets with IQR ≥ 1. The gene sets can be grouped into the three categories: 1) interferon pathway induction, 2) T-cell activity and 3) cell cycle/proliferation.

In a similar study, Schalper et al conducted a single-arm phase II clinical trial in which 30 glioblastoma patients (27 recurrent; 3 newly-diagnosed) received a presurgical dose of nivolumab followed by postsurgical nivolumab until disease progression or unacceptable toxicity [47]. Analysis of tumor tissue pre- and post-nivolumab dosing and from additional patients who did not receive nivolumab showed that neoadjuvant nivolumab also resulted in enhanced expression of chemokine transcripts, higher immune cell infiltration and augmented TCR clonal diversity among tumor-infiltrating T lymphocytes, supporting a local immunomodulatory effect of treatment. Although the median survival was only 7.3 months, two of the three patients treated with nivolumab before and after surgery remain alive 33 and 28 months later.

These studies suggest that neoadjuvant administration of PD1 antibody blockade may enhance both the local and systemic antitumor immune response and represents a potentially promising therapeutic strategy for treating glioblastoma. Similar promising results have been observed in neoadjuvant studies using checkpoint inhibitors [38] in a variety of systemic tumors including melanoma [48, 49] and non-small cell lung cancer [50].

2.3.1.1 Rational for Combination of PD1 Antibody with CTLA4 Antibody

In our study of neoadjuvant pembrolizumab [36], there was no significant change in T cell receptor diversity or TIL density, supporting previous findings that diversification of the T cell repertoire is not mediated via the PD-1 axis, but rather with CTLA-4 antibody blockade [51-53]. However, as our data indicate that increased baseline T cell receptor clonality may be associated with reduced survival, it appears that greater initial T cell diversity may portend improved responsiveness to PD-1 blockade. Our data suggest a role for combination immunotherapy with CTLA-4 blockade, which was shown to increase the number and

complexity of T cell receptor variants in patients with melanoma [51, 53] and has been demonstrated to be efficacious in other tumor types [54]. Although this combination was evaluated in a small cohort of recurrent glioblastoma patients lacking an obvious survival signal [55], it has yet to be explored in a neoadjuvant investigation. In this study we will evaluate whether neoadjuvant administration of the combination of the CTLA4 antibody ipilimumab with the PD1 antibody nivolumab with increase the antitumor immune response and outcome in patients with recurrent glioblastoma.

2.3.2 **Rationale for Endpoints**

2.3.2.1 Efficacy Endpoints

Efficacy will be a secondary endpoint measured as percent PFS-6.

Progression will be determined as per modified RANO criteria [1] with guidance per iRANO for confirmation of progression on follow-up imaging as provided by iRANO criteria [2] as assessed by the investigator will be used for efficacy endpoints (Section 11). RANO will also be used by the local site to determine eligibility and make treatment decisions.

2.3.2.2 Safety Endpoints

An important primary objective of this study is to characterize the safety and tolerability of nivolumab and the combination of nivolumab plus ipilimumab when administered in the pre- and post-operative setting in surgically resectable recurrent glioblastoma. The safety analysis will be based on subjects who experience toxicities as defined by CTCAE v4.0 criteria. Safety will be assessed by quantifying the toxicities and grades experienced by subjects who have received nivolumab and the combination of nivolumab plus ipilimumab including serious adverse events (SAEs) and events of clinical interest (ECIs).

Safety will be assessed by reported adverse experiences using CTCAE v4.0. The attribution to drug, time-of-onset, duration of the event, its resolution, and any concomitant medications administered will be recorded. AEs will be analyzed including but not limited to all AEs, SAEs, and fatal AEs. Furthermore, specific immune-related adverse events (irAEs) will be collected and designated as immune-related events of clinical interest (ECIs) as described in Section 7.3.3.

2.4 Correlative Studies Background

Despite clinical response to immunotherapies across multiple cancers, the majority of participants fail to respond due to incompletely defined mechanisms. To maximize the impact of therapy, well-characterized biomarkers are needed.

We will correlate biomarkers with pharmacodynamic activity and bioactivity of nivolumab and ipilimumab, in search of a novel tumor tissue-based assay that may predict efficacy, allow for

monitoring of response, and identify potential subclinical immune-driven toxicity.

The following biomarkers will be assessed:

- TIL density and TCR clonality will be quantitatively assessed with next generation TCR sequencing.[39]
- Tumor and peripheral blood gene expression signatures will be measured by RNA-seq and Nanostring IO360.
- T-cell inflamed gene expression profiling will evaluate tumor mRNA data and assess unsupervised hierarchical clustering.[46]
- Tumor and peripheral blood T cell subsets and activation markers will be assessed with flow cytometry to identify immune cell subsets that expand or contract in an unbiased fashion during treatment.
- Tumor quantification of PD-1, PD-L1, CD3, CD4, CD8, Iba-1, Ki-67 will be measured by immunohistochemistry of FFPE tissue. Multiplex immunofluorescence will be used to spatially quantitate TIL in the tumor microenvironment.

Recent work has confirmed that TIL density, TCR clonality, IFNγ-related genes and related gene expression signatures in the recurrent glioblastoma tumor microenvironment can be reliably quantified and correlate with clinical responses to neoadjuvant PD-1 antibodies. [26]

- A subset of participants will receive ^{89}Zr-Df-IAB22M2C and undergo PET imaging at baseline prior to initiation of therapy and again approximately 3 days before surgery +/- 2 days. Baseline ^{89}Zr-Df-IAB22M2C uptake will be compared to post-neoadjuvant therapy values between the treatment arms. ^{89}Zr-Df-IAB22M2C uptake after neoadjuvant immune checkpoint therapy will also be compared to CD8+ TIL density by IHC from resected tumors.

3. PARTICIPANT SELECTION

Male/female subjects of at least 18 years of age with glioblastoma who are candidates for surgical tumor debulking will be enrolled in this trial.

Screening evaluations are detailed in Study Calendar (Section 10). All assessments are to occur within 14 days of registration except where otherwise noted. The participant must be thoroughly informed about all aspects of the study, including the study visit schedule and required evaluations and all regulatory requirements for informed consent. The written informed consent must be obtained from the participant prior to enrollment.

Following registration, any additional laboratory assessments obtained prior to start of treatment will not be used to re-confirm eligibility. Please refer to Section 6 for toxicity management between registration and start of study treatment.

3.1 Eligibility Criteria

All the following criteria must be meet on screening examination to be eligible for participation:

3.1.1 Have histologically confirmed World Health Organization Grade IV *IDH* wildtype glioblastoma or variants including gliosarcoma or *IDH* wildtype glioma with molecularly features of glioblastoma [56].

3.1.2 Previous first line therapy with at least radiotherapy.

3.1.3 Patients must be undergoing surgery that is clinically indicated as determined by their care providers.

3.1.4 Be at first or second relapse. **Note**: Relapse is defined as progression following initial therapy (i.e., radiation ± chemotherapy).

3.1.5 Participants must have shown unequivocal evidence for tumor progression by MRI per RANO criteria.

3.1.6 Participants must have confirmation of availability of sufficient tissue from prior surgery revealing glioblastoma or variants for submission following registration. The following amount of tissue is required:

- 1 formalin-fixed paraffin-embedded (FFPE) tumor tissue block (preferred)

OR

- 10 FFPE unstained slides (5 µm thick)

3.1.7 An interval of at least 12 weeks from the completion of radiation therapy to registration unless there is unequivocal histologic confirmation of tumor progression.

3.1.8 Participants must have recovered to grade 0 or 1 or pre-treatment baseline from clinically significant toxic effects of prior therapy (including but not limited to exceptions of alopecia, laboratory values listed per inclusion criteria, and lymphopenia which is common after therapy with temozolomide).

3.1.9 An interval of at least 4 weeks (to registration) between prior surgical resection or one week for stereotactic biopsy.

3.1.10 From registration, the following time periods must have elapsed: 5 half-lives from any investigational agent, 4 weeks from cytotoxic therapy (except 23 days for temozolomide and 6 weeks from nitrosoureas), 4 weeks from antibodies, or 4 weeks (or 5 half-lives, whichever is shorter) from other anti-tumor therapies (including vaccines). No wash-out period required from tumor treating fields (TTF).

3.1.11 Be willing and able to provide written informed consent/assent for the trial.

3.1.12 Be ≥ 18 years of age on day of signing informed consent.

3.1.13 Have a Karnofsky performance status (KPS) ≥ 70 (Appendix A).

3.1.14 MRI within 14 days prior of registration.

3.1.15 Demonstrate adequate organ function as defined below in

3.1.16 **Table 1**, all screening labs should be performed within 14 days of registration.

Table 1: Adequate Organ Function Laboratory Values

System	Laboratory Value
**Hematological**	
Absolute neutrophil count (ANC)	≥1,500 /mcL
Platelets	≥100,000 / mcL

Hemoglobin	≥9 g/dL or ≥5.6 mmol/L without transfusion or EPO dependency (within 7 days of assessment)
**Renal**	
Serum creatinine **OR** measured or calculated [a] creatinine clearance (GFR can be used in place of creatinine or CrCl)	≤1.5 X institutional upper limit of normal (ULN) **OR** ≥60 mL/min for subject with creatinine levels > 1.5 X institutional ULN
[a] Creatinine clearance should be calculated per institutional standard.	
**Hepatic**	
Serum total bilirubin	≤ 1.5 X institutional ULN **OR**<br>Direct bilirubin ≤ institutional ULN for subjects with total bilirubin levels > 1.5 institutional ULN
AST (SGOT) and ALT (SGPT)	≤ 2.5 X institutional ULN **OR**<br>≤ 5 X institutional ULN for subjects with Gilberts syndrome
Albumin	≥2.5 mg/dL
**Coagulation**	
International Normalized Ratio (INR) or Prothrombin Time (PT)	≤1.5 X institutional ULN unless subject is receiving anticoagulant therapy as long as PT or PTT is within therapeutic range of intended use of anticoagulants
Activated Partial Thromboplastin Time (aPTT)	≤1.5 X institutional ULN unless subject is receiving anticoagulant therapy as long as PT or PTT is within therapeutic range of intended use of anticoagulants

3.1.17 Female subject of childbearing potential should have a negative urine or serum pregnancy within 72 hours prior to registration. If the urine test is positive or cannot be confirmed as negative, a serum pregnancy test will be required. Women are considered post-menopausal and not of child bearing potential if they have had 12 months of natural (spontaneous) amenorrhea with an appropriate clinical profile (e.g., age appropriate, history of vasomotor symptoms) or six months of spontaneous amenorrhea with serum FSH levels > 40 mIU/mL and estradiol < 20 pg/mL or have had surgical bilateral oophorectomy (with or without hysterectomy) at least six weeks ago. In the case of oophorectomy alone, only when the reproductive status of the woman has been confirmed by follow up hormone level assessment is she considered not of child bearing potential.

3.1.18 Women of child-bearing potential (WOCBP), defined as all women physiologically capable of becoming pregnant, must use highly effective contraception during study treatment and for 5 months after study discontinuation. Highly effective contraception is defined as either:

a. True Abstinence: When this is in line with the preferred and usual lifestyle of the subject. Periodic abstinence (e.g., calendar, ovulation, symptothermal, post-ovulation methods) and withdrawal are not acceptable methods of contraception.

b. Sterilization: Surgical bilateral oophorectomy (with or without hysterectomy) or tubal ligation at least six weeks ago. In case of oophorectomy alone, only when the reproductive status of the woman has been confirmed by follow up hormone

level assessment (as described in item 12 above).

c. Male Partner Sterilization (with the appropriate post-vasectomy documentation of the absence of sperm in the ejaculate). For female subjects on the study, the vasectomised male partner should be the sole partner for that participant.

d. Use of a combination of any two of the following:

1. Placement of an intrauterine device (IUD) or intrauterine system (IUS)
2. Barrier methods of contraception: Condom or Occlusive cap (diaphragm or cervical/vault caps) with spermicidal foam/gel/film/cream/vaginal suppository
3. Appropriate hormonal contraceptives (including any registered and marketed contraceptive agent that contains an estrogen and/or a progestational agent – including oral, subcutaneous, intrauterine, or intramuscular agents).

3.1.19 Male subjects should agree to use adequate method of contraception starting with the first dose of study therapy through 7 months after the last dose of therapy.

3.2 Exclusion Criteria

The subject must be excluded from participating in the trial if the subject:

3.2.1 IDH mutation by immunohistochemistry.

3.2.2 Current or planned participation in a study of an investigational agent or using an investigational device.

3.2.3 Has a diagnosis of immunodeficiency.

3.2.4 Has tumor primarily localized to the brainstem or spinal cord.

3.2.5 Has presence of diffuse leptomeningeal disease or extracranial disease.

3.2.6 Has received systemic immunosuppressive treatments, aside from systemic corticosteroids (such as methotrexate, chloroquine, azathioprine, etc) within six months of registration.

3.2.7 Has received bevacizumab or aflibercept. VEGFR inhibitors are allowed.

3.2.8 Requires treatment with high dose systemic corticosteroids defined as dexamethasone > 2 mg/day or bioequivalent for at least 3 consecutive days within 2 weeks of registration.

3.2.9 Has received prior interstitial brachytherapy, implanted chemotherapy, stereotactic radiosurgery or therapeutics delivered by local injection or convection enhanced delivery.

3.2.10 Has history of known coagulopathy that increases risk of bleeding or a history of clinically significant hemorrhage within 12 months of registration.

3.2.11 Has a known history of active TB (Bacillus Tuberculosis).

3.2.12 Has gastrointestinal bleeding or any other hemorrhage/bleeding event CTCAE Grade > 3 within 6 months of registration.

3.2.13 Has a known additional malignancy that is progressing or requires active treatment. Those patients whose natural history or treatment does not have the potential to interfere with the safety or efficacy assessment of the investigational regimen will be eligible including, but not limited to, basal cell carcinoma of the skin, squamous cell carcinoma of the skin, localized prostate cancer not requiring treatment, or in situ cervical cancer that has undergone potentially curative therapy.

3.2.14 Has active autoimmune disease that has required systemic treatment in the past 2 years (i.e. with use of disease modifying agents, corticosteroids or immunosuppressive drugs). Replacement therapy (e.g., thyroxine, insulin, or physiologic corticosteroid replacement therapy for adrenal or pituitary insufficiency, etc.) is not considered a form of systemic treatment.

3.2.15 Has known history of, or any evidence of active non-infectious pneumonitis.

3.2.16 Has an active infection requiring systemic therapy.

3.2.17 Has a history or current evidence of any condition, therapy, or laboratory abnormality that might confound the results of the trial, interfere with the subject's participation for the full duration of the trial, or is not in the best interest of the subject to participate, in the opinion of the treating investigator. Examples include but are not limited to symptomatic congestive heart failure, unstable angina pectoris, cardiac arrhythmia or psychiatric illness/social situations that would limit compliance with study requirements.

3.2.18 Has known psychiatric or substance abuse disorders that would interfere with cooperation with the requirements of the trial.

3.2.19 Is pregnant, breastfeeding, or expecting to conceive within the projected duration of the trial, starting with the screening visit through 5 months after the last dose of trial treatment. It is unknown whether nivolumab and/or ipilimumab is excreted in human milk or may have adverse effects on a fetus in utero. Since many drugs are excreted in human milk, and because of the potential for serious adverse reactions in the nursing infant or fetus, these subjects are not eligible for enrollment.

3.2.20 Has received prior therapy with an anti-PD-1, anti-PD-L1, anti-PD-L2, anti-CD137, or anti-Cytotoxic T-lymphocyte-Associated Antigen-4 (CTLA-4) antibody (including ipilimumab or any other antibody or drug specifically targeting T-cell co-stimulation or checkpoint pathways).

3.2.21 Has a known history of Human Immunodeficiency Virus (HIV) (HIV 1/2 antibodies).

3.2.22 Has known active Hepatitis B (e.g., HBsAg reactive) or Hepatitis C (e.g., HCV RNA [qualitative] is detected).

3.2.23 Has received a live vaccine within 30 days prior to registration.

3.2.24 Has a known hypersensitivity to any of the study therapy products.

3.3 Inclusion of Women and Minorities

Both men and women of all races and ethnic groups are eligible for this trial.

4. REGISTRATION AND RANDOMIZATION PROCEDURES

4.1 General Guidelines for DF/HCC Institutions

Institutions will register eligible participants in the Clinical Trials Management System (CTMS) OnCore. Registrations must occur prior to the initiation of any protocol-specific therapy or intervention. Any participant not registered to the protocol before protocol-specific therapy or intervention begins will be considered ineligible and registration will be denied.

An investigator will confirm eligibility criteria and a member of the study team will complete the ODQ protocol-specific eligibility checklist.

The eligibility checklist(s) and all pages of the consent form(s) will be faxed to the ODQ at 617-632-2295 or emailed to their central or designee. The ODQ will (a) review the eligibility checklist, (b) register the participant on the protocol, and (c) randomize the participant.

Randomization can only occur during ODQ business hours (8:30am – 5:00pm Eastern Time, Monday through Friday excluding holidays).

An email confirmation of the registration will be sent to the Overall PI, study coordinator(s) from the Lead Site, treating investigator and registering person immediately following the registration and/or randomization.

An email confirmation of the randomization assignment will be sent to the registering site's study pharmacist and the lead site's Unblinded Contact (or back-up) immediately following the registration and/or randomization.

Following registration, participants may begin protocol-specific therapy and/or intervention. Issues that would cause treatment delays should be discussed with the Principal Investigator (PI) of the registering site. If the subject does not receive protocol therapy following registration, the subject must be taken off study in the CTMS (OnCore) with an appropriate date and reason entered.

4.2 Registration Process for DF/HCC Institutions

Applicable DF/HCC policy (REGIST-101) must be followed. And refer to Appendix B (DSMP), Section 3.5.

4.3 General Guidelines for Other Investigative Sites

Eligible participants will be entered on study centrally at the DFCI Coordinating Center by the Study Coordinator. All sites should contact the Study Coordinator at NeuroOnc_Coor@dfci.harvard.edu to verify slot availability. See Appendix B, Section 3.5 (DF/HCC Multi-Center Data and Safety Monitoring Plan) for complete registration procedures and required forms.

Following registration, participants should begin protocol therapy within 5 days. Issues that would cause treatment delays should be discussed with the Sponsor-Investigator. If the subject does not receive protocol therapy following registration, the subject must be taken off study in the CTMS (OnCore) with an appropriate date and reason entered

4.4 Registration Process for Other Investigative Sites

See Appendix B Section 3.5 (DF/HCC Multi-Center Data and Safety Monitoring Plan) for complete registration procedures.

5. TREATMENT PLAN

5.1 Treatment Regimen

5.1.1 **Study Cohorts and Treatment Arms**

All participants will have a known diagnosis of glioblastoma.

Within 5 days from enrollment, subjects will be sequentially randomized to one of three treatment arms as described below. The neoadjuvant portion of this study will be blinded and placebo controlled. Subjects in Arm 1 will receive neoadjuvant treatment with nivolumab plus ipilimumab, subjects in Arm 2 will receive nivolumab plus placebo, while subjects in Arm 3 will not receive neoadjuvant checkpoint therapy but will receive respective placebo dosing prior to the planned surgery. **This randomization scheme should not negatively impact study accrual and will provide an invaluable control cohort for pharmacodynamic (PD) evaluations.** Post-operatively, all subjects will receive nivolumab plus ipilimumab, followed by nivolumab monotherapy as described below.

> Arm 1: 25 eligible patients will receive a single IV infusion of nivolumab at 3 mg/kg and a single IV infusion of ipilimumab at 1 mg/kg within 14±5 days from the scheduled surgical resection for recurrent glioblastoma. After recovery from surgery (but no more than 35 days following surgery), subjects will receive combination treatment with ipilimumab at 1 mg/kg every 3 weeks for 3 doses plus nivolumab at 3 mg/kg every 3 weeks for the first 9 weeks of post-operative treatment period. Thereafter these subjects will continue to receive nivolumab at 480 mg every 4 weeks.
>
> Arm 2: 25 eligible patients will receive a single IV infusion of nivolumab at 3 mg/kg and a single IV infusion ipilimumab-placebo within 14 ± 5 days from the scheduled surgical resection for recurrent glioblastoma. After recovery from surgery (but no more than 35 days following surgery), subjects will receive combination treatment with ipilimumab at 1 mg/kg every 3 weeks for 3 doses plus nivolumab at 3 mg/kg every 3 weeks for the first 9 weeks of post-operative treatment period. Thereafter these subjects will continue to receive nivolumab at 480 mg every 4 weeks.
>
> Arm 3: 10 eligible patients will receive a single IV infusion nivolumab-placebo and a single IV infusion ipilimumab-placebo within 14 ± 5 days from the scheduled surgical resection for recurrent glioblastoma. After recovery from surgery (but no more than 35

days following surgery), subjects will receive combination treatment with ipilimumab at 1 mg/kg every 3 weeks for 3 doses plus nivolumab at 3 mg/kg every 3 weeks for the first 9 weeks of post-operative treatment period. Thereafter these subjects will continue to receive nivolumab at 480 mg every 4 weeks.

^{89}Zr-Df-IAB22M2C PET Imaging: Up to 20 participants will undergo ^{89}Zr-Df-IAB22M2C administration and PET imaging prior to neoadjuvant therapy and 3 days +/- 2 days before surgical resection. The dose of ^{89}Zr-Df-IAB22M2C will be 1 mCi of ^{89}Zr-Df-IAB22M2C at 1.5 mg of minibody based on safety established in the prior phase 1 study.[31]

Subjects will be treated with nivolumab until criteria for treatment discontinuation is met (Section 5.5). If criteria for treatment discontinuation is not met, patients will be treated with nivolumab for a period of 2 years but can continue to be treated beyond that point if they are clinically benefiting from the drug, after discussion with the Sponsor-Investigator.

Tumor samples will be obtained from all subjects at time of surgery. The tissue from this surgery (fresh, frozen and FFPE) and the archived tissue from the initial surgery (FFPE) will be processed so as to best achieve the primary, secondary and exploratory PD objectives. A post-operative MRI scan should be performed with 72 hours of surgery; preferably within 24 hours.

Blood samples will be obtained from all subjects as pharmacodynamic markers throughout the study. Dose decreases and symptomatic management will occur based upon preset adverse event determination. DLTs will not be determined. The toxicity evaluation period will begin with registration and extend to 30 days after last treatment day. Subjects will be followed for MRI changes, clinical exam and steroid doses from the registration and extend until confirmed progression. After confirmed progression, subjects will be followed every 3 months for vital status until death.

The investigator shall take responsibility for and shall take all steps to maintain appropriate records and ensure appropriate supply, storage, handling, distribution, and usage of trial treatments in accordance with the protocol and any applicable laws and regulations.

5.1.2 **Trial Blinding/Masking**

The neoadjuvant treatment period of the trial will be blinded, and the subjects, Sponsor, site investigators and site staff will not know the treatment administered. An assigned Site Investigational Pharmacist will not be blinded. Designated staff of the DF/HCC Office of Data Quality will be unblinded to facilitate randomization and safety monitoring. Blinding of treatment assignment is critical to the integrity of this clinical study. However, in the event of a medical emergency or pregnancy in an individual subject in which knowledge of the investigational product is critical to the subject's management, the blind for that subject may be broken by the investigator. The subject's safety takes priority over any other considerations in determining if the treatment assignment should be unblinded.

The post-operative treatment period will be unblinded; therefore, the Sponsor, site investigators,

site staff and subject will know the treatment administered.

5.1.3 Patient Evaluability and Replacement

A subject will be replaced if any of the following criteria was met:

1) insufficient tumor tissue can be collected at protocol surgery
2) histopathology from the protocol surgery is confirmed as non-glioblastoma
3) the subject was unable to receive planned neoadjuvant treatment

Additional participants may be screened and randomized to the trial to make up the required number of at least 25 evaluable participants in Arm 1 and 2 respectively, and at least 10 evaluable participants in Arm 3.

A participant that discontinues the trial for progressive disease or a drug-related AE after the completion of the surgical resection will not be replaced and will be counted in the evaluable population of participant for the respective cohort.

5.2 Pre-Treatment Criteria

Dosing will continue unless criteria for dose delay (Section 6.2.3 for nivolumab and Section 6.3.3 for ipilimumab) or treatment discontinuation (Section 5.5) are met. In the event of a dose delay, dosing will be resumed when criteria to resume treatment (Section 6.2.4 for nivolumab and 6.3.4 for ipilimumab) are met.

5.3 Agent Administration

5.3.1 Neoadjuvant Dose Administration

Participants in Arms 1 and 2 will receive a single dose of immune checkpoint inhibitor treatment within 14 ± 5 days prior to scheduled surgical resection while those in Arm 3 will not receive neoadjuvant therapy but only placebo control as follows:

Arm 1: nivolumab at 3 mg/kg IV and ipilimumab at 1 mg/kg IV

Arm 2: nivolumab at 3 mg/kg IV and ipilimumab-placebo IV

Arm 3: nivolumab-placebo IV and ipilimumab-placebo IV

The neoadjuvant treatment to be used in this trial is outlined below.

Table 2: Neoadjuvant Trial Treatment Administration

Agent	Dose [a]	Frequency	Route [b]	Schedule	Use
Nivolumab /placebo [c]	3 mg/kg	Once	30 minute Intravenous (IV) infusion	Within 14 ± 5 days prior to scheduled surgical resection	Experimental
Ipilimumab /placebo [c]	1 mg/kg	Once	90 minute Intravenous (IV) infusion	Within 14 ± 5 days prior to scheduled surgical resection	Experimental

Agent	Dose [a]	Frequency	Route [b]	Schedule	Use
[a] Appropriate dose modifications are described in Section 6.<br>[b] Infusions should be completed within a -10/+30 minute window. When both nivolumab and ipilimumab are administered, nivolumab will be administered first with a 30-minute observation window before starting ipilimumab. At the end of the infusion, flush the line with a sufficient quantity of normal saline.<br>[c] matched infusions (0.9% sodium chloride for injection or 5% dextrose for injection) will be provided to subjects					

5.3.2 Post-Operative Therapy

For all participants, post-operative immune checkpoint therapy will begin upon recovery from surgery (once participant has recovered from surgery), but no more than 35 days following surgery as follows:

All Arms: ipilimumab at 1 mg/kg plus nivolumab at 3 mg/kg every 3 weeks for 3 doses over the first 9 weeks of post-operative therapy period followed by nivolumab at 480 mg every 4 weeks_During the combination treatment period, nivolumab and ipilimumab should be administered on Days 1 of Weeks 1, 4, and 7 after all procedures/assessments have been completed and reviewed as detailed on the Study Calendar in Section 10. During the nivolumab alone treatment period, nivolumab should be administered on Days 1 of each 4-week cycle after all procedures/assessments have been completed and reviewed as detailed on the Study Calendar in Section 10.

There will be a 2-day window for all treatment doses that are administered.

The post-operative treatment to be used in this trial is outlined below.

Table 3: Post-Operative Trial Treatment

	Agent [c]	Dose [a]	Frequency/ Cycle Length	Route [b]	Schedule	Use
**All arms**	Nivolumab	3 mg/kg	Every 3 weeks	30 minute IV infusion	Day 1 of each cycle, for first 3 cycles	Experimental
	Ipilimumab	1 mg/kg	Every 3 weeks	90 minute IV infusion	Day 1 of each cycle, for first 3 cycles	Experimental
	Nivolumab	480 mg	Every 4 weeks	30 minute IV infusion	Day 1 of each cycle beginning at Cycle 4	Experimental
[a] Appropriate dose modifications are described in Section 6.<br>[b] Infusions should be completed within a -10/+30 minute window. When both nivolumab and ipilimumab are administered, nivolumab will be administered first with a 30-minute observation window before starting ipilimumab. At the end of the infusion, flush the line with a sufficient quantity of normal saline.<br>[c] labs/assessment should be completed and reviewed within 7 days from the study drug administration.<br>Note: There is a 2-day administration window for all treatment doses.						

5.3.3 Crossover for Previously Enrolled Participants

Any participants enrolled on treatment arm 2 of the prior version of the protocol (Version 4.0 dated November 29, 2021) and still on treatment at the time of v5 activation will have the option

to cross over to the same post-surgical treatment plan outlined in v5. Subjects who cross over will restart the schedule of assessments at adjuvant C1D1 and follow the same schedule of assessments until any treatment discontinuation criteria are met.

5.4 General Concomitant Medication and Supportive Care Guidelines

Medications or vaccinations specifically prohibited in the exclusion criteria (Section 3.2) are not allowed during the ongoing trial except as outlined below. If there is a clinical indication for one of these or other medications or vaccinations specifically prohibited during the trial, discontinuation from trial therapy or vaccination may be required. The treating investigator should discuss any questions regarding this with the Sponsor-Investigator or his designee. The final decision on any supportive therapy or vaccination rests with the treating. However, the decision to continue the subject on trial therapy or vaccination schedule requires the mutual agreement of the Investigator, Sponsor-Investigator, or his designee, and the subject.

5.4.1 ^{89}Zr-Df-IAB22M2C

Up to 20 participants will receive a dose of 1.0 (±20%) mCi of ^{89}Zr-Df-IAB22M2C with 1.5mg of API administered intravenously over 5-10 minutes prior to neoadjuvant checkpoint therapy and again 3 days +/- 2 days before surgery. Participants will undergo both whole body and dedicated brain PET scan imaging approximately 24 hours after each ^{89}Zr-IAB22M2C infusion.

The dispensed dose will be infused under the supervision of a qualified nuclear medicine personnel as an IV infusion with a shielded infusion pump over no less than 5 minutes followed by saline flush. The total infusion time should be between 5 to 10 minutes. Vital signs will be assessed 15 (+/- 5 minutes) prior to infusion and within 60 minutes post infusion of ^{89}Zr-IAB22M2C.

5.4.2 Acceptable Concomitant Medications

All treatments that the investigator considers necessary for a subject’s welfare may be administered at the discretion of the investigator in keeping with the community standards of medical care. All concomitant medication will be recorded on the case report form (CRF) including all prescription, over-the-counter (OTC), herbal supplements, and IV medications and fluids. If changes occur during the trial period, documentation of drug dosage, frequency, route, and date may also be included on the CRF, when information is available.

All concomitant medications received from date of consent up to 30 days after the last dose of trial treatment should be recorded. Concomitant medications administered after 30 days after the last dose of trial treatment should be recorded for SAEs and ECIs.

5.4.3 Prohibited Concomitant Medications

Subjects are prohibited from receiving the following therapies during the Screening and Treatment Phase of this trial:

- Anti-cancer systemic chemotherapy or biological therapy

- Immunotherapy not specified in this protocol
- Investigational agents other than nivolumab, ipilimumab and ^{89}Zr-Df-IAB22M2C
- Live vaccines within 30 days prior to the first dose of trial treatment and while participating in the trial. Examples of live vaccines include, but are not limited to, the following: measles, mumps, rubella, varicella/zoster, yellow fever, rabies, BCG, and typhoid vaccine.
- Systemic glucocorticoids for any purpose other than to modulate symptoms from an event of clinical interest of suspected immunologic etiology or to control cerebral edema. The use of physiologic doses of corticosteroids are allowed.

Subjects who, in the assessment by the investigator, require the use of any of the aforementioned treatments for clinical management should be removed from the trial. Subjects may receive other medications that the investigator deems to be medically necessary.

Exclusion Criteria in Section 3.2 describes other medications which are prohibited in this trial.

There are no prohibited therapies during the Post-Treatment Follow-up Phase.

5.4.4 **Supportive Care Guidelines**

Subjects should receive appropriate supportive care measures as deemed necessary by the treating investigator. Suggested supportive care measures for the management of adverse events with potential immunologic etiology are outlined in Appendix C. Where appropriate, these guidelines include the use of oral or intravenous treatment with corticosteroids as well as additional anti-inflammatory agents if symptoms do not improve with administration of corticosteroids. Note that several courses of steroid tapering may be necessary as symptoms may worsen when the steroid dose is decreased. For each disorder, attempts should be made to rule out other causes such as bacterial or viral infection, which might require additional supportive care. The treatment guidelines are intended to be applied when the investigator determines the events to be related to nivolumab or ipilimumab.

Note: if after the evaluation the event is determined not to be related, the investigator does not need to follow the treatment guidance (as outlined in Appendix C). Refer to Section 6 for dose modification.

It may be necessary to perform conditional procedures such as bronchoscopy, endoscopy, or skin photography as part of evaluation of the event.

5.4.5 **Cerebral Edema**

Due to the immunologic nature of nivolumab and ipilimumab administration, cerebral edema could theoretically result as a consequence of nivolumab and/or ipilimumab administration due to immune infiltration of the brain. Symptoms related to cerebral edema may include headache or neurologic deficit that is either new or worsened. Patients with any signs or symptoms of cerebral edema should be treated as clinically appropriate including initiation or increased systemic corticosteroid dosing, treatment with an osmotic diuretic or surgical decompression. **In selected patients, after discussion with Sponsor-Investigator, bevacizumab may be used for treatment of peritumoral edema.**

Subsequent nivolumab and/or ipilimumab dosing should be immediately interrupted if significant clinical symptoms attributable to cerebral edema develop. Treatment with additional nivolumab and/or ipilimumab doses may only be re-initiated if clinically significant symptoms attributable to cerebral edema have resolved to grade ≤ 1 or pre-treatment baseline. Patients who develop CTCAE v4.0 Grade 4 cerebral edema attributable to nivolumab or ipilimumab administration should not receive further nivolumab and/or ipilimumab doses and should discontinue study therapy.

5.4.6 **Diet**

Subjects should maintain a normal diet unless modifications are required to manage an AE such as diarrhea, nausea or vomiting.

5.4.7 **Contraception**

Nivolumab and ipilimumab may have adverse effects on a fetus in utero. Furthermore, it is not known if nivolumab and/or ipilimumab has transient adverse effects on the composition of sperm. Non-pregnant, non-breast-feeding women may be enrolled if they are willing to use 2 methods of birth control or are considered highly unlikely to conceive. Highly unlikely to conceive is defined as 1) surgically sterilized, or 2) postmenopausal (a woman who is ≥45 years of age and has not had menses for greater than 1 year will be considered postmenopausal), or 3) not heterosexually active for the duration of the study. The two birth control methods can be either two barrier methods or a barrier method plus a hormonal method to prevent pregnancy. Subjects should start using birth control from study Visit 1 throughout the study period up to 5 months after the last dose of study therapy.

The following are considered adequate barrier methods of contraception: diaphragm, condom (by the partner), copper intrauterine device, sponge, or spermicide. Appropriate hormonal contraceptives will include any registered and marketed contraceptive agent that contains an estrogen and/or a progestational agent (including oral, subcutaneous, intrauterine, or intramuscular agents).

Subjects should be informed that taking the study medication may involve unknown risks to the fetus (unborn baby) if pregnancy were to occur during the study. In order to participate in the study, they must adhere to the contraception requirement (described above) for the duration of the study and during the follow-up period defined in Section 7.3.6 Reporting of Pregnancy and Lactation to the Sponsor and to BMS. If there is any question that a subject will not reliably comply with the requirements for contraception, that subject should not be entered into the study.

5.5 Criteria for Taking a Participant Off Treatment

Duration of therapy will depend on individual response, evidence of disease progression and tolerance. In the absence of treatment delays due to adverse event(s), treatment may continue until one of the following criteria applies:

- The subject (or legal representative) withdraws consent

- Confirmed radiographic disease progression
  Note: For unconfirmed radiographic disease progression, see Section 11
  Note: A subject may be granted an exception to continue treatment with confirmed radiographic progression if clinically stable or clinically improved, see Section 11.
- Unacceptable adverse event(s)
- Intercurrent illness that prevents further administration of treatment
- Investigator's decision to withdraw the subject
- The subject has a confirmed positive serum pregnancy test
- Noncompliance with trial treatment or procedure requirements
- General or specific changes in the participant's condition render the participant unacceptable for further treatment in the judgment of the treating investigator

Participants will be removed from the protocol therapy when any of these criteria apply. The reason for removal from protocol therapy, and the date the participant was removed, must be documented in the case report form (CRF). Alternative care options will be discussed with the participant.

When a participant is removed from protocol therapy and/or is off of the study, the participant's status must be updated in OnCore in accordance with REGIST-OP-1.

In the event of unusual or life-threatening complications, treating investigators must immediately notify the Sponsor-Investigator, Patrick Wen, M.D.

5.6 Duration of Follow-Up

The End of Treatment and Follow-up visit procedures are listed in Section 10 (Study Calendar). After the end of treatment, each subject will be followed for 30 days for adverse event monitoring and 100 days for serious adverse event reporting.

Participants removed from protocol therapy for unacceptable adverse events will be following until resolution or stabilization of the adverse event.

Subjects who discontinue for reasons other than progressive disease will have post-treatment follow-up for disease status until disease progression, initiating a non-study cancer treatment, withdrawing consent or becoming lost to follow-up.

5.7 Criteria for Taking a Patient Off Study

After documented disease progression each subject will be followed by telephone or medical record review for overall survival until they meet criteria for removal from study as detailed below.

Participants will be removed from study when any of the following criteria apply:

- Lost to follow-up
- Withdrawal of consent for data submission
- Death

The reason for taking a participant off study, and the date the participant was removed, must be documented in the case report form (CRF). In addition, the study team will ensure the participant's status is updated in OnCore in accordance with REGIST-OP-1.

5.8 Beginning and End of the Trial

The study begins when the first subject signs the informed consent (either pre-screening consent or main study consent). The end of the study may be designated as the time point when all subjects have discontinued the study or are a minimum of 6 months post initial study medication administration. If, by the end of the study, there remains at least 1 subject still on study treatment for at least 6 months, the subject(s) may enter additional treatment cycles.

The subject is considered on study until such time that he/she meets any of the discontinuation criteria and notification is given to the Sponsor.

6. DOSING DELAYS/DOSE MODIFICATIONS

Dosing delays and modifications of nivolumab and ipilimumab will be made as indicated below. The descriptions and grading scales found in the revised NCI Common Terminology Criteria for Adverse Events version 4.0 (CTCAE v4.0) will be utilized for dose delays and dose modifications. A copy of the CTCAE v4.0 can be downloaded from the CTEP website http://ctep.cancer.gov/protocolDevelopment/electronic_applications/ctc.htm.

If possible, symptoms should be managed as needed. In the case of toxicity, appropriate medical treatment should be used.

All adverse events experienced by participants will be collected from the time of the first dose of study treatment (first nivolumab or ipilimumab administration), through the study and until the 30-Day Post Drug study visit. Participants continuing to experience toxicity at the end of treatment visit may be contacted for additional assessments until the toxicity has resolved or is deemed irreversible.

6.1 Toxicity Management

Adverse events (both non-serious and serious) associated with nivolumab or ipilimumab exposure may represent an immunologic etiology. These adverse events may occur shortly after the first dose or several months after the last dose of treatment. Immune checkpoint therapy with nivolumab, or the combination of nivolumab plus ipilimumab must be withheld for drug-related toxicities (considered at least possibly related to study treatment) that are severe or life-threatening AEs. If a participant experiences a toxicity unlikely or unrelated to treatment with nivolumab or the combination of nivolumab plus ipilimumab, but may still warrant a hold or reduction of study drug for safety, discussion and written approval by the Sponsor-Investigator, Patrick Wen, MD, is recommended but not required.

6.1.1 **General Guidelines**

Subjects should be monitored for infusion reactions during nivolumab/ipilimumab

administration. If an acute reaction is noted, subjects should be managed according to section 6.2.1 for nivolumab and 6.3.1 for ipilimumab.

Appendix C contains management algorithms developed to assist investigators in assessing and managing the following groups of AEs:

- Gastrointestinal
- Renal
- Pulmonary
- Hepatic
- Endocrinopathies
- Skin
- Neurological
- Myocarditis

Doses of nivolumab/ipilimumab may be interrupted, delayed, or discontinued depending on how well the subject tolerates treatment. Nivolumab/placebo or ipilimumab/placebo should be delayed (see Section 6.2.3 and Section 6.3.3) for toxicities considered at least possibly related to nivolumab or possibly-related to ipilimumab.

No incompatibilities have been observed between nivolumab for injection and polyvinyl chloride (PVC), non-PVC/non-DHEP (di(2ethylhexyl)phthalate) IV components or glass bottles.

Dosing interruptions are permitted in the case of medical/surgical events or logistical reasons not related to study therapy (e.g., elective surgery, unrelated medical events, patient vacation, and/or holidays). Subjects should be placed back on study therapy within 3 weeks of the scheduled interruption, unless otherwise discussed with the Sponsor-Investigator. The reason for interruption should be documented in the patient's study record.

6.2 Nivolumab

6.2.1 **Treatment of Nivolumab Related Infusion Reactions**

Since Nivolumab contains only human immunoglobulin protein sequences, it is unlikely to be immunogenic and induce infusion or hypersensitivity reactions. However, if such a reaction were to occur, it might manifest with fever, chills, rigors, headache, rash, pruritis, arthralgias, hypo- or hypertension, bronchospasm, or other symptoms.

There are no premedications recommended for nivolumab on the first cycle.

All Grade 3 or 4 infusion reactions should be reported as an SAE if criteria are met. Infusion reactions should be graded according to NCI CTCAE v4.0.

Treatment recommendations are provided below and may be modified based on local treatment standards and guidelines as appropriate:

- **For Grade 1 symptoms:** (Mild reaction; infusion interruption not indicated; intervention not indicated) Remain at bedside and monitor subject until recovery from symptoms. The following prophylactic premedications are recommended for future infusions: diphenhydramine 50 mg (or equivalent) and/or paracetamol 325 to 1000 mg (acetaminophen) at least 30 minutes before additional Nivolumab administrations.

- **For Grade 2 symptoms**: (Moderate reaction requires therapy or infusion interruption but responds promptly to symptomatic treatment [eg, antihistamines, non-steroidal anti-inflammatory drugs, narcotics, corticosteroids, bronchodilators, IV fluids]; prophylactic medications indicated for 24 hours).

  Stop the Nivolumab infusion, begin an IV infusion of normal saline, and treat the subject with diphenhydramine 50 mg IV (or equivalent) and/or paracetamol 325 to 1000 mg (acetaminophen); remain at bedside and monitor subject until resolution of symptoms. Corticosteroid or bronchodilator therapy may also be administered as appropriate. If the infusion is interrupted, then restart the infusion at 50% of the original infusion rate when symptoms resolve; if no further complications ensue after 30 minutes, the rate may be increased to 100% of the original infusion rate. Monitor subject closely. If symptoms recur, then no further Nivolumab will be administered at that visit. Administer diphenhydramine 50 mg IV, and remain at bedside and monitor the subject until resolution of symptoms. The amount of study drug infused must be recorded on the electronic case report form (eCRF). The following prophylactic premedications are recommended for future infusions: diphenhydramine 50 mg (or equivalent) and/or paracetamol 325 to 1000 mg (acetaminophen) should be administered at least 30 minutes before additional Nivolumab administrations. If necessary, corticosteroids (recommended dose: up to 25 mg of IV hydrocortisone or equivalent) may be used.

- **For Grade 3 or Grade 4 symptoms:** (Severe reaction, Grade 3: prolonged [i.e., not rapidly responsive to symptomatic medication and/or brief interruption of infusion]; recurrence of symptoms following initial improvement; hospitalization indicated for other clinical sequelae [e.g., renal impairment, pulmonary infiltrates]). Grade 4: (life threatening; pressor or ventilatory support indicated). Immediately discontinue infusion of Nivolumab. Begin an IV infusion of normal saline and treat the subject as follows. Recommend bronchodilators, epinephrine 0.2 to 1 mg of a 1:1,000 solution for subcutaneous administration or 0.1 to 0.25 mg of a 1:10,000 solution injected slowly for IV administration, and/or diphenhydramine 50 mg IV with methylprednisolone 100 mg IV (or equivalent), as needed. Subject should be monitored until the investigator is comfortable that the symptoms will not recur. Nivolumab will be permanently discontinued. Investigators should follow their institutional guidelines for the treatment of anaphylaxis. Remain at bedside and monitor subject until recovery from symptoms. In the case of late-occurring hypersensitivity symptoms (e.g., appearance of a localized or generalized pruritis within 1 week after treatment), symptomatic treatment may be given (e.g., oral antihistamine, or corticosteroids).

6.2.2 Dose Modifications for Nivolumab/Placebo

No dose reductions or escalations (in dose or frequency) are permitted. Only dose holds are permitted per protocol.

The assessment for discontinuation of nivolumab should be made separately from the assessment made for discontinuation of ipilimumab. Although there is overlap among the discontinuation criteria, if discontinuation criteria are met for ipilimumab but not for nivolumab, treatment with nivolumab may continue if ipilimumab is discontinued.

If a participant in any of the nivolumab/ipilimumab combination arms meets criteria for discontinuation and investigator is unable to determine whether the event is related to both or one study drug, the participant should discontinue both nivolumab and ipilimumab and be taken off the treatment phase of the study.

6.2.3 Nivolumab Dose Delays

Because of the potential for clinically meaningful nivolumab-related AEs requiring early recognition and prompt intervention, management algorithms have been developed for suspected gastrointestinal, renal, pulmonary, endocrinopathies, skin, and neurological AEs, see **Appendix C**.

Tumor assessments should continue per protocol schedule, even if dosing is delayed.

Nivolumab administration should be delayed for the following:

- Any Grade ≥ 2 non-skin, drug-related AE, with the following exceptions:
    - Grade 2 drug-related fatigue or laboratory abnormalities do not require a treatment delay
- Any Grade 3 skin, drug-related AE
- Any Grade 3 drug-related laboratory abnormality, with the following exceptions:
    - Grade 3 lymphopenia or Asymptomatic amylase or lipase do not require dose delay.
    - Grade ≥3 AST/ALT or total bilirubin will require dose discontinuation
- Any AE, laboratory abnormality, or intercurrent illness which, in the judgment of the investigator, warrants delaying the dose of study medication.

Subjects who require delay of nivolumab should be re-evaluated weekly or more frequently if clinically indicated. Nivolumab dosing can be resumed on the established dosing schedule (q3 or q4 weeks) when retreatment criteria are met.

6.2.4 Criteria to Resume Nivolumab Treatment

Subjects may resume treatment with nivolumab when drug-related AE(s) resolve to Grade ≤1 or baseline value, with the following exceptions:

- Subjects may resume treatment in the presence of Grade 2 fatigue

- Subjects who have not experienced a Grade 3 drug-related skin AE may resume treatment in the presence of Grade 2 skin toxicity
- Subjects with baseline Grade 1 AST/ALT or total bilirubin who require dose delays for reasons other than a 2-grade shift in AST/ALT or total bilirubin may resume treatment in the presence of Grade 2 AST/ALT OR total bilirubin
- Subjects with combined Grade 2 AST/ALT AND total bilirubin values meeting discontinuation parameters should have treatment permanently discontinued
- Drug-related pulmonary toxicity, diarrhea, or colitis, must have resolved to baseline before treatment is resumed. Subjects with persistent Grade 1 pneumonitis after completion of a steroid taper over at least 1 month may be eligible for retreatment if discussed with and approved by Sponsor-Investigator.
- Subjects with drug-related endocrinopathies adequately controlled with only physiologic hormone replacement may resume treatment after consultation with the Sponsor-Investigator. Adrenal insufficiency requires discontinuation regardless of control with hormone replacement. If the criteria to resume treatment are met, the subject should restart treatment at the next scheduled timepoint per protocol. However, if the treatment is delayed past the next scheduled timepoint per protocol, the next scheduled timepoint will be delayed until dosing resumes.

If treatment is delayed > 6 weeks, the subject must be permanently discontinued from study therapy, except as specified in discontinuation section.

6.2.5 Nivolumab Discontinuation Criteria

Treatment should be permanently discontinued for the following:

- Any Grade 2 drug-related uveitis or eye pain or blurred vision that does not respond to topical therapy and does not improve to Grade 1 severity within the re-treatment period OR requires systemic treatment
- Any Grade 3 non-skin, drug-related adverse event lasting > 7 days or recurs, with the following exceptions for laboratory abnormalities, drug-related uveitis, pneumonitis, bronchospasm, neurologic toxicity, hypersensitivity reactions, infusion reactions, and endocrinopathies:
    - Grade 3 drug-related uveitis, pneumonitis, bronchospasm, neurologic toxicity, myocarditis, hypersensitivity reaction, or infusion reaction of any duration requires discontinuation
    - Grade 3 drug-related endocrinopathies adequately controlled with only physiologic hormone replacement do not require discontinuation. Adrenal insufficiency requires discontinuation regardless of control with hormone replacement.
    - Grade 3 drug-related laboratory abnormalities do not require treatment discontinuation except:
        - Grade 3 drug-related thrombocytopenia > 7 days or associated with bleeding requires discontinuation
        - Grade ≥ 3 drug-related AST, ALT, or Total Bilirubin requires discontinuation*
        - Concurrent AST or ALT > 3 x ULN and Total Bilirubin > 2 x ULN

*In most cases of Grade 3 AST or ALT elevation, study drug(s) will be permanently discontinued. If the investigator determines a possible favorable benefit/risk ratio that warrants continuation of study drug(s), a discussion between the investigator and the Sponsor-Investigator must occur.

- Any Grade 4 drug-related adverse event or laboratory abnormality (including but not limited to creatinine, AST, ALT, or Total Bilirubin), except for the following events which do not require discontinuation:
  - Grade 4 neutropenia ≤ 7 days
  - Grade 4 lymphopenia or leukopenia or asymptomatic amylase or lipase
  - Isolated Grade 4 electrolyte imbalances/abnormalities that are not associated with clinical sequelae and are corrected with supplementation/appropriate management within 72 hours of their onset
  - Grade 4 drug-related endocrinopathy adverse events, hyper- or hypothyroidism, or glucose intolerance, which resolve or are adequately controlled with physiologic hormone replacement (corticosteroids, thyroid hormones) or glucose-controlling agents, respectively, may not require discontinuation after discussion with and approval from the Sponsor-Investigator
- Any dosing interruption lasting > 6 weeks with the following exceptions:
  - Dosing interruptions to allow for prolonged steroid tapers to manage drug-related adverse events are allowed.
  - Dosing interruptions > 6 weeks from the previous dose that occur for non-drug-related reasons may be allowed if approved by the Sponsor-Investigator.

  Prior to re-initiating treatment in a subject with a dosing interruption lasting > 6 weeks, the Investigator must be consulted. Tumor assessments should continue as per protocol even if dosing is interrupted. Periodic study visits to assess safety and laboratory studies should also continue every 6 weeks or more frequently if clinically indicated during such dosing delays.
- Any adverse event, laboratory abnormality, or intercurrent illness which, in the judgment of the Principal Investigator, presents a substantial clinical risk to the subject with continued Nivolumab dosing.

6.3 Ipilimumab

6.3.1 Treatment of Ipilimumab Related Infusion Reactions

Since Ipilimumab contains only human immunoglobulin protein sequences, it is unlikely to be immunogenic and induce infusion or hypersensitivity reactions. However, if such a reaction were to occur, it might manifest with fever, chills, rigors, headache, rash, pruritis, arthralgias, hypo- or hypertension, bronchospasm, or other symptoms.

All Grade 3 or 4 injection reactions should be reported as an SAE if criteria are met. Injection reactions should be graded according to NCI CTCAE v4.0.

Treatment recommendations are provided below and may be modified based on local treatment standards and guidelines as appropriate:

- **For Grade 1 symptoms:** (Mild reaction; intervention not indicated)
  Remain at bedside and monitor subject until recovery from symptoms. The following prophylactic premedications are recommended for future injections: diphenhydramine 50 mg (or equivalent) and/or paracetamol 325 to 1000 mg (acetaminophen) at least 30 minutes before additional Ipilimumab administrations.
- **For Grade 2 symptoms**: (Moderate reaction requires therapy or injection interruption but responds promptly to symptomatic treatment [eg, antihistamines, non-steroidal anti-inflammatory drugs, narcotics, corticosteroids, bronchodilators, IV fluids]; prophylactic medications indicated for 24 hours).
  Stop Ipilimumab injections, begin an IV infusion of normal saline, and treat the subject with diphenhydramine 50 mg IV (or equivalent) and/or paracetamol 325 to 1000 mg (acetaminophen); remain at bedside and monitor subject until resolution of symptoms. Corticosteroid or bronchodilator therapy may also be administered as appropriate. The following prophylactic premedications are recommended for future injections: diphenhydramine 50 mg (or equivalent) and/or paracetamol 325 to 1000 mg (acetaminophen) should be administered at least 30 minutes before additional Ipilimumab administrations. If necessary, corticosteroids (recommended dose: up to 25 mg of IV hydrocortisone or equivalent) may be used.
- **For Grade 3 or Grade 4 symptoms:** (Severe reaction, Grade 3: prolonged [ie, not rapidly responsive to symptomatic medication; recurrence of symptoms following initial improvement; hospitalization indicated for other clinical sequelae [eg, renal impairment, pulmonary infiltrates]). Grade 4: (life threatening; pressor or ventilatory support indicated).
  Stop Ipilimumab injections. Begin an IV infusion of normal saline and treat the subject as follows. Recommend bronchodilators, epinephrine 0.2 to 1 mg of a 1:1,000 solution for subcutaneous administration or 0.1 to 0.25 mg of a 1:10,000 solution injected slowly for IV administration, and/or diphenhydramine 50 mg IV with methylprednisolone 100 mg IV (or equivalent), as needed. Subject should be monitored until the investigator is comfortable that the symptoms will not recur. Ipilimumab will be permanently discontinued. Investigators should follow their institutional guidelines for the treatment of anaphylaxis. Remain at bedside and monitor subject until recovery from symptoms. In the case of late-occurring hypersensitivity symptoms (eg, appearance of a localized or generalized pruritis within 1 week after treatment), symptomatic treatment may be given (eg, oral antihistamine, or corticosteroids).

6.3.2 Dose Modifications for Ipilimumab/Placebo

No dose reductions or escalations (in dose or frequency) are permitted. Only dose holds are permitted per protocol.

6.3.3 Ipilimumab Dose Delay

Because of the potential for clinically meaningful ipilimumab-related AEs requiring early recognition and prompt intervention, management algorithms have been developed for suspected gastrointestinal, renal, pulmonary, hepatic, endocrinopathies, skin, myocarditis and neurological AEs, see **Appendix C**.

Ipilimumab should be delayed for the following:

- Any Grade ≥ 2 non-skin, drug-related AE, with the following exceptions:
    - Grade 2 drug-related fatigue or laboratory abnormalities do not require a treatment delay
- Any Grade 3 skin, drug-related AE
- Any Grade 3 drug-related laboratory abnormality, with the following exceptions for lymphopenia, leukopenia, AST, ALT, total bilirubin, or asymptomatic amylase or lipase:
    - Grade 3 lymphopenia or leukopenia does not require dose delay.
    - If a subject has a baseline AST, ALT, or total bilirubin that is within normal limits, delay dosing for drug-related Grade ≥ 2 toxicity.
    - If a subject has baseline AST, ALT, or total bilirubin within the Grade 1 toxicity range, delay dosing for drug-related Grade ≥ 3 toxicity.
    - Asymptomatic amylase or lipase do not require a treatment delay
- Any AE, laboratory abnormality, or intercurrent illness which, in the judgment of the investigator, warrants delaying the dose of study medication.

6.3.4 Criteria to Resume Ipilimumab Treatment

Subjects may resume treatment with Ipilimumab when the drug-related AE(s) resolve to Grade ≤1 or baseline value, with the following exceptions:

- o Subjects may resume treatment in the presence of Grade 2 fatigue
- o Subjects who have not experienced a Grade 3 drug-related skin AE may resume treatment in the presence of Grade 2 skin toxicity
- o Subjects with baseline Grade 1 AST/ALT or total bilirubin who require dose delays for reasons other than a 2-grade shift in AST/ALT or total bilirubin may resume treatment in the presence of Grade 2 AST/ALT OR total bilirubin
- o Subjects with combined Grade 2 AST/ALT AND total bilirubin values meeting discontinuation parameters should have treatment permanently discontinued
- o Drug-related pulmonary toxicity, diarrhea, or colitis, must have resolved to baseline before treatment is resumed
- o Drug-related endocrinopathies adequately controlled with only physiologic hormone replacement may resume treatment

If the criteria to resume treatment are met, the subject should restart treatment at the next scheduled timepoint per protocol. However, if the treatment is delayed past the next scheduled timepoint per protocol, the next scheduled timepoint will be delayed until dosing resumes.

If treatment is delayed > 6 weeks, the subject must be permanently discontinued from study therapy, except as specified in discontinuation section.

6.3.5 Ipilimumab Discontinuation Criteria

Ipilimumab treatment should be permanently discontinued for the following:

- Any Grade 2 drug-related uveitis or eye pain or blurred vision that does not respond to topical therapy and does not improve to Grade 1 severity within the re-treatment period OR requires systemic treatment
- Any Grade 3 non-skin, drug-related adverse event lasting > 7 days, with the following exceptions for drug-related laboratory abnormalities, uveitis, pneumonitis, bronchospasm, diarrhea, colitis, neurologic adverse event, hypersensitivity reactions, and injection reactions
- Grade 3 drug-related uveitis, pneumonitis, bronchospasm, diarrhea, colitis, neurologic adverse event, hypersensitivity reaction, or injection reaction of any duration requires discontinuation
- Grade 3 drug-related laboratory abnormalities do not require treatment discontinuation except those noted below
- Grade 3 drug-related thrombocytopenia > 7 days or associated with bleeding requires discontinuation
- Any drug-related liver function test (LFT) abnormality that meets the following criteria require discontinuation:
    - AST or ALT > 8 x ULN
    - Total bilirubin > 5 x ULN
    - Concurrent AST or ALT > 3 x ULN and total bilirubin > 2 x ULN
- Any Grade 4 drug-related adverse event or laboratory abnormality, except for the following events which do not require discontinuation:
    - Isolated Grade 4 amylase or lipase abnormalities that are not associated with symptoms or clinical manifestations of pancreatitis and decrease to < Grade 4 within 1 week of onset.
    - Isolated Grade 4 electrolyte imbalances/abnormalities that are not associated with clinical sequelae and are corrected with supplementation/appropriate management within 72 hours of their onset
- Any dosing interruption lasting > 6 weeks with the following exceptions:
    - Dosing interruptions to allow for prolonged steroid tapers to manage drug-related adverse events are allowed. Prior to re-initiating treatment in a subject with a dosing interruption lasting > 6 weeks, the Principal Investigator must be consulted. Tumor assessments should continue as per protocol even if dosing is interrupted
    - Dosing interruptions > 6 weeks that occur for non-drug-related reasons may be allowed if approved by the Principal Investigator. Prior to re-initiating treatment in a subject with a dosing interruption lasting > 6 weeks, the Investigator must be consulted. Tumor assessments should continue as per protocol even if dosing is interrupted
- Any adverse event, laboratory abnormality, or intercurrent illness which, in the judgment of the Principal Investigator, presents a substantial clinical risk to the subject with continued Ipilimumab dosing

7. ADVERSE EVENTS: LIST AND REPORTING REQUIREMENTS

An Adverse Event (AE) is defined as any untoward medical occurrence in a patient or clinical

investigation subject administered a pharmaceutical product and which does not necessarily have to have a causal relationship with this treatment. An adverse event can therefore be any unfavorable and unintended sign (including an abnormal laboratory finding, for example), symptom, or disease temporally associated with the use of a medicinal product or protocol-specified procedure, whether or not considered related to the medicinal product or protocol-specified procedure. Any worsening (i.e., any clinically significant adverse change in frequency and/or intensity) of a preexisting condition that is temporally associated with the use of the BMS product(s), is also an adverse event.

BMS product(s) include any pharmaceutical product, biological product, device, diagnostic agent or protocol-specified procedure, whether investigational (including placebo or active comparator medication) or marketed, manufactured by, licensed by, provided by or distributed by BMS for human use.

Adverse events may occur during the course of the use of BMS product(s) in clinical trials or within the follow-up period specified by the protocol, or prescribed in clinical practice, from overdose (whether accidental or intentional), from abuse and from withdrawal.

Adverse event (AE) monitoring and reporting is a routine part of every clinical trial. The following list of reported and/or potential AEs (Section 7.1) and the characteristics of an observed AE (Section 7.2) will determine whether the event requires expedited reporting **in addition** to routine reporting.

Adverse events may also occur in screened subjects during any pre-allocation baseline period as a result of a protocol-specified intervention, including washout or discontinuation of usual therapy, diet, placebo treatment or a procedure.

All adverse events will be recorded registration through 30 days following cessation of treatment and at each examination on the Adverse Event case report forms/worksheets. The reporting timeframe for adverse events meeting any serious criteria is described in Section 7.3.2.

Adverse events will not be collected for subjects during the pre-screening period (for determination of archival tissue status) as long as that subject has not undergone any protocol-specified procedure or intervention. If the subject requires a blood draw, fresh tumor biopsy etc., the subject is first required to provide consent to the main study and AEs will be captured according to guidelines for standard AE reporting.

7.1 Expected Toxicities

A list of the adverse events and potential risks associated with nivolumab and ipilimumab in this study appears in Section 7.1.1.1 and 7.1.1.2, and will determine whether dose delays and modifications will be made or whether the event requires expedited reporting **in addition** to routine reporting. For patients who are receiving both nivolumab and ipilimumab, if one drug is held due to an AE, then both drugs will be held until criteria to resume therapy are met.

7.1.1 **Adverse Events List**

7.1.1.1 Adverse Events List for Nivolumab

<u>Very common side effects of nivolumab are: [≥ 1/10 or ≥10%]</u>

- Diarrhea
- Fatigue
- Itching
- Rash

<u>Common side effects of nivolumab include: [≥ 1/100 to < 1/10 or ≥1% to < 10%]</u>

- Abdominal pain
- Alkaline phosphatase increased: lab test result associated with liver or bone abnormalities
- Allergic reaction/hypersensitivity
- ALT increased: lab test result associated with abnormal liver function
- Amylase increased: lab test result associated with pancreas inflammation
- AST increased: lab test result associated with abnormal liver function
- Bilirubin (liver function blood test) increased
- Chills
- Constipation
- Cough
- Creatinine increased: lab test result associated with decreased kidney function
- Decreased appetite
- Dizziness or vertigo (feeling off balance which can lead to dizziness)
- Dry mouth
- Dry skin
- Fever
- Headache
- High blood pressure
- Lipase increased: lab test result associated with pancreas inflammation
- Increased blood sugar
- Inflammation of the colon
- Inflammation of the mouth
- Infusion related reaction
- Joint pain or stiffness
- Loss of color (pigment) from areas of skin
- Lung inflammation (pneumonitis - see details below)
- Musculoskeletal pain
- Nausea
- Redness (of the skin)
- Shortness of breath
- Sodium levels in the blood low

- Swelling, including face, arms, and legs
- Thyroid gland function decreased/thyroid stimulating hormone increased (lab test associated with abnormal thyroid function)
- Thyroid gland function increased
- Tingling, burning, numbness or weakness, possibly in arms, legs, hands and feet
- Upper respiratory tract infection
- Vomiting

Uncommon side effects of nivolumab include: [≥ 1/1,000 to < 1/100 or ≥0.1% to < 1%]

- Adrenal gland function decreased
- Bronchitis
- Cranial nerve disorder
- Diabetes
- Dry eye
- Hair loss
- Heart rate increased
- Heart rhythm abnormal
- Hives
- Inflammation of the eye
- Inflammation of the kidney
- Inflammation of the pancreas
- Inflammation of the pituitary gland
- Inflammation of the stomach
- Inflammation of the thyroid gland
- Liver inflammation
- Low blood pressure
- Lung infiltrates associated with infection or inflammation
- Pituitary gland function decreased
- Psoriasis: characterized by patches of abnormal, scaly skin
- Renal (kidney) failure or kidney injury
- Respiratory failure
- Vision blurred

Rare side effects of nivolumab include: [≥ 1/10,000 to < 1/1,000 or ≥0.01% to < 0.1%]

- Anaphylactic reaction (severe allergic reaction)
- Damage to the protective covering of the nerves in the brain and spinal cord
- Diabetes complications resulting in excess blood acids and diabetic coma
- Guillain-Barre syndrome, an autoimmune disorder associated with progressive muscle weakness or paralysis
- Inflammation of blood vessels
- Inflammation of the brain, potentially life-threatening or fatal
- Inflammation of the heart
- Muscle inflammation

- Myasthenic syndrome (neurologic syndrome characterized by muscle weakness) including myasthenia gravis, a nerve disease that may cause weakness of eye, face, breathing, and swallowing muscles.
- Pemphigoid: blistering of the skin or mouth caused by the immune system attacking healthy tissue
- Rhabdomyolysis: muscle fiber released into the blood stream which could damage your kidneys
- Rosacea: acne-like skin condition resulting in redness of face
- Sarcoidosis, a disease involving abnormal collections of inflammatory cells (granulomas) in organs such as lungs, skin, and lymph nodes
- Stevens Johnson syndrome: inflammatory disorder of skin and mucous membranes, resulting in blistering and shedding of skin
- Toxic epidermal necrolysis: a potentially fatal disease characterized by blistering and peeling of the top layer of skin resembling a severe burn
- Histiocytic necrotizing lymphadenitis or Kikuchi lymphadenitis: disorder of the lymph nodes which causes the lymph nodes to become enlarged, inflamed and painful, commonly affecting lymph nodes of the neck and possibly associated with fever or muscle and joint pains.
- Vogt Koyanagi Harada syndrome; a disease that affects the pigmented tissue; this may affect the eye leading to swelling, pain and/or blurred vision; the ear leading to hearing loss, ringing in the ears and /or the skin leading to loss of skin color

Lung Inflammation (pneumonitis): It is possible that nivolumab may cause inflammation of the tissues of the lung. This adverse effect has been reported in patients treated with nivolumab. While many patients with x-ray or CT abnormalities have not developed any symptoms, some patients have developed mild to severe symptoms and in rare cases, death has occurred as a result of their lung inflammation. Signs and symptoms of lung inflammation may include difficulty breathing, pain or discomfort while breathing, chest pain, cough, shortness of breath, increased rate of breathing, fever, low blood oxygen levels, or fatigue.

7.1.1.2 Adverse Events List for Ipilimumab

Ipilimumab has been most frequently studied in subjects with advanced melanoma. Other studies have been conducted in subjects with prostate cancer and lung cancer. Adverse reactions in patients with advanced melanoma treated with ipilimumab 3 mg/kg (n= 767) are listed below. These reactions are presented by system organ class and by frequency. Frequencies are defined as: very common (≥ 1/10); common (≥ 1/100 to < 1/10); uncommon (≥ 1/1,000 to < 1/100); rare (≥ 1/10,000 to < 1/1,000); very rare (< 1/10,000), not known (cannot be estimated from available post-marketing data). Within each frequency grouping, adverse reactions are presented in the order of decreasing seriousness.

**Table 4: Adverse reactions in patients with advanced melanoma treated with ipilimumab 3 mg/kg (n= 767)[a]**	
**Infections and infestations**	
Uncommon	sepsis[b], septic shock[b], urinary tract infection, respiratory tract infection
**Neoplasms benign, malignant and unspecified (including cysts and polyps)**	

Common	tumour pain
Uncommon	paraneoplastic syndrome
**Blood and lymphatic system disorders**	
Common	anaemia, lymphopenia
Uncommon	haemolytic anaemia[b], thrombocytopenia, eosinophilia, neutropenia
Not known	histiocytosis haematophagic[e]
**Immune system disorders**	
Uncommon	hypersensitivity
Very rare	anaphylactic reaction
**Endocrine disorders**	
Common	hypopituitarism (including hypophysitis)[c], hypothyroidism[c]
Uncommon	adrenal insufficiency[c], secondary adrenocortical insufficiency[d], hyperthyroidism[c], hypogonadism
Rare	autoimmune thyroiditis[d], thyroiditis[d]
**Metabolism and nutrition disorders**	
Very common	decreased appetite
Common	dehydration, hypokalemia
Uncommon	hyponatremia, alkalosis, hypophosphatemia, tumour lysis syndrome, hypocalcaemia[d]
**Psychiatric disorders**	
Common	confusional state
Uncommon	mental status changes, depression, decreased libido
**Nervous system disorders**	
Common	peripheral sensory neuropathy, dizziness, headache, lethargy
Uncommon	Guillain-Barré syndrome[b,c], meningitis (aseptic), autoimmune central neuropathy (encephalitis)[d], syncope, cranial neuropathy, brain oedema, peripheral neuropathy, ataxia, tremor, myoclonus, dysarthria
Rare	myasthenia gravis[d]
**Eye disorders**	
Common	blurred vision, eye pain
Uncommon	uveitis[c], vitreous haemorrhage, iritis[c], eye oedema[d], blepharitis[d], reduced visual acuity, foreign body sensation in eyes, conjunctivitis
Rare	Vogt-Koyanagi-Harada syndrome[e]
**Cardiac disorders**	
Uncommon	arrhythmia, atrial fibrillation
**Vascular disorders**	
Common	hypotension, flushing, hot flush
Uncommon	vasculitis, angiopathy[b], peripheral ischaemia, orthostatic hypotension
Rare	temporal arteritis[d]
**Respiratory, thoracic and mediastinal disorders**	
Common	dyspnea, cough
Uncommon	respiratory failure, acute respiratory distress syndrome[b], lung infiltration, pulmonary oedema, pneumonitis, allergic rhinitis
**Gastrointestinal disorders**	
Very common	diarrhoea[c], vomiting, nausea
Common	gastrointestinal haemorrhage, colitis[b,c], constipation, gastroesophageal reflux disease, abdominal pain, mucosal inflammation[d]
Uncommon	gastrointestinal perforation[b,c], large intestine perforation[b,c], intestinal perforation[b,c],
	peritonitisb, gastroenteritis, diverticulitis, pancreatitis, enterocolitis, gastric ulcer, large intestinal ulcer, oesophagitis, ileus[d]
Rare	proctitis[d]
**Hepatobiliary disorders**	
Common	abnormal hepatic function

Uncommon	hepatic failure[b,c], hepatitis, hepatomegaly, jaundice
**Skin and subcutaneous tissue disorders**	
Very common	rash[c], pruritus[c]
Common	dermatitis, erythema, vitiligo, urticaria, eczema[d], alopecia, night sweats, dry skin
Uncommon	toxic epidermal necrolysis[b,c], leukocytoclastic vasculitis, skin exfoliation, hair colour changes[d]
Rare	erythema multiforme[d], psoriasis[d], Drug Reaction with Eosinophilia and Systemic Symptoms (DRESS)[d]
Not known	pemphigoid
**Musculoskeletal and connective tissue disorders**	
Common	arthralgia, myalgia, musculoskeletal pain, muscle spasms
Uncommon	polymyalgia rheumatica, myositis[d], arthritis, muscular weakness[d]
Rare	polymyositis[d]
**Renal and urinary disorders**	
Uncommon	renal failure[b], glomerulonephritis[c], autoimmune nephritis[d], renal tubular acidosis, haematuria[d]
Rare	proteinuria[d]
**Reproductive system and breast disorders**	
Uncommon	amenorrhea
**General disorders and administration site conditions**	
Very common	fatigue, injection site reaction, pyrexia
Common	chills, asthenia, oedema, pain, influenza-like illness[d]
Uncommon	multi-organ failure[b,c], systemic inflammatory response syndrome[d], infusion related reaction
**Investigations**	
Common	increased alanine aminotransferase[c], increased aspartate aminotransferase[c], increased blood alkaline phosphatase[d], increased blood bilirubin, weight decreased
Uncommon	increased gamma-glutamyltransferase[d], increased blood creatinine, increased blood thyroid stimulating hormone, decreased blood cortisol, decreased blood corticotrophin, increased lipase[c], increased blood amylase[c], positive antinuclear antibody[d], decreased blood testosterone
Rare	decreased blood thyroid stimulating hormone[d], decreased thyroxine[d], abnormal blood prolactin[d]

a Frequencies are based on pooled data from 9 clinical trials investigating the ipilimumab 3 mg/kg dose in melanoma.
b Including fatal outcome.
c Additional information about these potentially inflammatory adverse reactions is provided in "Description of selected adverse reactions" and section 4.4 of IB. Data presented in those sections primarily reflect experience from a Phase 3 study, MDX010-20.
d Data outside the 9 completed clinical trials in melanoma were included in frequency determinations.
e Post-marketing event (also see section 4.4 of IB).

7.1.1.3 Risks Associated with Nivolumab Combined with Ipilimumab

Currently, there have been no new side effects seen with this combination which have not been already been identified in other trials separately evaluating nivolumab or ipilimumab. However, clinical studies to date demonstrate an increased incidence of severe side effects associated with the combination of nivolumab and ipilimumab compared with treatment with nivolumab or ipilimumab alone. Adverse reactions reported in the pooled dataset for patients treated with ipilimumab 3 mg/kg in combination with nivolumab 1 mg/kg (n = 448) are presented in below table. These reactions are presented by system organ class and by frequency. Frequencies are defined as: very common (≥ 1/10); common (≥ 1/100 to < 1/10); uncommon (≥ 1/1,000 to < 1/100); rare (≥ 1/10,000 to < 1/1,000); very rare (< 1/10,000), not known (cannot be estimated from available post-marketing data). Within each frequency grouping, adverse reactions are

presented in the order of decreasing seriousness.

Table 5: Adverse reactions with ipilimumab in combination with nivolumab	Ipilimumab 3 mg/kg in combination with nivolumab 1 mg/kg*	Ipilimumab 1 mg/kg in combination with nivolumab 3 mg/kg**
**Infections and infestations**		
Common	pneumonia, upper respiratory tract infection	pneumonia, upper respiratory tract infection, conjunctivitis
Uncommon	bronchitis	bronchitis, aseptic meningitis
**Blood and lymphatic system disorders**		
Common	eosinophilia	
Uncommon		eosinophilia
**Immune system disorders**		
Common	infusion related reaction, hypersensitivity	infusion-related reaction, hypersensitivity
Uncommon	sarcoidosis	
Not known	solid organ transplant rejection[h]	
**Endocrine disorders**		
Very common	hypothyroidism	hypothyroidism, hyperthyroidism
Common	adrenal insufficiency, hypopituitarism, hypophysitis, hyperthyroidism, thyroiditis	adrenal insufficiency[c], hypophysitis[c], thyroiditis, diabetes mellitus[c]
Uncommon	diabetic ketoacidosis[c], diabetes mellitus[c]	diabetic ketoacidosis[c], hypopituitarism
**Metabolism and nutrition disorders**		
Very common	decreased appetite	decreased appetite
Common	dehydration	dehydration
Uncommon		metabolic acidosis
Not known	tumour lysis syndrome[i]	
**Hepatobiliary disorders**		
Common	hepatitis[c]	hepatitis[c]
**Nervous system disorders**		
Very common	headache	
Common	peripheral neuropathy, dizziness	headache, peripheral neuropathy, dizziness
Uncommon	Guillain-Barré syndrome, polyneuropathy, neuritis, peroneal nerve palsy, autoimmune neuropathy (including facial and abducens nerve paresis), encephalitis[c]	polyneuropathy, autoimmune neuropathy (including facial and abducens nerve paresis), myasthenia gravis[c]
**Eye disorders**		
Common	uveitis, blurred vision	blurred vision
Uncommon		uveitis
Not known	Vogt-Koyanagi-Harada syndrome[h]	
**Cardiac disorders**		
Common	tachycardia	tachycardia
Uncommon	arrhythmia (including ventricular arrhythmia)[a,d], atrial fibrillation, myocarditis[a,f]	arrhythmia (including ventricular arrhythmia), myocarditis[c]
Not known	pericardial disorders[j]	
**Vascular disorders**		
Common	hypertension	hypertension

**Respiratory, thoracic and mediastinal disorders**		
Very common	dyspnoea	
Common	pneumonitisa,c, pulmonary embolisma, cough	pneumonitis, dyspnoea, pleural effusion, cough
Uncommon	pleural effusion	
**Gastrointestinal disorders**		
Very common	colitis[a], diarrhoea, vomiting, nausea, abdominal pain	diarrhoea, vomiting, nausea
Common	stomatitis, pancreatitis, constipation, dry mouth	colitis, stomatitis, pancreatitis, abdominal pain, constipation, dry mouth
Uncommon	intestinal perforation[a], gastritis, duodenitis	gastritis
**Skin and subcutaneous tissue disorders**		
Very common	rash[e], pruritus	rash[e], pruritus
Common	vitiligo, dry skin, erythema, alopecia, urticaria	dry skin, erythema, urticaria
Uncommon	psoriasis	Stevens-Johnson syndrome, vitiligo, erythema multiforme, alopecia, psoriasis
Rare	toxic epidermal necrolysis[a,f], Stevens-Johnson syndrome[f]	
**Musculoskeletal and connective tissue disorders**		
Very common	arthralgia	musculoskeletal pain[g], arthralgia
Common	musculoskeletal pain[g]	arthritis, muscle spasms, muscular weakness
Uncommon	spondyloarthropathy, Sjogren's syndrome, arthritis, myopathy, myositis (including polymyositis)[a,e], rhabdomyolysis[a,f]	polymyalgia rheumatica, myositis (including polymyositis), rhabdomyolysis
**Renal and urinary disorders**		
Common	renal failure (including acute kidney injury)[a,c]	renal failure (including acute kidney injury)[c]
Uncommon	tubulointerstitial nephritis	tubulointerstitial nephritis
**General disorders and administration site conditions**		
Very common	fatigue, pyrexia	fatigue, pyrexia
Common	oedema (including peripheral oedema), pain	oedema (including peripheral oedema), pain, chest pain, chills
Uncommon	chest pain	
**Investigations[b]**		
Very common	increased AST, increased ALT, increased total bilirubin, increased alkaline phosphatase, increased lipase, increased amylase, increased creatinine, hyperglycaemia[c], hypoglycaemia, lymphopaenia, leucopoenia, neutropaenia, thrombocytopaenia, anaemia, hypocalcaemia, hyperkalaemia, hypokalaemia, hypomagnesaemia, hyponatraemia	increased AST, increased ALT, increased total bilirubin, increased alkaline phosphatase, increased lipase, increased amylase, increased creatinine, hyperglycaemia[c], hypoglycaemia, lymphopaenia, leucopoenia, neutropaenia[c], thrombocytopaenia, anaemia, hypercalcaemia, hypocalcaemia, hyperkalaemia, hypokalaemia, hypomagnesaemia, hyponatraemia
Common	hypercalcaemia, hypermagnesaemia, hypernatraemia, weight decreased	hypermagnesaemia, hypernatraemia, weight decreased

* ipilimumab in combination with nivolumab for the first 4 doses then followed by nivolumab monotherapy in melanoma.

** ipilimumab in combination with nivolumab for the first 4 doses then followed by nivolumab

monotherapy in RCC.

[a] Fatal cases have been reported in completed or ongoing clinical studies

[b] Frequencies of laboratory terms reflect the proportion of patients who experienced worsening from baseline in laboratory measurements. See "Description of selected adverse reactions; laboratory abnormalities" below.

[c] Life-threatening cases have been reported in completed or ongoing clinical studies.

[d] The frequency of adverse events in the cardiac disorders system organ class regardless of causality was higher in the nivolumab group than in the chemotherapy group in post-CTLA4/BRAF inhibitor metastatic melanoma population. Incidence rates per 100 person-years of exposure were 9.3 vs. 0; serious cardiac events were reported by 4.9% patients in the nivolumab group vs. 0 in the investigator´s choice group.

The frequency of cardiac adverse events was lower in the nivolumab group than in the dacarbazine group in the metastatic melanoma without prior treatment population. All were considered not related to nivolumab by investigators except arrhythmia (atrial fibrillation, tachycardia and ventricular arrhythmia).

[e] Rash is a composite term which includes maculopapular rash, rash erythematous, rash pruritic, rash follicular, rash macular, rash morbilliform, rash papular, rash pustular, rash papulosquamous, rash vesicular, rash generalised, exfoliative rash, dermatitis, dermatitis acneiform, dermatitis allergic, dermatitis atopic, dermatitis bullous, dermatitis exfoliative, dermatitis psoriasiform, drug eruption and pemphigoid.

[f] Reported also in studies outside the pooled dataset. The frequency is based on the program-wide exposure.

[g] Musculoskeletal pain is a composite term which includes back pain, bone pain, musculoskeletal chest pain, musculoskeletal discomfort, myalgia, neck pain, pain in extremity, and spinal pain.

[h] Post-marketing event

[i] Reported in clinical studies and in the post-marketing setting.

[j] Pericardial disorders is a composite term which includes pericarditis, pericardial effusion, cardiac tamponade, and Dressler's syndrome.

7.2 Adverse Event Characteristics

- **CTCAE term (AE description) and grade:** The descriptions and grading scales found in the revised NCI Common Terminology Criteria for Adverse Events (CTCAE v4.0) will be utilized for AE reporting. All appropriate treatment areas should have access to a copy of the CTCAE v4.0. A copy of the CTCAE v4.0 can be downloaded from: http://ctep.cancer.gov/protocolDevelopment/electronic_applications/ctc.htm

An investigator who is a qualified physician will evaluate all adverse events according to the NCI Common Terminology for Adverse Events version 4.0 (CTCAE v4.0). Any adverse event which changes CTCAE grade over the course of a given episode will have each change of grade recorded on the adverse event case report form.

- **For expedited reporting purposes only:**
  - AEs for the agent(s) that are listed above should be reported only if the adverse

event varies in nature, intensity or frequency from the expected toxicity information which is provided.

All adverse events regardless of CTCAE grade must also be evaluated for seriousness as defined in Section 7.3.1 and Table 6. In addition, attribution of AEs must be classified based on the following definitions:

- **Attribution** of the AE:
  Definite – The AE *is clearly related* to the study treatment.
  Probable – The AE *is likely related* to the study treatment.
  Possible – The AE *may be related* to the study treatment.
  Unlikely – The AE *is doubtfully related* to the study treatment.
  Unrelated – The AE *is clearly NOT related* to the study treatment.

Table 6. Evaluating Adverse Events

Appropriately delegated study team members will evaluate all adverse events as to:

**V4.0 CTCAE Grading**	**Grade 1**	**Mild; asymptomatic or mid symptoms; clinical or diagnostic observations only; intervention not indicated.**
	**Grade 2**	**Moderate; minimal, local or noninvasive intervention indicated; limiting age-appropriate instrumental ADL.**
	**Grade 3**	**Severe or medically significant but not immediately life-threatening; hospitalization or prolongation or hospitalization indicated; disabling; limiting self-care ADL.**
	**Grade 4**	**Life threatening consequences; urgent intervention indicated.**
	**Grade 5**	**Death related to AE**
**Seriousness**	A serious adverse event is any adverse event occurring at any dose or during any use of BMS product that:	
	†**Results in death**; or	
	†**Is life threatening; or** places the subject, in the view of the investigator, at immediate risk of death from the event as it occurred (Note: This does not include an adverse event that, had it occurred in a more severe form, might have caused death.); or	
	†**Results in a persistent or significant disability/incapacity** (substantial disruption of one's ability to conduct normal life functions); or	
	†**Results in or prolongs an existing inpatient hospitalization** (hospitalization is defined as an inpatient admission, regardless of length of stay, even if the hospitalization is a precautionary measure for continued observation. (Note: Hospitalization for an elective procedure to treat a pre-existing condition that has not worsened is not a serious adverse event. A pre-existing condition is a clinical condition that is diagnosed prior to the use of a BMS product and is documented in the patient's medical history.); or	
	†**Is a congenital anomaly/birth defect** (in offspring of subject taking the product regardless of time to diagnosis); or	
	**Is a** new **cancer;** (that is not a condition of the study) **(**although not serious per ICH definition, is reportable to the Sponsor within 24 hours and to BMS within 2 working days to meet certain local requirements); **or**	
	**Is an overdose** (whether accidental or intentional). Any adverse event associated with an overdose is considered a serious adverse event for collection purposes. An overdose that is not associated with an adverse event is considered a non-serious event of clinical interest and must be reported within 24 hours to the Sponsor and to BMS within 2 working days.	
	**Other important medical events** that may not result in death, not be life threatening, or not require hospitalization may be considered a serious adverse event when, based upon appropriate medical judgment, the event may jeopardize the subject and may require medical or surgical intervention to prevent one of the outcomes listed previously (designated above by a †).	
**Duration**	Record the start and stop dates of the adverse event. If less than 1 day, indicate the appropriate length of time and units	
**Action taken**	Did the adverse event cause the BMS product to be discontinued?	
**Relationship to BMS Product**	Did BMS product cause the adverse event? The determination of the likelihood that BMS product caused the adverse event will be provided by an investigator who is a qualified physician. The investigator's signed/dated initials on the source document or worksheet that supports the causality noted on the AE form, ensures that a medically qualified assessment of causality was done. This initialed document must be retained for the required regulatory time frame. The criteria below are intended as reference guidelines to assist the investigator in assessing the likelihood of a relationship between the test drug and the adverse event based upon the available information.<br>**The following components are to be used to assess the relationship between BMS product and the AE**; the greater the correlation with the components and their respective elements (in number and/or intensity), the more likely BMS product caused the adverse event (AE):	
	**Exposure**	Is there evidence that the subject was actually exposed to BMS product such as: reliable history, acceptable compliance assessment (pill count, diary, etc.), expected pharmacologic effect, or measurement of drug/metabolite in bodily specimen?
	**Time Course**	Did the AE follow in a reasonable temporal sequence from administration of BMS product? Is the time of onset of the AE compatible with a drug-induced effect (applies to trials with investigational medicinal product)?
	**Likely Cause**	Is the AE not reasonably explained by another etiology such as underlying disease, other drug(s)/vaccine(s), or other host or environmental factors

**Relationship to BMS product (continued)**	**The following components are to be used to assess the relationship between the test drug and the AE: (continued)**	
	**Dechallenge**	Was BMS product discontinued or dose/exposure/frequency reduced?<br>If yes, did the AE resolve or improve?<br>If yes, this is a positive dechallenge. If no, this is a negative dechallenge.<br>(Note: This criterion is not applicable if: (1) the AE resulted in death or permanent disability; (2) the AE resolved/improved despite continuation of the BMS product; or (3) the trial is a single-dose drug trial); or (4) BMS product(s) is/are only used one time.)
	**Rechallenge**	Was the subject re-exposed to BMS product in this study?<br>If yes, did the AE recur or worsen?<br>If yes, this is a positive rechallenge. If no, this is a negative rechallenge.<br>(Note: This criterion is not applicable if: (1) the initial AE resulted in death or permanent disability, or (2) the trial is a single-dose drug trial); or (3) BMS product(s) is/are used only one time).<br>NOTE: IF A RECHALLENGE IS PLANNED FOR AN ADVERSE EVENT WHICH WAS SERIOUS AND WHICH MAY HAVE BEEN CAUSED BY BMS PRODUCT, OR IF REEXPOSURE TO BMS PRODUCT POSES ADDITIONAL POTENTIAL SIGNIFICANT RISK TO THE SUBJECT, THEN THE RECHALLENGE MUST BE APPROVED IN ADVANCE BY THE U.S. CLINICAL MONITOR AS PER DOSE MODIFICATION GUIDELINES IN THE PROTOCOL.
	**Consistency with Trial Treatment Profile**	Is the clinical/pathological presentation of the AE consistent with previous knowledge regarding the BMS product or drug class pharmacology or toxicology?
The assessment of relationship will be reported on the case report forms /worksheets by an investigator who is a qualified physician according to his/her best clinical judgment, including consideration of the above elements.		
**Record one of the following**		**Use the following scale of criteria as guidance (not all criteria must be present to be indicative of a BMS product relationship).**
**- Yes, there is a reasonable possibility of BMS product relationship.**		There is evidence of exposure to BMS product. The temporal sequence of the AE onset relative to the administration of BMS product is reasonable. The AE is more likely explained by BMS product than by another cause.
**- No, there is not a reasonable possibility BMS product relationship**		Subject did not receive BMS product OR temporal sequence of the AE onset relative to administration of BMS product is not reasonable OR there is another obvious cause of the AE. (Also entered for a subject with overdose without an associated AE.)

7.3 Adverse Event Reporting

In the event of an unanticipated problem or life-threatening complications treating investigators must immediately notify the PI.

Progression of the cancer under study is not considered an adverse event unless it results in hospitalization or death.

Changes resulting from normal growth and development that do not vary significantly in frequency or severity from expected levels are not to be considered adverse events. Examples of this may include, but are not limited to, onset of menses or menopause occurring at a physiologically appropriate time.

7.3.1 Serious Adverse Events

A serious adverse event (SAE) is any adverse event, occurring at any dose and regardless of causality that:

- Results in death
- Is life threatening or places the subject, in the view of the investigator, at immediate risk of death from the event as it occurred (Note: This does not include an adverse event that, had it occurred in a more severe form, might have caused death.)
- Results in persistent or significant disability/incapacity (substantial disruption of one's ability to conduct normal life functions);
- Results in or prolongs an existing inpatient hospitalization (hospitalization is defined as an inpatient admission, regardless of length of stay, even if the hospitalization is a precautionary measure for continued observation.
    - Note: Hospitalization for an elective procedure to treat a pre-existing condition that has not worsened is not a serious adverse event. A pre-existing condition is a clinical condition that is diagnosed prior to the use of a BMS product and is documented in the patient's medical history.
- Is a congenital anomaly/birth defect
- Is a new cancer (that is not a condition of the study)
- Is associated with an overdose
- Is another important medical event that may not result in death, not be life threatening, or not require hospitalization may be considered a serious adverse event when, based upon appropriate medical judgment, the event may jeopardize the subject and may require medical or surgical intervention to prevent one of the outcomes listed previously

7.3.2 Expedited Reporting Guidelines

Investigators must report any serious adverse event, or follow up to a serious adverse event, including death due to any cause other than progression of the cancer under study that occurs to any subject from the time the initiation of study treatment through 100 days following cessation of treatment (or the initiation of new anti-cancer therapy, whichever is earlier) whether or not related to BMS product, to the DFCI Coordinating Center, Sponsor-Investigator, and BMS Global Safety within 24 hours of learning of the event on the local institutional SAE form.

Additionally, any serious adverse event, considered by an investigator who is a qualified physician to be related to BMS product that is brought to the attention of the investigator at any time outside of the time period specified in the previous paragraph also must be reported immediately to the DFCI Coordinating Center, Sponsor-Investigator, and to BMS Global Safety.

All subjects with serious adverse events must be followed up for outcome.

A copy of all 15 Day Reports and Annual Progress Reports is submitted as required by FDA, European Union (EU), Pharmaceutical and Medical Devices agency (PMDA) or other local regulators. Investigators will cross reference this submission according to local regulations to the BMS Investigational Compound Number (IND, CSA, etc.) at the time of submission. Additionally, investigators will submit a copy of these reports to BMS Global Safety at the time of submission to FDA.

All participating sites will report AEs to the Sponsor-Investigator per DF/HCC requirements, and the IRB of record for each site as applicable per IRB policies. DF/HCC institutions will report AEs directly to the DFCI Office for Human Research Studies (OHRS) per the DFCI IRB reporting policy. External Sites will report AEs to their respective IRB in accordance with local policy and will submit a copy of the institutional AE report to the Sponsor-Investigator. The Sponsor-Investigator will submit SAE reports from outside institutions to the DFCI OHRS according to DFCI IRB policies and procedures in reporting adverse events.

Table 7 below indicates which adverse events must be reported to the DF/HCC Sponsor-Investigator and/or BMS Global Safety.

Table 7: Expedited Reporting Guidelines

Adverse Event Characteristics				Expedited Reporting Guidelines [a]	
	Toxicity Grade	Known Correlation [b]	Attribution to Study Treatment(s)	BMS Global Safety	Sponsor-Investigator (Patrick Wen, MD) at the DFCI Coordinating Center
Serious Event [c]	Any	Any (Expected or Unexpected)	Any	within 24 hours	within 24 hours
Non-Serious	Pregnancy, Lactation [d] or Overdose [e]	Any (Expected or Unexpected)	Any	within 24 hours	within 24 hours
Non-Serious	Grade 4	Unexpected	Any	Not Required	5 calendar days
Non-Serious	Grade 2 or 3	Unexpected	Possible, Probable, or Definite	Not Required	5 calendar days
Non-Serious	Event of Clinical Interest [f]	Any (Expected or Unexpected	Any	within 7 business days	within 7 business days

a) If participating study team does not become aware of a reportable adverse event immediately, the event should be reported within the required timeframe after learning of the event and document the time of first awareness of the adverse event as the when the event became reportable. See Section 7.3.3 for reporting procedures.
b) Expected adverse events are those previously identified as resulting from administration of the agent. For the purposes of this study, an adverse event is considered expected when it appears in the Expected Toxicities lists (Section 7.1) which are derived from the Investigator's Brochure.
c) Serious as defined in Section 7.3.1
d) See Section 7.3.6 for details on reporting lactation and pregnancy
e) See Section 7.3.5 for details on reporting overdose
f) See Section 7.3.4 for details on Events of Clinical Interest

7.3.3 Expedited Reporting Procedures

1. Complete the following:
   - MedWatch 3500A (downloadable at http://www.fda.gov/medwatch/getforms.htm)
   - Reportable AE Coversheet (found in Appendix D)

2. To report an AE to the Sponsor-Investigator via the DFCI Coordinating Center:
   - e-mail MedWatch and Coversheet to NeuroOnc_SAE@dfci.harvard.edu with the subject "20-494: Nivo/Ipi for GBM - SAE" All reports received at this account are

forwarded immediately to the Sponsor-Investigator (Dr. Patrick Wen) and to the DFCI Coordinating Center.
- submit documentation from local IRB submission for this event, if applicable

3. To report an AE to BMS Global Safety:
   - submit MedWatch and Coversheet to BMS Global Safety via e-mail at Worldwide.Safety@BMS.com or via SAE Facsimile Number: +1 609-818-3804

Note: It is preferred that MedWatch and Coversheet be e-mailed simultaneously to BMS and DFCI Coordinating Center/Sponsor-Investigator.

7.3.4 **Events of Clinical Interest**

Non-serious events of clinical interest must be reported to the DFCI Coordinating Center, Sponsor-Investigator, and BMS (Worldwide.Safety@BMS.com; SAE Facsimile Number: +1 609-818-3804) within 7 business days to comply with regulatory requirements. The DFCI Coordinating Center should be copied on the submission (NeuroOnc_SAE@dfci.harvard.edu).

Non-serious AEs of clinical interest should be followed to resolution or stabilization, or reported as SAEs if they become serious.

Follow-up is also required for non-serious AEs that cause interruption or discontinuation of the BMS product associated with this study and for those present at the end of the study, as appropriate.

Events of clinical interest for this trial include:

- Pneumonitis
- Colitis (Diarrhea)
- Hepatitis
- Nephritis/renal dysfunction
- Rash
- Endocrine [adrenal insufficiency, hypothyroidism/thyroiditis, hyperthyroidism, diabetes mellitus, and hypophysitis]

7.3.5 **Overdose**

An overdose is defined as the accidental or intentional administration of any dose of a product that is considered both excessive and medically important. No specific information is available on the treatment of overdose of nivolumab or ipilimumab. Appropriate supportive treatment should be provided if clinically indicated. In the event of overdose, the subject should be observed closely for signs of toxicity. Appropriate supportive treatment should be provided if clinically indicated.

If an adverse event(s) is associated with (“results from”) the overdose of a BMS product, the adverse event(s) is reported as a serious adverse event, even if no other seriousness criteria are met.

If a dose of BMS’s product meeting the protocol definition of overdose is taken without any associated clinical symptoms or abnormal laboratory results, the overdose is reported as a non-serious Event of Clinical Interest (ECI), using the terminology “accidental or intentional overdose without adverse effect.”

All reports of overdose with and without an adverse event must be reported within 24 hours to the DFCI Coordinating Center and Sponsor-Investigator and within 24 hours to BMS Global Safety (Worldwide.Safety@BMS.com; SAE Facsimile Number: +1 609-818-3804). The DFCI Coordinating Center should be copied on the submission (NeuroOnc_SAE@dfci.harvard.edu).

7.3.6 Reporting of Pregnancy and Lactation to the Sponsor and to BMS

Although pregnancy and lactation are not considered adverse events, it is the responsibility of investigators or their designees to report any pregnancy or lactation in a subject (spontaneously reported to them) that occurs during the trial. The study investigator will make every effort to obtain permission to follow the outcome of the pregnancy and report the condition of the fetus or newborn to the Sponsor and to BMS. If a male subject impregnates his female partner the study personnel at the site must be informed immediately and the pregnancy reported to the Sponsor and to BMS.

Pregnancies and lactations that occur after the consent form is signed but before treatment allocation/randomization must be reported by the investigator if they cause the subject to be excluded from the trial, or are the result of a protocol-specified intervention, including but not limited to washout or discontinuation of usual therapy, diet, placebo treatment or a procedure.

Pregnancies and lactations that occur from the time of treatment allocation/randomization through 120 days following cessation of treatment, or 30 days following cessation of treatment if the subject initiates new anticancer therapy, whichever is earlier, must be reported by the investigator. The site will contact the subject at least monthly and document the subject's status until completion/termination of the pregnancy. Pregnancy outcomes of spontaneous abortion, missed abortion, benign hydatidiform mole, blighted ovum, fetal death, intrauterine death, miscarriage and stillbirth must be reported as serious events (Important Medical Events). If the pregnancy continues to term, the outcome (health of infant) must also be reported.

Such events must be reported within 1 working day hours to the DFCI Coordinating Center and Sponsor-Investigator and within 24 hours to BMS Global Safety (Worldwide.Safety@BMS.com; SAE Facsimile Number: +1 609-818-3804). The DFCI Coordinating Center should be copied on the submission (NeuroOnc_SAE@dfci.harvard.edu).

7.4 Reporting to the Food and Drug Administration (FDA)

The Sponsor-Investigator will be responsible for all communications with the FDA. The Sponsor-Investigator will report to the FDA, regardless of the site of occurrence, any serious adverse event that meets the FDA's criteria for expedited reporting following the reporting requirements and timelines set by the FDA.

7.5 Reporting to Hospital Risk Management

Participating investigators will report to their local Risk Management office any participant safety reports, sentinel events, or unanticipated problems that require reporting per institutional policy.

7.6 Routine Adverse Event Reporting

All Adverse Events **must** be reported in routine study data submissions to the PI on the toxicity

case report forms. **AEs reported through expedited processes (e.g., reported to the IRB, FDA, etc.) must also be reported in routine study data submissions.**

8. PHARMACEUTICAL INFORMATION

A list of the adverse events and potential risks associated with the investigational or other agents administered in this study can be found in Section 7.1.

Study drug includes both Investigational Product (IP) and Non-investigational Product (Non-IP) and can consist of the following:

Table 8: Study Drugs

Product Description / Class and Dosage Form	Potency	IP/ Non-IP	Blinded or Open Label [a]	Packaging	Storage
Nivolumab Solution for Injection	100 mg (10 mg/ml)	IP	Open Label	10 mL per vial Clear to opalescent colorless to pale yellow liquid. May contain particles	2 to 8 °C. Protect from light and freezing
Ipilimumab Solution for Injection	50 mg (5 mg/ml)	IP	Open Label	10 mL per vial Clear to opalescent colorless to pale yellow liquid. May contain particles	2 to 8 °C. Protect from light and freezing
Placebo	N/A	IP	N/A	Prepared by local IP Pharmacy	Per institution

[a] The term “open-label” refers to the medication as it is upon receipt at the pharmacy. The neoadjuvant treatment period of the trial will be blinded, nivolumab/placebo and ipilimumab/placebo will be blinded to site investigators, site staff and patients. Site IP pharmacist will not be blinded.

NOTE: These products may be obtained by the investigational sites as local commercial product in certain countries if allowed by local regulations. In these cases, products may be a different pack size/potency than listed in the table. These products should be prepared/stored/administered in accordance with the package insert or summary of product characteristics

Investigational Product (IP)

An investigational product, also known as investigational medicinal product in some regions, is defined a pharmaceutical form of an active substance or placebo being tested or used as a reference in a clinical study, including products already with a marketing authorization but used or assembled (formulated or packaged) differently than the authorized form, or used for an unauthorized indication, or when used to gain further information about the authorized form.

The investigational product should be stored in a secure area according to local regulations. It is the responsibility of the investigator to ensure that investigational product is only dispensed to study subjects. The investigational product must be dispensed only from official study sites by authorized personnel according to local regulations.

In this protocol, investigational products are nivolumab, ipilimumab, nivolumab or ipilimumab placebo (0.9% sodium chloride for injection or 5% dextrose for injection).

Infusion-related supplies (e.g., IV bags, in-line filters (0.2-1.2 micron), 0.9% NaCl or 5% Dextrose solution) will not be supplied by the Sponsor and should be purchased locally if permitted by local Regulations.

Non-investigational Product (Non-IP)

Other medications used as support or escape medication for preventative, diagnostic, or therapeutic reasons, as components of the standard of care for a given diagnosis, may be considered as non-investigational products. No investigational or commercial agents or therapies other than those described below may be administered with the intent to treat the participant's malignancy.

8.1 Nivolumab

Additional detailed information on nivolumab is available in the Investigator's Brochure and/or package insert.

Nivolumab is a fully human monoclonal immunoglobulin G4 (IgG4-S228P) antibody that targets the programmed death (PD-1) cell surface membrane receptor.

The investigator shall take responsibility for and shall take all steps to maintain appropriate records and ensure appropriate supply, storage, handling, distribution and usage of investigational product in accordance with the protocol and any applicable laws and regulations.

8.1.1 **Description**

Nivolumab is a fully human monoclonal immunoglobulin G4 (IgG4-S228P) antibody that targets the programmed death (PD-1) cell surface membrane receptor.

8.1.2 **Form**

Nivolumab Injection Drug product is a sterile, non-pyrogenic, single-use, isotonic aqueous solution formulated at 10 mg/ml. The solution is a clear to opalescent, colorless to pale liquid and may contain particles. It is supplied in 100mg, 10mg/ml vials stoppered with butyl stoppers and sealed with aluminum seals.

8.1.3 **Storage and Stability**

Nivolumab must be stored at 2º-8ºC (36º-46ºF) and protected from light and freezing. IV bags containing undiluted and diluted solutions of Nivolumab prepared for dosing may be stored up to 20 hours in a refrigerator at 2°-8°C (36°-46°F) and used within 8 hours at room temperature and under room light. The maximum 8-hour period under room temperature and room light conditions

for undiluted and diluted solutions of Nivolumab injection in the IV bag should be inclusive of the product administration period.

8.1.4 Compatibility

Nivolumab will not be mixed with any other study agents.

8.1.5 Handling

There are no specific handling instructions for Nivolumab.

8.1.6 Availability

Nivolumab is an FDA-approved drug for the treatment of multiple cancer indications including metastatic melanoma, non-small cell lung cancer, small cell lung cancer, renal cell carcinoma, urothelial carcinoma. Nivolumab will be supplied by Bristol Myers Squibb to the participating institution's pharmacy.

8.1.7 Preparation

Dilute nivolumab with either 0.9% Sodium Chloride injection, USP or 5% Dextrose injection, USP to prepare an infusion with a final concentration ranging from 1 to 10 mg/ml. Mix diluted solution by gentle infusion. Do not shake. The maximum allowable volume of final infusion is 160 ml.

8.1.8 Administration

Nivolumab injection is to be administered as an IV infusion, using a volumetric pump with a 0.2 / 0.22 micron ore size, low-protein binding polyethersulfone membrane in-line filter at the protocol-specified doses.

8.1.9 Ordering

Nivolumab will be obtained from Bristol Myers Squibb (BMS) as an Investigator Initiated Study. Nivolumab can be ordered from BMS by providing a completed Drug Shipment Form.

8.1.10 Accountability

The investigator, or a responsible party designated by the investigator, should maintain a careful record of the inventory and disposition of the agent using the NCI Drug Accountability Record Form (DARF) or another comparable drug accountability form.

8.1.11 Destruction and Return

At the end of the study, unused supplies of Nivolumab should be destroyed according to institutional policies. Destruction will be documented in the Drug Accountability Record Form.

8.2 Ipilimumab

8.2.1 Description

Ipilimumab is a fully human monoclonal immunoglobulin G1κ antibody that targets the human cytotoxic T lymphocyte antigen 4 (CTLA-4) cell surface membrane receptor.

Additional detailed information can be found in the Investigator's Brochure and/or package insert.

8.2.2 Form

Ipilimumab Injection Drug product is a sterile, non-pyrogenic, single-use, isotonic aqueous solution formulated at 5 mg/ml (0.9% sodium chloride injection; USP, or 5% dextrose injection).

The solution is a clear to slightly opalescent colorless to pale yellow liquid and may contain a small amount of translucent-to-white particles.

8.2.3 Storage and Stability

Ipilimumab must be stored at 2º-8ºC (36º-46ºF) and protected from light and freezing. Partially used vials should be discarded after single use.

8.2.4 Compatibility

Ipilimumab will not be mixed with any other study agents.

8.2.5 Handling

Vials containing Ipilimumab should not be shaken and should be discarded if cloudy.

8.2.6 Availability

Ipilimumab is FDA approved for metastatic melanoma, renal cell carcinoma and colorectal cancer. In this study, it will be an investigational product and will be supplied by Bristol Myers Squibb to the participating institution's pharmacy.

8.2.7 Preparation

Ipilimumab injection, (5 mg/mL), can be used for intravenous (IV) administration without dilution after transferring to a polyvinyl chloride (PVC), non-PVC/non-di(2-ethylhexyl)phthalate (DEHP), or glass container and is stable for 24 hours at 2°C to 8°C or room temperature/room light. Ipilimumab injection may be diluted in 0.9% sodium chloride injection or 5% dextrose injection to concentrations between 1 mg/mL and 2 mg/mL and stored in PVC, non-PVC/non-DEHP, or glass containers for up to 24 hours at 2°C to 8°C or room temperature/room light. The product may be infused using a volumetric pump at the protocol specific dose(s) and rate(s) through a PVC IV solution infusion set with an in-line, sterile, nonpyrogenic, low protein binding filter (pore size of 0.2 µm to 1.2 µm). Ipilimumab injection must not be administered as an IV push or bolus injection. Care must be taken to assure sterility of the prepared solutions since the drug product does not contain any antimicrobial preservatives or bacteriostatic agents.

- Do not shake product.

- Inspect parenteral drug products visually for particulate matter and discoloration prior to administration. Discard vial if solution is cloudy, there is pronounced discoloration (solution may have pale-yellow color), or there is foreign particulate matter other than translucent-to white, amorphous particles.
- Allow the vials to stand at room temperature for approximately 5 minutes prior to preparation of infusion.
- Withdraw the required volume of ipilimumab and transfer into an intravenous bag.
- Dilute with 0.9% Sodium Chloride Injection, USP or 5% Dextrose Injection, USP to prepare a diluted solution with a final concentration ranging from 1 mg/mL to 2 mg/mL. Mix diluted solution by gentle inversion.
- Store the diluted solution for no more than 24 hours under refrigeration (2°C to 8°C, 36°F to 46°F) or at room temperature (20°C to 25°C, 68°F to 77°F).
- Discard partially used vials or empty vials of ipilimumab.

8.2.8 **Administration**

- Do not mix ipilimumab with, or administer as an infusion with, other medicinal products.
- Flush the intravenous line with 0.9% Sodium Chloride Injection, USP or 5% Dextrose Injection, USP after each dose.
- Administer diluted solution through an intravenous line containing a sterile, non-pyrogenic, low-protein-binding in-line filter.

When administered in combination with nivolumab, infuse nivolumab first followed by ipilimumab on the same day. Use separate infusion bags and filters for each infusion.

8.2.9 **Ordering**

Ipilimumab will be obtained from Bristol Myers Squibb (BMS) as an Investigator Initiated Study. Ipilimumab can be ordered from BMS by providing a completed Drug Shipment Form.

8.2.10 **Accountability**

The investigator, or a responsible party designated by the investigator, should maintain a careful record of the inventory and disposition of the agent using the NCI Drug Accountability Record Form (DARF) or another comparable drug accountability form. (See the NCI Investigator's Handbook for Procedures for Drug Accountability and Storage.)

8.2.11 **Destruction and Return**

At the end of the study, unused supplies of ipilimumab should be destroyed according to institutional policies. Destruction will be documented in the Drug Accountability Record Form.

8.3 ^{89}Zr-Df-IAB22M2C

8.3.1 **Description**

^{89}Zr-Df-IAB22M2C is an ~80 kDa minibody (Mb) molecular weight with high affinity to CD8 glycoprotein (binding EC_{50} = 0.4nM) that is conjugated with desferrioxamine (Df) and radiolabeled with ^{89}Zr for imaging CD8+ cells in humans.

Additional detailed information can be found in the Investigator's Brochure.

8.3.2 Storage and Handling

^{89}Zr-Df-IAB22M2C will be stored in a secure refrigerator at 2°C to 8°C (36°CF to 46°F), until one hour before the time of infusion. The dose of ^{89}Zr-Df-IAB22M2C should be dispensed and administered by the expiration time listed on the label.

Handling of ^{89}Zr-Df-IAB22M2C should be in keeping with the ALARA principles in order to minimize radiation dose to staff.

8.3.3 Preparation and Administration

A dose of 1.0 (±20%) mCi of ^{89}Zr-Df-IAB22M2C between with 1.5mg of API will be administered intravenously over 5-10 minutes, prior to the start of neoadjuvant treatment and approximately 3 days prior to surgery (allowed window: +/-2 days). Participants will undergo both whole body and dedicated brain PET scan imaging approximately 24 hours after each ^{89}Zr-Df-IAB22M2C infusion.

The dose will be delivered by a central manufacturing facility to the investigational site's radiopharmacy in the Nuclear Medicine Department. Upon receipt, qualified personnel will ensure that the study drug is delivered in good condition, inventoried, stored properly, labeled and dispensed in compliance with the institutional guidelines and procedures, all regulatory agencies and per the investigator physician's prescription order.

The dose of ^{89}Zr-Df-IAB22M2C should be removed from the secure refrigerator one hour before the time of infusion in order for the dose to come to room temperature. The ^{89}Zr Df-IAB22M2C dose will be measured by the qualified personnel in a dose calibrator prior to dispensing. Then the syringe will be placed in a shielded carrier along with a designated IV infusion pump for radioactive infusion and administration. The dispensed dose will be infused under the supervision of a qualified nuclear medicine personnel as an IV infusion with an infusion pump over no less than 5 minutes followed by 10-20cc of normal saline flush for injection slowly. The total infusion time should be between 5 to 10 minutes.

After the dose administration, the qualified personnel will return the syringe for residual measurement by the nuclear pharmacist at the site. Measured radioactivity values and times of measurement will be documented, as well as the total injected volume.

8.3.4 Accountability

The Investigator or designee will verify the contents of each shipment against the shipping documents. Verification of study treatment receipt will be documented according to the Sponsor's requirements.

Regulatory agencies require accounting for the disposition of all investigational drugs received by each clinical site. Information on drug disposition required by law consists of the date received, date dispensed, quantity dispensed, and the participant identification number to whom the drug was dispensed. The Investigator is responsible for accounting for all unused study drug and destroying all used study drug containers in compliance with institutional guidelines and

procedures.

A study treatment accountability log will be provided to the site for use by the Investigator to maintain current and accurate inventory records (batch, expiry, and quantity) covering the receipt, dispensing and the destruction of all the study treatments.

8.3.5 **Destruction and Returns**

At the conclusion of the study, or other situations as applicable (ex. site closure, IP expiry, etc.) the Investigator must agree to return or destroy all study materials as instructed by the Sponsor

9. BIOMARKER, CORRELATIVE, AND SPECIAL STUDIES

The precise mechanism by which nivolumab and ipilimumab exert anti-tumor activity is not clear but it is likely related to modulation of the immune system to generate anti-tumor immune responses that are capable of eliminating existing tumors and generating immune memory responses to prevent future relapse. The effect of PD-1/CTLA-4 blockade on effector T cells and regulatory T cells is a critical component of anti-tumor activity. The current study will explore baseline levels as well as changes following nivolumab and ipilimumab administration for association with outcome. In addition, archival tumor expression of PD-L1 will also be evaluated. Peripheral blood will be collected prior to initiation of study therapy, prior to surgery and periodically during the study. Archival tumor will also be collected on all patients. Since this is a surgery study, all tumor samples obtained during the study will be use to evaluate effect of therapy. In addition, if a biopsy or surgical resection is performed at the time of potential progression, a tumor sample (block or slides) should also be submitted if sufficient tumor material is available.
Evaluation of correlative immunologic biomarkers will be performed at the Brain Tumor Immunology Research Laboratory, UCLA Jonsson Comprehensive Cancer Center. Instructions for sample processing and shipping are provided in this section.

Please note that submitting institution is responsible for the costs of shipping and handling.

9.1 Immunophenotyping

The absolute lymphocyte count and proportion of specific lymphocyte subsets will be quantified at each time point. Using mass cytometry (CyToF) analysis, lymphocyte/leukocyte subsets, activation markers, and negative costimulatory molecules, chemokine receptors, and lymph node homing integrin markers will be evaluated before and after administration of ipilimumab, nivolumab and/or placebo. Unsupervised clustering will be performed to identify cell populations that change between timepoints, and also in between treatment groups. CyToF analysis will be performed on PBMC obtained from Ficoll density gradient separation of whole blood. Blood draws for this testing will be done pre-treatment, pre-surgery, and with every MRI scan done for tumor status.

9.2 TIL Density and TCR Overlap

In this study we plan to evaluate whether next generation sequencing of the T cell receptor (TCR) repertoire within glioblastoma and blood can identify shared TCRs, which can then effectively

track anti-tumor immune responses induced by PD-1 and/or CTLA-4 mAb blockade. Genomic DNA will be isolated from fresh-frozen tumor (protocol surgery) and peripheral blood (immune monitoring timepoints) and subjected to next generation sequencing through the TCRVβ region to quantify TIL density and the TCR clones that expand or contract after therapy, and to assess the overlap between tumor and peripheral blood. The TIL density, number of expanded clones, and TCR overlap will then be correlated with clinical variables to identify potential biomarkers with prognostic and predictive value for outcomes (ORR, PFS, OS and toxicity). The TCR next generation sequencing will be performed at Adaptive Biotechnologies (Seattle, WA). Investigators at the UCLA Brain Tumor Immunology Research Laboratory will perform analysis and quantify the parameters listed above. Guidance on peripheral blood collection, processing and shipping is provided in Table 9 below.

9.3 IHC Measurements

A minimum of 1 formalin-fixed paraffin-embedded (FFPE) tumor tissue block (preferred) or a minimum of 10 FFPE unstained sections from pre-study surgery confirming glioblastoma should be submitted preferably within 60 days of registration. Additionally, a minimum 1 formalin-fixed paraffin-embedded (FFPE) tumor tissue block (preferred) or a minimum of 10 FFPE unstained sections from the protocol surgery are to be submitted per Table 9 below. Paraffin blocks from the protocol surgery should be shipped to the UCLA Brain Tumor Research Lab. Multiplex IHC stained will be performed to assess: 1) the proportion of PD-L1 expression on GFAP+ tumor cells versus myeloid cells (CD68+ or CD163+) within the tumor microenvironment; 2) the density of CD4+ and CD8+ T cells within the tumor microenvironment; 3) the percentage of T cells that express PD-1. Staining will be performed on a Dako Autostainer, imaged on a new Vectra Polaris microscope, and analyzed using Halo digital pathology software.

9.4 Gene Expression Signatures and Somatic Mutations

Tumor samples from the protocol surgery should be immersed in Allprotect or RNALater tissue reagent solution (Qiagen) and shipped to the UCLA Brain Tumor Research Laboratory. RNA and exome Seq will be performed at the UCLA GenoSeq Core facility and analyzed. We will assess the number of somatic mutations in each tumor and this data will be correlated with clinical variables to identify potential biomarkers with prognostic and predictive value for outcomes (ORR, PFS, OS). We will also evaluate the presence of a T cell/IFN/cell cycle-related gene expression signature that we previously demonstrated to correlate with clinical responsiveness in adult recurrent glioblastoma patients treated with neoadjuvant PD-1 antibody blockade [36].

9.5 Sample Shipping Instructions and Details

Guidelines for submission of archival tumor tissue (FFPE), fresh-preserved tumor tissue, and/or whole blood (please see Table 9 below for details):

- A memorandum indicating the study, the date of submission, name of study site submitting the tissue, and a list of contents. The UCLA Brain Tumor Research lab will supply a template memorandum to sites at the time of the SIV or upon request.
- A copy of the pathology and surgical report for the sample being submitted should be included in the shipment.

- Slides should be shipped in a plastic slide holder/slide box. Place a small wad of padding in top of the container in order to avoid slides breaking during shipping and handling process.
- For immersion in Allprotect or RNALater tissue reagent, the tumor sample should first be weighed (minimum 200 mg total). Then, the tumor piece should be placed into a specimen shipping container and immersed at a concentration of 100 microliters of Allprotect/10 mg of tissue.
- 10 tubes of blood (BD vacutainer, green top, or approved alternative tubes in case of supply shortage) should be shipped via FedEx for overnight delivery to UCLA.
- An email is to be sent before or at the time of each shipment to the UCLA Brain Tumor Research lab below and the DFCI Neuro Oncology Coordinating Center at NeuroOnc_Coor@dfci.harvard.edu indicating what is being shipped and when.
- Please note that the submitting institution is responsible for the costs of shipping and handling.
- Ship samples to:

Robert M. Prins, Ph.D.
C/O Sylvia Odesa
Gonda Research Lab, Room 1554
695 Charles E. Young Drive South
Los Angeles, CA 90095
Telephone: 310-794-5663
Email: RPrins@mednet.ucla.edu **and** SOdesa@mednet.ucla.edu

Table 9: Correlative Sample Summary

Biomarker Name (Lead PI and Site)	Assay	Tissue/Body Fluid Tested and Timing of Assay	Collection and Packaging	Address to Send Sample
TIL Density and TCR Overlap<br>(R Prins/ T Cloughesy/ L Liau, UCLA)	TCR ImmunoSeq and TCR Overlap (Adaptive Biotech.) on tumor tissue and peripheral blood mononuclear cell genomic DNA	Tumor (protocol surgery) and peripheral blood (Pre-neoadjuvant Tx, Post-neoadjuvant Tx)	100 mg of fresh tissue sent in 2 mL Allprotect ® tissue reagent (Qiagen) or RNALater<br><br>Pre-neoadjuvant Tx and Post-neoadjuvant Tx PBMC can be used from blood collected for flow cytometry (see below)	Robert M. Prins, Ph.D.<br>C/O Sylvia Odesa<br>Gonda Research Lab, Room 1554<br>695 Charles E. Young Drive South<br>Los Angeles, CA 90095<br>Telephone: 310-794-5663<br>Email:<br>RPrins@mednet.ucla.edu and SOdesa@mednet.ucla.edu
IHC measurements<br>(R Prins/T Clougesy/L Liau, UCLA)	PD-1/PD-L1/CD8/CD4/CD8/Iba-1/CD68/GFAP IHC on FFPE tumor tissue	Archived and protocol surgery/10 unstained slides from tumor block	Archived: 10 continuous 5 micron thick slides on Superfrost Plus glass slides<br>Protocol Surgery: 10 continuous 5 micron thick slides on Superfrost Plus glass slides	
Peripheral blood T cell subsets/activation markers<br>(R Prins/ T Cloughesy/ L Liau, UCLA)	CyTOF on peripheral blood mononuclear cells	Peripheral blood (Pre-neoadjuvant Tx, Post-neoadjuvant Tx, with every Tx)	Peripheral blood to be collected in 10x 10ml Green top tubes, at least 10ml per tube at the following time points:<br>Prior to neoadjuvant treatment, post-neoadjuvant (7±2 days from neoadjuvant treatment), prior to each post-surgery study treatment.	
Gene expression signatures and somatic mutations<br>(R Prins/ T Cloughesy/ L Liau, UCLA)	RNA Seq on tumor RNA<br>Exome seq on tumor/PBMC gDNA	Tumor (protocol surgery) and peripheral blood (Pre-neoadjuvant Tx, Post-neoadjuvant Tx)	100 mg of tissue sent in 2 mL Allprotect ® tissue reagent (Qiagen) or RNALater<br><br>Pre-neoadjuvant Tx and Post-neoadjuvant Tx PBMC can be used from blood collected for flow cytometry (see above)	

10. STUDY CALENDAR

Study specific screening procedures may not occur until after the informed consent process is complete. Informed Consent must be obtained by MD attending and may be obtained within 28 days of registration. Screening evaluations are to be conducted within 14 days of registration unless indicated otherwise. Randomization is to be performed within 5 days of registration, and neoadjuvant treatment with must be initiated within 5 days of randomization. Assessments must be performed and reviewed prior to administration of any study agent at any treatment visit. Study drug should be administered within ±2 days of the protocol-specified Day 1 for each post-surgery adjuvant treatment cycle.

Visit	Screening	Neoadjuvant Treatment[1]	Post-Neoadjuvant Tx	Surgery[23]	Post-Surgery Adjuvant Tx [2]		End of Tx [3]	30-Day Post Drug [4]	Active FU [5]	Long Term FU [6]	EDC
					Cycles 1-3	Subsequent Cycles (4-week cycle)					
Visit Window	≤ 14 days to registration	14 ± 5 days before surgery	Day 7 ± 2 days after Tx		C1D1 ⩽ 35 days of surgery		≤ 7 days	30 ±7 days		3 (±1) months	
Medical & GBM History	X										Screening
Vital Signs [7]	X	X			X	X	X				Screening, NA Tx, All Tx Cycles, EOT
Neurologic Exam	X				X	X	X				Screening, All Tx Cycles, EOT
Directed Physical Exam	X	X			X	X	X				Screening, NA Tx, All Tx Cycles, EOT
Karnofsky Performance Status [8]	X	X		X	X	X	X	X			Screening, NA Tx, Surgery, All Tx Cycles, EOT, 30 Day
Concomitant Medications	------------------------------------X--										Screening, NA Tx, Surgery, All Tx Cycles, EOT, 30 Day
Adverse Event Assessment [9]		--------------------------------------X--									NA Tx, Surgery, All Tx Cycles, EOT, 30 Day
Pregnancy Test [10]	X	X[1]			X	X	X				Screening, NA Tx, All Tx Cycles, EOT
PT/INR, PT, PTT	X			X							Screening, Surgery
Hematology [11]	X	X[1]		X	X	X	X				Screening, NA Tx, Surgery, All Tx Cycles, EOT
Serum Chemistry [12]	X	X[1]		X	X	X	X				Screening, NA Tx, Surgery, All Tx Cycles, EOT
FT3, FT4, TSH [13]	X	X[1]			X	X	X				Screening, NA Tx, All Tx Cycles, EOT
Virology [14]	X										Screening
Cortisol [15]				X							Surgery
Urinalysis [16]	X			X	X		X				Screening, Surgery, All Tx Cycles
Imaging – MRI [17]	X			X	X	X	X		X		Screening, Surgery, All Tx Cycles, EOT, Active FU
Response Assessment (RANO)					X	X	X		X		All Tx Cycles, EOT, Active FU
Nivolumab / placebo [18]		X			X	X					NA Tx, All Tx Cycles (All Arms)
Ipilimumab / placebo [18]		X			X						NA Tx, Arm 1: Tx Cycles 1-2, Arm 2: Cycles 1-4
Research Blood [19]		X	X		X	X	X				NA Tx, Surgery, All Tx Cycles, EOT
Tissue Submission [20]	X			X							Screening, Surgery
Survival & Post-EOT Therapies [21]								X	X	X	30 Day, Active FU, LTFU
^{89}Zr-Df-IAB22M2C& PET Scan [22]	X		X								Screening and pre surgery

1. Screening assessments may serve as neoadjuvant treatment assessment within ≤7 days of treatment, except in the event that there is any indication the participant's condition is deteriorating for which laboratory evaluations should be repeated within 48 hours prior to initiation of neoadjuvant treatment.
2. Nivolumab and ipilimumab must be administered ±2 days from Day 1 of each post-surgery cycle. Required assessments should be performed within 7 days of scheduled visit days and reviewed prior to study agent administration.
3. End of Treatment assessments to be performed within 7 days after last drug administration or within 7 days after decision to end treatment. Assessments may continue for ongoing reportable adverse events. Do not repeat the safety laboratory tests and exams if the previous assessments were completed within 14 days from the EOT visit.
4. 30 Day Post Drug: A phone contact or clinic visit is to be performed with a study nurse or clinician. All participants will be followed until resolution or stabilization of any serious or reportable adverse events occurring during treatment or starting within 30 days of last study drug.
5. Active Follow-Up: For participants who discontinue study treatment for reasons other than disease progression, every effort should be made to continue monitoring their disease status by radiologic imaging at a schedule developed by the treating investigator until (1) documented disease progression, (2) death or (3) the end of the study, whichever occurs first.
6. Long Term Follow Up: participants will be followed every 3 (±1) months via contact or medical record review until death for post-treatment therapies, including start/stop dates, names of treatment regimens, and reason for stopping those therapies, and survival.
7. Vital Signs: weight, heart rate, blood pressure, respiration rate, temperature. Must be performed prior to administration of treatment(s) on treatment days. Height required only at screening.
8. Performance Status: to be assessed per Appendix A for KPS scale.
9. AEs will be collected and recorded from the first study treatment until the 30-Day Post Drug Visit. All SAEs and ECIs will be collected and reported until 100 days after the last dose of trial treatment or the start of new anti-cancer treatment, whichever comes first. See Section 7.3 for AE reporting details.
10. For WOCBP, a urine or serum pregnancy test must be performed. If urine pregnancy results cannot be confirmed as negative, a serum pregnancy test will be required. A negative urine or serum pregnancy test is required within 72 hours prior to registration.
11. Hematology: RBC, hemoglobin, hematocrit, platelets, total WBC plus differential (neutrophils, lymphocytes, monocytes, eosinophils, basophils).
12. Serum Chemistry: albumin, alkaline phosphatase, bicarbonate, BUN, calcium, chloride, creatinine, glucose, potassium, SGOT/AST, SGPT/ALT, sodium, total protein, total bilirubin, and phosphorus.
13. FT3, FT4, TSH must be drawn prior to study drug/placebo administration. Treatment may proceed if results are not available for review prior to study drug administration, but results should be reviewed when available.
14. Virology: Hepatitis A Antibody, Hepatitis B Core IgG and M Antibody (Anti-HBC, total), Hepatitis B Surface Antibody (Anti-HBs), Hepatitis B Surface Antigen (HBsAg), Hepatitis C Surface Antibody (anti-HCV), HIV-1/2 Ag/Ab. If any hepatitis serology is positive, qualitative PCR for Hepatitis B DNA or Hepatitis C RNA will be obtained and results must be negative for eligibility.
15. Cortisol: to be collected in the morning, prior to surgery, to monitor for subclinical immune-mediated adrenal insufficiency
16. Urinalysis: blood, glucose, protein, specific gravity. If abnormal, microscopic exam must be performed.
17. Imaging: gadolinium-enhanced contrast and non-contrast MRI. Screening MRI should be within 14 days prior to registration. MRIs pre and post-surgery will be performed per standard of care according to institutions' standard practice (within 72 hours after surgery; preferably within 24 hours). Post-surgery imaging should be performed prior to Cycle 1 Day 1 if 28 days or more from surgery. MRIs will be repeated every 8 ± 4 weeks. The same imaging technique should be used in a participant throughout the trial, if feasible. Local reading (investigator assessment) will be used to determine eligibility and for participant management. Starting from EOT visit, subjects will be followed up by radiologic imaging per standard of care.
18. See Treatment Plan (Section 5) for nivolumab, ipilimumab, and placebo administration details
19. Research Blood: see Table 9 - Correlative Sample Summary for details
20. Tissue: see Table 9 - Correlative Sample Summary for details
21. Date of death and reason should be collected for overall survival purposes, when applicable.
22. To be performed after registration but before starting neoadjuvant treatment and again 3 days before surgery +/- 2 days
23. There is a ±1 day window for day of surgery assessments, but all should be collected prior to surgery if possible.

11. MEASUREMENT OF EFFECT

Tumor response will be assessed every 8 weeks for patients treated on this study using modified RANO criteria [1] with incorporation of guidance from iRANO criteria [2] for confirmation of progression on follow-up imaging as outlined. Clinicians may repeat response assessment more frequently as clinically indicated.

11.1 Antitumor Effect Definitions

Evaluable for toxicity. All participants who receive at least one dose of study treatment will be evaluable for toxicity from the time of their first treatment.

Evaluable for objective response. Only those participants who have measurable disease present at baseline (obtained within 28 days of cycle 1, day 1) scan and have received at least one dose of therapy will be considered evaluable for response. These participants will have their response classified according to the definitions stated below. (Note: Participants who exhibit objective disease progression or die prior to the end of cycle 1 will also be considered evaluable.)

Measurable disease. Bi-dimensionally, contrast-enhancing, measurable lesions with clearly defined margins by CT or MRI scan, with a minimal diameter of 1 cm, and visible on 2 axial slices which are at least 5 mm apart with 0 mm skip. Measurement of tumor around a cyst or surgical cavity, if necessary, requires a minimum thickness of 3 mm. If there are too many measurable lesions to measure at each evaluation, the investigator must choose the largest two to be followed before a participant is entered on study. The remaining lesions will be considered non-measurable for the purpose of objective response determination. Unless progression is observed, objective response can only be determined when all measurable and non-measurable lesions are assessed.

Non-measurable evaluable disease. Unidimensionally measurable lesions, masses with margins not clearly defined, lesions with maximal diameter < 1cm.

11.2 Methods for Evaluation of Disease

All measurements should be taken and recorded in metric notation, using a ruler, calipers, or digital measurement tool. All baseline evaluations should be performed as closely as possible to the beginning of treatment and never more than 14 days before the beginning of the treatment.

The same method of assessment and the same technique should be used to characterize each identified and reported lesion at baseline and during follow-up

11.3 Response Criteria

Complete response (CR). All of the following criteria must be met:

a) Complete disappearance of all enhancing measurable and non-measurable disease sustained for at least 4 weeks. In the absence of a confirming scan 4 weeks later, this scan will be considered only stable disease.

b) No new lesions.
c) All measurable and non-measurable lesions must be assessed using the same techniques as baseline.
d) Participants must be on no steroids or on physiologic replacement doses only.
e) Stable or improved non-enhancing (T2/FLAIR) lesions
f) Stable or improved clinically, for clinical signs and symptoms present at baseline and recorded to be disease related

Participants with non-measurable disease cannot have a complete response. The best response possible is stable disease.

<u>Partial response (PR).</u> All of the following criteria must be met:

a) Greater than or equal to 50% decrease compared to baseline in the sum of products of perpendicular diameters of all measurable enhancing lesions sustained for at least 4 weeks. In the absence of a confirming scan 4 weeks later, this scan will be considered only stable disease.
b) No progression of non-measurable disease.
c) No new lesions.
d) All measurable and non-measurable lesions must be assessed using the same techniques as baseline.
e) The steroid dose at the time of the scan evaluation should be no greater than the dose at time of baseline scan.
f) Stable or improved non-enhancing (T2/FLAIR) lesions on same or lower dose of corticosteroids compared to baseline scan.
g) Stable or improved, for clinical signs and symptoms present at baseline and recorded to be disease related clinically.

Participants with non-measurable disease cannot have a partial response. The best response possible is stable disease.

<u>Progressive disease (PD).</u> Any of the following criterion must be met:

a) > 25% increase in sum of the products of perpendicular diameters of enhancing lesions (over best response or baseline if no decrease) on stable or increasing doses of corticosteroids
b) Any new enhancing measurable lesion
c) Clear clinical deterioration not attributable to other causes apart from the tumor (e.g. seizures, medication side effects, complications of therapy, cerebrovascular events, infection, etc.). The definition of clinical deterioration is left to the discretion of the investigator but it is recommended that a decline in the Karnofsky Performance Score (KPS) from 100 or 90 to 70 or less, a decline in KPS of at least 20 from 80 or less, or a decline in KPS from any baseline to 50 or less, for at least 7 days, be considered neurologic deterioration, unless attributable to co-morbid events or changes in corticosteroid dose.
d) Failure to return for evaluation due to death or deteriorating condition

<u>Stable disease (SD).</u> All of the following criteria must be met:

a) Does not qualify for CR, PR, or progression.
b) All measurable and non-measurable sites must be assessed using the same techniques as baseline.
c) Stable clinically.

Unknown response status. Progressive disease has not been documented and one or more measurable or non-measurable lesions have not been assessed.

The modified RANO Response Criteria to be used in this study are summarized in Table 10.

Table 10 Summary of the RANO Response Criteria

	CR	PR	SD	PD#
T1-Gd +	None	≥50% decrease	<50% decrease-<25% increase	≥25% increase*
T2/FLAIR	Stable or decrease	Stable or decrease	NA	NA
New Lesion	None	None	None	Present*
Corticosteroids	None	Stable or decrease	Stable or decrease	Stable or increasing
Clinical Status	Stable or increase	Stable or increase	Stable or increase	Decrease*
Requirement for Response	All	All	All	Any*

CR=complete response; PR=partial response; SD=stable disease; PD=progressive disease; NA= not applicable

#: Progression occurs when any of the criteria with * is present

Increase in corticosteroids alone will not be taken into account in determining progression in the absence of persistent clinical deterioration

Of note, patients who require increased corticosteroids within two weeks of MRI assessment (relative to the dose taken at the time of the prior assessment) cannot be classified as CR, PR or SD and should be classified as non-evaluable at that time point. Conversely, patients who decrease corticosteroids within two weeks of MRI assessment (relative to the dose taken at the time of the prior assessment) cannot be classified as PD and should be classified as non-evaluable.

11.4 Evaluation of Best Response

The best overall response is the best response recorded from the start of the treatment until disease progression (taking as reference for progressive disease the smallest measurements recorded since the treatment started). If a response recorded at one scheduled MRI does not persist at the next regular scheduled MRI, the response will still be recorded based on the prior scan but will be designated as a non-sustained response. If the response is sustained, i.e. still present on the subsequent MRI at least four weeks later, it will be recorded as a sustained response, lasting until the time of tumor progression. Participants without measurable disease may only achieve SD or

PD as their best "response."

11.5 Study Continuation Beyond Initial Progressive Disease

Immunotherapeutic agents such as nivolumab and ipilimumab may produce antitumor effects by potentiating endogenous cancer-specific immune responses which may manifest as initial worsening of enhancement and edema on MRI or CT scans (i.e. pseudoprogression). In addition, the response patterns seen with immunotherapeutics may extend beyond the typical time course of responses seen with cytotoxic agents and can manifest a clinical response after an initial increase in tumor burden or even the appearance of new lesions. For these reasons, the immune-related response criteria (irRC) have endorsed continuation of study therapy beyond initial radiographic evidence of progression for clinically stable patients undergoing immune based therapies.[57]
A major advance of the RANO criteria [1] to assess response in neuro-oncology over the previously used Macdonald criteria [58] includes recognition of the prevalence of pseudoprogression during the first three months following completion of radiation and daily temozolomide.[59, 60] Specifically, RANO permits patients with such progressive MRI findings to continue temozolomide therapy for up to three months in order to avoid inaccurately classifying such patients as progressive. Furthermore, RANO permits patients with progressive radiographic findings at any time to continue current therapy pending follow-up imaging if the etiology of progressive imaging findings is unclear. Standard RANO may not provide an accurate response assessment of immunotherapeutic agents such as nivolumab and ipilimumab.

Therefore, the following adaptations of the RANO criteria will be used to assess response for patients treated on this study (Table 11):

- <u>Potential Pseudoprogression</u>: If radiologic imaging shows initial PD, subjects who are not experiencing significant clinical decline, may be allowed to continue study treatment for up to three months. Patients who have radiographic evidence of further progression after up to three months, or who decline significantly at any time, will be classified as progressive with the date of disease progression back-dated to the first date that the subject met criteria for progression and such subjects will be discontinued from study therapy. Although the kinetics of pseudoprogression due to immune checkpoint blockade among glioblastoma patients is currently unknown, three months is a reasonable estimate based on: 1) the peak time for XRT/daily temozolomide-related pseudoprogression is usually within three months of completion for glioblastoma patients [59, 60] and; 2) three months is also the most common timeframe for pseudoprogression observed among patients with advanced melanoma or other solid tumors treated with PD-1/PD-L1immune checkpoint blockade to date.[61-63]

  Among patients on this study with initial radiographic PD, tumor assessment should be repeated regularly (every 1-2 months) in order to confirm PD with the option of continuing treatment as described below while awaiting radiologic confirmation of progression. If repeat imaging shows a stabilization or reduction in the tumor burden compared to the initial scan demonstrating PD, treatment may be continued / resumed. If repeat imaging after up to three months confirms progressive disease, then the date of disease progression will be the first date the subject met criteria for progression and subjects will be discontinued from study therapy. Subjects who have confirmed disease progression will

discontinue study medication and enter the follow up/survival phase of the study. In determining whether or not the tumor burden has increased or decreased, investigators should consider all target lesions as well as non-target lesions.

- Tumor Enhancement to Define Progression: RANO expanded the previously utilized Macdonald criteria[58] to include the development of "significantly" increased T2 or FLAIR abnormality in the definition of progressive disease because such changes can be a major component defining radiographic progression following therapeutic use of VEGF/VEGFR-targeting therapeutics which are known to elicit potent anti-permeability changes that limit contrast uptake. However, immune based therapies are expected to be associated with inflammatory changes that may include edema. Therefore, our study will define radiographic progressive disease by assessment of enhancing tumor burden only and will not include assessment of T2 or FLAIR changes as outlined in RANO because:
  - There is no expectation that immunotherapy agents including PD-1 inhibitors will falsely diminish enhancing tumor burden as has been noted with anti-angiogenic therapies; and
  - Immune based therapies are expected to induce inflammatory responses which may be associated with increased edema and T2/FLAIR changes. Such radiographic finding may inaccurately be interpreted to represent tumor progression (i.e. pseudoprogression).

In subjects who have initial evidence of radiographic PD, it is at the discretion of the treating physician whether to continue a subject on study treatment for up to three months pending confirmation of PD on follow-up imaging as described in the iRANO criteria.[2] This clinical judgment decision should be based on the subject's overall clinical condition, including performance status, clinical symptoms, and laboratory data. Subjects may receive study treatment while waiting for confirmation of PD if they are not experiencing significant clinical decline and if:

- The subject is believed to demonstrate clinical benefit from the study regimen as determined by the treating physician;
- The subject is adequately tolerating study therapy.

When feasible, study therapy should not be discontinued until radiographic progression is confirmed. This allowance to continue treatment despite initial radiologic progression takes into account the observation that some subjects can have a transient tumor flare in the first few months after the start of immunotherapy, but with subsequent disease response.[57] Subjects that are exhibiting significant neurologic decline are not required to have repeat imaging for confirmation of progressive disease.

Table 11: Imaging and Treatment After 1st Radiologic Evidence of PD

	No Significant Neurologic Decline		Significant Neurologic Decline	
	Imaging	Treatment	Imaging	Treatment
1st radiologic evidence of PD	Repeat imaging for up to 3 months to confirm PD	May continue study treatment at the Investigator's discretion for up to 3 months while awaiting confirmatory scans	Repeat imaging >6 weeks later to confirm PD if possible	Discontinue treatment
Repeat scan after 3 months confirms PD	No additional imaging. Date of progression is backdated to date of 1st radiographic PD	Discontinue treatment	No additional imaging required	Not applicable
Repeat scan shows SD, PR or CR	Continue regularly scheduled imaging assessments every 8 weeks	Continue study treatment at the Investigator's discretion	Continue regularly scheduled imaging assessments every 8 weeks	May restart study treatment if condition has improved and/or clinically stable per Investigator's discretion

Participants with progressive radiographic findings are encouraged to undergo surgical intervention in order to delineate pseudoprogression due to inflammation associated with PD-1 blockade from true tumor progression. Participants with histopathologic findings of significant immune infiltrate and evolving gliosis will be allowed to continue study therapy. In contrast, those with clear evidence of progressive tumor by histopathologic evaluation will be defined as progressive and discontinued from study therapy. For such patients, the date of tumor progression will be the first date the participant met radiographic criteria for PD

11.6 Central Radiology Review

The central review of neuroimaging (MRI or CT) will be performed at Dana-Farber Cancer Institute or UCLA (as determined by Sponsor-Investigator) on participants when requested from the DFCI Coordinating Center on behalf of the Sponsor-Investigator. All films of all views from pre-registration and subsequent scans must be submitted for central review. CDs are preferred.

A copy of the local treating investigator's tumor measurements and response assessment must be submitted with the films. A copy of all scan reports must be attached for inclusion in the submission. Once the Central Review is complete the Reviewing Physician will document the review results. Once the Central Review is complete, the central review results can be made available to the local PI.

When requested from the DFCI Coordinating Center, please send a copy of all scans to:

Dr. Patrick Wen c/o Brenda Acevedo
Center for Neuro-Oncology
Dana-Farber Cancer Institute

450 Brookline Ave, LG-GC12D
Boston, MA 02215
ph: 617-632-3438
fax: 617-582-7782

A memo must be submitted to the DFCI Coordinating Center at NeuroOnc_Coor@dfci.harvard.edu each time submissions are made including DFCI study number, participant identifiers and details of what is being submitted.

The submitting institution is responsible for the costs of shipping and handling.

12. DATA REPORTING / REGULATORY REQUIREMENTS

Adverse event lists, guidelines, and instructions for AE reporting can be found in Section 7.0 (Adverse Events: List and Reporting Requirements).

12.1 Data Reporting

12.1.1 **Method**

The DF/HCC Office of Data Quality (ODQ) will collect, manage, and perform quality checks on the data for this study.

12.1.2 **Responsibility for Data Submission**

Investigative sites are responsible for submitting data and/or data forms to the Office of Data Quality (ODQ) in accordance with DF/HCC policies.

12.2 Data Safety Monitoring

The DF/HCC Data and Safety Monitoring Committee (DSMC) will review and monitor toxicity and accrual data from this study. The committee is composed of medical oncologists, research nurses, pharmacists and biostatisticians with direct experience in cancer clinical research. Information that raises any questions about participant safety will be addressed with the Sponsor-Investigator and study team.

The DSMC generally reviews each protocol up to four times a year with the frequency determined by the outcome of previous reviews. Information to be provided to the committee may include: up-to-date participant accrual; current dose level information; DLT information; all grade 2 or higher unexpected adverse events that have been reported across all sites; summary of all deaths occurring within 30 days of intervention for Phase I or II protocols; for gene therapy protocols, summary of all deaths while being treated and during active follow-up; any response information;

audit results, and a summary provided by the study team. Other information (e.g. scans, laboratory values) will be provided upon request.

12.3 Multicenter Guidelines

This protocol will adhere to DF/HCC Policy MULTI-100 and the requirements of the DF/HCC Multi-Center Data and Safety Monitoring Plan. The specific responsibilities of the Sponsor-Investigator, Coordinating Center, and Participating Institutions and the procedures for auditing are presented in Appendix B.

13. STATISTICAL CONSIDERATIONS

This section outlines the statistical analysis strategy and procedures for the study. If, after the study has begun, changes are made to primary and/or key secondary hypotheses, or the statistical methods related to those hypotheses, then the protocol will be amended (consistent with ICH Guideline E-9). Changes to exploratory or other non-confirmatory analyses made after the protocol has been finalized, along with an explanation as to when and why they occurred, will be listed in the Clinical Study Report (CSR) for the study. Post hoc exploratory analyses will be clearly identified in the CSR.

13.1 Statistical Analysis Plan Summary

This section contains a brief summary of the statistical analyses for this trial. The primary outcome, tumor infiltrating T lymphocyte (TIL) density, will be assessed and compared between the three arms (Arm 1 vs Arm 3; Arm 2 vs Arm 3; Arm 1 vs Arm 2). The secondary efficacy endpoint of PFS-6 will be assessed using pooled data Arm 1, Arm 2 and Arm 3 patients and compared to appropriate historical controls.

13.1.1 Efficacy Analyses

The primary and key secondary endpoints, primary analysis population, and statistical methods that will be employed for the efficacy analyses are discussed in detail in the following sections**.**

13.1.2 Safety Analyses

Adverse events will be graded according to Common Terminology Criteria for Adverse Events (CTCAE) version 4.0. All patients who receive any amount of nivolumab or ipilimumab will be evaluable for toxicity. The All-Patients-as-Treated population will be employed for safety analyses. Immune related adverse experiences are prespecified as Events of Clinical Interest (Section 7.3.4).

If in the first 9 patients one-third or more of patients (3 or more) experience drug-associated adverse events which compromise or delay surgical resection the safety of the study will be evaluated.

13.2 Statistical Analysis Plan

The main purpose of this new neoadjuvant trial is to evaluate whether the combination of CTLA-4 (ipilimumab) + PD-1 antibody blockade (nivolumab) significantly increases the influx of T lymphocytes into the tumor (measured by TIL Density) above that from PD-1 antibody blockade (nivolumab) alone. We also would like to evaluate whether PD-1 antibody blockade (nivolumab) increases the influx of T lymphocytes as compared to the control placebo group. 60 patients will be randomized into three Arms as depicted in the schema (Figure 1): 25 patients for Arm 1 (nivo + ipi), 25 patients for Arm 2 (nivo + placebo-ipi), and 10 patients for Arm 3 (placebo-nivo + placebo-ipi).

Since we already have TIL Density data from our previous single agent neoadjuvant PD-1 antibody blockade trial in the same population, and with the same eligibility criteria, it makes sense to put more patients in the combination treatment group in this trial.

13.2.1 **Responsibility for Analyses**

The statistical analysis of the data obtained from this study will be the responsibility of the study responsible biostatistician, Ms. Jiyoon Kim of JCCC BASE SR.

The neoadjuvant treatment period of this trial is blinded, i.e., subjects, site investigators, site staff, and SPONSOR personnel will be unaware of subject treatment assignments after each subject is randomized and treatment is assigned.

The adjuvant treatment period of the trial is being conducted as an open-label study, i.e., subjects, site investigators, site staff, and SPONSOR personnel will be aware of subject treatment assignments after each subject is enrolled and treatment is assigned.

13.2.2 **Hypotheses/Estimation**

Objectives and hypotheses of the study are stated in Section 1.0.

13.2.3 **Power and Sample Size for Primary Endpoint**

60 patients will be randomly assigned to Arm 1 (25), Arm 2 (25), or Arm 3 (10) prior to surgery. The two-sample t-test will be used to compare different groups (Arm 1 vs Arm 3, Arm 2 vs Arm 3, and Arm 1 vs Arm 2) for the primary endpoint as measured by TIL density. No multiple test adjustment is planned for this early phase clinical trial.

With 25 patients in Arm 1 and 25 patients in Arm 2, there is about 80% power to detect an effect size of 0.71 of TIL density comparing Arm 1 versus Arm 2 at an alpha level of 0.05 (1-sided). With 25 patients in a treatment group (Arm 1 or Arm 2) and 10 patients in Arm 3, there is about 80% power to detect an effect size of 0.95 of TIL density comparing different groups (Arm 1 vs Arm 3, and Arm 2 vs Arm 3) at an alpha level of 0.05 (1-sided).

13.2.4 **Power and Sample size for Secondary (Efficacy) Endpoint**

The secondary endpoint of this trial is to evaluate PFS6 in comparison with a historical control PFS6 rate of 10%, which was documented from a pooled analysis of Phase II experience in recurrent Grade IV gliomas who have undergone surgery either just prior to starting treatment or as part of the protocol [64]. Using a one sided alpha of 5% with a sample size of 25 (Arm 1 or Arm 2), there is above 94.37% power to detect an improvement over the historical PFS6 rate of 10%

for ineffective agents if the true PFS6 rate is 35%. No multiple test adjustment is planned for this early phase clinical trial.

13.3 Stratification Factors

No stratification based on age, gender or other characteristic will be used in this trial.

13.4 Analysis Endpoints

Efficacy and safety endpoints that will be evaluated for within- and/or between-treatment differences are listed below, followed by the descriptions of the derivations of selected endpoints

13.4.1 Efficacy Endpoints

Efficacy will be measured by percent PFS6.

13.4.2 Safety Endpoints

The primary safety endpoints are AEs graded using CTCAE v4.0 criteria. Safety will be assessed by quantifying the toxicities and grades experienced by subjects who have received nivolumab, ipilimumab or the combination of nivolumab plus ipilimumab, including serious adverse events (SAEs) and events of clinical interest (ECIs). Immune related adverse experiences (irAEs) (as defined Section 7.3.4) are prespecified as events of interest. Other safety endpoints include laboratory safety assessments, KPS status, vital signs and physical examinations.

13.5 Analysis Population

13.5.1 Efficacy Analysis Populations

The Full Analysis Set (FAS) population will serve as the primary population for the analysis of efficacy data in this study. The FAS population consists of all subjects within each cohort who have undergone the scheduled tumor resection.

Two supportive analyses of the primary and selected secondary efficacy endpoints will be conducted. The first supportive analysis will be conducted in the FAS-2 population, defined as all subjects who meet the FAS population definition and have a post baseline scan OR discontinue the trial due to progressive disease/drug related AE. The second analysis will be conducted using the intention to treat (ITT) population, defined as all randomized subjects.

Subjects will be included in the cohort to which they are randomized for the analysis of efficacy data.

13.5.2 Safety Analysis Populations

The All Patients as Treated (APaT) population will be used for the analysis of safety data in this study. The APaT population consists of all allocated subjects who received at least one dose of study treatment.

At least one laboratory or vital sign measurement obtained subsequent to at least one dose of study treatment is required for inclusion in the analysis of each specific parameter. To assess change

from baseline, a baseline measurement is also required.

13.6 Statistical Methods

Nominal p–values may be computed for efficacy analyses as a measure of strength of association between the endpoint and the treatment effect rather than formal tests of hypotheses. Unless otherwise stated, all statistical tests will be conducted at the $\alpha=0.05$ level.

13.6.1 **Statistical Methods for Primary Endpoint and Efficacy Analyses**

A two-sample one-sided Student's t-test will be used to test the difference of tumor infiltrating T lymphocyte (TIL) density between different arms (Arm 1 vs Arm 3, Arm 2 vs Arm 3, and Arm 1 vs Arm 2). Data distribution will be examined prior to analysis. Data transformations will be performed as deemed appropriate.

For PFS endpoint, the analyses will be conducted using pooled Arm 1 or Arm 2 patients. Percent PFS6 will be estimated and compared using the exact Binomial test.

Kaplan-Meier (KM) curves and median estimates from the KM curves will be provided as appropriate. Median OS will be estimated and compared using the KM curves.

13.6.2 **Statistical Methods for Safety Analyses**

Safety and tolerability will be assessed by clinical review of all relevant parameters including adverse experiences (AEs), laboratory tests, and vital signs. Safety summaries will be reported for both cohorts.

Immune related adverse experiences (irAEs, as defined in Section 7.3.4) are prespecified as events of interest. These events will be summarized in separate tables from other AEs by toxicity grade and will include the counts, percentage, and 95% CI. Any AE of unknown etiology associated with nivolumab, ipilimumab or the combination of nivolumab plus ipilimumab exposure will be evaluated to determine if it is possibly an event of clinical interest (ECI) of a potentially immunologic etiology (irECI). Other ECIs listed in Section 7.3.4 will also be summarized in the same manner as irAEs.

Adverse experiences (specific terms as well as system organ class terms) and predefined limits of change in laboratory, and vital sign parameters that are not pre-specified as events of interest will be summarized with descriptive statistics (counts, percentage, mean, standard deviation, etc.).

Continuous measures such as changes from baseline in laboratory, and vital signs parameters that are not pre-specified as events of interest will be summarized using descriptive statistics (mean, standard deviation, etc.) for baseline, on-treatment, and change from baseline values.

13.6.3 **Statistical Methods for Exploratory Analyses**

Variables involved in exploratory analyses will be examined graphically and summarized by descriptive statistics. Data transformation may be applied to quantitative variables prior to analyses if deemed necessary.

Bivariate association analyses will be conducted to evaluate the associations between exploratory biomarkers and clinical outcomes and adverse events for the objectives listed in 1.4. The analyses

will be performed for each group separately and/or combined as deemed appropriate. Specifically, Pearson or Kendall's tau correlation coefficients will be calculated to evaluate the association between quantitative variables, Chi-squared tests or Fisher's exact tests will be used to examine the association between categorical or ordinal variables, and ANOVA will be performed to study the association between a quantitative variable and a categorical variable. The association between biomarkers and clinical outcomes (PFS and OS) will be evaluated using Cox regression. For markers with multiple measurements over time, data at a specific time point or change from pre-treatment will be used in the above bivariate analyses as deemed appropriate.

Changes in markers pre- and post- treatment will be assessed using paired t-tests.

For participants undergoing ^{89}Zr-Df-IAB22M2C administration with PET imaging, ^{89}Zr-Df-IAB22M2C uptake in tumors will be determined using SUV-based quantitative measures (e.g. SUVmax, SUVpeak, SUVmean, CD8 tumor volume defined as volume of tumor tissues with increased CD8 uptake, and Tumor: Reference ratio) with CD8+ TIL density determined by IHC from biopsy samples. In addition, comparison of baseline and post-neoadjuvant immune checkpoint therapy uptake and distribution of ^{89}Zr-Df-IAB22M2C in tumors will be assessed. Descriptive statistics (n, mean, standard deviation, median, minimum and maximum) will be presented for continuous variables. Frequencies and percentages will be presented for categorical variables. All inferential statistical analyses will be based on a two-sided test with a Type I error rate of 0.05.

13.7 Summary of Baseline Characteristics and Demographics

Baseline characteristics will be assessed by the use of tables and/or graphs for each cohort separately. No statistical hypothesis tests will be performed on these characteristics. The number and percentage of subjects screened, randomized, the primary reasons for screening failure, and the primary reason for discontinuation will be displayed. Demographic variables (e.g., age, gender), baseline characteristics, primary and secondary diagnoses, and prior and concomitant therapies will be summarized by treatment either by descriptive statistics or categorical tables.

13.8 Compliance (Medication Adherence)

A day within the study will be considered an On-Therapy day if the subject receives the study medication infusion. The number of Days Should be on Therapy is the total number of days from the first day of study medication to the date of the last dose of study medication. For each subject, percent compliance will then be calculated using the following formula:

Percent Compliance = (Number of Days on Therapy/Number of Days Should be on Therapy) x100

Summary statistics will be provided on percent compliance by treatment group for the FAS population.

13.9 Extent of Exposure

Extent of Exposure for a subject is defined as number of cycles in which the subject receives the study medication infusion. Summary statistics will be provided on Extent of Exposure for APaT

population.

14. PUBLICATION PLAN

This trial is intended for publication, even if terminated prematurely. Publication may include any or all of the following: posting of a synopsis online, abstract and/or presentation at a scientific conference, or publication of a full manuscript. The Sponsor will work with the authors to submit a manuscript describing trial results within 12 months after the last data become available, which may take up to several months after the last subject visit in some cases. The Sponsor-Investigator will post a synopsis of trial results for approved products on www.clinicaltrials.gov by 12 months after the last subject's last visit for the primary outcome, 12 months after the decision to discontinue development, or product marketing (dispensed, administered, delivered or promoted), whichever is later.

These timelines may be extended for products that are not yet marketed, if additional time is needed for analysis, to protect intellectual property, or to comply with confidentiality agreements with other parties. Authors of the primary results manuscript will be provided the complete results from the Clinical Study Report, subject to the confidentiality agreement. When a manuscript is submitted to a biomedical journal, the Sponsor's policy is to also include the protocol and statistical analysis plan to facilitate the peer and editorial review of the manuscript. If the manuscript is subsequently accepted for publication, the Sponsor will allow the journal, if it so desires, to post on its website the key sections of the protocol that are relevant to evaluating the trial, specifically those sections describing the trial objectives and hypotheses, the subject inclusion and exclusion criteria, the trial design and procedures, the efficacy and safety measures, the statistical analysis plan, and any amendments relating to those sections. The Sponsor reserves the right to redact proprietary information.

For multicenter trials, subsequent to the multicenter publication (or after public disclosure of the results online at www.clinicaltrials.gov if a multicenter manuscript is not planned), an investigator and his/her colleagues may publish their data independently. In most cases, publication of individual trial site data does not add value to complete multicenter results, due to statistical concerns. In rare cases, publication of single trial site data prior to the main paper may be of value. Limitations of single trial site observations in a multicenter trial should always be described in such a manuscript.

Authorship credit should be based on 1) substantial contributions to conception and design, or acquisition of data, or analysis and interpretation of data; 2) drafting the article or revising it critically for important intellectual content; and 3) final approval of the version to be published. Authors must meet conditions 1, 2 and 3. Significant contributions to trial execution may also be taken into account to determine authorship, provided that contributions have also been made to all three of the preceding authorship criteria. Although publication planning may begin before conducting the trial, final decisions on authorship and the order of authors' names will be made based on participation and actual contributions to the trial and writing, as discussed above. The first author is responsible for defending the integrity of the data, method(s) of data analysis and the scientific content of the manuscript.

The Sponsor must have the opportunity to review all proposed abstracts, manuscripts or presentations regarding this trial 45 days prior to submission for publication/presentation. Any information identified by the Sponsor as confidential must be deleted prior to submission; this confidentiality does not include efficacy and safety results. Sponsor review can be expedited to meet publication timelines.

REFERENCES

[1] P.Y. Wen, D.R. Macdonald, D.A. Reardon, T.F. Cloughesy, A.G. Sorensen, E. Galanis, J. Degroot, W. Wick, M.R. Gilbert, A.B. Lassman, C. Tsien, T. Mikkelsen, E.T. Wong, M.C. Chamberlain, R. Stupp, K.R. Lamborn, M.A. Vogelbaum, M.J. van den Bent, S.M. Chang, Updated response assessment criteria for high-grade gliomas: response assessment in neuro-oncology working group, J Clin Oncol, 28 (2010) 1963-1972.
[2] H. Okada, M. Weller, R. Huang, G. Finocchiaro, M.R. Gilbert, W. Wick, B.M. Ellingson, N. Hashimoto, I.F. Pollack, A.A. Brandes, E. Franceschi, C. Herold-Mende, L. Nayak, A. Panigrahy, W.B. Pope, R. Prins, J.H. Sampson, P.Y. Wen, D.A. Reardon, Immunotherapy response assessment in neuro-oncology: a report of the RANO working group, The Lancet. Oncology, 16 (2015) e534-542.
[3] R. Stupp, W.P. Mason, M.J. van den Bent, M. Weller, B. Fisher, M.J. Taphoorn, K. Belanger, A.A. Brandes, C. Marosi, U. Bogdahn, J. Curschmann, R.C. Janzer, S.K. Ludwin, T. Gorlia, A. Allgeier, D. Lacombe, J.G. Cairncross, E. Eisenhauer, R.O. Mirimanoff, Radiotherapy plus concomitant and adjuvant temozolomide for glioblastoma, The New England journal of medicine, 352 (2005) 987-996.
[4] K.V. Ballman, J.C. Buckner, P.D. Brown, C. Giannini, P.J. Flynn, B.R. LaPlant, K.A. Jaeckle, The relationship between six-month progression-free survival and 12-month overall survival end points for phase II trials in patients with glioblastoma multiforme, Neuro Oncol, 9 (2007) 29-38.
[5] K.R. Lamborn, W.K. Yung, S.M. Chang, P.Y. Wen, T.F. Cloughesy, L.M. DeAngelis, H.I. Robins, F.S. Lieberman, H.A. Fine, K.L. Fink, L. Junck, L. Abrey, M.R. Gilbert, M. Mehta, J.G. Kuhn, K.D. Aldape, J. Hibberts, P.M. Peterson, M.D. Prados, C. North American Brain Tumor, Progression-free survival: an important end point in evaluating therapy for recurrent high-grade gliomas, Neuro Oncol, 10 (2008) 162-170.
[6] W. Wu, K.R. Lamborn, J.C. Buckner, P.J. Novotny, S.M. Chang, J.R. O'Fallon, K.A. Jaeckle, M.D. Prados, Joint NCCTG and NABTC prognostic factors analysis for high-grade recurrent glioma, Neuro Oncol, 12 (2010) 164-172.
[7] M.L. Disis, Immune regulation of cancer, J Clin Oncol, 28 (2010) 4531-4538.
[8] R.H. Thompson, H. Dong, C.M. Lohse, B.C. Leibovich, M.L. Blute, J.C. Cheville, E.D. Kwon, PD-1 is expressed by tumor-infiltrating immune cells and is associated with poor outcome for patients with renal cell carcinoma, Clinical cancer research : an official journal of the American Association for Cancer Research, 13 (2007) 1757-1761.
[9] L.M. Francisco, P.T. Sage, A.H. Sharpe, The PD-1 pathway in tolerance and autoimmunity, Immunol Rev, 236 (2010) 219-242.
[10] J.A. Brown, D.M. Dorfman, F.R. Ma, E.L. Sullivan, O. Munoz, C.R. Wood, E.A. Greenfield, G.J. Freeman, Blockade of programmed death-1 ligands on dendritic cells enhances T cell activation and cytokine production, J Immunol, 170 (2003) 1257-1266.
[11] H. Dong, S.E. Strome, D.R. Salomao, H. Tamura, F. Hirano, D.B. Flies, P.C. Roche, J. Lu, G. Zhu, K. Tamada, V.A. Lennon, E. Celis, L. Chen, Tumor-associated B7-H1 promotes T-cell apoptosis: a potential mechanism of immune evasion, Nat Med, 8 (2002) 793-800.
[12] A.H. Sharpe, G.J. Freeman, The B7-CD28 superfamily, Nat Rev Immunol, 2 (2002) 116-126.
[13] J.E. Talmadge, M. Donkor, E. Scholar, Inflammatory cell infiltration of tumors: Jekyll or Hyde, Cancer metastasis reviews, 26 (2007) 373-400.
[14] A. Usubutun, A. Ayhan, M.C. Uygur, H. Ozen, C. Toklu, S. Ruacan, Prognostic factors in renal cell carcinoma, Journal of experimental & clinical cancer research : CR, 17 (1998) 77-81.

[15] E. Lazar-Molnar, Q. Yan, E. Cao, U. Ramagopal, S.G. Nathenson, S.C. Almo, Crystal structure of the complex between programmed death-1 (PD-1) and its ligand PD-L2, Proc Natl Acad Sci U S A, 105 (2008) 10483-10488.
[16] K.A. Sheppard, L.J. Fitz, J.M. Lee, C. Benander, J.A. George, J. Wooters, Y. Qiu, J.M. Jussif, L.L. Carter, C.R. Wood, D. Chaudhary, PD-1 inhibits T-cell receptor induced phosphorylation of the ZAP70/CD3zeta signalosome and downstream signaling to PKCtheta, FEBS Lett, 574 (2004) 37-41.
[17] J.M. Chemnitz, R.V. Parry, K.E. Nichols, C.H. June, J.L. Riley, SHP-1 and SHP-2 associate with immunoreceptor tyrosine-based switch motif of programmed death 1 upon primary human T cell stimulation, but only receptor ligation prevents T cell activation, J Immunol, 173 (2004) 945-954.
[18] K.M. Lee, E. Chuang, M. Griffin, R. Khattri, D.K. Hong, W. Zhang, D. Straus, L.E. Samelson, C.B. Thompson, J.A. Bluestone, Molecular basis of T cell inactivation by CTLA-4, Science, 282 (1998) 2263-2266.
[19] D.R. Plas, R. Johnson, J.T. Pingel, R.J. Matthews, M. Dalton, G. Roy, A.C. Chan, M.L. Thomas, Direct regulation of ZAP-70 by SHP-1 in T cell antigen receptor signaling, Science, 272 (1996) 1173-1176.
[20] R.V. Parry, J.M. Chemnitz, K.A. Frauwirth, A.R. Lanfranco, I. Braunstein, S.V. Kobayashi, P.S. Linsley, C.B. Thompson, J.L. Riley, CTLA-4 and PD-1 receptors inhibit T-cell activation by distinct mechanisms, Mol Cell Biol, 25 (2005) 9543-9553.
[21] L.V. Riella, A.M. Paterson, A.H. Sharpe, A. Chandraker, Role of the PD-1 pathway in the immune response, Am J Transplant, 12 (2012) 2575-2587.
[22] Y. Agata, A. Kawasaki, H. Nishimura, Y. Ishida, T. Tsubata, H. Yagita, T. Honjo, Expression of the PD-1 antigen on the surface of stimulated mouse T and B lymphocytes, Int Immunol, 8 (1996) 765-772.
[23] M. Kloor, Lymphocyte infiltration and prognosis in colorectal cancer, The Lancet. Oncology, 10 (2009) 840-841.
[24] F. Hillen, C.I. Baeten, A. van de Winkel, D. Creytens, D.W. van der Schaft, V. Winnepenninckx, A.W. Griffioen, Leukocyte infiltration and tumor cell plasticity are parameters of aggressiveness in primary cutaneous melanoma, Cancer immunology, immunotherapy : CII, 57 (2008) 97-106.
[25] H.E. Lee, S.W. Chae, Y.J. Lee, M.A. Kim, H.S. Lee, B.L. Lee, W.H. Kim, Prognostic implications of type and density of tumour-infiltrating lymphocytes in gastric cancer, British journal of cancer, 99 (2008) 1704-1711.
[26] N. Leffers, M.J. Gooden, R.A. de Jong, B.N. Hoogeboom, K.A. ten Hoor, H. Hollema, H.M. Boezen, A.G. van der Zee, T. Daemen, H.W. Nijman, Prognostic significance of tumor-infiltrating T-lymphocytes in primary and metastatic lesions of advanced stage ovarian cancer, Cancer immunology, immunotherapy : CII, 58 (2009) 449-459.
[27] H. Nishimura, T. Honjo, N. Minato, Facilitation of beta selection and modification of positive selection in the thymus of PD-1-deficient mice, The Journal of experimental medicine, 191 (2000) 891-898.
[28] T. Inozume, K. Hanada, Q.J. Wang, M. Ahmadzadeh, J.R. Wunderlich, S.A. Rosenberg, J.C. Yang, Selection of CD8+PD-1+ lymphocytes in fresh human melanomas enriches for tumor-reactive T cells, J Immunother, 33 (2010) 956-964.

[29] C. Lebbe, N. Meyer, L. Mortier, I. Marquez-Rodas, C. Robert, P. Rutkowski, A.M. Menzies, T. Eigentler, P.A. Ascierto, M. Smylie, D. Schadendorf, M. Ajaz, I.M. Svane, R. Gonzalez, L. Rollin, J. Lord-Bessen, A. Saci, E. Grigoryeva, J. Pigozzo, Evaluation of Two Dosing Regimens for Nivolumab in Combination With Ipilimumab in Patients With Advanced Melanoma: Results From the Phase IIIb/IV CheckMate 511 Trial, J Clin Oncol, 37 (2019) 867-875.
[30] L. Devine, P.B. Kavathas, Molecular analysis of protein interactions mediating the function of the cell surface protein CD8, Immunol Res, 19 (1999) 201-210.
[31] N. Pandit-Taskar, M. Postow, M. Hellmann, J. Harding, C. Barker, J. O'Donoghue, M. Ziolkowska, S. Ruan, S. Lyashchenko, F. Tsai, M. Farwell, T. Mitchell, R. Korn, W. Le, J. Lewis, W. Weber, D. Behera, I. Wilson, M. Gordon, A. Wu, J. Wolchok, First-in-human imaging with (89)Zr-Df-IAB22M2C anti-CD8 minibody in patients with solid malignancies: preliminary pharmacokinetics, biodistribution, and lesion targeting, J Nucl Med, (2019).
[32] S. Deorah, C.F. Lynch, Z.A. Sibenaller, T.C. Ryken, Trends in brain cancer incidence and survival in the United States: Surveillance, Epidemiology, and End Results Program, 1973 to 2001, Neurosurg Focus, 20 (2006) E1.
[33] J.H. Sampson, M.D. Gunn, P.E. Fecci, D.M. Ashley, Brain immunology and immunotherapy in brain tumours, Nat Rev Cancer, 20 (2020) 12-25.
[34] T.B. Davidson, A. Lee, M. Hsu, S. Sedighim, J. Orpilla, J. Treger, M. Mastall, S. Roesch, C. Rapp, M. Galvez, A. Mochizuki, J. Antonios, A. Garcia, N. Kotecha, N. Bayless, D. Nathanson, A. Wang, R. Everson, W.H. Yong, T.F. Cloughesy, L.M. Liau, C. Herold-Mende, R.M. Prins, Expression of PD-1 by T Cells in Malignant Glioma Patients Reflects Exhaustion and Activation, Clinical cancer research : an official journal of the American Association for Cancer Research, 25 (2019) 1913-1922.
[35] K. Woroniecka, P. Chongsathidkiet, K. Rhodin, H. Kemeny, C. Dechant, S.H. Farber, A.A. Elsamadicy, X. Cui, S. Koyama, C. Jackson, L.J. Hansen, T.M. Johanns, L. Sanchez-Perez, V. Chandramohan, Y.A. Yu, D.D. Bigner, A. Giles, P. Healy, G. Dranoff, K.J. Weinhold, G.P. Dunn, P.E. Fecci, T-Cell Exhaustion Signatures Vary with Tumor Type and Are Severe in Glioblastoma, Clinical cancer research : an official journal of the American Association for Cancer Research, (2018).
[36] T.F. Cloughesy, A.Y. Mochizuki, J.R. Orpilla, W. Hugo, A.H. Lee, T.B. Davidson, A.C. Wang, B.M. Ellingson, J.A. Rytlewski, C.M. Sanders, E.S. Kawaguchi, L. Du, G. Li, W.H. Yong, S.C. Gaffey, A.L. Cohen, I.K. Mellinghoff, E.Q. Lee, D.A. Reardon, B.J. O'Brien, N.A. Butowski, P.L. Nghiemphu, J.L. Clarke, I.C. Arrillaga-Romany, H. Colman, T.J. Kaley, J.F. de Groot, L.M. Liau, P.Y. Wen, R.M. Prins, Neoadjuvant anti-PD-1 immunotherapy promotes a survival benefit with intratumoral and systemic immune responses in recurrent glioblastoma, Nat Med, 25 (2019) 477-486.
[37] R.N. Amaria, A.M. Menzies, E.M. Burton, R.A. Scolyer, M.T. Tetzlaff, R. Antdbacka, C. Ariyan, R. Bassett, B. Carter, A. Daud, M. Faries, L.A. Fecher, K.T. Flaherty, J.E. Gershenwald, O. Hamid, A. Hong, J.M. Kirkwood, S. Lo, K. Margolin, J. Messina, M.A. Postow, H. Rizos, M.I. Ross, E.A. Rozeman, R.P.M. Saw, V. Sondak, R.J. Sullivan, J.M. Taube, J.F. Thompson, B.A. van de Wiel, A.M. Eggermont, M.A. Davies, m. International Neoadjuvant Melanoma Consortium, P.A. Ascierto, A.J. Spillane, A.C.J. van Akkooi, J.A. Wargo, C.U. Blank, H.A. Tawbi, G.V. Long, Neoadjuvant systemic therapy in melanoma: recommendations of the International Neoadjuvant Melanoma Consortium, The Lancet. Oncology, 20 (2019) e378-e389.

[38] J.S. O'Donnell, E.P. Hoefsmit, M.J. Smyth, C.U. Blank, M.W.L. Teng, The Promise of Neoadjuvant Immunotherapy and Surgery for Cancer Treatment, Clinical cancer research : an official journal of the American Association for Cancer Research, (2019).
[39] H.S. Robins, N.G. Ericson, J. Guenthoer, K.C. O'Briant, M. Tewari, C.W. Drescher, J.H. Bielas, Digital genomic quantification of tumor-infiltrating lymphocytes, Sci Transl Med, 5 (2013) 214ra169.
[40] M.S. Hsu, S. Sedighim, T. Wang, J.P. Antonios, R.G. Everson, A.M. Tucker, L. Du, R. Emerson, E. Yusko, C. Sanders, H.S. Robins, W.H. Yong, T.B. Davidson, G. Li, L.M. Liau, R.M. Prins, TCR Sequencing Can Identify and Track Glioma-Infiltrating T Cells after DC Vaccination, Cancer Immunol Res, 4 (2016) 412-418.
[41] I. Datar, M.F. Sanmamed, J. Wang, B.S. Henick, J. Choi, T. Badri, W. Dong, N. Mani, M. Toki, L.D. Mejias, M.D. Lozano, J.L. Perez-Gracia, V. Velcheti, M.D. Hellmann, J.F. Gainor, K. McEachern, D. Jenkins, K. Syrigos, K. Politi, S. Gettinger, D.L. Rimm, R.S. Herbst, I. Melero, L. Chen, K.A. Schalper, Expression Analysis and Significance of PD-1, LAG-3, and TIM-3 in Human Non-Small Cell Lung Cancer Using Spatially Resolved and Multiparametric Single-Cell Analysis, Clinical cancer research : an official journal of the American Association for Cancer Research, (2019).
[42] Z. Feng, S. Puri, T. Moudgil, W. Wood, C.C. Hoyt, C. Wang, W.J. Urba, B.D. Curti, C.B. Bifulco, B.A. Fox, Multispectral imaging of formalin-fixed tissue predicts ability to generate tumor-infiltrating lymphocytes from melanoma, J Immunother Cancer, 3 (2015) 47.
[43] G. Madonna, C. Ballesteros-Merino, Z. Feng, C. Bifulco, M. Capone, D. Giannarelli, D. Mallardo, E. Simeone, A.M. Grimaldi, C. Caraco, G. Botti, B.A. Fox, P.A. Ascierto, PD-L1 expression with immune-infiltrate evaluation and outcome prediction in melanoma patients treated with ipilimumab, Oncoimmunology, 7 (2018) e1405206.
[44] P.C. Tumeh, C.L. Harview, J.H. Yearley, I.P. Shintaku, E.J. Taylor, L. Robert, B. Chmielowski, M. Spasic, G. Henry, V. Ciobanu, A.N. West, M. Carmona, C. Kivork, E. Seja, G. Cherry, A.J. Gutierrez, T.R. Grogan, C. Mateus, G. Tomasic, J.A. Glaspy, R.O. Emerson, H. Robins, R.H. Pierce, D.A. Elashoff, C. Robert, A. Ribas, PD-1 blockade induces responses by inhibiting adaptive immune resistance, Nature, 515 (2014) 568-571.
[45] C. Wickenhauser, D. Bethmann, Z. Feng, S.M. Jensen, C. Ballesteros-Merino, C. Massa, A. Steven, M. Bauer, P. Kaatzsch, N. Pazaitis, G. Toma, C.B. Bifulco, B.A. Fox, B. Seliger, Multispectral Fluorescence Imaging Allows for Distinctive Topographic Assessment and Subclassification of Tumor-Infiltrating and Surrounding Immune Cells, Methods Mol Biol, 1913 (2019) 13-31.
[46] M. Ayers, J. Lunceford, M. Nebozhyn, E. Murphy, A. Loboda, D.R. Kaufman, A. Albright, J.D. Cheng, S.P. Kang, V. Shankaran, S.A. Piha-Paul, J. Yearley, T.Y. Seiwert, A. Ribas, T.K. McClanahan, IFN-gamma-related mRNA profile predicts clinical response to PD-1 blockade, J Clin Invest, 127 (2017) 2930-2940.
[47] K.A. Schalper, M.E. Rodriguez-Ruiz, R. Diez-Valle, A. Lopez-Janeiro, A. Porciuncula, M.A. Idoate, S. Inoges, C. de Andrea, A. Lopez-Diaz de Cerio, S. Tejada, P. Berraondo, F. Villarroel-Espindola, J. Choi, A. Gurpide, M. Giraldez, I. Goicoechea, J. Gallego Perez-Larraya, M.F. Sanmamed, J.L. Perez-Gracia, I. Melero, Neoadjuvant nivolumab modifies the tumor immune microenvironment in resectable glioblastoma, Nat Med, 25 (2019) 470-476.
[48] R.N. Amaria, S.M. Reddy, H.A. Tawbi, M.A. Davies, M.I. Ross, I.C. Glitza, J.N. Cormier, C. Lewis, W.J. Hwu, E. Hanna, A. Diab, M.K. Wong, R. Royal, N. Gross, R. Weber, S.Y. Lai, R.

Ehlers, J. Blando, D.R. Milton, S. Woodman, R. Kageyama, D.K. Wells, P. Hwu, S.P. Patel, A. Lucci, A. Hessel, J.E. Lee, J. Gershenwald, L. Simpson, E.M. Burton, L. Posada, L. Haydu, L. Wang, S. Zhang, A.J. Lazar, C.W. Hudgens, V. Gopalakrishnan, A. Reuben, M.C. Andrews, C.N. Spencer, V. Prieto, P. Sharma, J. Allison, M.T. Tetzlaff, J.A. Wargo, Neoadjuvant immune checkpoint blockade in high-risk resectable melanoma, Nat Med, 24 (2018) 1649-1654.
[49] C.U. Blank, E.A. Rozeman, L.F. Fanchi, K. Sikorska, B. van de Wiel, P. Kvistborg, O. Krijgsman, M. van den Braber, D. Philips, A. Broeks, J.V. van Thienen, H.A. Mallo, S. Adriaansz, S. Ter Meulen, L.M. Pronk, L.G. Grijpink-Ongering, A. Bruining, R.M. Gittelman, S. Warren, H. van Tinteren, D.S. Peeper, J. Haanen, A.C.J. van Akkooi, T.N. Schumacher, Neoadjuvant versus adjuvant ipilimumab plus nivolumab in macroscopic stage III melanoma, Nat Med, 24 (2018) 1655-1661.
[50] P.M. Forde, J.E. Chaft, K.N. Smith, V. Anagnostou, T.R. Cottrell, M.D. Hellmann, M. Zahurak, S.C. Yang, D.R. Jones, S. Broderick, R.J. Battafarano, M.J. Velez, N. Rekhtman, Z. Olah, J. Naidoo, K.A. Marrone, F. Verde, H. Guo, J. Zhang, J.X. Caushi, H.Y. Chan, J.W. Sidhom, R.B. Scharpf, J. White, E. Gabrielson, H. Wang, G.L. Rosner, V. Rusch, J.D. Wolchok, T. Merghoub, J.M. Taube, V.E. Velculescu, S.L. Topalian, J.R. Brahmer, D.M. Pardoll, Neoadjuvant PD-1 Blockade in Resectable Lung Cancer, The New England journal of medicine, (2018).
[51] E. Cha, M. Klinger, Y. Hou, C. Cummings, A. Ribas, M. Faham, L. Fong, Improved survival with T cell clonotype stability after anti-CTLA-4 treatment in cancer patients, Sci Transl Med, 6 (2014) 238ra270.
[52] A. Ribas, Adaptive Immune Resistance: How Cancer Protects from Immune Attack, Cancer Discov, 5 (2015) 915-919.
[53] L. Robert, J. Tsoi, X. Wang, R.O. Emerson, B. Homet, T. Chodon, S. Mok, R.R. Huang, A.J. Cochran, B. Comin-Anduix, R.C. Koya, T. Graeber, H.S. Robins, A. Ribas, CTLA4 blockade broadens the peripheral T cell receptor repertoire, Clinical cancer research : an official journal of the American Association for Cancer Research, (2014).
[54] J.D. Wolchok, H. Kluger, M.K. Callahan, M.A. Postow, N.A. Rizvi, A.M. Lesokhin, N.H. Segal, C.E. Ariyan, R.A. Gordon, K. Reed, M.M. Burke, A. Caldwell, S.A. Kronenberg, B.U. Agunwamba, X. Zhang, I. Lowy, H.D. Inzunza, W. Feely, C.E. Horak, Q. Hong, A.J. Korman, J.M. Wigginton, A. Gupta, M. Sznol, Nivolumab plus ipilimumab in advanced melanoma, The New England journal of medicine, 369 (2013) 122-133.
[55] A. Omuro, G. Vlahovic, M. Lim, S. Sahebjam, J. Baehring, T. Cloughesy, A. Voloschin, S.H. Ramkissoon, K.L. Ligon, R. Latek, R. Zwirtes, L. Strauss, P. Paliwal, C.T. Harbison, D.A. Reardon, J.H. Sampson, Nivolumab with or without ipilimumab in patients with recurrent glioblastoma: results from exploratory phase I cohorts of CheckMate 143, Neuro Oncol, 20 (2018) 674-686.
[56] D.J. Brat, K. Aldape, H. Colman, E.C. Holland, D.N. Louis, R.B. Jenkins, B.K. Kleinschmidt-DeMasters, A. Perry, G. Reifenberger, R. Stupp, A. von Deimling, M. Weller, cIMPACT-NOW update 3: recommended diagnostic criteria for "Diffuse astrocytic glioma, IDH-wildtype, with molecular features of glioblastoma, WHO grade IV", Acta Neuropathol, 136 (2018) 805-810.
[57] J.D. Wolchok, A. Hoos, S. O'Day, J.S. Weber, O. Hamid, C. Lebbe, M. Maio, M. Binder, O. Bohnsack, G. Nichol, R. Humphrey, F.S. Hodi, Guidelines for the evaluation of immune therapy activity in solid tumors: immune-related response criteria, Clinical cancer research : an official journal of the American Association for Cancer Research, 15 (2009) 7412-7420.

[58] D.R. Macdonald, T.L. Cascino, S.C. Schold, Jr., J.G. Cairncross, Response criteria for phase II studies of supratentorial malignant glioma, Journal of Clinical Oncology, 8 (1990) 1277-1280.
[59] A.A. Brandes, A. Tosoni, F. Spagnolli, G. Frezza, M. Leonardi, F. Calbucci, E. Franceschi, Disease progression or pseudoprogression after concomitant radiochemotherapy treatment: pitfalls in neurooncology, Neuro Oncol, 10 (2008) 361-367.
[60] D. Brandsma, L. Stalpers, W. Taal, P. Sminia, M.J. van den Bent, Clinical features, mechanisms, and management of pseudoprogression in malignant gliomas, The Lancet. Oncology, 9 (2008) 453-461.
[61] O. Hamid, C. Robert, A. Daud, F.S. Hodi, W.J. Hwu, R. Kefford, J.D. Wolchok, P. Hersey, R.W. Joseph, J.S. Weber, R. Dronca, T.C. Gangadhar, A. Patnaik, H. Zarour, A.M. Joshua, K. Gergich, J. Elassaiss-Schaap, A. Algazi, C. Mateus, P. Boasberg, P.C. Tumeh, B. Chmielowski, S.W. Ebbinghaus, X.N. Li, S.P. Kang, A. Ribas, Safety and tumor responses with lambrolizumab (anti-PD-1) in melanoma, The New England journal of medicine, 369 (2013) 134-144.
[62] J.R. Brahmer, S.S. Tykodi, L.Q. Chow, W.J. Hwu, S.L. Topalian, P. Hwu, C.G. Drake, L.H. Camacho, J. Kauh, K. Odunsi, H.C. Pitot, O. Hamid, S. Bhatia, R. Martins, K. Eaton, S. Chen, T.M. Salay, S. Alaparthy, J.F. Grosso, A.J. Korman, S.M. Parker, S. Agrawal, S.M. Goldberg, D.M. Pardoll, A. Gupta, J.M. Wigginton, Safety and activity of anti-PD-L1 antibody in patients with advanced cancer, The New England journal of medicine, 366 (2012) 2455-2465.
[63] S.L. Topalian, F.S. Hodi, J.R. Brahmer, S.N. Gettinger, D.C. Smith, D.F. McDermott, J.D. Powderly, R.D. Carvajal, J.A. Sosman, M.B. Atkins, P.D. Leming, D.R. Spigel, S.J. Antonia, L. Horn, C.G. Drake, D.M. Pardoll, L. Chen, W.H. Sharfman, R.A. Anders, J.M. Taube, T.L. McMiller, H. Xu, A.J. Korman, M. Jure-Kunkel, S. Agrawal, D. McDonald, G.D. Kollia, A. Gupta, J.M. Wigginton, M. Sznol, Safety, activity, and immune correlates of anti-PD-1 antibody in cancer, The New England journal of medicine, 366 (2012) 2443-2454.
[64] J.L. Clarke, M.M. Ennis, W.K. Yung, S.M. Chang, P.Y. Wen, T.F. Cloughesy, L.M. Deangelis, H.I. Robins, F.S. Lieberman, H.A. Fine, L. Abrey, M.R. Gilbert, M. Mehta, J.G. Kuhn, K.D. Aldape, K.R. Lamborn, M.D. Prados, Is surgery at progression a prognostic marker for improved 6-month progression-free survival or overall survival for patients with recurrent glioblastoma?, Neuro-oncology, (2011).

APPENDIX A KARNOFSKY PERFORMANCE STATUS

Grade	Description
100	Normal, no complaints; no evidence of disease
90	Able to carry on normal activity; minor signs or symptoms of disease.
80	Normal activity with effort; some signs or symptoms of disease.
70	Cares for self; unable to carry on normal activity or to do active work.
60	Requires occasional assistance, but is able to care for most of his personal needs.
50	Requires considerable assistance and frequent medical care.
40	Disabled; requires special care and assistance.
30	Severely disabled; hospital admission is indicated although death not imminent.
20	Very sick; hospital admission necessary; active supportive treatment necessary.
10	Moribund; fatal processes progressing rapidly.
0	Dead.
* Schag CC, Heinrich RL, Ganz PA. Karnofsky performance status revisited: Reliability, validity and guidelines. J Clin Oncol 2:187-193, 1984.	

APPENDIX B DF/HCC MULTI-CENTER DATA AND SAFETY MONITORING PLAN

DF/HCC Protocol #: 20-494

Dana-Farber/Harvard Cancer Center
Multi-Center Data and Safety Monitoring Plan

TABLE OF CONTENTS

1.0 INTRODUCTION 98
1.1 Purpose 98

2.0 GENERAL ROLES AND RESPONSIBILITIES 98
2.1 Coordinating Center 98
2.2 External Site 99

3.0 DF/HCC REQUIREMENTS FOR MULTI-CENTER PROTOCOLS 99
3.1 Protocol Revisions and Closures 100
3.2 Informed Consent Requirements 100
3.3 IRB Re-Approval 100
3.4 DF/HCC Multi-Center Protocol Confidentiality 101
3.5 Participant Registration and Randomization 101
3.6 Data Management 102
3.7 Protocol Reporting Requirements 102

4.0 MONITORING: QUALITY CONTROL 103
4.1 Ongoing Monitoring of Protocol Compliance 103
4.2 Monitoring Reports 105
4.3 Accrual Monitoring 105

5.0 AUDITING: QUALITY ASSURANCE 105
5.1 DF/HCC Internal Audits 105
5.2 Audit Notification 105
5.3 Audit Reports 105
5.4 External Site Performance 105

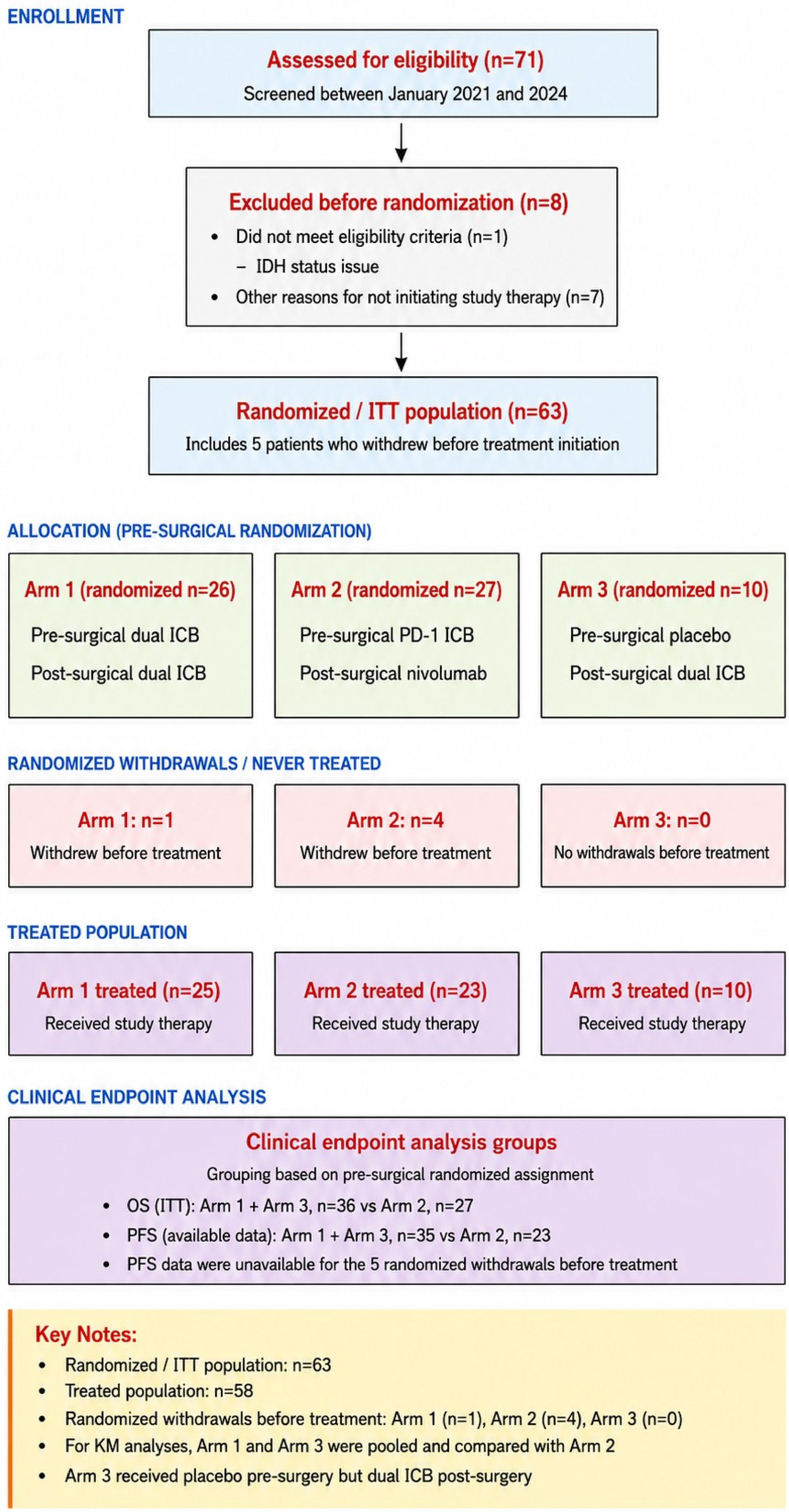

Supplementary Figure 10 CONSORT flow diagram: Dual immune checkpoint blockade in recurrent glioblastoma (NCT04606316).

1.0 INTRODUCTION

The Dana-Farber/Harvard Cancer Center Multi-Center Data and Safety Monitoring Plan (DF/HCC DSMP) outlines the procedures for conducting a DF/HCC Multi-Center research protocol. The DF/HCC DSMP serves as a reference for any sites external to DF/HCC that are participating in a DF/HCC clinical trial.

1.1 Purpose

To establish standards that will ensure that a Dana-Farber/Harvard Cancer Center Multi-Center protocol will comply with Federal Regulations, Health Insurance Portability and Accountability Act (HIPAA) requirements and applicable DF/HCC Policies and Operations.

2.0 GENERAL ROLES AND RESPONSIBILITIES

For DF/HCC Multi-Center Protocols, the following general responsibilities apply, in addition to those outlined in DF/HCC Policies for Sponsor-Investigators:

2.1 Coordinating Center

The Coordinating Center is the entity that provides administrative support to the DF/HCC Sponsor in order that he/she may fulfill the responsibilities outlined in the protocol document and DSMP, and as specified in applicable regulatory guidelines (i.e. CTEP Multi-Center Guidelines).

The general responsibilities of the Coordinating Center may include but are not limited to:

- Assist in protocol development
- Maintain FDA correspondence, as applicable.
- Review registration materials for eligibility and register participants from Participating Institutions in the DF/HCC clinical trial management system (CTMS).
- Distribute protocol and informed consent document updates to External Sites as needed.
- Oversee the data collection process from External Sites.
- Maintain documentation of Serious Adverse Event (SAE) reports and deviations/violations submitted by External Sites and provide to the DF/HCC Sponsor for timely review and submission to the IRB of record, as necessary.
- Distribute serious adverse events reported to the DF/HCC Sponsor that fall under the reporting requirements for the IRB of record to all External Sites.
- Provide External Sites with information regarding DF/HCC requirements that they will be expected to comply with.
- Carry out plan to monitor External Sites either by on-site or remote monitoring.
- Maintain Regulatory documents of all External Sites which includes but is not limited to the following: local IRB approvals/notifications from all External Sites,

confirmation of Federalwide Assurances (FWAs) for all sites, all SAE submissions, Screening Logs for all sites, IRB approved consents for all sites.
- Conduct regular communications with all External Sites (conference calls, emails, etc.) and maintain documentation of all relevant communications.

2.2 External Site

An External Site is an institution that is outside the DF/HCC and DF/PCC consortium that is collaborating with DF/HCC on a protocol where the sponsor is a DF/HCC investigator. The External Site acknowledges the DF/HCC Sponsor as having the ultimate authority and responsibility for the overall conduct of the study.

Each External Site is expected to comply with all applicable DF/HCC requirements stated within this Data and Safety Monitoring Plan and/or the protocol document.

The general responsibilities for each External Site may include but are not limited to:
- Document the delegation of research specific activities to study personnel.
- Commit to the accrual of participants to the protocol.
- Submit protocol and/or amendments to their IRB of record. For studies under a single IRB, the Coordinating Center will facilitate any study-wide submissions.
- Maintain regulatory files as per ICH GCP and federal requirements.
- Provide the Coordinating Center with regulatory documents or source documents as requested.
- Participate in protocol training prior to enrolling participants and throughout the trial as required.
- Update Coordinating Center with research staff changes on a timely basis.
- Register participants through the Coordinating Center prior to beginning research related activities when required by the sponsor.
- Submit Serious Adverse Event (SAE) reports to sponsor, Coordinating Center, and IRB of record as applicable in accordance with DF/HCC requirements.
- Submit protocol deviations and violations to the Sponsor, Coordinating Center, and IRB of record as applicable.
- *Order, store and dispense investigational agents and/or other protocol mandated drugs per federal guidelines and protocol requirements.*
- Participate in any quality assurance activities and meet with monitors or auditors at the conclusion of a visit to review findings.
- Promptly provide follow-up and/or corrective action plans for any monitoring queries or audit findings.
- Notify the sponsor immediately of any regulatory authority inspection of this protocol at the External Site

3.0 DF/HCC REQUIREMENTS FOR MULTI-CENTER PROTOCOLS

Certain DF/HCC Policy requirements apply to External Sites participating in DF/HCC

research. The following section will clarify DF/HCC requirements and further detail the expectations for participating in a DF/HCC Multi-Center protocol.

3.1 Protocol Revisions and Closures

The External Sites will receive notification of protocol revisions and closures from the Coordinating Center. When under a separate IRB, it is the individual External Site's responsibility to notify its IRB of these revisions.

- ***Protocol revisions:*** *External Sites will receive written notification of protocol revisions from the Coordinating Center. All protocol revisions should be IRB approved and implemented within a timely manner from receipt of the notification.*

- **Protocol closures and temporary holds:** External Sites will receive notification of protocol closures and temporary holds from the Coordinating Center. Closures and holds will be effective immediately. In addition, the Coordinating Center, will update the External Sites on an ongoing basis about protocol accrual data so that they will be aware of imminent protocol closures.

3.2 Informed Consent Requirements

The DF/HCC approved informed consent document will serve as a template for the informed consent for External Sites. The External Site consent form must follow the consent template as closely as possible and should adhere to specifications outlined in the DF/HCC Guidance Document on Model Consent Language for Investigator-Sponsored Multi-Center Trials. This document will be provided separately to each External Site upon request.

External Sites must send their version of the informed consent document to the Coordinating Center for sponsor review and approval. If the HIPAA authorization is a separate document, please submit to the sponsor for the study record. Once sponsor approval is obtained, the External site may submit to their IRB of record, as applicable. In these cases, the approved consent form must also be submitted to the Coordinating Center after approval by the local IRB for all consent versions.

The Principal Investigator (PI) at each External Site will identify the appropriate members of the study team who will be obtaining consent and signing the consent form for protocols. External Sites must follow the DF/HCC requirement that for all interventional drug, biologic, or device research, only attending physicians may obtain initial informed consent and any re-consent that requires a full revised consent form.

3.3 IRB Re-Approval

Verification of IRB re-approval for the External Sites is required in order to continue research activities. There is no grace period for continuing approvals.

The Coordinating Center will not register participants if a re-approval letter is not received for the External Site on or before the anniversary of the previous approval date.

3.4 DF/HCC Multi-Center Protocol Confidentiality

All documents, investigative reports, or information relating to the participant are strictly confidential. Whenever reasonably feasible, any participant specific reports (i.e. Pathology Reports, MRI Reports, Operative Reports, etc.) submitted to the Coordinating Center should be de-identified. It is recommended that the assigned protocol case number be used for all participant specific documents. Participant initials may be included or retained for cross verification of identification

3.5 Participant Registration and Randomization

To register a participant, the following documents should be completed by the External Site and e-mailed to the Coordinating Center at NeuroOnc_Coor@dfci.harvard.edu:

- Completed DF/HCC study specific Eligibility Checklist
- Protocol required test results required at screening for eligibility
(e.g. coagulation, virology, UA, thyroid, CBC w/ diff, CMP, and BHCG)
- Pathology and surgical reports
- Clinic note(s) and/or other medical records documenting consent process, screening, and eligibility
- List of current concomitant medications (obtained within the protocol-specified screening window) signed and dated by RN/other clinician and documentation of when reviewed/confirmed with patient)
- Signed informed consent document
- Signed HIPAA authorization form (if separate from the informed consent document)

The Coordinating Center will review the submitted documents in order to verify eligibility and consent. To complete the registration process, the Coordinating Center will:

- Register the participant on the study with the DF/HCC Clinical Trial Management System (CTMS).
- Upon receiving confirmation of registration, the Coordinating Center will inform the External Site and provide the study specific participant case number, and, if applicable, assigned treatment and/or dose level.
- The assigned treatment arm will only be communicated with the previously designated unblinded Site Investigational Pharmacist due to the blinding of the neoadjuvant treatment portion of the study.

At the time of registration, the following identifiers are required for all subjects: initials, date of birth, gender, race, and ethnicity. Once eligibility has been established and the participant successfully registered, the participant is assigned a unique protocol case number. External Sites should submit all de-identified subsequent communication

and documents to the Coordinating Center, using this case number to identify the subject.

Randomization can only occur during normal business hours, Monday through Friday from 8:00 AM to 5:00 PM Eastern Standard Time.

Initiation of Therapy

Participants must be registered with the DF/HCC CTMS before the initiation of treatment or other protocol-specific interventions. Treatment and other protocol-specific interventions may not be initiated until the External Site receives confirmation of the participant's registration from the Coordinating Center. The DF/HCC Sponsor and IRB of record must be notified of any violations to this policy.

Eligibility Exceptions

No exceptions to the eligibility requirements for a protocol without IRB approval will be permitted. All External Sites are required to fully comply with this requirement.

3.6 Data Management

DF/HCC develops case report forms (CRF/eCRFs), for use with the protocol. These forms are designed to collect data for the study. The DF/HCC provides a web-based training for eCRF users.

Data Forms Review

Data submissions are monitored for timeliness and completeness of submission. If study forms are received with missing or questionable data, the submitting institution will receive a written or electronic query from the DF/HCC Office of Data Quality, Coordinating Center, or designee.

Responses to all queries should be completed and submitted within 14 calendar days.

If study forms are not submitted on schedule, the External Sites will periodically receive a Missing Form Report from the Coordinating Center noting the missing forms.

3.7 Protocol Reporting Requirements

Protocol Deviations, Exceptions, and Violations

Federal Regulations require an IRB to review proposed changes in a research activity to ensure that researchers do not initiate changes in approved research without IRB review and approval, except when necessary to eliminate apparent immediate hazards to the

participant. DF/HCC requires all departures from the defined procedures set forth in the IRB approved protocol to be reported to the DF/HCC Sponsor and to the IRB of record.

Reporting Procedures

Request to deviate from the protocol require approval from the IRB of record and the sponsor.

All protocol violations must be sent to the Coordinating Center in a timely manner. The Coordinating Center will provide training for the requirements for the reporting of violations.

Guidelines for Processing IND Safety Reports

The DF/HCC Sponsor will review all IND Safety Reports per DF/HCC requirements, and ensure that all IND Safety Reports are distributed to the External Sites as required by DF/HCC Policy. External Sites will review/submit to the IRB according to their institutional policies and procedures.

4.0 MONITORING: QUALITY CONTROL

The Coordinating Center, with the aid of the DF/HCC Office of Data Quality, provides quality control oversight for the protocol.

4.1 Ongoing Monitoring of Protocol Compliance

The External Sites may be required to submit participant source documents to the Coordinating Center for monitoring. External Sites may also be subject to on-site monitoring conducted by the Coordinating Center.

The Coordinating Center will implement ongoing monitoring activities to ensure that External Sites are complying with regulatory and protocol requirements, data quality, and participant safety. Monitoring practices may include but are not limited to source data verification, and review and analysis of eligibility requirements, informed consent procedures, adverse events and all associated documentation, review of study drug administration/treatment, regulatory files, protocol departures, pharmacy records, response assessments, and data management.

Interim monitoring visits will occur on the following schedule:

- Once a site has registered a participant, up until all participants (and planned participants) have discontinued taking study agent (may be in follow-up), interim monitoring visits will occur at least twice per year, either on-site or virtually. The first interim monitoring visit will occur approximately two months after the registration of the site’s first participant.

- Once a site is closed to accrual and all participants have discontinued study agent, interim monitoring visits will occur virtually or on-site, as needed.

On-site Monitoring. On-site monitoring will occur on a regular basis. Participating Institutions will be required to provide access to participants' complete medical record and source documents for source documentation verification during the on-site visit. In addition, upon request from a monitor or auditor, Participating Institutions should provide access to regulatory documents, pharmacy records, local policies related to the conduct of research, and any other trial-related documentation maintained by the participating site. If there are concerns for protocol compliance, issues that impact participant safety or the integrity of the study are found, or trends identified based on areas of need, additional monitoring visits may be scheduled.

Virtual Monitoring: The Coordinating Center will request source documentation from participating Institutions as needed to complete monitoring activities. Because of limited on-site monitoring visits, Participating Sites will be required to submit (either electronically or via paper) requested source documents to the Coordinating Center for source verification.

Teleconferences: The Coordinating Center will host all-site teleconferences on a monthly basis and more frequently as needed. During the teleconferences, sites will be expected to convey the following information:

- Participant updates: including holds, dose reductions, significant events, status updates, and dates of progression or death as applicable
- Protocol status: the version is being used, the status of any amendments, etc.
- Any new reportable AEs or deviations/violations that have yet to be communicated to the sponsor team (informing the sponsor should not wait for the call, and the call does not supplant communicating the events via the regular email methods of communication).
- Review of prospective participants

If sites are not able to have a representative participant, they should email this information.

During the teleconferences, the Coordinating Center Specialist may discuss any or all of the following:

- Accrual updates
- Pending amendments or other study updates
- Safety Reports recently circulated or pending circulation
- Date updates including percentage of missing forms and/or open queries
- To be updated at least every three months: for participants in follow up, the date of their last follow-up.
- Review of recent deviations and/or violations
- Review of reportable AEs either newly received or pending resolution

4.2 Monitoring Reports

The DF/HCC Sponsor will review all monitoring reports to ensure protocol compliance. The DF/HCC Sponsor may increase the monitoring activities at External Sites that are unable to comply with the protocol, DF/HCC Sponsor requirements or federal and local regulations.

4.3 Accrual Monitoring

Prior to extending a protocol to an external site, the DF/HCC Sponsor will establish accrual requirements for each participating institution. Accrual will be monitored for each External Site by the DF/HCC Sponsor or designee. Sites that are not meeting their accrual expectations may be subject to termination.

Participating Institutions accrual requirement of 3 participants per site annually will be implemented.

5.0 AUDITING: QUALITY ASSURANCE

5.1 DF/HCC Internal Audits

All External Sites are subject to audit by the DF/HCC Office of Data Quality (ODQ). Typically, approximately 3-4 participants would be audited at the site over a 2-day period. If violations which impact participant safety or the integrity of the study are found, more participant records may be audited.

5.2 Audit Notification

It is the External Site’s responsibility to notify the Coordinating Center of all external audits or inspections (e.g., FDA, EMA, NCI) that involve this protocol. All institutions will forward a copy of final audit and/or re-audit reports and corrective action plans (if applicable) to the Coordinating Center, within 12 weeks after the audit date.

5.3 Audit Reports

The DF/HCC Sponsor will review all final audit reports and corrective action plans, if applicable. The Coordinating Center must forward these reports to the DF/HCC ODQ per DF/HCC policy for review by the DF/HCC Audit Committee. For unacceptable audits, the DF/HCC Audit Committee would forward the final audit report and corrective action plan to the DFCI IRB as applicable.

5.4 External Site Performance

The DF/HCC Sponsor and the IRB of record are charged with considering the totality of an institution’s performance in considering institutional participation in the protocol.

External Sites that fail to meet the performance goals of accrual, submission of timely and accurate data, adherence to protocol requirements, and compliance with state and federal regulations, may be put on hold or closed.

APPENDIX C MANAGEMENT ALGORITHMS

Nivolumab

These general guidelines constitute guidance to the Investigator and may be supplemented by discussions with the Sponsor-Investigator. The guidance was designed for all immuno-oncology (I-O) agents and regimens, and – for the purpose of this trial – will be utilized for nivolumab.

• Where applicable the Approved Label should be used for guidance around dose modifications and discontinuation

• A general principle is that differential diagnoses should be diligently evaluated according to standard medical practice. Non-inflammatory etiologies should be considered and appropriately treated.

• Corticosteroids are a primary therapy for immuno-oncology drug-related adverse events. The oral equivalent of the recommended IV doses may be considered for ambulatory patients with low-grade toxicity. The lower bioavailability of oral corticosteroids should be taken into account when switching to the equivalent dose of oral corticosteroids.

• Consultation with a medical or surgical specialist, especially prior to an invasive diagnostic or therapeutic procedure, is recommended.

• The frequency and severity of the related adverse events covered by these algorithms will depend on the immuno-oncology agent or regimen being used.

• Investigators should refer to the most current version of the IB or Approved Label for current recommendations for management of a specific Adverse Event of interest.

• On the next 7 pages, find nivolumab Management Algorithms for the following AEs:
o GI AEs
o Renal AEs
o Pulmonary AEs
o Hepatic AEs
o Endocrinopathy
o Skin AEs
o Neurological AEs

GI Adverse Event Management Algorithm

Rule out non-inflammatory causes. If non-inflammatory cause is identified, treat accordingly and continue I-O therapy. Opiates/narcotics may mask symptoms of perforation. Infliximab should not be used in cases of perforation or sepsis.

Grade of Diarrhea/ Colitis (NCI CTCAE v4)	Management	Follow-up
**Grade 1**<br>Diarrhea: < 4 stools/day over baseline;<br>Colitis: asymptomatic	• Continue I-O therapy per protocol<br>• Symptomatic treatment	• Close monitoring for worsening symptoms.<br>• Educate patient to report worsening immediately<br>If worsens:<br>• Treat as Grade 2 or 3/4
**Grade 2**<br>Diarrhea: 4-6 stools per day over baseline; IV fluids indicated <24 hrs; not interfering with ADL<br>Colitis: abdominal pain; blood in stool	• Delay I-O therapy per protocol<br>• Symptomatic treatment	If improves to grade 1:<br>• Resume I-O therapy per protocol<br>If persists > 5-7 days or recur:<br>• 0.5-1.0 mg/kg/day methylprednisolone or oral equivalent<br>• When symptoms improve to grade 1, taper steroids over at least 1 month, consider prophylactic antibiotics for opportunistic infections, and resume I-O therapy per protocol.<br>If worsens or persists > 3-5 days with oral steroids:<br>• Treat as grade 3/4
**Grade 3-4**<br>Diarrhea (G3): ≥7 stools per day over baseline; incontinence; IV fluids ≥24 hrs; interfering with ADL<br>Colitis (G3): severe abdominal pain, medical intervention indicated, peritoneal signs<br>G4: life-threatening, perforation	• Discontinue I-O therapy per protocol<br>• 1.0 to 2.0 mg/kg/day methylprednisolone IV or IV equivalent<br>• Add prophylactic antibiotics for opportunistic infections<br>• Consider lower endoscopy	If improves:<br>• Continue steroids until grade 1, then taper over at least 1 month<br>If persists > 3-5 days, or recurs after improvement:<br>• Add infliximab 5 mg/kg (if no contraindication). Note: Infliximab should not be used in cases of perforation or sepsis

Patients on IV steroids may be switched to an equivalent dose of oral corticosteroids (e.g. prednisone) at start of tapering or earlier, once sustained clinical improvement is observed. Lower bioavailability of oral corticosteroids should be taken into account when switching to the equivalent dose of oral corticosteroids.

Renal Adverse Event Management Algorithm

Rule out non-inflammatory causes. If non-inflammatory cause, treat accordingly and continue I-O therapy

Grade of Creatinine Elevation (NCI CTCAE v4)	Management	Follow-up
**Grade 1**<br>Creatinine > ULN and > than baseline but ≤ 1.5x baseline	• Continue I-O therapy per protocol<br>• Monitor creatinine weekly	If returns to baseline:<br>•Resume routine creatinine monitoring per protocol<br>If worsens:<br>•Treat as Grade 2 or 3/4
**Grade 2-3**<br>Creatinine > 1.5x baseline to ≤ 6x ULN	• Delay I-O therapy per protocol<br>• Monitor creatinine every 2-3 days<br>• 0.5 to 1.0 mg/kg/day methylprednisolone IV or oral equivalent<br>• Consider renal biopsy with nephrology consult	If returns to Grade 1:<br>•Taper steroids over at least 1 month, consider prophylactic antibiotics for opportunistic infections, and resume I-O therapy and routine creatinine monitoring per protocol<br>If elevations persist > 7 days or worsen:<br>•Treat as Grade 4
**Grade 4**<br>Creatinine > 6x ULN	• Discontinue I-O therapy per protocol<br>• Monitor creatinine daily<br>• 1.0-2.0 mg/kg/day methylprednisolone IV or IV equivalent<br>• Consult nephrologist<br>• Consider renal biopsy	If returns to Grade 1 : Taper steroids over at least 1 month and add prophylactic antibiotics for opportunistic infections

Patients on IV steroids may be switched to an equivalent dose of oral corticosteroids (e.g. prednisone) at start of tapering or earlier, once sustained clinical improvement is observed. Lower bioavailability of oral corticosteroids should be taken into account when switching to the equivalent dose of oral corticosteroids.

Pulmonary Adverse Event Management Algorithm

Rule out non-inflammatory causes. If non-inflammatory cause, treat accordingly and continue I-O therapy. Evaluate with imaging and pulmonary consultation.

Grade of Pneumonitis (NCI CTCAE v4)	Management	Follow-up
**Grade 1**<br>Radiographic changes only	• Consider delay of I-O therapy<br>• Monitor for symptoms every 2-3 days<br>• Consider Pulmonary and ID consults	• Re-image at least every 3 weeks<br><u>If worsens:</u><br>• Treat as Grade 2 or 3-4
**Grade 2**<br>Mild to moderate new symptoms	• Delay I-O therapy per protocol<br>• Pulmonary and ID consults<br>• Monitor symptoms daily, consider hospitalization<br>• 1.0 mg/kg/day methyl-prednisolone IV or oral equivalent<br>• Consider bronchoscopy, lung biopsy	• Re-image every 1-3 days<br><u>If improves</u>:<br>• When symptoms return to near baseline, taper steroids over at least 1 month and then resume I-O therapy per protocol and consider prophylactic antibiotics<br><u>If not improving after 2 weeks or worsening:</u><br>• Treat as Grade 3-4
**Grade 3-4**<br>Severe new symptoms; New/worsening hypoxia; Life-threatening	• Discontinue I-O therapy per protocol<br>• Hospitalize<br>• Pulmonary and ID consults<br>• 2-4 mg/kg/day methylprednisolone IV or IV equivalent<br>• Add prophylactic antibiotics for opportunistic infections<br>• Consider bronchoscopy, lung biopsy	<u>If improves to baseline:</u><br>• Taper steroids over at least 6 weeks<br><u>If not improving after 48 hours or worsening:</u><br>• Add additional immunosuppression

Patients on IV steroids may be switched to an equivalent dose of oral corticosteroids (e.g. prednisone) at start of tapering or earlier, once sustained clinical improvement is observed. Lower bioavailability of oral corticosteroids should be taken into account when switching to the equivalent dose of oral corticosteroids.

Hepatic Adverse Event Management Algorithm

Rule out non-inflammatory causes. If non-inflammatory cause, treat accordingly and continue I-O therapy. Consider imaging for obstruction.

Grade of Liver Test Elevation (NCI CTCAE v4)	Management	Follow-up
**Grade 1**<br>AST or ALT > ULN to 3.0 x ULN <u>and/or</u> T. bili > ULN to 1.5 x ULN	• Continue I-O therapy per protocol	• Continue LFT monitoring per protocol<br><u>If worsens:</u><br>• Treat as Grade 2 or 3-4
**Grade 2**<br>AST or ALT > 3.0 to ≤ 5 x ULN <u>and/or</u> T. bili > 1.5 to ≤ 3 x ULN	• Delay I-O therapy per protocol<br>• Increase frequency of monitoring to every 3 days	<u>If returns to baseline</u>:<br>•Resume routine monitoring, resume I-O therapy per protocol<br><br><u>If elevations persist > 5-7 days or worsen :</u><br>• 0.5-1 mg/kg/day methylprednisolone or oral equivalent and when LFT returns to grade 1 or baseline, taper steroids over at least 1 month, consider prophylactic antibiotics for opportunistic infections, and resume I-O therapy per protocol
**Grade 3-4**<br>AST or ALT > 5 x ULN <u>or</u> T.bili >3 x ULN	• Discontinue I-O therapy*<br>• Increase frequency of monitoring to every 1-2 days<br>• 1.0 to 2.0 mg/kg/day methylprednisolone IV or IV equivalent**<br>• Add prophylactic antibiotics for opportunistic infections<br>• Consult gastroenterologist	<u>If returns to grade 2</u>:<br>• Taper steroids over at least 1 month<br><br><u>If does not improve in >3-5 days, worsens or rebounds:</u><br>• Add mycophenolate mofetil 1 g BID<br>• If no response within an additional 3-5 days, consider other immunosuppressants per local guidelines

Patients on IV steroids may be switched to an equivalent dose of oral corticosteroids (e.g. prednisone) at start of tapering or earlier, once sustained clinical improvement is observed. Lower bioavailability of oral corticosteroids should be taken into account when switching to the equivalent dose of oral corticosteroids.

***I-O therapy may be delayed rather than discontinued if AST/ALT ≤ 8 x ULN or T.bili ≤ 5 x ULN.**

**The recommended starting dose for grade 4 hepatitis is 2 mg/kg/day methylprednisolone IV.

Endocrinopathy Management Algorithm

Rule out non-inflammatory causes. If non-inflammatory cause, treat accordingly and continue I-O therapy. Consider visual field testing, endocrinology consultation, and imaging.

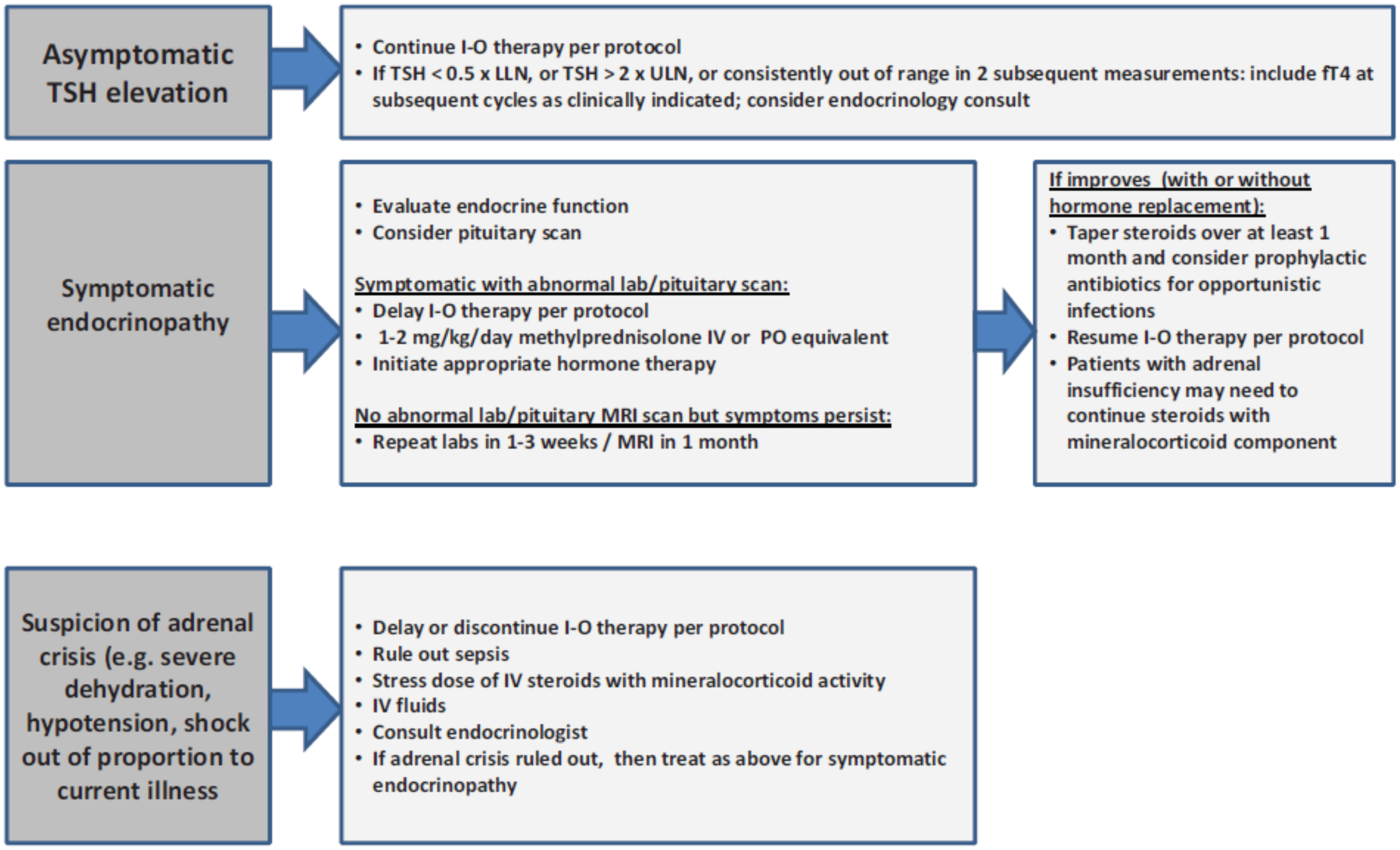

Patients on IV steroids may be switched to an equivalent dose of oral corticosteroids (e.g. prednisone) at start of tapering or earlier, once sustained clinical improvement is observed. Lower bioavailability of oral corticosteroids should be taken into account when switching to the equivalent dose of oral corticosteroids.

Skin Adverse Event Management Algorithm

Rule out non-inflammatory causes. If non-inflammatory cause, treat accordingly and continue I-O therapy.

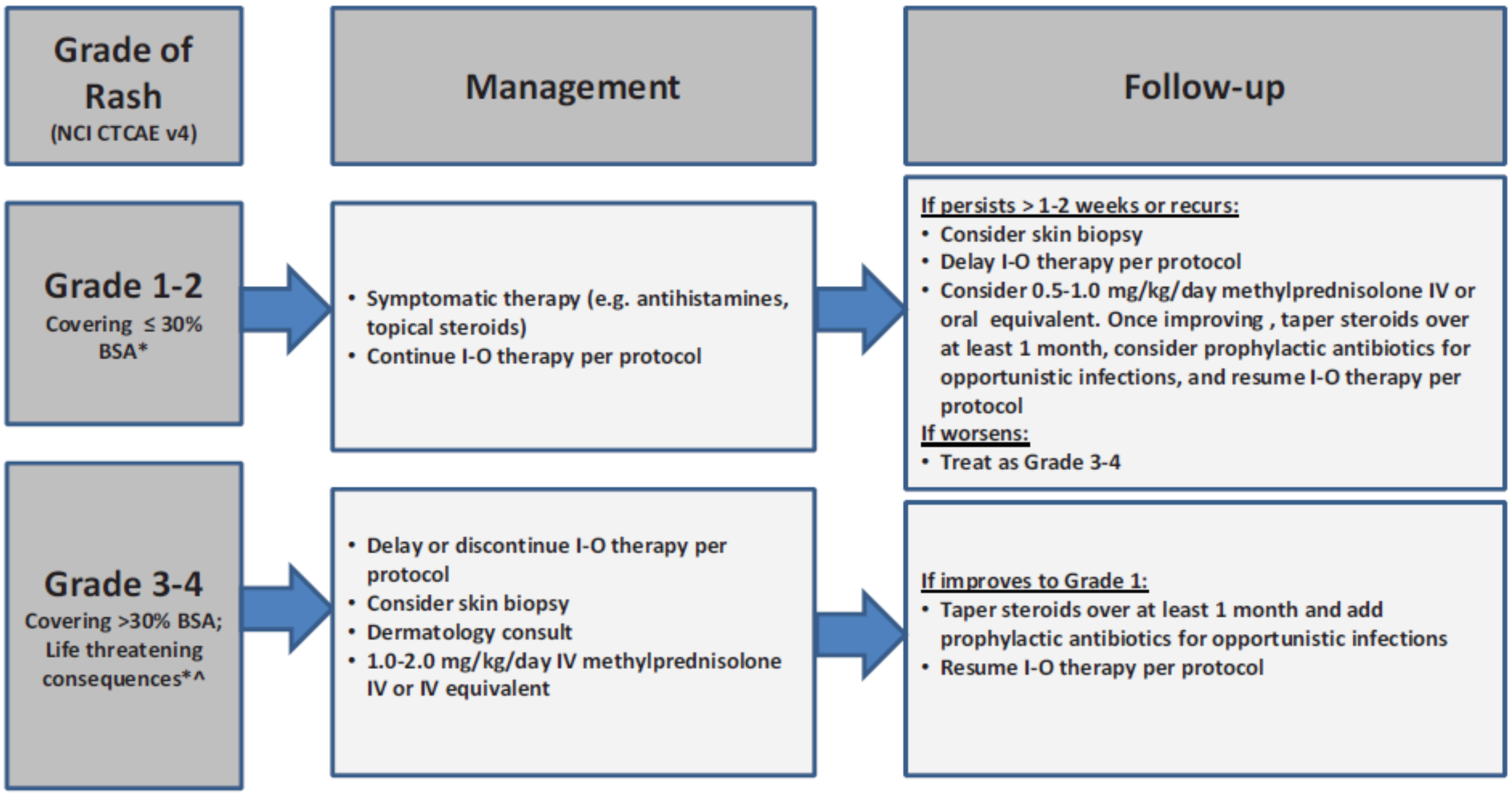

Patients on IV steroids may be switched to an equivalent dose of oral corticosteroids (e.g. prednisone) at start of tapering or earlier, once sustained clinical improvement is observed. Lower bioavailability of oral corticosteroids should be taken into account when switching to the equivalent dose of oral corticosteroids.
*Refer to NCI CTCAE v4 for term-specific grading criteria.
^If SJS/TEN is suspected, withhold I-O therapy and refer patient for specialized care for assessment and treatment. If SJS or TEN is diagnosed, permanently discontinue I-O therapy.

Neurological Adverse Event Management Algorithm

Rule out non-inflammatory causes. If non-inflammatory cause, treat accordingly and continue I-O therapy.

Grade of Neurological Toxicity (NCI CTCAE v4)	Management	Follow-up
**Grade 1**<br>Asymptomatic or mild symptoms; Intervention not indicated	• Continue I-O therapy per protocol	Continue to monitor the patient.<br><u>If worsens:</u><br>• Treat as Grade 2 or 3-4
**Grade 2**<br>Moderate symptoms; Limiting instrumental ADL	• Delay I-O therapy per protocol<br>• Treat symptoms per local guidelines<br>• Consider 0.5 to 1.0 mg/kg/day methylprednisolone IV or PO equivalent	<u>If improves to baseline</u>:<br>•Resume I-O therapy per protocol when improved to baseline<br><u>If worsens</u>:<br>• Treat as Grade 3-4
**Grade 3-4**<br>Severe symptoms; Limiting self-care ADL; Life-threatening	• Discontinue I-O therapy per protocol<br>• Obtain neurology consult<br>• Treat symptoms per local guidelines<br>• 1.0-2.0 mg/kg/day IV methylprednisolone IV or IV equivalent<br>• Add prophylactic antibiotics for opportunistic infections	<u>If improves to Grade 2</u>:<br>• Taper steroids over at least 1 month<br><u>If worsens or atypical presentation:</u><br>• Consider IVIG or other immunosuppressive therapies per local guidelines

Patients on IV steroids may be switched to an equivalent dose of oral corticosteroids (e.g. prednisone) at start of tapering or earlier, once sustained clinical improvement is observed. Lower bioavailability of oral corticosteroids should be taken into account when switching to the equivalent dose of oral corticosteroids.

<u>Ipilimumab</u>

These general guidelines constitute guidance to the Investigator and may be supplemented by discussions with the Sponsor-Investigator. This guidance will be utilized for ipilimumab.

• Where applicable the Approved Label should be used for guidance around dose modifications and discontinuation

• A general principle is that differential diagnoses should be diligently evaluated according to standard medical practice. Non-inflammatory etiologies should be considered and appropriately treated.

• Corticosteroids are a primary therapy for immuno-oncology drug-related adverse events.

The oral equivalent of the recommended IV doses may be considered for ambulatory patients with low-grade toxicity. The lower bioavailability of oral corticosteroids should be taken into account when switching to the equivalent dose of oral corticosteroids.

• Consultation with a medical or surgical specialist, especially prior to an invasive diagnostic or therapeutic procedure, is recommended.

• The frequency and severity of the related adverse events covered by these algorithms will depend on the immuno-oncology agent or regimen being used.

• Investigators should refer to the most current version of the IB or Approved Label for current recommendations for management of a specific Adverse Event of interest.

• On the next 6 pages, find Ipilimumab Management Algorithms for the following AEs:
o GI AEs
o Renal AEs
o Pulmonary AEs
o Hepatic AEs
o Skin AEs
o Endocrinopathy AEs
o Neurological AEs
o Myocarditis AEs

GI Adverse Event Management Algorithm

Rule out non-inflammatory causes. If non-inflammatory cause is identified, treat accordingly and continue I-O therapy. Opiates/narcotics may mask symptoms of perforation. Infliximab should not be used in cases of perforation or sepsis.

Grade of Diarrhea/ Colitis (NCI CTCAE v4)	Management	Follow-up
**Grade 1**<br>Diarrhea: < 4 stools/day over baseline;<br>Colitis: asymptomatic	• Continue I-O therapy per protocol<br>• Symptomatic treatment	• Close monitoring for worsening symptoms.<br>• Educate patient to report worsening immediately<br>If worsens:<br>• Treat as Grade 2 or 3/4
**Grade 2**<br>Diarrhea: 4-6 stools per day over baseline; IV fluids indicated <24 hrs; not interfering with ADL<br>Colitis: abdominal pain; blood in stool	• Delay I-O therapy per protocol<br>• Symptomatic treatment	If improves to grade 1:<br>• Resume I-O therapy per protocol<br>If persists > 5-7 days or recur:<br>• 0.5-1.0 mg/kg/day methylprednisolone or oral equivalent<br>• When symptoms improve to grade 1, taper steroids over at least 1 month, consider prophylactic antibiotics for opportunistic infections, and resume I-O therapy per protocol.<br>If worsens or persists > 3-5 days with oral steroids:<br>• Treat as grade 3/4
**Grade 3-4**<br>Diarrhea (G3): ≥7 stools per day over baseline; incontinence; IV fluids ≥24 hrs; interfering with ADL<br>Colitis (G3): severe abdominal pain, medical intervention indicated, peritoneal signs<br>G4: life-threatening, perforation	• Discontinue I-O therapy per protocol<br>• 1.0 to 2.0 mg/kg/day methylprednisolone IV or IV equivalent<br>• Add prophylactic antibiotics for opportunistic infections<br>• Consider lower endoscopy	If improves:<br>• Continue steroids until grade 1, then taper over at least 1 month<br>If persists > 3-5 days, or recurs after improvement:<br>• Add infliximab 5 mg/kg (if no contraindication). Note: Infliximab should not be used in cases of perforation or sepsis

Patients on IV steroids may be switched to an equivalent dose of oral corticosteroids (e.g. prednisone) at start of tapering or earlier, once sustained clinical improvement is observed. Lower bioavailability of oral corticosteroids should be taken into account when switching to the equivalent dose of oral corticosteroids.

Renal Adverse Event Management Algorithm

Rule out non-inflammatory causes. If non-inflammatory cause, treat accordingly and continue I-O therapy.

Grade of Creatinine Elevation (NCI CTCAE v4)	Management	Follow-up
**Grade 1** Creatinine > ULN and > than baseline but ≤ 1.5x baseline	• Continue I-O therapy per protocol<br>• Monitor creatinine weekly	If returns to baseline:<br>•Resume routine creatinine monitoring per protocol<br>If worsens:<br>•Treat as Grade 2 or 3/4
**Grade 2-3** Creatinine > 1.5x baseline to ≤ 6x ULN	• Delay I-O therapy per protocol<br>• Monitor creatinine every 2-3 days<br>• 0.5 to 1.0 mg/kg/day methylprednisolone IV or oral equivalent<br>• Consider renal biopsy with nephrology consult	If returns to Grade 1:<br>•Taper steroids over at least 1 month, consider prophylactic antibiotics for opportunistic infections, and resume I-O therapy and routine creatinine monitoring per protocol<br>If elevations persist > 7 days or worsen:<br>•Treat as Grade 4
**Grade 4** Creatinine > 6x ULN	• Discontinue I-O therapy per protocol<br>• Monitor creatinine daily<br>• 1.0-2.0 mg/kg/day methylprednisolone IV or IV equivalent<br>• Consult nephrologist<br>• Consider renal biopsy	If returns to Grade 1 : Taper steroids over at least 1 month and add prophylactic antibiotics for opportunistic infections

Patients on IV steroids may be switched to an equivalent dose of oral corticosteroids (e.g. prednisone) at start of tapering or earlier, once sustained clinical improvement is observed. Lower bioavailability of oral corticosteroids should be taken into account when switching to the equivalent dose of oral corticosteroids.

Pulmonary Adverse Event Management Algorithm

Rule out non-inflammatory causes. If non-inflammatory cause, treat accordingly and continue I-O therapy. Evaluate with imaging and pulmonary consultation.

Grade of Pneumonitis (NCI CTCAE v4)	Management	Follow-up
**Grade 1** Radiographic changes only	• Consider delay of I-O therapy<br>• Monitor for symptoms every 2-3 days<br>• Consider Pulmonary and ID consults	• Re-image at least every 3 weeks<br>If worsens:<br>• Treat as Grade 2 or 3-4
**Grade 2** Mild to moderate new symptoms	• Delay I-O therapy per protocol<br>• Pulmonary and ID consults<br>• Monitor symptoms daily, consider hospitalization<br>• 1.0 mg/kg/day methyl-prednisolone IV or oral equivalent<br>• Consider bronchoscopy, lung biopsy	• Re-image every 1-3 days<br>If improves:<br>• When symptoms return to near baseline, taper steroids over at least 1 month and then resume I-O therapy per protocol and consider prophylactic antibiotics<br>If not improving after 2 weeks or worsening:<br>• Treat as Grade 3-4
**Grade 3-4** Severe new symptoms; New/worsening hypoxia; Life-threatening	• Discontinue I-O therapy per protocol<br>• Hospitalize<br>• Pulmonary and ID consults<br>• 2-4 mg/kg/day methylprednisolone IV or IV equivalent<br>• Add prophylactic antibiotics for opportunistic infections<br>• Consider bronchoscopy, lung biopsy	If improves to baseline:<br>• Taper steroids over at least 6 weeks<br>If not improving after 48 hours or worsening:<br>• Add additional immunosuppression

Patients on IV steroids may be switched to an equivalent dose of oral corticosteroids (e.g. prednisone) at start of tapering or earlier, once sustained clinical improvement is observed. Lower bioavailability of oral corticosteroids should be taken into account when switching to the equivalent dose of oral corticosteroids

Hepatic Adverse Event Management Algorithm

Rule out non-inflammatory causes. If non-inflammatory cause, treat accordingly and continue I-O therapy. Consider imaging for obstruction.

Grade of Liver Test Elevation (NCI CTCAE v4)	Management	Follow-up
**Grade 1**<br>AST or ALT > ULN to 3.0 x ULN <u>and/or</u> T. bili > ULN to 1.5 x ULN	• Continue I-O therapy per protocol	• Continue LFT monitoring per protocol<br><u>If worsens:</u><br>• Treat as Grade 2 or 3-4
**Grade 2**<br>AST or ALT > 3.0 to ≤ 5 x ULN <u>and/or</u> T. bili > 1.5 to ≤ 3 x ULN	• Delay I-O therapy per protocol<br>• Increase frequency of monitoring to every 3 days	<u>If returns to baseline:</u><br>•Resume routine monitoring, resume I-O therapy per protocol<br><br><u>If elevations persist > 5-7 days or worsen :</u><br>• 0.5-1 mg/kg/day methylprednisolone or oral equivalent and when LFT returns to grade 1 or baseline, taper steroids over at least 1 month, consider prophylactic antibiotics for opportunistic infections, and resume I-O therapy per protocol
**Grade 3-4**<br>AST or ALT > 5 x ULN <u>or</u> T.bili >3 x ULN	• Discontinue I-O therapy*<br>• Increase frequency of monitoring to every 1-2 days<br>• 1.0 to 2.0 mg/kg/day methylprednisolone IV or IV equivalent*<br>• Add prophylactic antibiotics for opportunistic infections<br>• Consult gastroenterologist	<u>If returns to grade 2:</u><br>• Taper steroids over at least 1 month<br><br><u>If does not improve in >3-5 days, worsens or rebounds:</u><br>• Add mycophenolate mofetil 1 g BID<br>• If no response within an additional 3-5 days, consider other immunosuppressants per local guidelines

Patients on IV steroids may be switched to an equivalent dose of oral corticosteroids (e.g. prednisone) at start of tapering or earlier, once sustained clinical improvement is observed. Lower bioavailability of oral corticosteroids should be taken into account when switching to the equivalent dose of oral corticosteroids.

*The recommended starting dose for grade 4 hepatitis is 2 mg/kg/day methylprednisolone IV.

Skin Adverse Event Management Algorithm

Rule out non-inflammatory causes. If non-inflammatory cause, treat accordingly and continue I-O therapy.

Grade of Rash (NCI CTCAE v4)	Management	Follow-up
**Grade 1-2** Covering ≤ 30% BSA*	• Symptomatic therapy (e.g. antihistamines, topical steroids)<br>• Continue I-O therapy per protocol	<u>If persists > 1-2 weeks or recurs:</u><br>• Consider skin biopsy<br>• Delay I-O therapy per protocol<br>• Consider 0.5-1.0 mg/kg/day methylprednisolone IV or oral equivalent. Once improving , taper steroids over at least 1 month, consider prophylactic antibiotics for opportunistic infections, and resume I-O therapy per protocol<br><u>If worsens:</u><br>• Treat as Grade 3-4
**Grade 3-4** Covering >30% BSA; Life threatening consequences*^	• Delay or discontinue I-O therapy per protocol<br>• Consider skin biopsy<br>• Dermatology consult<br>• 1.0-2.0 mg/kg/day IV methylprednisolone IV or IV equivalent	<u>If improves to Grade 1:</u><br>• Taper steroids over at least 1 month and add prophylactic antibiotics for opportunistic infections<br>• Resume I-O therapy per protocol

Patients on IV steroids may be switched to an equivalent dose of oral corticosteroids (e.g. prednisone) at start of tapering or earlier, once sustained clinical improvement is observed. Lower bioavailability of oral corticosteroids should be taken into account when switching to the equivalent dose of oral corticosteroids.

*Refer to NCI CTCAE v4 for term-specific grading criteria.

^If SJS/TEN is suspected, withhold I-O therapy and refer patient for specialized care for assessment and treatment. If SJS or TEN is diagnosed, permanently discontinue I-O therapy.

Endocrinopathy Adverse Event Management Algorithm

Rule out non-inflammatory causes. If non-inflammatory cause, treat accordingly and continue I-O therapy. Consider visual field testing, endocrinology consultation, and imaging.

Asymptomatic TSH elevation →

- Continue I-O therapy per protocol
- If TSH < 0.5 x LLN, or TSH > 2 x ULN, or consistently out of range in 2 subsequent measurements: include fT4 at subsequent cycles as clinically indicated; consider endocrinology consult

Symptomatic endocrinopathy →

- Evaluate endocrine function
- Consider pituitary scan

Symptomatic with abnormal lab/pituitary scan:
- Delay I-O therapy per protocol
- 1-2 mg/kg/day methylprednisolone IV or PO equivalent
- Initiate appropriate hormone therapy

No abnormal lab/pituitary MRI scan but symptoms persist:
- Repeat labs in 1-3 weeks / MRI in 1 month

→ If improves (with or without hormone replacement):
- Taper steroids over at least 1 month and consider prophylactic antibiotics for opportunistic infections
- Resume I-O therapy per protocol
- Patients with adrenal insufficiency may need to continue steroids with mineralocorticoid component

Suspicion of adrenal crisis (e.g. severe dehydration, hypotension, shock out of proportion to current illness →

- Delay or discontinue I-O therapy per protocol
- Rule out sepsis
- Stress dose of IV steroids with mineralocorticoid activity
- IV fluids
- Consult endocrinologist
- If adrenal crisis ruled out, then treat as above for symptomatic endocrinopathy

Patients on IV steroids may be switched to an equivalent dose of oral corticosteroids (e.g. prednisone) at start of tapering or earlier, once sustained clinical improvement is observed. Lower bioavailability of oral corticosteroids should be taken into account when switching to the equivalent dose of oral corticosteroids.

Neurological Adverse Event Management Algorithm

Rule out non-inflammatory causes. If non-inflammatory cause, treat accordingly and continue I-O therapy.

Grade of Neurological Toxicity (NCI CTCAE v4)	Management	Follow-up
**Grade 1**<br>Asymptomatic or mild symptoms; Intervention not indicated	• Continue I-O therapy per protocol	Continue to monitor the patient.<br>If worsens:<br>• Treat as Grade 2 or 3-4
**Grade 2**<br>Moderate symptoms; Limiting instrumental ADL	• Delay I-O therapy per protocol<br>• Treat symptoms per local guidelines<br>• Consider 0.5 to 1.0 mg/kg/day methylprednisolone IV or PO equivalent	If improves to baseline:<br>•Resume I-O therapy per protocol when improved to baseline<br>If worsens:<br>• Treat as Grade 3-4
**Grade 3-4**<br>Severe symptoms; Limiting self-care ADL; Life-threatening	• Discontinue I-O therapy per protocol<br>• Obtain neurology consult<br>• Treat symptoms per local guidelines<br>• 1.0-2.0 mg/kg/day IV methylprednisolone IV or IV equivalent<br>• Add prophylactic antibiotics for opportunistic infections	If improves to Grade 2:<br>• Taper steroids over at least 1 month<br><br>If worsens or atypical presentation:<br>• Consider IVIG or other immunosuppressive therapies per local guidelines

Patients on IV steroids may be switched to an equivalent dose of oral corticosteroids (e.g. prednisone) at start of tapering or earlier, once sustained clinical improvement is observed. Lower bioavailability of oral corticosteroids should be taken into account when switching to the equivalent dose of oral corticosteroids.

Myocarditis Adverse Event Management Algorithm

Grade of Myocarditis (NCI CTCAE v4)	Management	Follow-up
**Grade 2**<br><br>Symptoms with mild to moderate activity or exertion	• Delay I-O therapy; hospitalization with cardiac monitoring<br>• Urgent cardiology consultation for evaluation and management<br>o Troponin and BNP<br>o ECG ± continuous cardiac monitoring<br>o Echocardiogram<br>o Cardiac MRI<br>• Prompt initiation of 2 mg/kg/day methylprednisolone IV or equivalent	• If worsens, intensify treatment according to grade<br>• Upon recovery, taper steroids over at least 1 month with close monitoring of troponin and BNP as well as for new symptoms<br>• Repeat cardiac MRI for post treatment assessment and cardiology follow-up<br>• Retreatment may be considered after recovery and completion of steroid taper
**Grade 3**: Severe with symptoms at rest or with minimal activity or exertion; intervention indicated<br><br>**Grade 4**: Life-threatening consequences; urgent intervention indicated (e.g., continuous IV therapy or mechanical hemodynamic support)	• Permanently discontinue I-O therapy<br>• Hospitalize to intensive cardiac monitoring<br>• Cardiac evaluation to include:<br>– Troponin and BNP monitoring<br>– ECG ± continuous cardiac monitoring<br>– Echocardiogram<br>– Cardiac MRI<br>– Myocardial biopsy if feasible<br>• Immediate initiation of 2 mg/kg/day methylprednisolone IV or 1 g IV bolus<br>• Consider adding a second immunosuppressive agent<br>Additionally, for Grade 4:<br>• Hospitalize/transfer to institution with expertise in intensive cardiac monitoring<br>• Consider ATG as second agent given its immediate effect	• If no improvement, consider additional immunosuppression<br><br>• Upon recovery, taper steroids over at least 1 month with close monitoring of troponin and BNP as well as for new symptoms<br><br>• Repeat cardiac MRI for post treatment assessments and cardiology follow-up

Patients on IV steroids may be switched to an equivalent dose of oral corticosteroids (eg, prednisone) at start of tapering or earlier, once sustained clinical improvement is observed. Lower bioavailability of oral corticosteroids should be taken into account when switching to the equivalent dose of oral corticosteroids.
Prophylactic antibiotics should be considered in the setting of ongoing immunosuppression.
ATG = anti-thymocyte globulin; BNP = B-type natriuretic peptide; ECG = electrocardiogram; IV = intravenous; MRI = magnetic resonance imaging

APPENDIX D REPORTABLE AE COVERSHEET

DF/HCC Protocol No. **20-494** BMS Protocol No. **CA209-7GR**
Date: _______________ No. of Pages (including coversheet): ___

To:
☐ Patrick Wen, MD (Sponsor-Investigator) and DFCI Coordinating Center at **NeuroOnc_SAE@dfci.harvard.edu**
☐ BMS Global Safety at **Worldwide.Safety@BMS.com** or via fax at +1 609-818-3804
check off recipient(s) above and include this coversheet with completed MedWatch
NOTE: use additional coversheet(s) if more than 3 events are being reported

**From:**	**Institution:**
**Phone:**	**Fax:**
**Participant No. and Initials:**	**Date Event Met Reporting Criteria (per protocol):**
**Type of Report**: ☐ Initial ☐ Follow-up	**Will this event be reported you your local IRB?** ☐ Yes ☐ No

Event #1 Description (CTCAE v4.0 Term):

Meets Definition of Serious?	Toxicity Grade	Attribution to Nivolumab	Historical/Known Correlation to Nivolumab	Attribution to Ipilimumab	Historical/Known Correlation to Ipilimumab
☐ Serious<br>☐ Not Serious	☐ G1: Mild<br>☐ G2: Moderate<br>☐ G3: Severe<br>☐ G4: Life Threatening<br>☐ G5: Death	☐ Unrelated<br>☐ Unlikely<br>☐ Possible<br>☐ Probable<br>☐ Definite	☐ Expected<br>☐ Unexpected	☐ Unrelated<br>☐ Unlikely<br>☐ Possible<br>☐ Probable<br>☐ Definite	☐ Expected<br>☐ Unexpected

Event #2 Description (CTCAE v4.0 Term):

Meets Definition of Serious?	Toxicity Grade	Attribution to Nivolumab	Historical/Known Correlation to Nivolumab	Attribution to Ipilimumab	Historical/Known Correlation to Ipilimumab
☐ Serious<br>☐ Not Serious	☐ G1: Mild<br>☐ G2: Moderate<br>☐ G3: Severe<br>☐ G4: Life Threatening<br>☐ G5: Death	☐ Unrelated<br>☐ Unlikely<br>☐ Possible<br>☐ Probable<br>☐ Definite	☐ Expected<br>☐ Unexpected	☐ Unrelated<br>☐ Unlikely<br>☐ Possible<br>☐ Probable<br>☐ Definite	☐ Expected<br>☐ Unexpected

Event #3 Description (CTCAE v4.0 Term):

Meets Definition of Serious?	Toxicity Grade	Attribution to Nivolumab	Historical/Known Correlation to Nivolumab	Attribution to Ipilimumab	Historical/Known Correlation to Ipilimumab
☐ Serious<br>☐ Not Serious	☐ G1: Mild<br>☐ G2: Moderate<br>☐ G3: Severe<br>☐ G4: Life Threatening<br>☐ G5: Death	☐ Unrelated<br>☐ Unlikely<br>☐ Possible<br>☐ Probable<br>☐ Definite	☐ Expected<br>☐ Unexpected	☐ Unrelated<br>☐ Unlikely<br>☐ Possible<br>☐ Probable<br>☐ Definite	☐ Expected<br>☐ Unexpected

Reporting Investigator:

Signature of Investigator: ___________________________________ **Date:** __________
