# Supplementary material for: Neoadjuvant PD1 plus CTLA-4 immune checkpoint blockade in surgically resectable recurrent glioblastoma: a randomized surgical window-of-opportunity trial
Source: Nat Commun. 2026 Sep 5;17:10560. doi: 10.1038/s41467-026-77433-z (PMC13642311; doi:10.1038/s41467-026-77433-z)
Supplement: Supplementary file 4 [file 41467_2026_77433_MOESM2_ESM.pdf]

Description of Additional Supplementary Files

Title: Supplementary Data 1

Description: Patient clinical metadata.

Title: Supplementary Data 2

Description: Adverse events.

Title: Supplementary Data 3

Description: GSVA scores of selected gene signatures significantly enriched in tumors from Arm 1 versus Arm 3.

Title: Supplementary Data 4

Description: CD8+, CD4+ T-cell, and lymphocyte/myeloid ratios quantified by mIF.

Title: Supplementary Data 5

Description: Pearson correlations between Ayers et al. IFN genes and immune subset-specific signatures.

Title: Supplementary Data 6

Description: Summary statistics from Cox proportional hazards modeling for selected gene signatures.

Title: Supplementary Data 7

Description: Summary statistics from timepoint comparisons of interferon signaling and immune cell-type signatures.

Title: Supplementary Data 8

Description: Summary statistics from Cox proportional hazards modeling for inflammation- and interferon-related gene signatures in PBMCs at the PostNeo timepoint.

Title: Supplementary Data 9

Description: Differential gene set enrichment analysis of PBMC gene signatures across multiple timepoints comparing trajectories 1 and 2.

Title: Supplementary Data 10

Description: Summary statistics from Cox proportional hazards modeling for genes in PBMCs at baseline.
